# Supplementary material for: Mutation hotspots at CTCF binding sites coupled to chromosomal instability in gastrointestinal cancers
Source: Nat Commun. 2018 Apr 18;9:1520. doi: 10.1038/s41467-018-03828-2 (PMC5906695; doi:10.1038/s41467-018-03828-2)
Supplement: Supplementary file 1 — Supplementary Information [file 41467_2018_3828_MOESM1_ESM.pdf]

Supplementary Information for:

Mutation hotspots at CTCF binding sites coupled to chromosomal instability in gastrointestinal cancers

Yu Amanda Guo[1], Mei Mei Chang[1], Weitai Huang[1,2], Wen Fong Ooi[3], Manjie Xing[3,4], Patrick Tan[4,5] & Anders Jacobsen Skanderup[1]

[1]Computational & Systems Biology, Genome Institute of Singapore, Agency for Science, Technology and Research, Singapore 138672

[2]Graduate School of Integrative Sciences and Engineering, National University of Singapore, Singapore 117456

[3]Cancer Therapeutics and Stratified Oncology, Genome Institute of Singapore, Agency for Science, Technology and Research, Singapore 138672

[4]Cancer and Stem Cell Biology Program, Duke-NUS Medical School, Singapore 169857

[5]Cancer Science Institute of Singapore, National University of Singapore, Singapore 117599

Correspondence should be addressed to A.J.S. (skanderupamj@gis.a-star.edu.sg) and P.T. (tanbop@gis.a-star.edu.sg).

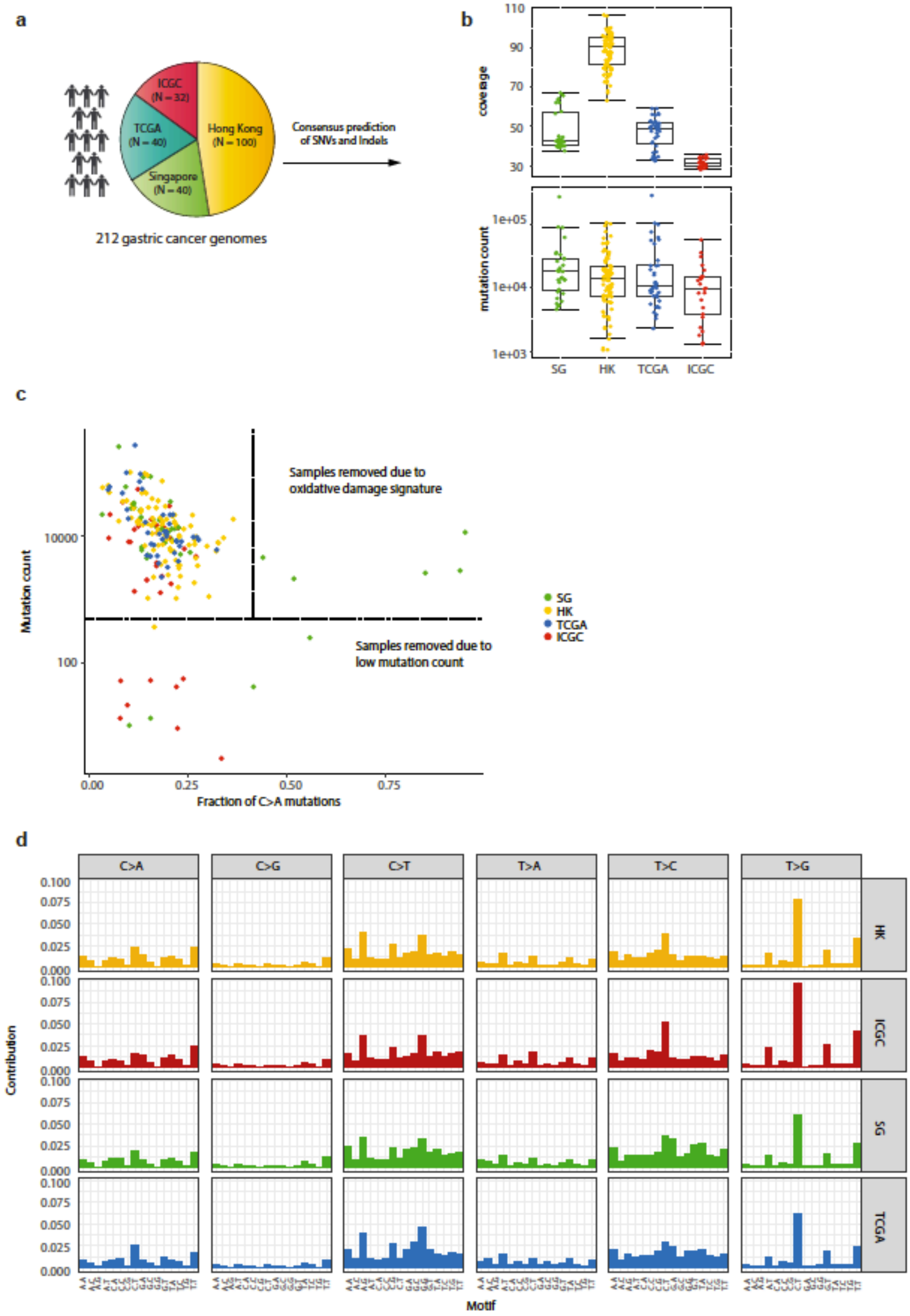
a
ICGC (N = 32)
TCGA (N = 40)
Hong Kong (N = 100)
Singapore (N = 40)
212 gastric cancer genomes
Consensus prediction of SNVs and Indels
b
coverage
mutation count
SG
HK
TCGA
ICGC
c
Samples removed due to oxidative damage signature
Samples removed due to low mutation count
Mutation count
Fraction of C>A mutations
SG
HK
TCGA
ICGC
d
C>A
C>G
C>T
T>A
T>C
T>G
HK
ICGC
SG
TCGA
Contribution
Motif

Supplementary Figure 1 Summary of mutation data of 212 gastric cancer genomes. **(a)** A total of 212 gastric cancer whole genome sequences were collated from 4 sources and uniformly processed to obtain high-confidence somatic mutation calls. **(b)** Mutation count and coverage of individual tumors from the 4 cohorts. **(c)** Individual samples were plotted by their mutation counts on the y-axis against the fractions of C.G>A.T mutations on the x-axis. Samples are colored by their source. Seven samples were removed due to data corruption. Thirteen tumors with low mutation counts were removed, as these are likely low-quality samples. Finally, 5 samples showing signature of oxidative DNA damage (high fraction of C.G>A.T mutations) were removed. **(d)** The mutation spectrums of tumors from the 4 cohorts are similar after uniform alignment and mutation calling.

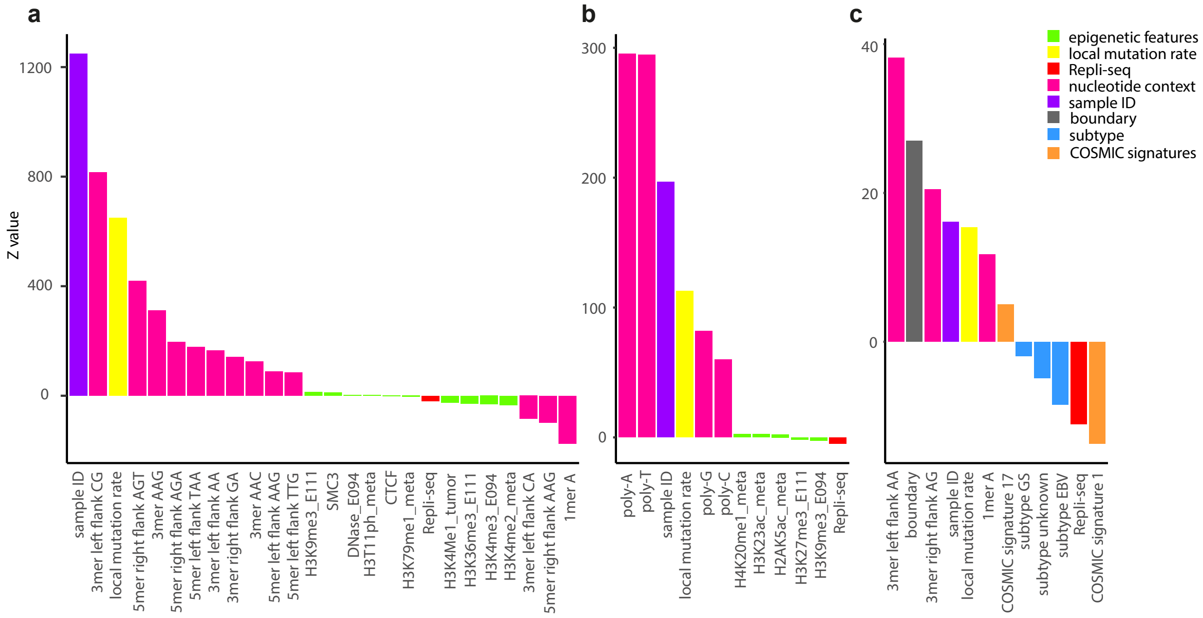

Supplementary Figure 2 Features used in each background mutation model. Sequence and epigenetic features that are most correlated with somatic mutation rates were selected by LASSO regression. Selected features in the **(a)** SNV background model, **(b)** indel background model, and **(c)** CBS-specific background model.

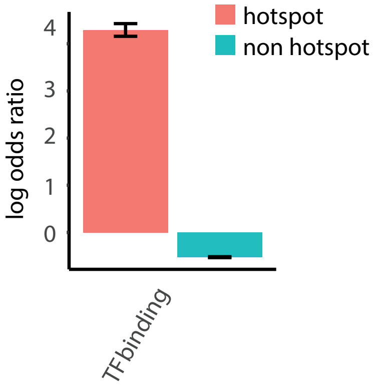

Supplementary Figure 3 Log odds ratio of the enrichment of hotspot mutations and non-hotspot mutations in constitutive transcription factor binding regions. Error bars indicate the s.e.m of the log odds ratio.

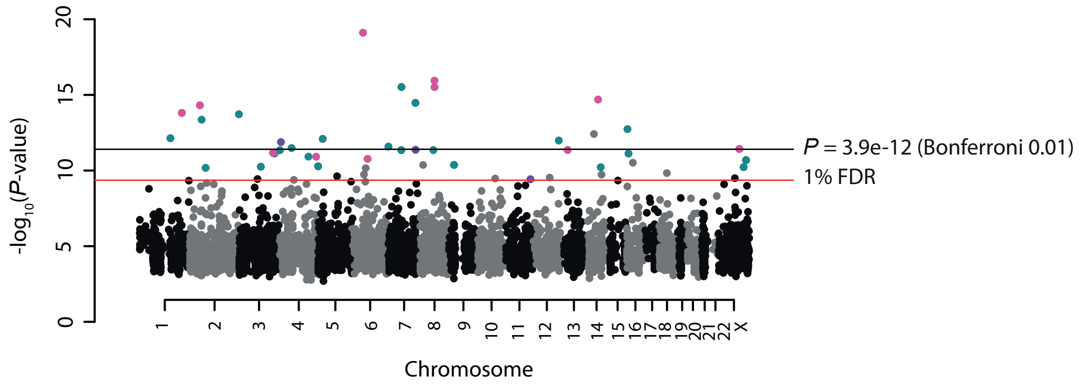

Supplementary Figure 4 Mutation hotspot analysis using 41bp windows. The negative log P-values of SNV recurrence for all 41bp regions genome-wide, only regions with at least 3 mutations are displayed. CBS hotspots previously identified with 21-bp windows are highlight in magenta; Non-CBS hotspots previously identified are highlighted in green. Three additional significantly mutated CBSs identified by the CBS-specific model are highlighted in purple. The horizontal lines mark the Bonferroni adjusted P-values of 0.01 and 1% FDR respectively.

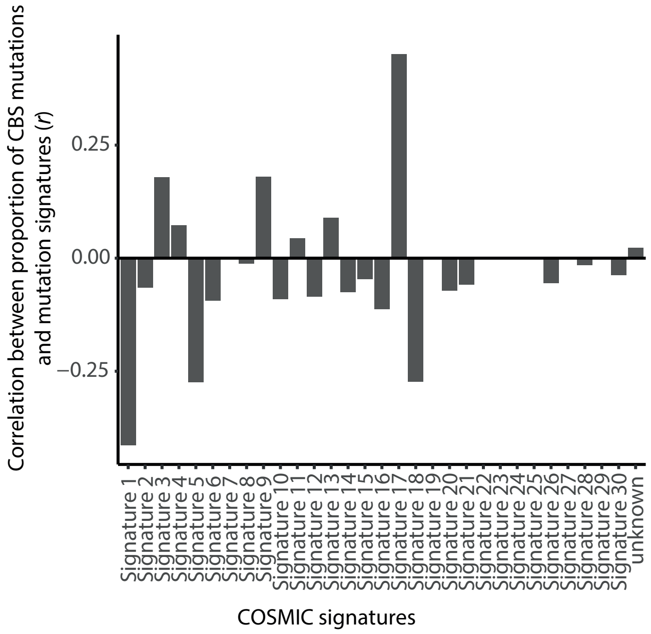

Supplementary Figure 5 Correlation between CBS mutation rate of each sample with COSMIC signatures.

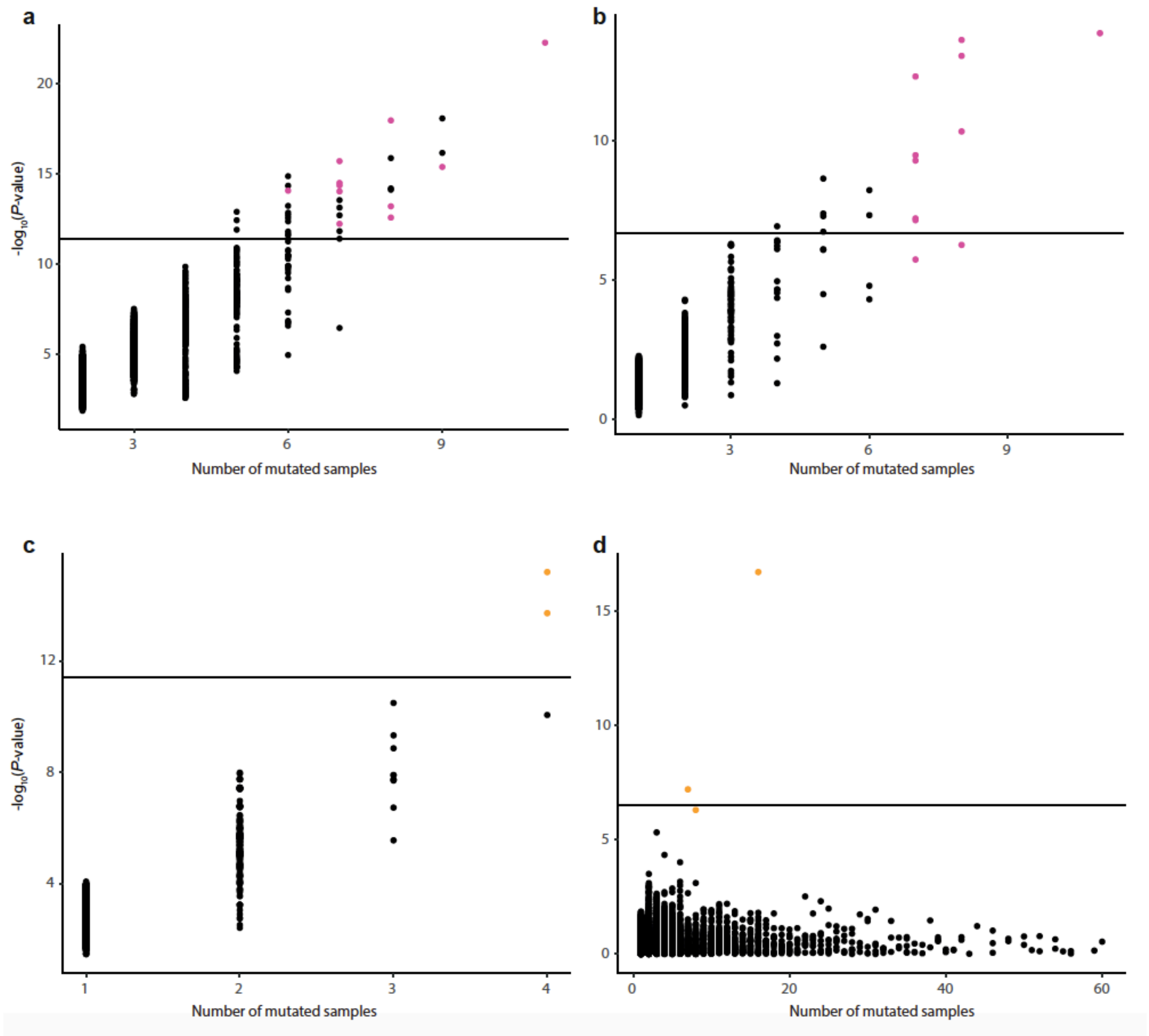

Supplementary Figure 6 Negative log P-values of mutation recurrence plotted against the number of mutated samples in each non-coding region. **(a)** Genome-wide SNV hotspot model. Significantly mutated hotspots overlapping CBSs are highlighted. **(b)** CBS-specific model. CBS hotspots identified in **(a)** are highlighted. **(c)** Genome-wide indel hotspot model. 2 significantly mutated regions are highlighted. **(d)** Gene-based indel recurrence model. 3 significantly mutated genes are highlighted.

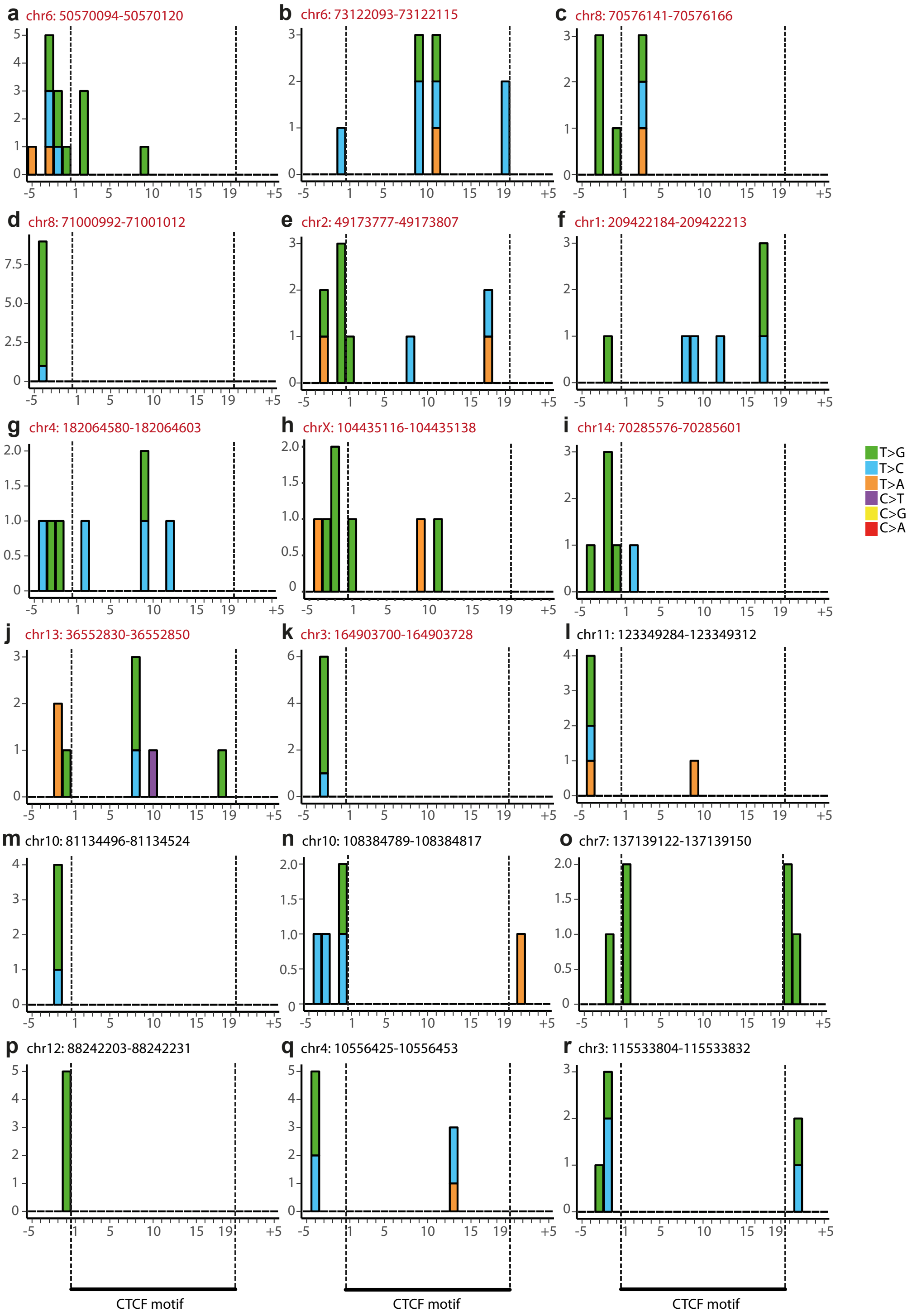

Supplementary Figure 7 Distribution of mutations within each CBS hotspot. **(a-r)** Somatic substitution patterns within each CBS hotspot. CBS hotspots identified from genome-wide analysis of non-coding SNV hotspots are highlighted in red. y-axis shows the mutation count and x-axis shows the position relative to CTCF motif.

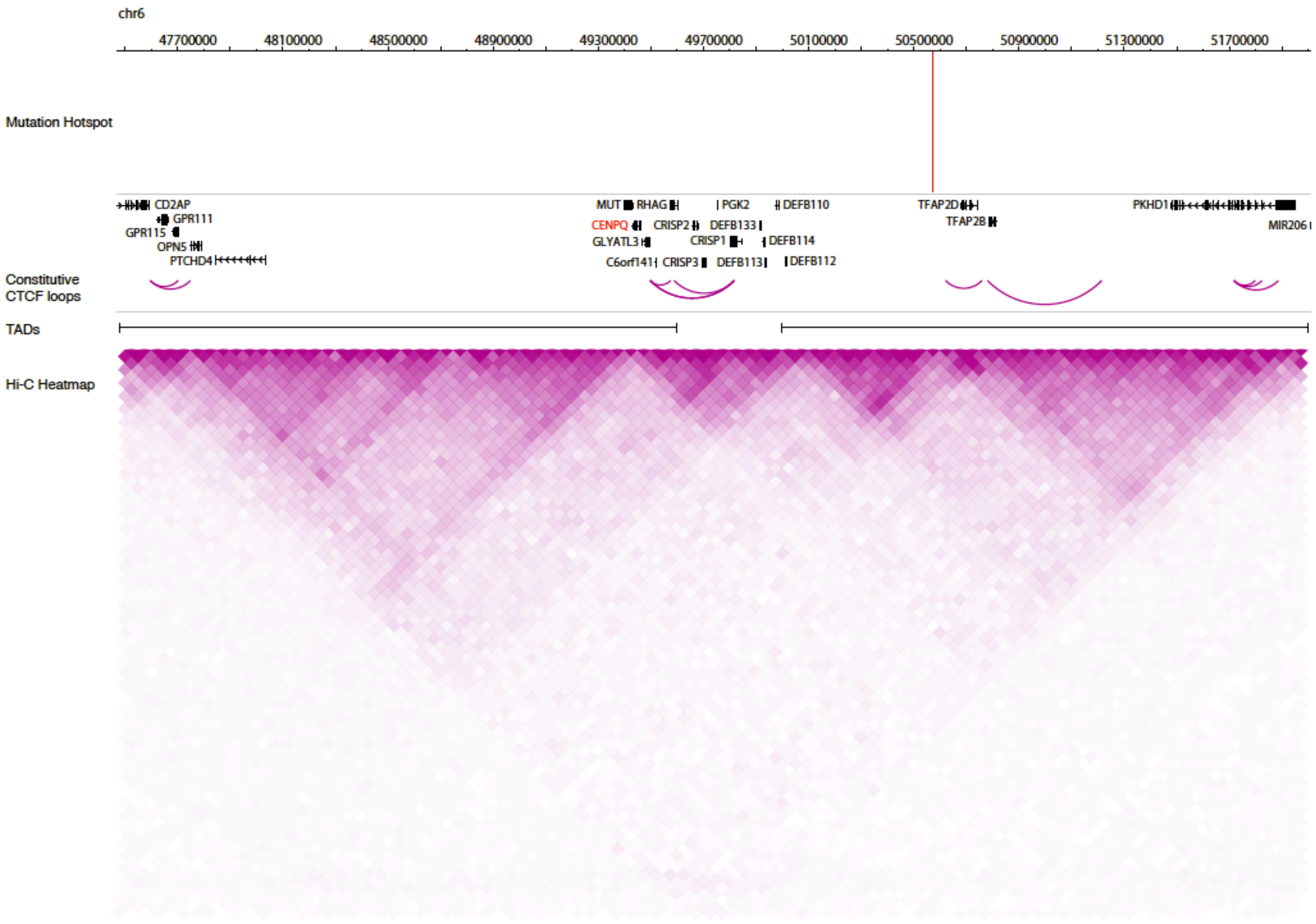

Supplementary Figure 8 Chromatin neighborhood of CBS hotspot at chr6:50570094-50570120. Candidate gene with expression change associated with the mutation status of the hotspot is highlighted in red. The magenta arcs represent constitutive CTCF loops defined by Hnisz et al., *Science*, 2016. The heatmap shows the normalized Hi-C interaction frequencies in IMR90 cells (Dixon et al., *Nature*, 2012). TADs were called by Dixon et al., *Nature*, 2012.

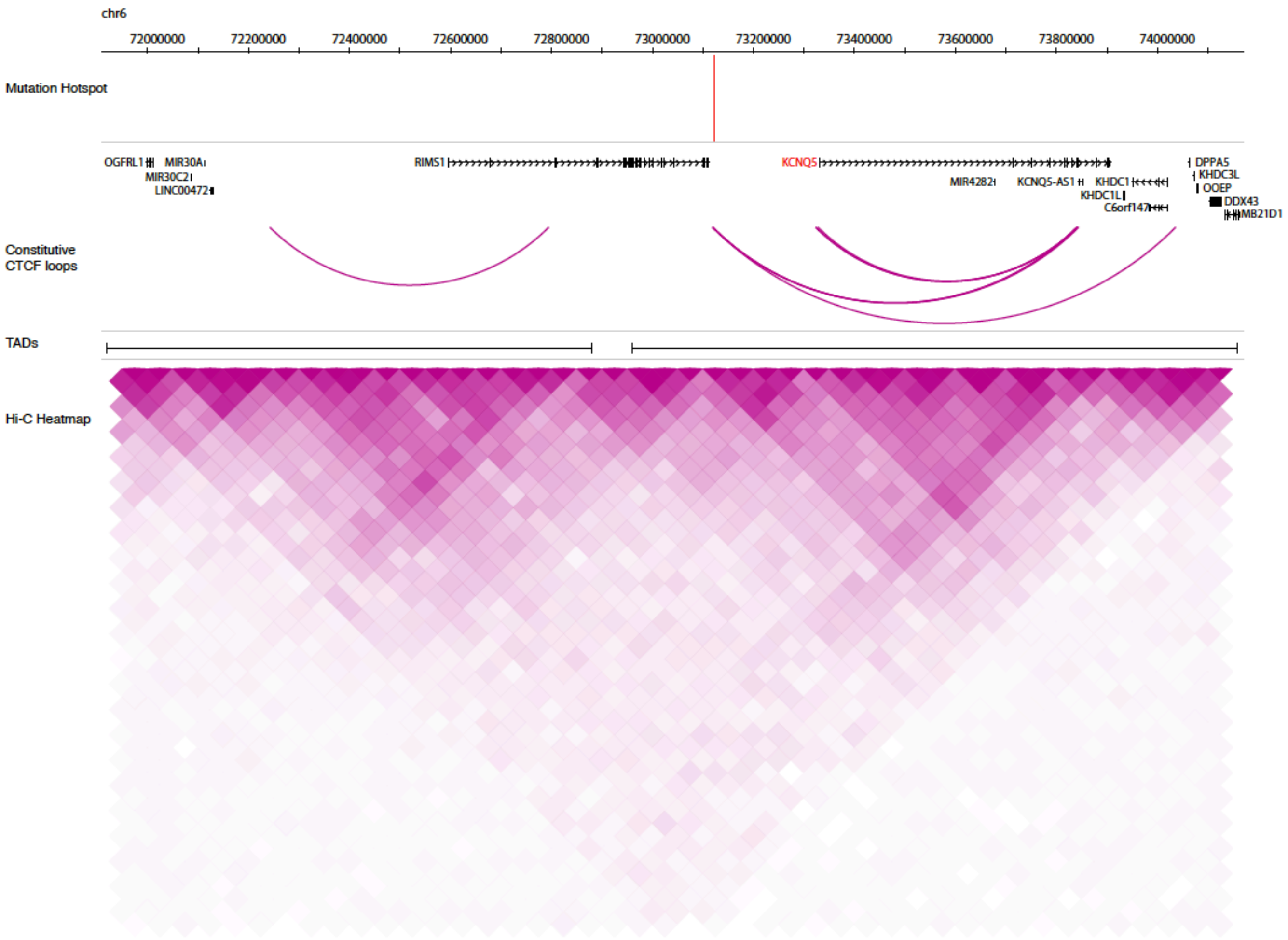

Supplementary Figure 9 Chromatin neighborhood of CBS hotspot at chr6:73122084-73122123. Candidate gene with expression change associated with the mutation status of the hotspot is highlighted in red. The magenta arcs represent constitutive CTCF loops defined by Hnisz et al., *Science*, 2016. The heatmap shows the normalized Hi-C interaction frequencies in IMR90 cells (Dixon et al., *Nature*, 2012). TADs were called by Dixon et al., *Nature*, 2012.

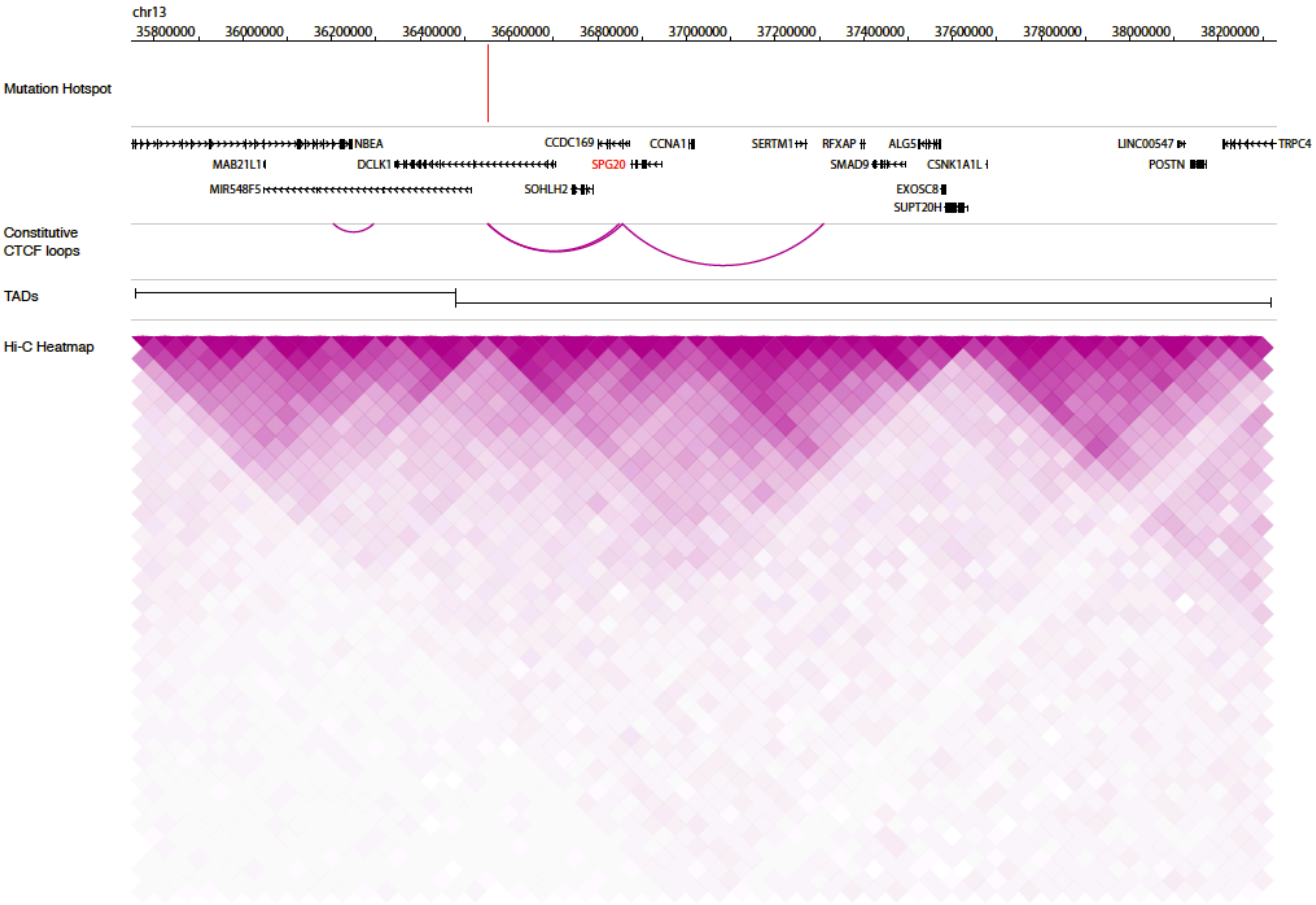

Supplementary Figure 10 Chromatin neighborhood of CBS hotspot at chr13:36552821-36552860. Candidate gene with expression change associated with the mutation status of the hotspot is highlighted in red. The magenta arcs represent constitutive CTCF loop defined by Hnisz et al., *Science*, 2016. The heatmap shows the normalized Hi-C interaction frequencies in IMR90 cells (Dixon et al., *Nature*, 2012). TADs were called by Dixon et al., *Nature*, 2012.

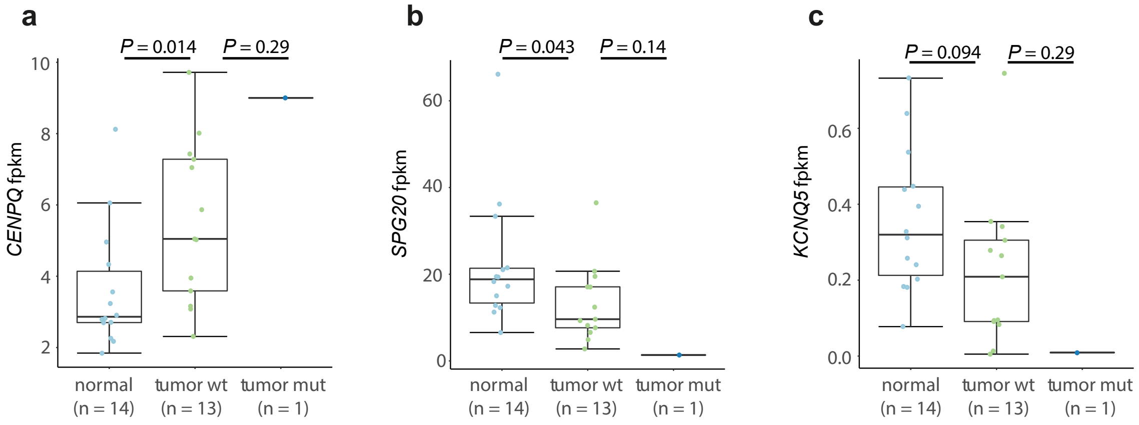

Supplementary Figure 11 Correlation between CBS hotspot mutations and expression of candidate genes using expression data from 14 tumors of the Singapore cohort. (**a-c)** The gene expressions of *CENPQ* **(a)**, *KCNQ5* **(b)** and *SPG20* **(c)** in matched normal gastric tissue, tumors wildtype at the corresponding CBS hotspot and tumors mutated at the corresponding CBS hotspot. Wilcoxon rank-sum test *P*-values are shown.

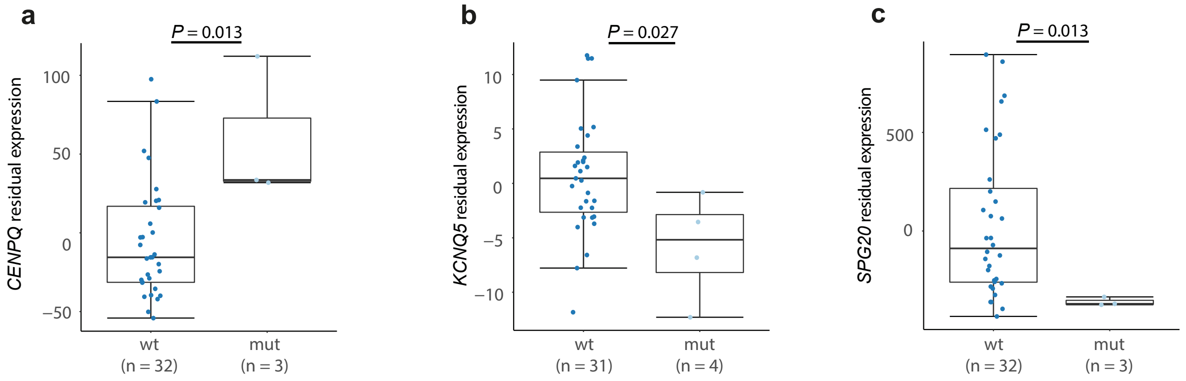

Supplementary Figure 12 Correlation between CBS hotspot mutations and the residual expression of candidate genes after correcting for tumor purity and copy number. (**a-c)** The gene expressions of *CENPQ* **(a)**, *KCNQ5* **(b)** and *SPG20* **(c)** in tumors wildtype at the corresponding CBS hotspot and tumors mutated at the corresponding CBS hotspot. Wilcoxon rank-sum test *P*-values are shown.

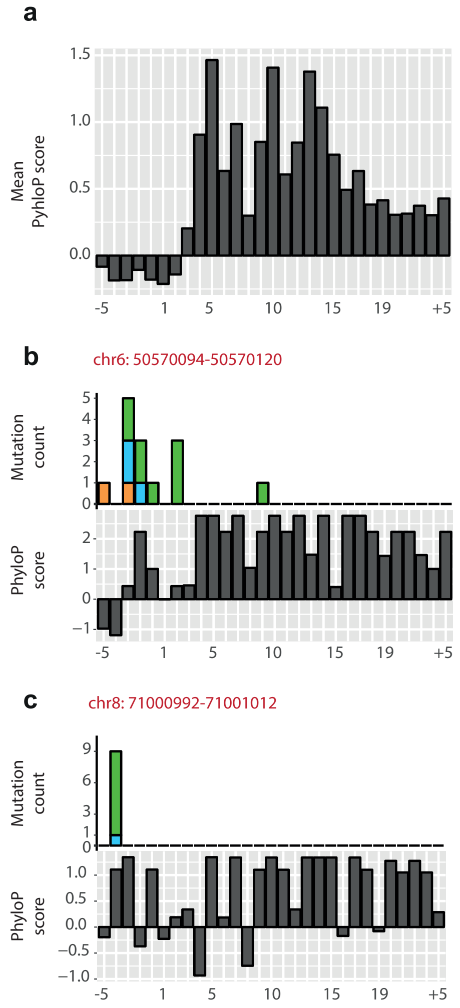

Supplementary Figure 13 Evolutionary conservation of the consensus CTCF motif and flanking sequences. **(a)** Average PhyloP scores of the CTCF-binding motif and ±5 flanking bases of all mutated CBSs. **(b-c)** Two CBS hotspots (b is hotspot upstream of *CENPQ*) where mutations at 5' flanks of CTCF-binding motifs coincide with conserved bases.

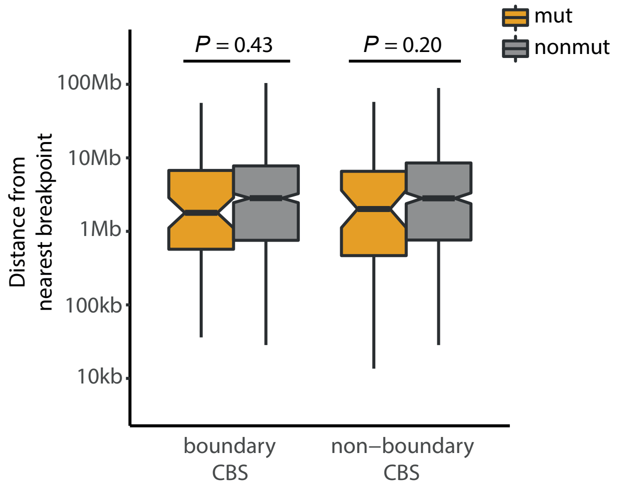

Supplementary Figure 14 Distance to the nearest CNV breakpoint from CBSs at loop boundary and non-boundary CBSs for GS tumors. Wilcoxon rank-sum test *P*-values are shown.

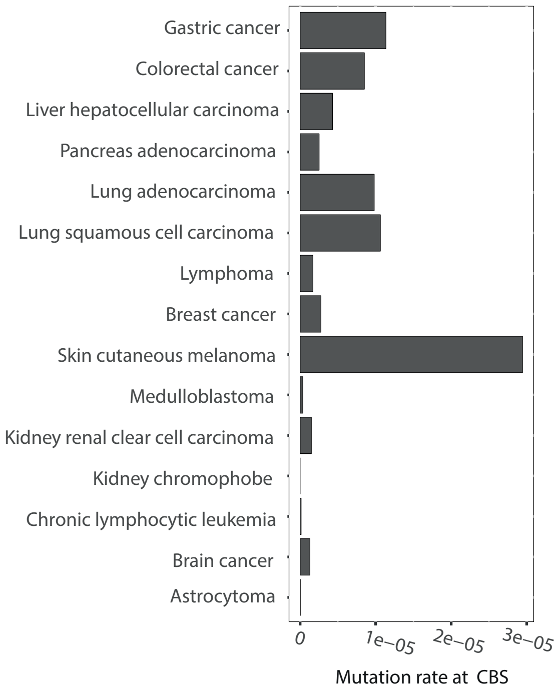

Supplementary Figure 15 Mutation rate of tissue-specific CBSs in different cancer types.

Supplementary Table 1. Indel hotspots. Significantly mutated non-coding indel hotspots identified by a genome-wide scan of 21-bp windows.

**Chr**	**Start**	**End**	***P*-value**	**Length**	**# mutated samples**	**adjusted *P*-value**
chr6	168136120	168136140	6.45E-16	21	4	1.63E-06
chr6	41709379	41709409	1.93E-14	31	4	4.90E-05

Supplementary Table 2. Genes enriched for non-coding indels

**Gene Name**	**Chr**	**Gene Start**	**Gene End**	**Gene Length**	**# mutated samples**	***P*-value**	**Adjusted *P*-value**
LIPF	chr10	90424198	90438571	14807	16	1.89E-17	6.39E-13
PGC	chr6	41704449	41721847	16717	7	6.17E-08	2.08E-03
MUC6	chr11	1012821	1036706	17851	8	4.92E-07	1.66E-02

Supplementary Table 3. SNV hotspots. Significantly mutated non-coding SNV hotspots identified by a genome-wide scan of 21-bp windows.

Chr	Start	End	*P-value*	Length	# mutated samples	Adjusted *P*-value	Annotation
chr6	50570094	50570120	5.40E-23	27	11	1.37E-13	CBS
chr7	68391104	68391132	8.36E-19	29	9	2.12E-09	intergenic
chr8	71000992	71001012	1.09E-18	21	8	2.75E-09	CBS
chr7	136495924	136495948	6.73E-17	25	9	1.71E-07	intergenic
chr2	57627616	57627640	1.32E-16	25	8	3.34E-07	intergenic
chr1	209422184	209422222	1.94E-16	39	7	4.90E-07	CBS
chr2	49173770	49173816	4.05E-16	47	9	1.03E-06	CBS
chr2	239033350	239033370	1.32E-15	21	6	3.35E-06	ESPNL intron
chr4	182064578	182064613	3.09E-15	36	7	7.83E-06	CBS
chrX	104435106	104435140	4.26E-15	35	7	1.08E-05	CBS
chr16	8381278	8381302	4.44E-15	25	6	1.13E-05	intergenic
chr5	23824204	23824224	6.27E-15	21	8	1.59E-05	intergenic
chr7	67614923	67614943	7.42E-15	21	8	1.88E-05	intergenic
chr14	70285576	70285601	8.40E-15	26	6	2.13E-05	CBS
chr6	73122084	73122123	9.16E-15	40	7	2.32E-05	CBS
chr8	65161396	65161420	2.79E-14	25	7	7.08E-05	intergenic
chr7	4937707	4937736	5.80E-14	30	6	1.47E-04	intergenic
chr8	70576141	70576184	6.12E-14	44	8	1.55E-04	CBS
chr12	126996666	126996686	7.21E-14	21	7	1.83E-04	intergenic
chr1	153607104	153607124	1.24E-13	21	5	3.13E-04	CHTOP intron
chr4	5415060	5415082	1.39E-13	23	6	3.52E-04	STK32B intron
chr16	13516145	13516165	1.87E-13	21	6	4.73E-04	intergenic
chrX	137405623	137405655	1.93E-13	33	7	4.88E-04	intergenic
chr13	36552821	36552860	2.57E-13	40	8	6.51E-04	CBS
chr4	62653076	62653096	2.68E-13	21	6	6.80E-04	LPHN3 intron
chr3	171164993	171165017	3.61E-13	25	5	9.15E-04	TNIK intron
chr4	144748744	144748764	4.17E-13	21	6	1.06E-03	intergenic
chr3	164903700	164903728	5.72E-13	29	7	1.45E-03	CBS
chr5	1472143	1472163	1.21E-12	21	5	3.07E-03	LPCAT1 intron
chr9	25481736	25481758	1.44E-12	23	7	3.66E-03	intergenic
chr2	77150455	77150477	1.53E-12	23	6	3.88E-03	LRRTM4 intron
chr3	104801455	104801477	2.12E-12	23	6	5.38E-03	intergenic
chrX	125548690	125548710	2.50E-12	21	6	6.33E-03	intergenic
chr14	83046706	83046744	3.82E-12	39	7	9.67E-03	intergenic

Supplementary Table 4. Recurrently mutated CBSs under the CBS-specific background model

Chr	Start	End	*P*-value	Length	# mutated samples	Adjusted *P*-value
chr6	50570082	50570110	1.32E-14	29	11	6.28E-10
chr8	70576149	70576177	2.31E-14	29	8	1.10E-09
chr8	71000975	71001003	8.65E-14	29	8	4.10E-09
chr14	70285585	70285613	4.75E-13	29	7	2.26E-08
chr6	50570080	50570108	1.22E-12	29	10	5.78E-08
chr2	49173785	49173813	4.48E-11	29	8	2.13E-06
chrX	104435103	104435131	3.21E-10	29	7	1.52E-05
chr3	164903684	164903712	4.98E-10	29	7	2.37E-05
chr3	115533804	115533832	2.22E-09	29	5	1.05E-04
chr4	10556425	10556453	5.78E-09	29	6	2.74E-04
chr12	88242203	88242231	4.01E-08	29	5	1.90E-03
chr7	137139122	137139150	4.59E-08	29	6	2.18E-03
chr10	108384789	108384817	4.97E-08	29	5	2.36E-03
chr1	209422187	209422215	6.00E-08	29	7	2.85E-03
chr6	73122090	73122118	6.92E-08	29	7	3.28E-03
chr10	81134496	81134524	1.15E-07	29	4	5.45E-03
chr11	123349284	123349312	1.80E-07	29	5	8.56E-03

Supplementary Table 5. DeepBind analysis on hotspot mutations flanking CTCF-binding motifs

Hotspot Location	Mutation	Sample ID	Motif creation	Motif disruption
chr2: 49173777-49173807	chr2:49173789 T>G	apollo10	ATF2	-
chr2: 49173777-49173807	chr2:49173789 T>G	HK-pfg146	ATF2	-
chr2: 49173777-49173807	chr2:49173789 T>G	tan980437	ATF2	-
chr13: 36552830-36552850	chr13 36552831 A>T	HK-pfg054	RCOR1	-
chr13: 36552830-36552850	chr13:36552831 A>T	tan76629543	RCOR1	-
chr14: 70285576-70285601	chr14:70285588 T>G	HK-pfg092	-	SIN3A
chr14: 70285576-70285601	chr14:70285588 T>G	HK-pfg344	-	SIN3A
chr14: 70285576-70285601	chr14:70285588 T>G	TCGA-D7-6528	-	SIN3A

Supplementary Table 6. CBS hotspot mutations identified in previous genome-wide studies of gastrointestinal tumors and the COSMIC database.

Chr	Start	End	# mut in COSMIC	# mut in Katainen et al.	# mut in Umer et al.	Cancer types
chr6	50570094	50570120	12	15	0	BRCA, CRC, ESAD, GC, PACA
chr8	71000992	71001012	5	6	0	CRC, ESAD, HCC, LYMP
chr1	209422184	209422222	1	0	0	BRCA, CRC
chr2	49173770	49173816	24	4	6	CRC, ESAD, HCC, PACA, PRAD, OV
chr4	182064578	182064613	5	6	3	GC, HCC
chrX	104435106	104435140	6	5	0	BRCA, CRC, ESAD, GC, PACA
chr14	70285576	70285601	2	0	0	CRC, ESAD
chr6	73122084	73122123	16	9	7	BRCA, GC, ESAD, HCC, PACA
chr8	70576141	70576184	10	0	2	BRCA, ESAD, GC, HCC, LYMP, PACA
chr13	36552821	36552860	11	6	0	BRCA, ESAD, GC, HCC, KC, OV, PACA,
chr3	164903700	164903728	11	0	0	ESAD, GC, HCC, LYMP, PACA

Legend
# mut in COSMIC: Number of confirmed somatic mutations at CBS hotspots in COSMICv83
# mut in Katainen et al.: mutation clusters identified by Katainen et al. (Table S4)
# mut in Umer et al.: CBSs with at least 2 motif breaking mutations from Table S5 of Umer et al.

Kataien et al. studied colorectal cancer; Umer et al. studied liver, gastric, esophageal and pancreatic cancers

Cancer types: cancer types with mutations at CBS hotspots identified in previous studies
BRCA Breast cancer
CRC Colorectal cancer
ESAD Esophageal cancer
GC Gastric cancer
HCC Hepatocellular carcinoma
KC Kidney cancer
LYMP lymphoid neoplasm
OV Ovarian cancer
PACA Pancreatic cancer
PRAD Prostate cancer
