# Supplementary material for: Mutation hotspots at CTCF binding sites coupled to chromosomal instability in gastrointestinal cancers
Source: Nat Commun. 2018 Apr 18;9:1520. doi: 10.1038/s41467-018-03828-2 (PMC5906695; doi:10.1038/s41467-018-03828-2)
Supplement: Supplementary file 3 — Description of Additional Supplementary Files [file 41467_2018_3828_MOESM3_ESM.pdf]

Description of Additional Supplementary Files

File Name: Supplementary Data 1
Description: Clinical and WGS data of the 212 gastric cancer tumors.

File Name: Supplementary Data 2
Description: Epigenetic features for LASSO selection.

File Name: Supplementary Data 3
Description: Final set of SNV calls used for analysis.

File Name: Supplementary Data 4
Description: Final set of indel calls used for analysis.

File Name: Supplementary Data 5
Description: R markdown documents of all figures. Data files for the markdowns are available on request.
