# Supplementary material for: Mutation hotspots at CTCF binding sites coupled to chromosomal instability in gastrointestinal cancers
Source: Nat Commun. 2018 Apr 18;9:1520. doi: 10.1038/s41467-018-03828-2 (PMC5906695; doi:10.1038/s41467-018-03828-2)
Supplement: Supplementary file 8 — Supplementary Data 5 [file 41467_2018_3828_MOESM8_ESM.zip › Rmarkdowns/Figure 1/Figure1_summary_of_data_rev.html]

Figure 1 - Summary of Data


# Figure 1 - Summary of Data

This is the R Markdown for Figure 1, which consists of 3 parts.

```
cores=1 # cores=16
```

## Figure A

Mutation/Indel count/SNV count/Coverage across samples

```
# coverage
meta_data=read.delim("gastric_consolidated_meta.tsv", stringsAsFactors=FALSE)
meta_data[which(meta_data$Sample.ID=="apollo1"),"Sample.ID"]<-"apollo1_new"
maf.gastric=maf.to.granges("gastric_RF_prefiltered.MAF")
```

```
## [1] ">> Reading compact MAF ..."
```

```
maf.gastric=maf.gastric[-which(seqnames(maf.gastric)=="chrY")] # 4139879
maf.gastric=maf.gastric[-which(maf.gastric$tal=="FALSE")] # 4139877
# remove five samples tan2001206; tan20021007; tan980319; tan2000986; tan980436
maf.gastric=maf.gastric[-which(maf.gastric$sid %in% c("tan2001206", "tan20021007", "tan980319", "tan2000986", "tan980436"))] # 4116297
meta_data=meta_data[which(meta_data$Sample.ID %in% unique(maf.gastric$sid)),] # 187
meta_data=meta_data[,c("Sample.ID","WGS.mean.coverage","Batch")]
meta_data$batch=numeric(nrow(meta_data))
maf.gastric$ctype=as.character(maf.gastric$ctype)
for (i in 1:nrow(meta_data)){
  meta_data$batch[i]=unique(maf.gastric[which(maf.gastric$sid==meta_data$Sample.ID[i])]$ctype)
  # print(i)
}
map=list("TCGA"="TCGA", "HK"="HK", "ICGC"="ICGC", "tan"="SG")
meta_data$batch=sapply(meta_data$batch, function(x) {map[[x]]})
colnames(meta_data)[2]="coverage"
meta_data$coverage=as.numeric(meta_data$coverage) # 28.1 106.6

# snv count
snv=as.data.frame(maf.gastric)
snv=aggregate(width~sid,snv,sum) # 187 2
colnames(snv)[2]="count" # 1018 278054
snv=merge(snv,meta_data,by.x="sid",by.y="Sample.ID")
snv$batch=factor(snv$batch)
snv=snv[with(snv,order(batch,count)),] # order by batch and snv mutation count
snv$sid=as.character(snv$sid)
sid.levels=snv$sid
snv$sid=factor(snv$sid,levels=sid.levels)
snv$logcount=log10(snv$count) # 3.007748 5.444129
snv$type="snv"
q=ggplot(snv,aes(x=sid,y=logcount))+
  geom_bar(aes(fill=type),stat="identity",colour="black")+
  theme(panel.grid.major = element_blank(),
        panel.grid.minor = element_blank(),
        panel.background = element_blank(),
        axis.line = element_line(colour="black"))+
  theme(axis.text.x = element_text(angle = 90, hjust = 1))+
  scale_fill_manual(values=c("#FFFFFF"))+
  ylim(c(0,5.5))
print(q)
```

```
meta_data$Sample.ID=factor(meta_data$Sample.ID,levels=sid.levels)
meta_data$type="coverage"
p=ggplot(meta_data,aes(x=Sample.ID,y=coverage))+
  geom_bar(aes(fill=type),stat="identity",colour="black")+
  theme(panel.grid.major = element_blank(),
        panel.grid.minor = element_blank(),
        panel.background = element_blank(),
        axis.line = element_line(colour="black"))+
  theme(axis.text.x = element_text(angle = 90, hjust = 1))+
  scale_fill_manual(values=c("#FFFFFF"))+
  ylim(c(0,110))
print(p)
```

```
# indel count
indel=read.delim("gastric_RF_indels_prefiltered.MAF",header=FALSE,stringsAsFactors = FALSE) # 2081336
colnames(indel)=c("seqnames","start","end","ral","tal","sid","batch")
indel=indel[-which(indel$seqnames=="chrY"),] # 2079959
indel=indel[-which(indel$sid %in% c("tan2001206", "tan20021007", "tan980319", "tan2000986", "tan980436")),] # 2079690
indel=aggregate(seqnames~sid,indel,length) # 187 tan20021007 not in indels
colnames(indel)[2]="count" # 8 183299
indel$sid=factor(indel$sid,levels=sid.levels)
indel$logcount=log10(indel$count) # 0.90309 5.26316
indel$type="indel"
r=ggplot(indel,aes(x=sid,y=logcount))+
  geom_bar(aes(fill=type),stat="identity",colour="black")+
  theme(panel.grid.major = element_blank(),
        panel.grid.minor = element_blank(),
        panel.background = element_blank(),
        axis.line = element_line(colour="black"))+
  theme(axis.text.x = element_text(angle = 90, hjust = 1))+
  scale_fill_manual(values=c("#FFFFFF"))+
  ylim(c(0,5.5))
print(r)
```

```
# mutation spectrum
df=as.data.frame(maf.gastric)
df$mut=paste(df$ral,df$tal,sep=">")
table(df$mut)
```

```
## 
##    A>C    A>G    A>T    C>A    C>G    C>T    G>A    G>C    G>T    T>A 
## 357324 528310 193848 285962  90561 600793 601268  90667 286706 194294 
##    T>C    T>G 
## 527320 359244
```

```
df[which(df$mut=="A>T"),"mut"]<-"T>A"
df[which(df$mut=="A>C"),"mut"]<-"T>G"
df[which(df$mut=="A>G"),"mut"]<-"T>C"
df[which(df$mut=="G>A"),"mut"]<-"C>T"
df[which(df$mut=="G>C"),"mut"]<-"C>G"
df[which(df$mut=="G>T"),"mut"]<-"C>A"
table(df$mut)
```

```
## 
##     C>A     C>G     C>T     T>A     T>C     T>G 
##  572668  181228 1202061  388142 1055630  716568
```

```
df2=aggregate(width~sid+mut,df,sum) # 1122=187*6
colnames(df2)[3]="mut.count"
df2=merge(df2,snv,by="sid")
colnames(df2)[4]<-"total.count"
df2$prop=df2$mut.count/df2$total.count
df2$sid=factor(df2$sid,levels=sid.levels)
df2$mut=factor(df2$mut,levels=c("T>G","T>C","T>A","C>T","C>G","C>A"))
s=ggplot(df2,aes(x=sid,y=prop,fill=mut))+
  geom_bar(stat="identity",colour="black")+
  theme(panel.grid.major = element_blank(),
        panel.grid.minor = element_blank(),
        panel.background = element_blank(),
        axis.line = element_line(colour="black"))+
  theme(axis.text.x = element_text(angle = 90, hjust = 1))+
  scale_fill_manual(values = c("#33CC00","#33CCFF","#FF9933","#CC33FF","#FFFF33","#FF0000"))
print(s)
```

```
# subtype
consolidated=read.delim("gastric_consolidated_meta.tsv", stringsAsFactors=FALSE)
consolidated[which(consolidated$Sample.ID=="apollo1"),"Sample.ID"]<-"apollo1_new"
sum(consolidated$Sample.ID %in% snv$sid) # 187
```

```
## [1] 187
```

```
subtype=consolidated[which(consolidated$Sample.ID %in% snv$sid),c("Sample.ID","Molecular.Subtype")]
subtype[which(subtype$Sample.ID=="CGP_donor_GC00031"),"Molecular.Subtype"]<-"MSI"
subtype[which(subtype$Molecular.Subtype==""),"Molecular.Subtype"]<-"others"
subtype$val=1
subtype$Sample.ID=factor(subtype$Sample.ID,levels=sid.levels)
subtype$Molecular.Subtype=factor(subtype$Molecular.Subtype,levels=c("others","CIN","GS","EBV","MSI"))
table(subtype$Molecular.Subtype)
```

```
## 
## others    CIN     GS    EBV    MSI 
##     99     41     11     17     19
```

```
t=ggplot(subtype,aes(x=Sample.ID,y=val,fill=Molecular.Subtype))+
  geom_tile(colour="white")+
  theme(panel.grid.major = element_blank(),
        panel.grid.minor = element_blank(),
        panel.background = element_blank(),
        axis.line = element_line(colour="black"))+
  theme(axis.text.x = element_text(angle = 90, hjust = 1))+
  scale_fill_manual(values=c("#CCCCCC","#9966FF","#33FFFF","#669900","#FF6666"))
print(t)
```

```
# lauren
lauren=consolidated[which(consolidated$Sample.ID %in% snv$sid),c("Sample.ID","Laurens.Classification")]
lauren[which(lauren$Laurens.Classification==""),"Laurens.Classification"]<-"unknown"
lauren$val=1
lauren$Sample.ID=factor(lauren$Sample.ID,levels=sid.levels)
lauren[which(lauren$Laurens.Classification=="nos"),"Laurens.Classification"]<-"unknown"
lauren$Laurens.Classification=factor(lauren$Laurens.Classification,levels=c("unknown","mixed","diffuse","intestinal"))
table(lauren$Laurens.Classification)
```

```
## 
##    unknown      mixed    diffuse intestinal 
##         40         14         43         90
```

```
u=ggplot(lauren,aes(x=Sample.ID,y=val,fill=Laurens.Classification))+
  geom_tile(colour="white")+
  theme(panel.grid.major = element_blank(),
        panel.grid.minor = element_blank(),
        panel.background = element_blank(),
        axis.line = element_line(colour="black"))+
  theme(axis.text.x = element_text(angle = 90, hjust = 1))+
  scale_fill_manual(values=c("#CCCCCC","#FFFF00","#FF0000","#003300"))
print(u)
```

```
grid.arrange(arrangeGrob(p+theme(axis.title.x=element_blank(),
                                 axis.text.x=element_blank(),
                                 axis.ticks.x=element_blank(),
                                 axis.text.y=element_blank(),
                                 axis.title.y = element_blank(),
                                 legend.position = "none"),
                         q+theme(axis.title.x=element_blank(),
                                 axis.text.x=element_blank(),
                                 axis.ticks.x=element_blank(),
                                 axis.text.y=element_blank(),
                                 axis.title.y = element_blank(),
                                 legend.position="none"),
                         r+theme(axis.title.x=element_blank(),
                                 axis.text.x=element_blank(),
                                 axis.ticks.x=element_blank(),
                                 axis.text.y=element_blank(),
                                 axis.title.y = element_blank(),
                                 legend.position = "none"),
                         s+theme(axis.title.x=element_blank(),
                                 axis.text.x=element_blank(),
                                 axis.ticks.x=element_blank(),
                                 legend.position="none",
                                 axis.text.y=element_blank(),
                                 axis.title.y = element_blank()),
                         t+theme(axis.title.x=element_blank(),
                                 axis.text.x=element_blank(),
                                 axis.ticks.x=element_blank(),
                                 legend.position="none",
                                 axis.text.y=element_blank(),
                                 axis.title.y = element_blank()),
                         u+theme(legend.position="none",
                                 axis.text.y=element_blank(),
                                 axis.title.y = element_blank(),
                                 axis.text.x=element_blank()), 
                         ncol=1,nrow=6,heights=c(4,4,4,4,2,2)))
```

## Figure B

Correlation between roadmap epigenetic features and mutation rate

```
binnedMean2=function(bins, numvar) {
  stopifnot(is(bins, "GRangesList"))
  stopifnot(is(numvar, "RleList"))
  mean.list <- mclapply(names(bins),
                        function(binname) {
                          print(binname)
                          seqname=unique(as.character(seqnames(bins[[binname]])))
                          views <- Views(numvar[[seqname]],ranges(bins[[binname]]))
                          sum(viewSums(views))/sum(as.numeric(width(bins[[binname]])))
                        },mc.cores=9)
  names(mean.list)=names(bins)
  mean.list=unlist(mean.list)
  mean.list
}

bigwig.summarize.bins2=function(bigWigUrl,bins, type='mean') {
  print(paste0('>>',bigWigUrl))
  ss.cov = import(as.character(bigWigUrl),as='RleList')
  ss.cov = ss.cov[intersect(names(ss.cov),seqlevels(bins))]
  # add empty missing chromosomes
  for (c in setdiff(seqlevels(bins),names(ss.cov))) {ss.cov[[c]] = Rle()}
  if (type=='mean') {
    # compute binned Mean
    ss.bins = binnedMean2(bins,ss.cov)
  } else if (type=='max') {
    # compute binned Max
    ss.bins = binnedMax(bins,ss.cov,'score')     
  }
  ss.bins[is.infinite(ss.bins)] <- 0
  ss.bins[is.nan(ss.bins)] <- 0
  ss.bins
}

chrOrder<-c(paste("chr",1:22,sep=""),"chrX")
seqi = seqinfo(Hsapiens)[seqnames(Hsapiens)[1:23]]
seqnames=seqnames(seqinfo(Hsapiens))[1:23]

## Tile genome into equally sized bins
binsize = 1000*1e3
cores=4
genome.bins <- tileGenome(seqi, tilewidth=binsize, cut.last.tile.in.chrom=TRUE)
nbin = length(genome.bins) # 3053 1mb bins; 30376 100kb bins
names(genome.bins)=paste("n", seq(1:nbin), sep="")
genome.bins.grl<- split(genome.bins, names(genome.bins))

## mask regions
mappability=import("wgEncodeCrgMapabilityAlign75mer.bigWig") 
## define reads that can map to more than 1 genomic location as non-mappable
nonmappable=mappability[mappability$score<1,]
# convert zero-based coordinates to one-based coordinates
nonmappable=shift(nonmappable,1)
nonmappable= reduce(nonmappable)
nonmappable=nonmappable[seqnames(nonmappable) %in% seqnames(seqi)]
seqlevels(nonmappable)=as.character(unique(seqnames(nonmappable)))

# trim ends of each chromosome that are N's
mappability.grl=split(mappability, seqnames(mappability))
ranges=lapply(mappability.grl, range)
ranges=GRangesList(ranges)
ranges=unlist(ranges)
extrSeq=Views(Hsapiens,ranges) # check that all N's at chromosome ends are trimmed
# define chromosome ends to be masked
ranges=ranges[seqnames(ranges) %in% seqnames(seqi)] # 2441960
seqlevels(ranges)=as.character(unique(seqnames(ranges)))
end1=GRanges(seqnames(ranges), IRanges(1, start(ranges)-1))
end2=GRanges(seqnames(ranges), IRanges(end(ranges)+1, seqlengths(seqi[as.character(seqnames(ranges))])))

## mask CDS and ig loci
roi.cds <- bed.to.granges('Ensembl75.CDS.bed')
roi.cds.ext <- reduce(roi.cds + 5) # extend each region with +/- 5 bases and get all non-overlapping regions
# immunoglobulin loci
ig.loci <- bed.to.granges('ig_loci.bed') # /mnt/projects/guoy1/wgs/Gastric_Cancer/data/ig_loci.bed
ig.loci <- reduce(ig.loci + 10**5) # extend each region with 100kb and get all non-overlapping regions
# combine mask regions
mask.regions=reduce(trim(c(ig.loci,roi.cds.ext,nonmappable,end1,end2))) 

genome.bins.grl.masked= subtract.regions.from.roi(genome.bins.grl, mask.regions, cores=cores)
genome.bins.grl.masked=genome.bins.grl.masked[sum(width(genome.bins.grl.masked))>=250000] # 2924
mean(sum(width(genome.bins.grl.masked))) # 904014.2
median(sum(width(genome.bins.grl.masked))) # 927924.5

## calculate mean value of roadmap epigenome feature for each bin
roadmap.urls <- read.table('roadmap_all_urls.txt', header=F, sep="\t")
roadmap.gastric.urls=roadmap.urls[roadmap.urls[,2]%in% c("E094", "E092", "E110", "E111"),] # 27 features
roadmap.gastric.urls[,3]=as.character(roadmap.gastric.urls[,3])
roadmap.gastric.features2=mclapply(roadmap.gastric.urls[,3],function(f) {bigwig.summarize.bins2(f,genome.bins.grl.masked,'mean')},mc.cores=cores,mc.preschedule = FALSE, mc.silent = FALSE)
roadmap.gastric.features= do.call(cbind,roadmap.gastric.features2)
colnames(roadmap.gastric.features) <- paste(roadmap.gastric.urls[,1], roadmap.gastric.urls[,2], sep="_") # 2924 27
roadmap.gastric.features=data.frame(roadmap.gastric.features)

## calculate mean value of replication timing and local_mutrate for each bin
mean_rep_time2=bigwig.summarize.bins2("wgEncodeUwRepliSeqWaveSignalMean.bigWig", genome.bins.grl.masked, 'mean')
roadmap.gastric.features=cbind(roadmap.gastric.features,mean_rep_time2) # 2924 28
colnames(roadmap.gastric.features)[ncol(roadmap.gastric.features)]="mean_rep_time"
```

```
subtype_classification <- read.delim("subtype_classification.txt", stringsAsFactors=FALSE)
CIN=subtype_classification[which(subtype_classification$Molecular.Subtype=="CIN"),"Sample.ID"] # 42
EBV=subtype_classification[which(subtype_classification$Molecular.Subtype=="EBV"),"Sample.ID"] # 17
GS=subtype_classification[which(subtype_classification$Molecular.Subtype=="GS"),"Sample.ID"] # 19
GS=c(GS,"apollo1_new") # 20

maf.gastric.msi <- maf.to.granges('gastric_RF_MSI_prefiltered.MAF') # 1698980
maf.gastric.msi=maf.gastric.msi[seqnames(maf.gastric.msi) %in% seqnames(seqi)] # 1697924
seqlengths(maf.gastric.msi)[names(seqlengths(seqi))]<-seqlengths(seqi)

maf.gastric.nonmsi <- maf.to.granges('gastric_RF_nonMSI_prefiltered.MAF') # 2444729
maf.gastric.nonmsi=maf.gastric.nonmsi[-which(maf.gastric.nonmsi$sid %in% c("tan2001206", "tan20021007", "tan980319", "tan2000986", "tan980436"))] # 2420832, ~-1%
maf.gastric.nonmsi=maf.gastric.nonmsi[seqnames(maf.gastric.nonmsi) %in% seqnames(seqi)] # 2418375
seqlengths(maf.gastric.nonmsi)[names(seqlengths(seqi))]<-seqlengths(seqi)

# will only use chr 1:22 and X
maf.gastric.msi.cov<-coverage(maf.gastric.msi)[seqnames(seqi)]
maf.gastric.msi$sid=as.character(maf.gastric.msi$sid)
levels(maf.gastric.msi$sid)=unique(maf.gastric.msi$sid)
npatients.msi = length(levels(maf.gastric.msi$sid)) # 19 patients

maf.gastric.cin=maf.gastric.nonmsi[which(maf.gastric.nonmsi$sid %in% CIN)] # 729958
maf.gastric.cin.cov <- coverage(maf.gastric.cin)[seqnames(seqi)]
maf.gastric.cin$sid=as.character(maf.gastric.cin$sid)
levels(maf.gastric.cin$sid)=unique(maf.gastric.cin$sid)
npatients.cin = length(levels(maf.gastric.cin$sid)) # 41 patients

maf.gastric.ebv=maf.gastric.nonmsi[which(maf.gastric.nonmsi$sid %in% EBV)] # 251708
maf.gastric.ebv.cov <- coverage(maf.gastric.ebv)[seqnames(seqi)]
maf.gastric.ebv$sid=as.character(maf.gastric.ebv$sid)
levels(maf.gastric.ebv$sid)=unique(maf.gastric.ebv$sid)
npatients.ebv = length(levels(maf.gastric.ebv$sid)) # 17 patients

maf.gastric.gs=maf.gastric.nonmsi[which(maf.gastric.nonmsi$sid %in% GS)] # 121767
maf.gastric.gs.cov <- coverage(maf.gastric.gs)[seqnames(seqi)]
maf.gastric.gs$sid=as.character(maf.gastric.gs$sid)
levels(maf.gastric.gs$sid)=unique(maf.gastric.gs$sid)
npatients.gs = length(levels(maf.gastric.gs$sid)) # 11 patients

binnedSum2=function(bins, numvar, mcolname) {
  stopifnot(is(bins, "GRangesList"))
  stopifnot(is(numvar, "RleList"))
  mean.list <- mclapply(names(bins),
                        function(binname) {
                          print(binname)
                          seqname=unique(as.character(seqnames(bins[[binname]])))
                          views <- Views(numvar[[seqname]],ranges(bins[[binname]]))
                          sum(viewSums(views))
                        },mc.cores=9)
  names(mean.list)=names(bins)
  unlist(mean.list)
}

## calculate mutation rate for each bin
binwidth=lapply(genome.bins.grl.masked,FUN=function(x) {
  sum(as.numeric(width(x)))
})
binwidth=unlist(binwidth)

genome.bins.mutrate.msi <- binnedSum2(genome.bins.grl.masked,maf.gastric.msi.cov,'score')
mut.rate.msi=genome.bins.mutrate.msi/(binwidth*npatients.msi)

genome.bins.mutrate.cin <- binnedSum2(genome.bins.grl.masked,maf.gastric.cin.cov,'score')
mut.rate.cin=genome.bins.mutrate.cin/(binwidth*npatients.cin)

genome.bins.mutrate.ebv <- binnedSum2(genome.bins.grl.masked,maf.gastric.ebv.cov,'score')
mut.rate.ebv=genome.bins.mutrate.ebv/(binwidth*npatients.ebv)

genome.bins.mutrate.gs <- binnedSum2(genome.bins.grl.masked,maf.gastric.gs.cov,'score')
mut.rate.gs=genome.bins.mutrate.gs/(binwidth*npatients.gs)

# combine roadmap.gastric.features and genome.bins.mutrate.msi, genome.bins.mutrate.cin, genome.bins.mutrate.ebv and genome.bins.mutrate.gs
roadmap.cor.msi=apply(roadmap.gastric.features,2,function(x) cor.test(x,mut.rate.msi)$estimate)
roadmap.cor.msi=data.frame(roadmap.cor.msi,epi=colnames(roadmap.gastric.features),type="msi")
colnames(roadmap.cor.msi)[1]="corr"
roadmap.cor.cin=apply(roadmap.gastric.features,2,function(x) cor.test(x,mut.rate.cin)$estimate)
roadmap.cor.cin=data.frame(roadmap.cor.cin,epi=colnames(roadmap.gastric.features),type="cin")
colnames(roadmap.cor.cin)[1]="corr"
roadmap.cor.ebv=apply(roadmap.gastric.features,2,function(x) cor.test(x,mut.rate.ebv)$estimate)
roadmap.cor.ebv=data.frame(roadmap.cor.ebv,epi=colnames(roadmap.gastric.features),type="ebv")
colnames(roadmap.cor.ebv)[1]="corr"
roadmap.cor.gs=apply(roadmap.gastric.features,2,function(x) cor.test(x,mut.rate.gs)$estimate)
roadmap.cor.gs=data.frame(roadmap.cor.gs,epi=colnames(roadmap.gastric.features),type="gs")
colnames(roadmap.cor.gs)[1]="corr"

df=rbind(roadmap.cor.msi,roadmap.cor.cin,roadmap.cor.ebv,roadmap.cor.gs) #112 3
df=df[which(rownames(df) %in% c("H3K27ac_E094","H3K36me3_E094","H3K4me3_E094","DNase_E094","H3K9ac_E110","H3K27me3_E094","H3K9me3_E094","H3K4me1_E094","mean_rep_time","H3K27ac_E0941","H3K36me3_E0941","H3K4me3_E0941","DNase_E0941","H3K9ac_E1101","H3K27me3_E0941","H3K9me3_E0941","H3K4me1_E0941","mean_rep_time1","H3K27ac_E0942","H3K36me3_E0942","H3K4me3_E0942","DNase_E0942","H3K9ac_E1102","H3K27me3_E0942","H3K9me3_E0942","H3K4me1_E0942","mean_rep_time2","H3K27ac_E0943","H3K36me3_E0943","H3K4me3_E0943","DNase_E0943","H3K9ac_E1103","H3K27me3_E0943","H3K9me3_E0943","H3K4me1_E0943","mean_rep_time3")),] #36 3
df$type=as.character(df$type)
df$type=factor(df$type,levels=c("cin","gs","ebv","msi"))
df$epi=as.character(df$epi)
df$epi=factor(df$epi,levels=c("mean_rep_time","DNase_E094","H3K36me3_E094","H3K4me3_E094",
                              "H3K9ac_E110","H3K27ac_E094","H3K4me1_E094","H3K9me3_E094","H3K27me3_E094"))

# write summarized output to a table
write.table(df,"fig1.correlation.table.masked.txt",sep="\t",quote=FALSE,col.names=TRUE,row.names=TRUE)

## error bar by sample
# will only use chr 1:22 and X
mut.rate.msi=mclapply(unique(as.character(maf.gastric.msi$sid)), FUN=function(x){
   maf.gastric.msi.cov<-coverage(maf.gastric.msi[which(maf.gastric.msi$sid==x)])[seqnames(seqi)]
   npatients.msi=1
   genome.bins.mutrate.msi <- binnedSum2(genome.bins.grl.masked,maf.gastric.msi.cov,'score')
   mut.rate.msi=genome.bins.mutrate.msi/(binwidth*npatients.msi)
   mut.rate.msi
}, mc.cores=5,mc.preschedule = FALSE)
names(mut.rate.msi)=unique(as.character(maf.gastric.msi$sid))

mut.rate.cin=mclapply(unique(as.character(maf.gastric.cin$sid)), FUN=function(x){
   maf.gastric.cin.cov <- coverage(maf.gastric.cin[which(maf.gastric.cin$sid==x)])[seqnames(seqi)]
   npatients.cin=1
   genome.bins.mutrate.cin <- binnedSum2(genome.bins.grl.masked,maf.gastric.cin.cov,'score')
   mut.rate.cin=genome.bins.mutrate.cin/(binwidth*npatients.cin)
},mc.cores=5,mc.preschedule = FALSE)
names(mut.rate.cin)=unique(as.character(maf.gastric.cin$sid))

mut.rate.ebv=mclapply(unique(as.character(maf.gastric.ebv$sid)), FUN=function(x){
   maf.gastric.ebv.cov <- coverage(maf.gastric.ebv[which(maf.gastric.ebv$sid==x)])[seqnames(seqi)]
   npatients.ebv=1
   genome.bins.mutrate.ebv <- binnedSum2(genome.bins.grl.masked,maf.gastric.ebv.cov,'score')
   mut.rate.ebv=genome.bins.mutrate.ebv/(binwidth*npatients.ebv)
},mc.cores=5,mc.preschedule = FALSE)
names(mut.rate.ebv)=unique(as.character(maf.gastric.ebv$sid))

mut.rate.gs=mclapply(unique(as.character(maf.gastric.gs$sid)), FUN=function(x){
  maf.gastric.gs.cov <- coverage(maf.gastric.gs[which(maf.gastric.gs$sid==x)])[seqnames(seqi)]
  npatients.gs=1
  genome.bins.mutrate.gs <- binnedSum2(genome.bins.grl.masked,maf.gastric.gs.cov,'score')
  mut.rate.gs=genome.bins.mutrate.gs/(binwidth*npatients.gs)
}, mc.cores=5, mc.preschedule = FALSE)
names(mut.rate.gs)=unique(as.character(maf.gastric.gs$sid))

# combine roadmap.gastric.features and genome.bins.mutrate.msi, genome.bins.mutrate.cin, genome.bins.mutrate.ebv and genome.bins.mutrate.gs
roadmap.cor.gs=lapply(mut.rate.gs,FUN=function(x) {
  apply(roadmap.gastric.features,2,function(j) cor.test(j,x)$estimate)
}) #11
roadmap.cor.gs=do.call(rbind,roadmap.cor.gs)
roadmap.cor.msi=lapply(mut.rate.msi,FUN=function(x) {
  apply(roadmap.gastric.features,2,function(j) cor.test(j,x)$estimate)
}) #19
roadmap.cor.msi=do.call(rbind,roadmap.cor.msi)
roadmap.cor.cin=lapply(mut.rate.cin,FUN=function(x) {
  apply(roadmap.gastric.features,2,function(j) cor.test(j,x)$estimate)
}) #41
roadmap.cor.cin=do.call(rbind,roadmap.cor.cin)
roadmap.cor.ebv=lapply(mut.rate.ebv,FUN=function(x) {
  apply(roadmap.gastric.features,2,function(j) cor.test(j,x)$estimate)
}) #17
roadmap.cor.ebv=do.call(rbind,roadmap.cor.ebv)

se <- function(x) sqrt(var(x)/length(x))
gs.mean=apply(roadmap.cor.gs,2,FUN=function(x) mean(x))
gs.se=apply(roadmap.cor.gs,2,FUN=function(x) se(x))
gs.sd=apply(roadmap.cor.gs,2,FUN=function(x) sd(x))

msi.mean=apply(roadmap.cor.msi,2,FUN=function(x) mean(x))
msi.se=apply(roadmap.cor.msi,2,FUN=function(x) se(x))
msi.sd=apply(roadmap.cor.msi,2,FUN=function(x) sd(x))

ebv.mean=apply(roadmap.cor.ebv,2,FUN=function(x) mean(x))
ebv.se=apply(roadmap.cor.ebv,2,FUN=function(x) se(x))
ebv.sd=apply(roadmap.cor.ebv,2,FUN=function(x) sd(x))

cin.mean=apply(roadmap.cor.cin,2,FUN=function(x) mean(x))
cin.se=apply(roadmap.cor.cin,2,FUN=function(x) se(x))
cin.sd=apply(roadmap.cor.cin,2,FUN=function(x) sd(x))

gs.df=data.frame(epi=names(gs.mean),mean=gs.mean,se=gs.se,sd=gs.sd)
gs.df$type="gs"
msi.df=data.frame(epi=names(msi.mean),mean=msi.mean,se=msi.se,sd=msi.sd)
msi.df$type="msi"
ebv.df=data.frame(epi=names(ebv.mean),mean=ebv.mean,se=ebv.se,sd=ebv.sd)
ebv.df$type="ebv"
cin.df=data.frame(epi=names(cin.mean),mean=cin.mean,se=cin.se,sd=cin.sd)
cin.df$type="cin"

# df=rbind(roadmap.cor.msi,roadmap.cor.cin,roadmap.cor.ebv,roadmap.cor.gs) #112 3
df=rbind(gs.df,msi.df,ebv.df,cin.df) #112 5

df=df[which(rownames(df) %in% c("H3K27ac_E094","H3K36me3_E094","H3K4me3_E094","DNase_E094","H3K9ac_E110","H3K27me3_E094",
                                "H3K9me3_E094","H3K4me1_E094","mean_rep_time",
                                "H3K27ac_E0941","H3K36me3_E0941","H3K4me3_E0941","DNase_E0941","H3K9ac_E1101",
                                "H3K27me3_E0941","H3K9me3_E0941","H3K4me1_E0941","mean_rep_time1",
                                "H3K27ac_E0942","H3K36me3_E0942","H3K4me3_E0942","DNase_E0942","H3K9ac_E1102",
                                "H3K27me3_E0942","H3K9me3_E0942","H3K4me1_E0942","mean_rep_time2",
                                "H3K27ac_E0943","H3K36me3_E0943","H3K4me3_E0943","DNase_E0943","H3K9ac_E1103",
                                "H3K27me3_E0943","H3K9me3_E0943","H3K4me1_E0943","mean_rep_time3")),] #36 5
df$type=as.character(df$type)
df$type=factor(df$type,levels=c("cin","gs","ebv","msi"))
df$epi=as.character(df$epi)
df$epi=factor(df$epi,levels=c("mean_rep_time","DNase_E094","H3K36me3_E094","H3K4me3_E094",
                              "H3K9ac_E110","H3K27ac_E094","H3K4me1_E094","H3K9me3_E094","H3K27me3_E094"))

# write summarized output to a table
write.table(df,"fig1.correlation.table.masked.sample.txt",sep="\t",quote=FALSE,col.names=TRUE,row.names=TRUE)
```

Read in summarized table

```
df=read.table("fig1.correlation.table.masked.txt")
df.sample=read.table("fig1.correlation.table.masked.sample.txt")
```

```
df$type=as.character(df$type)
df$type=factor(df$type,levels=c("cin","gs","ebv","msi"))
df$epi=as.character(df$epi)
df$epi=factor(df$epi,levels=c("mean_rep_time","DNase_E094","H3K36me3_E094","H3K4me3_E094",
                              "H3K9ac_E110","H3K27ac_E094","H3K4me1_E094","H3K9me3_E094","H3K27me3_E094"))

print(ggplot(df,aes(x=epi,y=corr,fill=type))+
  geom_bar(position=position_dodge(0.9),stat="identity",colour="black")+
  scale_fill_manual(values=c("#9966FF","#33FFFF","#669900","#FF6666"))+
  theme(axis.text.x = element_text(angle = 90, hjust = 1))+
  theme(panel.grid.major = element_blank(),
        panel.grid.minor = element_blank(),
        panel.background = element_blank(),
        axis.line = element_line(colour="black"))+
  theme(axis.text.y = element_text(size=15))+
  theme(legend.text = element_text(size=15))+
  xlab("Features")+ylab("Correlation")+
  ggtitle(paste("Correlation between features (roadmap epigenetic) and", "mutation rate for each subtype (binned)",sep="\n")))
```

```
df.sample$type=as.character(df.sample$type)
df.sample$type=factor(df.sample$type,levels=c("cin","gs","ebv","msi"))
df.sample$epi=as.character(df.sample$epi)
df.sample$epi=factor(df.sample$epi,levels=c("mean_rep_time","DNase_E094","H3K36me3_E094","H3K4me3_E094",
                              "H3K9ac_E110","H3K27ac_E094","H3K4me1_E094","H3K9me3_E094","H3K27me3_E094"))

print(ggplot(df.sample,aes(x=epi,y=mean,fill=type))+
  geom_bar(position=position_dodge(),stat="identity",colour="black")+
  geom_errorbar(aes(ymin=mean-se,ymax=mean+se),width=0.25,position=position_dodge(0.9))+
  theme(axis.text.x = element_text(angle = 90, hjust = 1))+
    scale_fill_manual(values=c("#9966FF","#33FFFF","#669900","#FF6666"))+
#  ylim(c(-0.5,0.7))+
  theme(panel.grid.major = element_blank(),
        panel.grid.minor = element_blank(),
        panel.background = element_blank(),
        axis.line = element_line(colour="black"))+
  theme(axis.text.y = element_text(size=15))+
  theme(legend.text = element_text(size=15))+
  xlab("Features")+ylab("Correlation")+
  ggtitle(paste("Correlation between features (roadmap epigenetic) and", "mutation rate for each subtype (binned)","per sample, 1std.error",sep="\n")))
```

## Figure C

PCA roadmap epigenetic contributions

```
multiplot <- function(..., plotlist=NULL, file, cols=1, layout=NULL) {
  library(grid)
  
  # Make a list from the ... arguments and plotlist
  plots <- c(list(...), plotlist)
  
  numPlots = length(plots)
  
  # If layout is NULL, then use 'cols' to determine layout
  if (is.null(layout)) {
    # Make the panel
    # ncol: Number of columns of plots
    # nrow: Number of rows needed, calculated from # of cols
    layout <- matrix(seq(1, cols * ceiling(numPlots/cols)),
                     ncol = cols, nrow = ceiling(numPlots/cols))
  }
  
  if (numPlots==1) {
    print(plots[[1]])
    
  } else {
    # Set up the page
    grid.newpage()
    pushViewport(viewport(layout = grid.layout(nrow(layout), ncol(layout))))
    
    # Make each plot, in the correct location
    for (i in 1:numPlots) {
      # Get the i,j matrix positions of the regions that contain this subplot
      matchidx <- as.data.frame(which(layout == i, arr.ind = TRUE))
      
      print(plots[[i]], vp = viewport(layout.pos.row = matchidx$row,
                                      layout.pos.col = matchidx$col))
    }
  }
}

maf.gastric <- maf.to.granges('gastric_RF.MAF')
```

```
## [1] ">> Reading compact MAF ..."
```

```
maf.gastric.grl <- split(maf.gastric, maf.gastric$sid)
```

```
epi.urls <- read.table('pca_covariates_urls.txt') # previously used "hotspot_selected_urls_gastric.txt" => epi.urls=epi.urls[-(4:5),] /mnt/projects/guoy1/wgs/Gastric_Cancer/data/pca_covariates_urls.txt
epi.features = mclapply(epi.urls[,2],function(f) {bigwig.summarize.bins2(f,genome.bins.grl.masked)}, mc.cores=cores, mc.preschedule = FALSE, mc.silent = FALSE)
epi.features = do.call(cbind,epi.features)
colnames(epi.features) = as.character(epi.urls[,1])

mut.cor=mclapply(maf.gastric.grl, function(f){
  maf.cov <- coverage(f)[seqnames(seqi)]
  mut.count<-binnedSum2(genome.bins.grl.masked, maf.cov,'score')
  mut.rate <-mut.count/(binwidth)  
  cor.int=apply(epi.features,2,function(x) cor.test(x,mut.rate)$estimate)  
}, mc.cores=cores, mc.preschedule = FALSE, mc.silent = FALSE)

mut.cor=do.call(rbind, mut.cor) # 192 10
# write summarized output to a table
write.table(mut.cor,"mut_epi_correlation_1000kb_masked.txt",sep="\t",quote=FALSE,col.names=TRUE,row.names=TRUE)
```

```
# read in summarized table
mut.cor=read.table("mut_epi_correlation_1000kb_masked.txt") 
apply(mut.cor,2, median)
```

```
## local_mutrate mean_rep_time    DNase_E094  H3K4me1_E094  H3K4me3_E094 
##     0.6899701    -0.3805161    -0.3037659    -0.3207476    -0.2769219 
##   H3K9ac_E110  H3K9me3_E094  H3K27ac_E094 H3K27me3_E094 H3K36me3_E094 
##    -0.3119420     0.2330829    -0.2910978     0.1422648    -0.2777212
```

```
row.names(mut.cor)[10]="apollo1" # apollo1_new
mut.cor=mut.cor[,-1]
# remove 5 samples with oxidative damage
mut.cor=mut.cor[!rownames(mut.cor)%in%c("tan2001206", "tan20021007", "tan980319", "tan2000986", "tan980436"),]

meta = read.delim("GC_meta_info.txt",sep="\t",header=TRUE, stringsAsFactors = FALSE) #
meta=meta[meta$Sample.ID%in%row.names(mut.cor),]
subtype= meta$Molecular.Subtype
subtype[subtype==""]="unknown"
names(subtype)=meta$Sample.ID
subtype=subtype[row.names(mut.cor)]

# principle component analysis
pca<-prcomp(mut.cor,retx=TRUE,center=TRUE,scale=TRUE)
scores<-pca$x
# PCA color by MSI status
colorCodes2 <- c("MSI"="#FF6666", "Non-MSI"="#999999")
MSIseq=read.delim("MSIseq_classification.txt",sep="\t",header=TRUE, stringsAsFactors = FALSE)
MSIseq$MSI_status=ifelse(MSIseq$MSI_status=="MSI-H","MSI","Non-MSI")
MSI=MSIseq$MSI_status
names(MSI)=MSIseq$Tumor_Sample_Barcode
names(MSI)[34]="apollo1"
MSI=MSI[names(subtype)] # sort sample names

par(mfrow=c(2,2))
plot(pca)
legend('topright',inset=0.05, legend=names(colorCodes2),pch=16, col=colorCodes2, bty="n", cex=0.8)
plot(scores[,1],scores[,2],col=colorCodes2[MSI], pch=16, xlab="PC1", yla="PC2")
plot(scores[,1],scores[,3],col=colorCodes2[MSI], pch=16, xlab="PC1", yla="PC3")
plot(scores[,2],scores[,3],col=colorCodes2[MSI], pch=16, xlab="PC2", yla="PC3")
```

```
colorCodes3<-c("MSI"="#FFFF66","EBV"="#33FF99","CIN"="#3399FF","GS"="#FF6666")
par(mfrow=c(2,2))
plot(pca)
legend('topright',inset=0.05, legend=names(colorCodes3),pch=16, col=colorCodes3, bty="n", cex=0.8)
plot(scores[,1],scores[,2],col=colorCodes3[subtype], pch=16, xlab="PC1", yla="PC2")
plot(scores[,1],scores[,3],col=colorCodes3[subtype], pch=16, xlab="PC1", yla="PC3")
plot(scores[,2],scores[,3],col=colorCodes3[subtype], pch=16, xlab="PC2", yla="PC3")
```

```
aload <- abs(pca$rotation)
load.frac=sweep(aload, 2, colSums(aload), "/")
load.frac
```

```
##                        PC1         PC2          PC3          PC4
## mean_rep_time 0.1294858128 0.038072059 0.1808593074 0.0001811129
## DNase_E094    0.1302907203 0.065607388 0.0819337521 0.4657520121
## H3K4me1_E094  0.1336083044 0.009898685 0.0129863001 0.0508778729
## H3K4me3_E094  0.1326796020 0.010512993 0.0974178377 0.0845797685
## H3K9ac_E110   0.1326924914 0.051790504 0.0166540362 0.0926119450
## H3K9me3_E094  0.0007199789 0.437043117 0.2084405733 0.0173548737
## H3K27ac_E094  0.1333262684 0.030181941 0.0003969922 0.0902157594
## H3K27me3_E094 0.0759119992 0.342603752 0.2584292209 0.0372349155
## H3K36me3_E094 0.1312848225 0.014289560 0.1428819803 0.1611917400
##                        PC5         PC6         PC7          PC8
## mean_rep_time 0.3710266318 0.023662565 0.031503429 0.1050497316
## DNase_E094    0.0458084280 0.004020917 0.001371999 0.0351942952
## H3K4me1_E094  0.0007330274 0.195321660 0.239006264 0.2601172407
## H3K4me3_E094  0.0068567106 0.027690028 0.300980882 0.1917866541
## H3K9ac_E110   0.0477161824 0.254317998 0.064315288 0.0375497945
## H3K9me3_E094  0.1655009322 0.061370897 0.051118824 0.0001761279
## H3K27ac_E094  0.0680575140 0.207558362 0.217087447 0.0591128463
## H3K27me3_E094 0.1957913312 0.028407877 0.076469411 0.0310280895
## H3K36me3_E094 0.0985092424 0.197649696 0.018146456 0.2799852202
##                       PC9
## mean_rep_time 0.002251839
## DNase_E094    0.005175672
## H3K4me1_E094  0.097057439
## H3K4me3_E094  0.197101353
## H3K9ac_E110   0.327114443
## H3K9me3_E094  0.019584236
## H3K27ac_E094  0.285303248
## H3K27me3_E094 0.006270763
## H3K36me3_E094 0.060141006
```

```
###
pca <- as.data.frame(load.frac)
df=data.frame(class=rep(c("PC1","PC2"),each=9),roadmap=rep(rownames(pca),2),values=c(pca$PC1,pca$PC2))
sum(df[which(df$class=="PC1"),"values"])
```

```
## [1] 1
```

```
sum(df[which(df$class=="PC2"),"values"])
```

```
## [1] 1
```

```
df$roadmap=as.character(df$roadmap)
df$roadmap=factor(df$roadmap,levels=c("mean_rep_time","DNase_E094","H3K36me3_E094","H3K4me3_E094",
                                      "H3K9ac_E110","H3K27ac_E094","H3K4me1_E094","H3K9me3_E094","H3K27me3_E094"))

ggplot(df,aes(x=class,y=values))+geom_bar(aes(fill=roadmap),stat="identity",position=position_stack(reverse=TRUE))+
  coord_flip()+  theme(panel.grid.major = element_blank(),
                       panel.grid.minor = element_blank(),
                       panel.background = element_blank(),
                       axis.line = element_line(colour="black"))+
  theme(axis.text.y = element_text(size=15))+scale_fill_brewer(palette="Set3")
```
