# Supplementary material for: Mutation hotspots at CTCF binding sites coupled to chromosomal instability in gastrointestinal cancers
Source: Nat Commun. 2018 Apr 18;9:1520. doi: 10.1038/s41467-018-03828-2 (PMC5906695; doi:10.1038/s41467-018-03828-2)
Supplement: Supplementary file 8 — Supplementary Data 5 [file 41467_2018_3828_MOESM8_ESM.zip › Rmarkdowns/Figure 2/Figure2_indel_candidates_rev.html]

Figure 2 - Indel candidates


# Figure 2 - Indel candidates

This is the R Markdown for Figure 2, which consists of 6 parts.

```
cores=1 # cores=12
```

## Figure B

Manhattan plot for indel hotspots

```
hotspot_candidates <- read.delim("LRmodel_hotspot_indel_nonMSI_prefiltered_poly_v2_remove5_corrected.tsv", stringsAsFactors=FALSE) #62893
hotspot_candidates$CHR=ifelse(hotspot_candidates$chrom=="chrX","23",substr(hotspot_candidates$chrom,4,nchar(hotspot_candidates$chrom)))
hotspot_candidates$CHR=as.numeric(hotspot_candidates$CHR)
hotspot_candidates$bp=ceiling((hotspot_candidates$start+hotspot_candidates$end)/2)
hotspot_candidates$mut_region=rownames(hotspot_candidates)
hotspot_candidates$id=hotspot_candidates$mut_region
hotspot_candidates=GRanges(seqnames=hotspot_candidates$chrom,
                           IRanges(start=hotspot_candidates$start,end=hotspot_candidates$end),
                           pval=hotspot_candidates$pval,
                           fdr=hotspot_candidates$fdr,
                           mut_region=hotspot_candidates$mut_region,
                           length=hotspot_candidates$length,
                           p.bg=hotspot_candidates$p.bg,
                           k=hotspot_candidates$k)
# plot only the unique hotspots
hotspot=reduce(hotspot_candidates) #62739
hotspot$hotspot=c(1:length(hotspot))
z=findOverlaps(hotspot_candidates,hotspot)
t=as.data.frame(hotspot_candidates[queryHits(z)])
t$hotspot=hotspot[subjectHits(z)]$hotspot
t=t[order(t$pval,decreasing=FALSE),] #62893
q=t[!duplicated(t$hotspot),] #62739
hotspot_candidates=q

hotspot_candidates$seqnames=as.character(hotspot_candidates$seqnames)
hotspot_candidates$CHR=ifelse(hotspot_candidates$seqnames=="chrX","23",substr(hotspot_candidates$seqnames,4,nchar(hotspot_candidates$seqnames)))
hotspot_candidates$CHR=as.numeric(hotspot_candidates$CHR)
hotspot_candidates$bp=ceiling((hotspot_candidates$start+hotspot_candidates$end)/2)
hotspot_candidates$transcript_id=hotspot_candidates$mut_region

x=hotspot_candidates
highlight=c("Mutation49512","Mutation46295") # highlight top 2 significant hotspots
cutoff=0.01/2533374732 # 0.01/n1

manhattan.hs=function(x,div,highlight,cutoff,color){
# modified manhattan function
chr="CHR"
bp="bp"
p="pval"
snp="transcript_id"
fdr="fdr"
col = c("gray9","gray49")
chrlabs=c(1:22,"X")
# highlight=NULL
logp=TRUE
annotatePval=NULL
annotateTop=TRUE
suggestiveline=FALSE
genomewideline=FALSE

CHR=BP=P=FDR=index=NULL

if (!(chr %in% names(x))) stop(paste("Column", chr, "not found!"))
if (!(bp %in% names(x))) stop(paste("Column", bp, "not found!"))
if (!(p %in% names(x))) stop(paste("Column", p, "not found!"))
if (!(fdr %in% names(x))) stop(paste("Column", fdr, "not found!"))
if (!is.numeric(x[[chr]])) stop(paste(chr, "column should be numeric. Do you have 'X', 'Y', 'MT', etc? If so change to numbers"))
if (!is.numeric(x[[bp]])) stop(paste(bp, "column should be numeric."))
if (!is.numeric(x[[p]])) stop(paste(p, "column should be numeric."))
if (!is.numeric(x[[fdr]])) stop(paste(fdr, "column should be numeric."))

d=data.frame(transcript_id=x[["transcript_id"]], CHR=x[[chr]], BP=x[[bp]], P=x[[p]], FDR=x[[fdr]])

if (!is.null(x[[snp]])) d=transform(d, SNP=x[[snp]])

d <- subset(d, (is.numeric(CHR) & is.numeric(BP) & is.numeric(P) & is.numeric(FDR)))
d <- d[order(d$CHR, d$BP),]
if(logp){
  d$logp <- -log10(d$P)
} else {
  d$logp <- d$P
}
d$pos=NA

d$index=NA
ind = 0
for (i in unique(d$CHR)){
  ind = ind + 1
  d[d$CHR==i,]$index = ind
}

nchr=length(unique(d$CHR))
if(nchr==1){ 
  d$pos=d$BP
  ticks=floor(length(d$pos))/2+1
  xlabel = paste('Chromosome', unique(d$CHR),'position')
  labs = ticks
} else { 
  lastbase=0
  ticks=NULL
  for (i in unique(d$index)){
    if (i==1){
      d[d$index==i,]$pos=d[d$index==i, ]$BP
    } else {
      lastbase=lastbase+tail(subset(d,index==i-1)$BP,1)
      d[d$index==i,]$pos=d[d$index==i,]$BP+lastbase
    }
    ticks=c(ticks,(min(d[d$index==i,]$pos)+max(d[d$index==i,]$pos))/2+1)
  }
  xlabel='Chromosome'
  labs<-unique(d$CHR)
}

xmax=ceiling(max(d$pos)*1.03)
xmin=floor(max(d$pos)*-0.03)

# def_args<-list(xaxt='n',bty='n',xaxs='i',yaxs='i',las=1,pch=20,
#                xlim=c(xmin,xmax),ylim=c(0,ceiling(max(d$logp))),
#                xlab=xlabel,ylab=expression(-log[10](italic(p))))
# dotargs <- as.list(match.call())[-1L]
# do.call("plot",c(NA,dotargs,def_args[!names(def_args) %in% names(dotargs)]))

plot(runif(10), runif(10), 
     xlim=c(xmin,xmax), ylim=c(0,ceiling(max(d$logp))), 
     axes=FALSE, #Don't plot the axis 
     type="n",  #hide the points
     ylab=expression(-log[10](italic(p))), xlab=xlabel)

axis(2, seq(0, ceiling(max(d$logp)), div))

if (!is.null(chrlabs)){
  if(is.character(chrlabs)){
    if (length(chrlabs)==length(labs)){
      labs<-chrlabs
    } else {
      warning("You're trying to specify chromosome labels but the number of labels != number of chromosomes.")
    }
  } else {
    warning ("If you're trying to specify chromosome labels, chrlabs must be a character vector")
  }
}

if(nchr==1){ 
  axis(1,...)
} else {
  axis(1, at=ticks, labels=labs,las=2,cex.axis=0.7)
}

col=rep(col, max(d$CHR))

if (nchr==1){
  with(d, points(pos, logp, pch=20, col=col[1],...))
} else {
  icol=1
  for (i in unique(d$index)){
 with(d[d$index==unique(d$index)[i],],points(pos,logp,col=col[icol],pch=16))
    icol=icol+1
  }
}

if (suggestiveline) abline(h=suggestiveline, col="blue")
if (genomewideline) abline(h=genomewideline, col="red")

par(xpd=FALSE)

  # Highlight snps from a character vector
if (!is.null(highlight)){
  if (any(!(highlight %in% d$transcript_id))) warning("You're trying to highlight SNPs that don't exist in your results.")
  d.highlight=d[which(d$transcript_id %in% highlight),]
  with(d.highlight, points(pos, logp, col=color, pch=16))
}

abline(h=-log10(cutoff),col="black")
}

manhattan.hs(x,5,highlight,cutoff,"orange")
```

## Figure C

Manhattan plot for indel gene recurrence analysis

```
mut.rec.hotspot=read.delim("LRmodel_gene_region_nonMSI_indel_remove5_edited_annotated_min1.tsv",stringsAsFactors = FALSE) #9741
results=mut.rec.hotspot
results$CHR=ifelse(results$Chromosome.scaffold.name=="X","23",results$Chromosome.scaffold.name)
results$CHR=as.numeric(results$CHR)
results$bp=ceiling(results$Gene.Start..bp.+results$Gene.End..bp.)/2
manhattan(results, chr="CHR",bp="bp",p="pval",snp="id",col = c("salmon4","red3","palegreen4","purple3","orange1","maroon1","navy","lightsteelblue4","gray8","navajowhite4"),chrlabs=c(1:22,"X"),suggestiveline = FALSE,genomewideline = FALSE)
```

```
x=results
colnames(x)[1]="transcript_id"
highlight=c("ENSG00000182333","ENSG00000096088","ENSG00000184956") # highlight top 3 significant genes
cutoff=0.01/33786 #0.01/n1

manhattan.hs(x,5,highlight,cutoff,"orange")
```

## Figure D-F

Boxplots for top 3 gene candidates

```
mut <- read.delim("LRmodel_gene_region_nonMSI_indel_remove5_edited_annotated_min1.tsv", stringsAsFactors=FALSE)

## TAN expression data
rnaseq.tan=read.table("gene_fpkm.txt", header=T, sep="\t", check.names=F)
rnaseq.tan=rnaseq.tan[,-1]

rnaseq.tan.boxplot <- function(gene, mut.samples, rnaseq.data) {
  gene.rnaseq=rnaseq.data[rnaseq.data[,1]==gene,]
  gene.rnaseq=(gene.rnaseq[-1])
  gene.df=data.frame(id=names(gene.rnaseq),exp=as.numeric(gene.rnaseq), group=c(rep("Normal",19),rep("Tumor_WT",19)),stringsAsFactors =F)
  gene.df$group[names(gene.rnaseq)%in%mut.samples]="Tumor_Mut"
  print(gene.df[gene.df$group=="Tumor_Mut",])
  print(paste("median of Tumor_Mut: ",median(gene.df$exp[gene.df$group=="Tumor_Mut"])))
  print(paste("median of Tumor_WT: ",median(gene.df$exp[gene.df$group=="Tumor_WT"])))
  print(paste("median of Normal: ",median(gene.df$exp[gene.df$group=="Normal"])))
  print(wilcox.test(gene.df$exp[gene.df$group=="Tumor_Mut"],gene.df$exp[gene.df$group=="Tumor_WT"]))
  print(wilcox.test(gene.df$exp[gene.df$group=="Tumor_WT"],gene.df$exp[gene.df$group=="Normal"]))
  if (max(gene.df$exp[gene.df$group=="Tumor_Mut"])<1&&max(gene.df$exp[gene.df$group=="Tumor_WT"])<1) {
    print(paste(gene," not expressed"))
    return()
  }  
  if(sum(gene.df$exp==0)!=0){
    gene.df$exp=gene.df$exp+1
  }
  
  p=ggplot(gene.df, aes(x=factor(group, levels = c("Normal","Tumor_WT","Tumor_Mut")), y=exp))+ stat_boxplot(geom ='errorbar')+geom_boxplot(outlier.shape = NA)+
    geom_jitter(aes(colour=group), size=2, width=0.1)+ scale_color_brewer(palette="Paired")+ylab(paste(gene,"fpkm"))+xlab(NULL)+
    theme(text = element_text(size=20),axis.text.x = element_text(size=20))+theme(legend.position="none")+
    theme(panel.grid.major = element_blank(),
          panel.grid.minor = element_blank(),
          panel.background = element_blank(),
          axis.line = element_line(colour="black"))+
    scale_y_log10() 
  print(p)
}

## TCGA expression data
rnaseq.tcga=read.table("STAD.rnaseqv2_RSEM_genes_normalized.txt", header=T, sep="\t", check.names=F, stringsAsFactors = F)
rnaseq.tcga=rnaseq.tcga[-1,]
names(rnaseq.tcga)=substr(names(rnaseq.tcga),1,15)
rnaseq.gene.id=do.call(rbind,strsplit(rnaseq.tcga[,1],"[|]"))
rnaseq.tcga[,1]=rnaseq.gene.id[,1]
tcga.samples=read.table("TCGA_sample_ID.txt", header=F, sep="\t", colClasses=c('character', 'character'))
all.tcga.samples=names(rnaseq.tcga)
tcga.tissue=substr(all.tcga.samples,14,15)
unique(tcga.tissue) # [1] "01" "11"
```

```
## [1] " R" "01" "11"
```

```
sum(tcga.tissue=="11") # 35
```

```
## [1] 35
```

```
normal.samples=all.tcga.samples[tcga.tissue=="11"]
tumor.samples=all.tcga.samples[all.tcga.samples%in%substr(tcga.samples[,1],1,15)]
rnaseq.tcga=rnaseq.tcga[,c("Hybridization R",tumor.samples,normal.samples)]

rnaseq.tcga.boxplot <- function(gene, mut.samples, rnaseq.data) {
  gene.rnaseq=rnaseq.data[rnaseq.data[,1]==gene,]
  gene.rnaseq=(gene.rnaseq[-1])
  gene.df=data.frame(id=names(gene.rnaseq),exp=as.numeric(gene.rnaseq), group=c(rep("Tumor_WT",35),rep("Normal",35)),stringsAsFactors =F)
  gene.df$group[names(gene.rnaseq)%in%mut.samples]="Tumor_Mut"
  print(gene.df[gene.df$group=="Tumor_Mut",])
  print(paste("median of Tumor_Mut: ",median(gene.df$exp[gene.df$group=="Tumor_Mut"])))
  print(paste("median of Tumor_WT: ",median(gene.df$exp[gene.df$group=="Tumor_WT"])))
  print(paste("median of Normal: ",median(gene.df$exp[gene.df$group=="Normal"])))
  print(wilcox.test(gene.df$exp[gene.df$group=="Tumor_Mut"],gene.df$exp[gene.df$group=="Tumor_WT"]))
  print(wilcox.test(gene.df$exp[gene.df$group=="Tumor_WT"],gene.df$exp[gene.df$group=="Normal"]))
  if (max(gene.df$exp[gene.df$group=="Tumor_Mut"])<5&&max(gene.df$exp[gene.df$group=="Tumor_WT"])<5) {
    print(paste(gene," not expressed"))
    return()
  }  

  if(sum(gene.df$exp==0)!=0){
    gene.df$exp=gene.df$exp+1
  }
    
  p=ggplot(gene.df, aes(x=factor(group, levels = c("Normal","Tumor_WT","Tumor_Mut")), y=exp))+ stat_boxplot(geom ='errorbar')+geom_boxplot(outlier.shape = NA)+
    geom_jitter(aes(colour=group), size=2,width=0.1)+ scale_color_brewer(palette="Paired")+ylab(paste(gene,"fpkm"))+xlab(NULL)+
    theme(text = element_text(size=20),axis.text.x = element_text(size=20))+theme(legend.position="none")+
    theme(panel.grid.major = element_blank(),
          panel.grid.minor = element_blank(),
          panel.background = element_blank(),
          axis.line = element_line(colour="black"))+
    scale_y_log10()
  print(p) 
}

maf.gastric <- maf.to.granges('gastric_RF_indels_nonMSI_prefiltered_noPoly_v2.MAF')
```

```
## [1] ">> Reading compact MAF ..."
```

```
maf.gastric=maf.gastric[-which(maf.gastric$sid %in% c("tan2001206", "tan20021007", "tan980319", "tan2000986", "tan980436"))]

chrOrder<-c(paste("chr",1:22,sep=""),"chrX")
seqi = seqinfo(Hsapiens)[seqnames(Hsapiens)[1:23]]
seqnames=seqnames(seqinfo(Hsapiens))[1:23]
```

```
mappability=import("wgEncodeCrgMapabilityAlign75mer.bigWig")
## define reads that can map to more than 1 genomic location as non-mappable
nonmappable=mappability[mappability$score<1,]
# convert zero-based coordinates to one-based coordinates
nonmappable=shift(nonmappable,1)
nonmappable= reduce(nonmappable)
nonmappable=nonmappable[seqnames(nonmappable) %in% seqnames(seqi)]
seqlevels(nonmappable)=as.character(unique(seqnames(nonmappable)))

## mask CDS and ig loci
roi.cds <- bed.to.granges('C:/Users/changmm/Documents/wgs/non_msi/script/CTCF/Figures/Figure3/Ensembl75.CDS.bed')
roi.cds.ext <- reduce(roi.cds + 5) # extend each region with +/- 5 bases and get all non-overlapping regions
# immunoglobulin loci
ig.loci <- bed.to.granges('C:/Users/changmm/Documents/wgs/non_msi/script/CTCF/Figures/Figure3/ig_loci.bed')
ig.loci <- reduce(ig.loci + 10**5) # extend each region with 100kb and get all non-overlapping regions
# combine mask regions
# one of the ig.loci located on sequence chr14 is out-of-bound, trim at this stage as there is no seqinfo associated with ig.loci and roi.cds.ext
mask.regions=reduce(trim(c(ig.loci,roi.cds.ext,nonmappable))) 

mut.regions=read.delim("gene_coordinates_biomart_16feb17.txt",sep="\t",header=T) #37693
mut.regions=GRanges(seqnames=paste("chr",mut.regions$Chromosome.scaffold.name,sep=""),IRanges(start=mut.regions$Gene.Start..bp.,end=mut.regions$Gene.End..bp.),id=mut.regions$Gene.ID)
mut.regions=mut.regions[seqnames(mut.regions) %in% seqnames(seqi)] # 34228
mut.regions=mut.regions+1000
names(mut.regions)=mut.regions$id
mut.regions=split(mut.regions,mut.regions$id) 
mut.regions=subtract.regions.from.roi(mut.regions,mask.regions,cores=cores) # 33801
mut.regions=unlist(mut.regions) # 2736500
seqlevels(mut.regions)=as.character(unique(seqnames(mut.regions)))
mut.regions.reduced=reduce(mut.regions) # 2532641
mut.regions.reduced=as.data.frame(mut.regions.reduced)
mut.regions.reduced$seqnames=as.character(mut.regions.reduced$seqnames)
mut.regions.reduced=mut.regions.reduced[order(mut.regions.reduced$seqnames,mut.regions.reduced$start),]
mut.regions.reduced=GRanges(seqnames=mut.regions.reduced$seqnames,IRanges(mut.regions.reduced$start,mut.regions.reduced$end))
mut.regions=split(mut.regions,names(mut.regions))
# save summarized output to rds
saveRDS(mut.regions,file="fig2.mut.regions.RDS")
```

Read in summarized file

```
mut.regions=readRDS("fig2.mut.regions.RDS")
```

## Figure D

ENSG00000182333 - LIPF

```
z=findOverlaps(maf.gastric,mut.regions[[mut$id[1]]])
maf=maf.gastric[queryHits(z)] # 18 mutations
sid=unique(maf$sid) # 16 samples
print(maf)
```

```
## GRanges object with 18 ranges and 4 metadata columns:
##        seqnames               ranges strand   |
##           <Rle>            <IRanges>  <Rle>   |
##    [1]    chr10 [90425251, 90425251]      *   |
##    [2]    chr10 [90425844, 90425856]      *   |
##    [3]    chr10 [90426126, 90426127]      *   |
##    [4]    chr10 [90427564, 90427567]      *   |
##    [5]    chr10 [90429303, 90429306]      *   |
##    ...      ...                  ...    ... ...
##   [14]    chr10 [90436380, 90436384]      *   |
##   [15]    chr10 [90436785, 90436788]      *   |
##   [16]    chr10 [90437475, 90437477]      *   |
##   [17]    chr10 [90437964, 90437966]      *   |
##   [18]    chr10 [90438615, 90438616]      *   |
##                                              ral      tal          sid
##                                         <factor> <factor>     <factor>
##    [1]                                     A/A/A AT/AT/AT    HK-pfg072
##    [2] GTGGGCAAACTAT/GTGGGCAAACTAT/GTGGGCAAACTAT    G/G/G    HK-pfg317
##    [3]                            ATAAT/ATAAT/NA   A/A/NA TCGA-HU-8245
##    [4]                            CTTT/CTTT/CTTT    C/C/C    HK-pfg119
##    [5]                            CATT/CATT/CATT    C/C/C TCGA-HU-A4G6
##    ...                                       ...      ...          ...
##   [14]                         TCTAA/TCTAA/TCTAA    T/T/T TCGA-BR-7722
##   [15]                            CTCT/CTCT/CTCT    C/C/C    HK-pfg119
##   [16]                               CAA/CAA/CAA    C/C/C    HK-pfg416
##   [17]                               ATT/ATT/ATT    A/A/A    HK-pfg130
##   [18]                                  AT/AT/AT    A/A/A TCGA-D7-6518
##           ctype
##        <factor>
##    [1]       HK
##    [2]       HK
##    [3]     TCGA
##    [4]       HK
##    [5]     TCGA
##    ...      ...
##   [14]     TCGA
##   [15]       HK
##   [16]       HK
##   [17]       HK
##   [18]     TCGA
##   -------
##   seqinfo: 24 sequences from an unspecified genome; no seqlengths
```

```
LIPF.mut=c("TCGA-HU-A4G6-01","TCGA-CG-4443-01","TCGA-BR-6456-01","TCGA-D7-A4YX-01","TCGA-BR-7722-01","TCGA-D7-6518-01")
rnaseq.tcga.boxplot("LIPF", LIPF.mut, rnaseq.tcga)
```

```
##                 id        exp     group
## 4  TCGA-BR-6456-01 43492.8717 Tumor_Mut
## 6  TCGA-BR-7722-01    44.6271 Tumor_Mut
## 16 TCGA-CG-4443-01     0.0000 Tumor_Mut
## 20 TCGA-D7-6518-01   159.0146 Tumor_Mut
## 27 TCGA-D7-A4YX-01     0.0000 Tumor_Mut
## 33 TCGA-HU-A4G6-01     0.0000 Tumor_Mut
## [1] "median of Tumor_Mut:  22.31355"
## [1] "median of Tumor_WT:  5.3174"
## [1] "median of Normal:  3341.1064"
```

```
## Warning in wilcox.test.default(gene.df$exp[gene.df$group == "Tumor_Mut"], :
## cannot compute exact p-value with ties
```

```
## 
##  Wilcoxon rank sum test with continuity correction
## 
## data:  gene.df$exp[gene.df$group == "Tumor_Mut"] and gene.df$exp[gene.df$group == "Tumor_WT"]
## W = 82.5, p-value = 0.8594
## alternative hypothesis: true location shift is not equal to 0
```

```
## Warning in wilcox.test.default(gene.df$exp[gene.df$group == "Tumor_WT"], :
## cannot compute exact p-value with ties
```

```
## 
##  Wilcoxon rank sum test with continuity correction
## 
## data:  gene.df$exp[gene.df$group == "Tumor_WT"] and gene.df$exp[gene.df$group == "Normal"]
## W = 265.5, p-value = 0.001102
## alternative hypothesis: true location shift is not equal to 0
```

## Figure E

ENSG00000096088 - PGC

```
z=findOverlaps(maf.gastric,mut.regions[[mut$id[2]]])
maf=maf.gastric[queryHits(z)] # 7 mutations
sid=unique(maf$sid) # 7 samples
print(maf)
```

```
## GRanges object with 7 ranges and 4 metadata columns:
##       seqnames               ranges strand |                  ral      tal
##          <Rle>            <IRanges>  <Rle> |             <factor> <factor>
##   [1]     chr6 [41703919, 41703923]      * |    TTGTC/TTGTC/TTGTC    T/T/T
##   [2]     chr6 [41709386, 41709391]      * | AAAAAC/AAAAAC/AAAAAC    A/A/A
##   [3]     chr6 [41709386, 41709391]      * | AAAAAC/AAAAAC/AAAAAC    A/A/A
##   [4]     chr6 [41709386, 41709391]      * | AAAAAC/AAAAAC/AAAAAC    A/A/A
##   [5]     chr6 [41709397, 41709401]      * |    AAAAC/AAAAC/AAAAC    A/A/A
##   [6]     chr6 [41710362, 41710362]      * |                G/G/G GT/GT/GT
##   [7]     chr6 [41714668, 41714668]      * |                C/C/C CT/CT/CT
##                sid    ctype
##           <factor> <factor>
##   [1]    HK-pfg030       HK
##   [2]     apollo10      tan
##   [3]    HK-pfg052       HK
##   [4]    HK-pfg144       HK
##   [5]    HK-pfg132       HK
##   [6] TCGA-HU-8608     TCGA
##   [7] TCGA-F1-6875     TCGA
##   -------
##   seqinfo: 24 sequences from an unspecified genome; no seqlengths
```

```
PGC.mut=c("TCGA-HU-8608-01", "TCGA-F1-6875-01")
rnaseq.tcga.boxplot("PGC", PGC.mut, rnaseq.tcga)
```

```
##                 id     exp     group
## 29 TCGA-F1-6875-01 42.2049 Tumor_Mut
## 32 TCGA-HU-8608-01 22.7355 Tumor_Mut
## [1] "median of Tumor_Mut:  32.4702"
## [1] "median of Tumor_WT:  788.1893"
## [1] "median of Normal:  13724.0577"
## 
##  Wilcoxon rank sum test
## 
## data:  gene.df$exp[gene.df$group == "Tumor_Mut"] and gene.df$exp[gene.df$group == "Tumor_WT"]
## W = 9, p-value = 0.1008
## alternative hypothesis: true location shift is not equal to 0
```

```
## Warning in wilcox.test.default(gene.df$exp[gene.df$group == "Tumor_WT"], :
## cannot compute exact p-value with ties
```

```
## 
##  Wilcoxon rank sum test with continuity correction
## 
## data:  gene.df$exp[gene.df$group == "Tumor_WT"] and gene.df$exp[gene.df$group == "Normal"]
## W = 456, p-value = 0.1376
## alternative hypothesis: true location shift is not equal to 0
```

## Figure F

ENSG00000184956 - MUC6

```
z=findOverlaps(maf.gastric,mut.regions[[mut$id[3]]])
maf=maf.gastric[queryHits(z)] # 8 mutations
sid=unique(maf$sid) # 8 sids
print(maf)
```

```
## GRanges object with 8 ranges and 4 metadata columns:
##       seqnames             ranges strand |
##          <Rle>          <IRanges>  <Rle> |
##   [1]    chr11 [1014276, 1014284]      * |
##   [2]    chr11 [1015117, 1015122]      * |
##   [3]    chr11 [1022995, 1022996]      * |
##   [4]    chr11 [1024712, 1024721]      * |
##   [5]    chr11 [1028470, 1028474]      * |
##   [6]    chr11 [1032259, 1032261]      * |
##   [7]    chr11 [1032670, 1032672]      * |
##   [8]    chr11 [1032750, 1032754]      * |
##                                    ral      tal               sid    ctype
##                               <factor> <factor>          <factor> <factor>
##   [1]    GGCCTGTGT/GGCCTGTGT/GGCCTGTGT    G/G/G      TCGA-CG-4474     TCGA
##   [2]             CGAGGT/CGAGGT/CGAGGT    C/C/C         HK-pfg167       HK
##   [3]                         TA/TA/TA    T/T/T CGP_donor_GC00002     ICGC
##   [4] TCTCTGCACC/TCTCTGCACC/TCTCTGCACC    T/T/T         HK-pfg217       HK
##   [5]                TCCTC/TCCTC/TCCTC    T/T/T CGP_donor_GC00049     ICGC
##   [6]                      CGT/CGT/CGT    C/C/C CGP_donor_GC00020     ICGC
##   [7]                      GGT/GGT/GGT    G/G/G CGP_donor_GC00051     ICGC
##   [8]                GTGTC/GTGTC/GTGTC    G/G/G         HK-pfg035       HK
##   -------
##   seqinfo: 24 sequences from an unspecified genome; no seqlengths
```

```
MUC6.mut=c("TCGA-CG-4474-01")
rnaseq.tcga.boxplot("MUC6", MUC6.mut, rnaseq.tcga)
```

```
##                 id      exp     group
## 17 TCGA-CG-4474-01 4441.617 Tumor_Mut
## [1] "median of Tumor_Mut:  4441.6168"
## [1] "median of Tumor_WT:  163.7447"
## [1] "median of Normal:  7647.2149"
## 
##  Wilcoxon rank sum test
## 
## data:  gene.df$exp[gene.df$group == "Tumor_Mut"] and gene.df$exp[gene.df$group == "Tumor_WT"]
## W = 26, p-value = 0.5143
## alternative hypothesis: true location shift is not equal to 0
## 
## 
##  Wilcoxon rank sum test
## 
## data:  gene.df$exp[gene.df$group == "Tumor_WT"] and gene.df$exp[gene.df$group == "Normal"]
## W = 445, p-value = 0.07261
## alternative hypothesis: true location shift is not equal to 0
```
