# Supplementary material for: Mutation hotspots at CTCF binding sites coupled to chromosomal instability in gastrointestinal cancers
Source: Nat Commun. 2018 Apr 18;9:1520. doi: 10.1038/s41467-018-03828-2 (PMC5906695; doi:10.1038/s41467-018-03828-2)
Supplement: Supplementary file 8 — Supplementary Data 5 [file 41467_2018_3828_MOESM8_ESM.zip › Rmarkdowns/Figure 3/Figure3_CBS_SNV_hotspots_rev.html]

Figure 3 - SNV/CBS hotspots


# Figure 3 - SNV/CBS hotspots

This is the R Markdown for Figure 3, which consists of 3 parts.

## Figure A

Manhattan plot for SNV hotspots

```
hotspot_candidates <- read.delim("LRmodel_hotspot_nonMSI_prefiltered-5_corrected.tsv", stringsAsFactors=FALSE) # 103627
hotspot_candidates$CHR=ifelse(hotspot_candidates$chrom=="chrX","23",substr(hotspot_candidates$chrom,4,nchar(hotspot_candidates$chrom)))
hotspot_candidates$CHR=as.numeric(hotspot_candidates$CHR)
hotspot_candidates$bp=ceiling((hotspot_candidates$start+hotspot_candidates$end)/2)
hotspot_candidates$mut_region=rownames(hotspot_candidates)
hotspot_candidates$id=hotspot_candidates$mut_region
hotspot_candidates=GRanges(seqnames=hotspot_candidates$chrom,
                           IRanges(start=hotspot_candidates$start,end=hotspot_candidates$end),
                           pval=hotspot_candidates$pval,
                           fdr=hotspot_candidates$fdr,
                           mut_region=hotspot_candidates$mut_region,
                           length=hotspot_candidates$length,
                           p.bg=hotspot_candidates$p.bg,
                           k=hotspot_candidates$k)
# plot only the unique hotspots
hotspot=reduce(hotspot_candidates) #64988
hotspot$hotspot=c(1:length(hotspot))
z=findOverlaps(hotspot_candidates,hotspot)
t=as.data.frame(hotspot_candidates[queryHits(z)])
t$hotspot=hotspot[subjectHits(z)]$hotspot
t=t[order(t$pval,decreasing=FALSE),] #103627
q=t[!duplicated(t$hotspot),] #64988
hotspot_candidates=q

hotspot_candidates$seqnames=as.character(hotspot_candidates$seqnames)
hotspot_candidates$CHR=ifelse(hotspot_candidates$seqnames=="chrX","23",substr(hotspot_candidates$seqnames,4,nchar(hotspot_candidates$seqnames)))
hotspot_candidates$CHR=as.numeric(hotspot_candidates$CHR)
hotspot_candidates$bp=ceiling((hotspot_candidates$start+hotspot_candidates$end)/2)
hotspot_candidates$transcript_id=hotspot_candidates$mut_region

x=hotspot_candidates

manhattan.hs=function(x,div,highlight,cutoff,color){
# modified manhattan function
chr="CHR"
bp="bp"
p="pval"
snp="transcript_id"
fdr="fdr"
col = c("gray9","gray49")
chrlabs=c(1:22,"X")
# highlight=NULL
logp=TRUE
annotatePval=NULL
annotateTop=TRUE
suggestiveline=FALSE
genomewideline=FALSE

CHR=BP=P=FDR=index=NULL

if (!(chr %in% names(x))) stop(paste("Column", chr, "not found!"))
if (!(bp %in% names(x))) stop(paste("Column", bp, "not found!"))
if (!(p %in% names(x))) stop(paste("Column", p, "not found!"))
if (!(fdr %in% names(x))) stop(paste("Column", fdr, "not found!"))
if (!is.numeric(x[[chr]])) stop(paste(chr, "column should be numeric. Do you have 'X', 'Y', 'MT', etc? If so change to numbers"))
if (!is.numeric(x[[bp]])) stop(paste(bp, "column should be numeric."))
if (!is.numeric(x[[p]])) stop(paste(p, "column should be numeric."))
if (!is.numeric(x[[fdr]])) stop(paste(fdr, "column should be numeric."))

d=data.frame(transcript_id=x[["transcript_id"]], CHR=x[[chr]], BP=x[[bp]], P=x[[p]], FDR=x[[fdr]])

if (!is.null(x[[snp]])) d=transform(d, SNP=x[[snp]])

d <- subset(d, (is.numeric(CHR) & is.numeric(BP) & is.numeric(P) & is.numeric(FDR)))
d <- d[order(d$CHR, d$BP),]
if(logp){
  d$logp <- -log10(d$P)
} else {
  d$logp <- d$P
}
d$pos=NA

d$index=NA
ind = 0
for (i in unique(d$CHR)){
  ind = ind + 1
  d[d$CHR==i,]$index = ind
}

nchr=length(unique(d$CHR))
if(nchr==1){ 
  d$pos=d$BP
  ticks=floor(length(d$pos))/2+1
  xlabel = paste('Chromosome', unique(d$CHR),'position')
  labs = ticks
} else { 
  lastbase=0
  ticks=NULL
  for (i in unique(d$index)){
    if (i==1){
      d[d$index==i,]$pos=d[d$index==i, ]$BP
    } else {
      lastbase=lastbase+tail(subset(d,index==i-1)$BP,1)
      d[d$index==i,]$pos=d[d$index==i,]$BP+lastbase
    }
    ticks=c(ticks,(min(d[d$index==i,]$pos)+max(d[d$index==i,]$pos))/2+1)
  }
  xlabel='Chromosome'
  labs<-unique(d$CHR)
}

xmax=ceiling(max(d$pos)*1.03)
xmin=floor(max(d$pos)*-0.03)

# def_args<-list(xaxt='n',bty='n',xaxs='i',yaxs='i',las=1,pch=20,
#                xlim=c(xmin,xmax),ylim=c(0,ceiling(max(d$logp))),
#                xlab=xlabel,ylab=expression(-log[10](italic(p))))
# dotargs <- as.list(match.call())[-1L]
# do.call("plot",c(NA,dotargs,def_args[!names(def_args) %in% names(dotargs)]))

plot(runif(10), runif(10), 
     xlim=c(xmin,xmax), ylim=c(0,ceiling(max(d$logp))), 
     axes=FALSE, #Don't plot the axis 
     type="n",  #hide the points
     ylab=expression(-log[10](italic(p))), xlab=xlabel)

axis(2, seq(0, ceiling(max(d$logp)), div))

if (!is.null(chrlabs)){
  if(is.character(chrlabs)){
    if (length(chrlabs)==length(labs)){
      labs<-chrlabs
    } else {
      warning("You're trying to specify chromosome labels but the number of labels != number of chromosomes.")
    }
  } else {
    warning ("If you're trying to specify chromosome labels, chrlabs must be a character vector")
  }
}

if(nchr==1){ 
  axis(1,...)
} else {
  axis(1, at=ticks, labels=labs,las=2,cex.axis=0.7)
}

col=rep(col, max(d$CHR))

if (nchr==1){
  with(d, points(pos, logp, pch=20, col=col[1],...))
} else {
  icol=1
  for (i in unique(d$index)){
 with(d[d$index==unique(d$index)[i],],points(pos,logp,col=col[icol],pch=16))
    icol=icol+1
  }
}

if (suggestiveline) abline(h=suggestiveline, col="blue")
if (genomewideline) abline(h=genomewideline, col="red")

par(xpd=FALSE)

  # Highlight snps from a character vector
if (!is.null(highlight)){
  if (any(!(highlight %in% d$transcript_id))) warning("You're trying to highlight SNPs that don't exist in your results.")
  d.highlight=d[which(d$transcript_id %in% highlight),]
  with(d.highlight, points(pos, logp, col=color, pch=16))
}

abline(h=-log10(cutoff),col="black")
}

hotspot=read.delim("LRmodel_hotspot_nonMSI_prefiltered-5_corrected.tsv", header=T, stringsAsFactors = F) #103627
hotspot$mut_region=rownames(hotspot)
hotspot=GRanges(seqnames=hotspot$chrom,IRanges(start=hotspot$start,end=hotspot$end),pval=hotspot$pval,fdr=hotspot$fdr,mut_region=hotspot$mut_region)
hotspot=hotspot[which(hotspot$pval<(0.01/2533374732))] #67
hotspot.reduce=reduce(hotspot) #34
hotspot.reduce$hotspot=paste("hs",c(1:length(hotspot.reduce)),sep="")
hs=hotspot[queryHits(findOverlaps(hotspot,hotspot.reduce))]
hs$hotspot=hotspot.reduce[subjectHits(findOverlaps(hotspot,hotspot.reduce))]$hotspot
hs$red.start=start(hotspot.reduce[subjectHits(findOverlaps(hotspot,hotspot.reduce))])
hs$red.end=end(hotspot.reduce[subjectHits(findOverlaps(hotspot,hotspot.reduce))])
hs=as.data.frame(hs)
hs=hs[order(hs$pval,decreasing=FALSE),]
hs=hs[!duplicated(hs$hotspot),]
uniq.hs=with(hs,GRanges(seqnames,IRanges(red.start,red.end),mut_region=mut_region))
roi.ctcf <- bed.to.granges("ctcf_motif_union.bed")
cbs.hotspots=uniq.hs[unique(queryHits(findOverlaps(uniq.hs,roi.ctcf)))]$mut_region #11

highlight=cbs.hotspots # highlight 11 significant CBS hotspots
cutoff=0.01/2533374732 #0.01/n1

manhattan.hs(x,5,highlight,cutoff,"maroon1")
```

## Figure B

Enrichment of hotspots

```
chrOrder<-c(paste("chr",1:22,sep=""),"chrX")
seqi = seqinfo(Hsapiens)[seqnames(Hsapiens)[1:23]]
seqnames=seqnames(seqinfo(Hsapiens))[1:23]

###
### import mask regions
###
mappability=import("wgEncodeCrgMapabilityAlign75mer.bigWig")
## define reads that can map to more than 1 genomic location as non-mappable
nonmappable=mappability[mappability$score<1,]
# convert zero-based coordinates to one-based coordinates
nonmappable=shift(nonmappable,1)
nonmappable= reduce(nonmappable)
nonmappable=nonmappable[seqnames(nonmappable) %in% seqnames(seqi)]
seqlevels(nonmappable)=as.character(unique(seqnames(nonmappable)))

# trim ends of each chromosome that are N's
mappability.grl=split(mappability, seqnames(mappability))
ranges=lapply(mappability.grl, range)
ranges=GRangesList(ranges)
ranges=unlist(ranges)
extrSeq=Views(Hsapiens,ranges) # check that all N's at chromosome ends are trimmed
# define chromosome ends to be masked
ranges=ranges[seqnames(ranges) %in% seqnames(seqi)] #2441960
seqlevels(ranges)=as.character(unique(seqnames(ranges)))
end1=GRanges(seqnames(ranges), IRanges(1, start(ranges)-1))
end2=GRanges(seqnames(ranges), IRanges(end(ranges)+1, seqlengths(seqi[as.character(seqnames(ranges))])))

## mask CDS and ig loci
roi.cds <- bed.to.granges('Ensembl75.CDS.bed')
roi.cds.ext <- reduce(roi.cds + 5) # extend each region with +/- 5 bases and get all non-overlapping regions
# immunoglobulin loci
ig.loci <- bed.to.granges('ig_loci.bed')
ig.loci <- reduce(ig.loci + 10**5) # extend each region with 100kb and get all non-overlapping regions
# combine mask regions
mask.regions=reduce(trim(c(ig.loci,roi.cds.ext,nonmappable,end1,end2))) 

# get a list of all mutations that pass PoN
maf.gastric <- maf.to.granges('gastric_RF_nonMSI_prefiltered.MAF') #2444729
```

```
## [1] ">> Reading compact MAF ..."
```

```
# remove 5 samples with oxidative damage
maf.gastric=maf.gastric[!maf.gastric$sid%in%c("tan2001206", "tan20021007", "tan980319", "tan2000986", "tan980436")]
maf.gastric=maf.gastric[seqnames(maf.gastric) %in% seqnames(seqi)] # 2418375
seqlevels(maf.gastric)=as.character(unique(seqnames(maf.gastric)))
maf.gastric.filtered=maf.gastric[-queryHits(findOverlaps(maf.gastric,mask.regions))] # 2323814

# define hotspot regions at fdr<0.1, and with less than 20% of mutations in PoN
lr.hotspot=read.delim("LRmodel_hotspot_nonMSI_prefiltered-5_corrected.tsv", header=T, stringsAsFactors = F) #7513
hotspot.filtered=lr.hotspot[lr.hotspot$pval<(0.01/2533374732),] #67
hotspot.ranges=with(hotspot.filtered, GRanges(chrom, IRanges(start, end), id=hotspot.filtered$mut_region))
hotspot.ranges=reduce(hotspot.ranges) # 34 unique ranges

# get a list of hotspot mutations and a list of non hotspot mutations
hotspot.muts=maf.gastric.filtered[queryHits(findOverlaps(maf.gastric.filtered,hotspot.ranges))] # 241
non.hotspot.muts=maf.gastric.filtered[-queryHits(findOverlaps(maf.gastric.filtered,hotspot.ranges))] # 2323573

# import functional regions bigwig; use the overlap between ENCODE tf-binding peaks and DNaseI in E094
tf.binding.region=import("AllTFBS_ovl.bw",as='RleList')
tf.binding.region=tf.binding.region[1:23]

# count length of genome after removing mask regions
tot.length=sapply(tf.binding.region, function(f) {sum(runLength(f))})
tot.length=sum(as.numeric(tot.length))-sum(width(mask.regions)) # 2648619504

# get a genomic range of functional regions
tf.binding.gr=lapply(names(tf.binding.region), function(f) {v=slice(tf.binding.region[[f]], 0.01); GRanges(f, v@ranges)})
tf.binding.gr=suppressWarnings(do.call(c, tf.binding.gr))
# mask tf.binding regions
tf.binding.gr=subtract.regions.from.roi(tf.binding.gr, mask.regions, cores=1) # cores=4 

# count length of genome in tf binding regions
tf.binding.length=sum(width(tf.binding.gr)) # 27020529

# count fraction of hotspot mutations in functional regions
hotspot.muts.tf=hotspot.muts[queryHits(findOverlaps(hotspot.muts, tf.binding.gr))] #86

# count fraction of non hotspot mutations in functional regions
non.hotspot.muts.tf=non.hotspot.muts[queryHits(findOverlaps(non.hotspot.muts, tf.binding.gr))] #13447
```

```
# import functional regions in bed format
functional.region.urls=read.table('functional_regions_urls.txt')
# only consider gerp scores
functional.region.urls=functional.region.urls[1:2,]
functional.regions = lapply(functional.region.urls[,2],function(f) {bed.to.granges(as.character(f))})
names(functional.regions)=functional.region.urls[,1]
functional.regions=reduce(c(functional.regions$gerp37, functional.regions$grep21))

# save summarized output to rds
saveRDS(functional.regions,"functional.regions.RDS")
```

Read in summarized files

```
functional.regions=readRDS("functional.regions.RDS")
```

```
hotspot.enrichment <- function(hotspot.muts, non.hotspot.muts, functional.region) {
  functional.region=functional.region[seqnames(functional.region)!="chrY"]
  functional.region=subtract.regions.from.roi(functional.region, mask.regions, cores=1) # cores=4 
  
  # count fraction of genome in functional regions
  functional.length=sum(width(functional.region))
  
  # count fraction of hotspot mutations in functional regions
  hotspot.muts.f=hotspot.muts[queryHits(findOverlaps(hotspot.muts, functional.region))]
  
  # count fraction of non hotspot mutations in functional regions
  non.hotspot.muts.f=non.hotspot.muts[queryHits(findOverlaps(non.hotspot.muts, functional.region))]
  c(functional.length,length(hotspot.muts.f), length(non.hotspot.muts.f))
}  

#results=sapply(functional.regions, function(f){hotspot.enrichment(hotspot.muts, non.hotspot.muts,f)})
results=hotspot.enrichment(hotspot.muts, non.hotspot.muts,functional.regions)
results=cbind(TF.binding=c(tf.binding.length,length(hotspot.muts.tf), length(non.hotspot.muts.tf)), results)
frac=apply(results,2, function(f){f/c(tot.length,length(hotspot.muts), length(non.hotspot.muts))})
od=apply(frac,2,function(x){od.hotspot=(x[2]/(1-x[2]))/(x[1]/(1-x[1]));
  od.non.hotspot=(x[3]/(1-x[3]))/(x[1]/(1-x[1]))
  return(c(od.hotspot,od.non.hotspot))})
row.names(od)=c("hotspot","non.hotspot")

se.lod.hotspot=apply(results,2, function(x) sqrt(1/x[2]+1/(length(hotspot.muts)-x[2])+1/x[1]+1/(tot.length-x[1])))
se.lod.nonhotspot=apply(results,2,function(x) sqrt(1/x[3]+1/(length(non.hotspot.muts)-x[3])+1/x[1]+1/(tot.length-x[1])))
se.lod=c(rbind(se.lod.hotspot,se.lod.nonhotspot))

df=melt(od)
limits=aes(ymax = log(df$value) + se.lod, ymin = log(df$value) - se.lod)
colnames(df)[1]="Var1"
colnames(df)[2]="Var2"
df$Var2=as.character(df$Var2)
df$Var2=ifelse(df$Var2=="results","conservation",df$Var2)
df$Var2=factor(df$Var2,levels=c("TF.binding","conservation"))
plot<-ggplot(df, aes(x=Var2, y=log(value), fill=Var1)) + geom_bar(stat="identity",position=position_dodge())+
  geom_errorbar(limits, position = position_dodge(0.9), width = 0.25)+
  labs(x = "Functional regions", y = "log odds ratio") +
  ggtitle("Enrichment of hotspot mutations in functional regions")+
  theme(axis.text.x=element_text(size=20,angle=90,vjust=1))+
  theme(panel.grid.major = element_blank(),
        panel.grid.minor = element_blank(),
        panel.background = element_blank(),
        axis.line = element_line(colour="black"))

print(plot)
```

## Figure C part 1

CBS hotspots

```
hotspot=read.delim("LRmodel_hotspot_nonMSI_prefiltered-5_corrected.tsv", header=T, stringsAsFactors = F) #103627
hotspot=GRanges(seqnames=hotspot$chrom,IRanges(start=hotspot$start,end=hotspot$end),pval=hotspot$pval,fdr=hotspot$fdr)
hotspot=hotspot[which(hotspot$pval<(0.01/2533374732))] #67
hotspot=reduce(hotspot) #34
hotspot$hotspot=paste("hotspot",c(1:length(hotspot)),sep="")

###
### overlap with CTCF motif
###
ctcf.motif=read.table("fimo_all.txt",sep="\t") # 1751592 9
unique(ctcf.motif$V4-ctcf.motif$V3)+1 # 19
```

```
## [1] 19
```

```
ctcf.motif=GRanges(seqnames=ctcf.motif$V2,IRanges(start=ctcf.motif$V3,end=ctcf.motif$V4),pval=ctcf.motif$V7,qval=ctcf.motif$V8)

z=findOverlaps(hotspot,ctcf.motif)
ctcf_motif=hotspot[queryHits(z)] # 13
ctcf_motif=as.data.frame(ctcf_motif) 
ctcf_motif=unique(ctcf_motif) # 11

df1=data.frame(hotspot=paste("hotspot",c(1:34),sep=""),ctcf_motif=numeric(34))
df1=merge(df1,ctcf_motif,by="hotspot",all.x=TRUE)
sum(is.na(df1$seqnames)) #23
```

```
## [1] 23
```

```
df1$ctcf_motif=ifelse(is.na(df1$seqnames),0,1)
df1$hotspot=as.character(df1$hotspot)
df1$hotspot=factor(df1$hotspot,levels=paste("hotspot",c(1:34),sep=""))
df1$type="ctcf_motif"
df1$val=1

df1=df1[with(df1,order(-ctcf_motif,hotspot)),]
df1$ctcf_motif=factor(df1$ctcf_motif)
df1$hotspot=as.character(df1$hotspot)
df1$hotspot=factor(df1$hotspot,levels=df1$hotspot)

###
### in conserved region? choose most significant hotspot for that hotspot region
###
k <- read.delim("gastric_hotspot_snv_unique_annotated_remove5_corrected.tsv", stringsAsFactors=FALSE)
k=GRanges(seqnames=k$chrom,IRanges(start=k$start,end=k$end),mut_region=k$mut_region,pval=k$pval,gerp37=k$gerp37)

z=findOverlaps(k,hotspot) #34
k=k[queryHits(z)]
k=as.data.frame(k)
k$hotspot=hotspot[subjectHits(z)]$hotspot
k=k[order(k$pval),]
k$gerp37=ifelse(is.na(k$gerp37),0,k$gerp37)

df2=aggregate(gerp37~hotspot,k,unique)

df2$cons=ifelse(df2$gerp37=="0",0,1)
df2$cons=factor(df2$cons)
df2$type="conservation"
df2$val=1

df2$hotspot=as.character(df2$hotspot)
df2$hotspot=factor(df2$hotspot,levels=df1$hotspot)

###
### overlap with DNase peak
###
dnase.peak=import("E094-DNase.all.peaks.bed")
sum(dnase.peak$score>0) # 111721
```

```
## [1] 111721
```

```
dnase.fdr=read.table("E094-DNase.fdr0.01.peaks.bed")
dnase.fdr=GRanges(seqnames=dnase.fdr$V1,IRanges(start=dnase.fdr$V2,end=dnase.fdr$V3))

dnase.mac=read.table("E094-DNase.macs2.narrowPeak")
dnase.mac=GRanges(seqnames=dnase.mac$V1,IRanges(start=dnase.mac$V2,dnase.mac$V3))

dnase.peak=GRanges(seqnames=seqnames(dnase.peak),IRanges(start=start(dnase.peak),end=end(dnase.peak)))
dnase=c(dnase.peak,dnase.fdr,dnase.mac) # 482077
dnase=reduce(dnase) # 223139

z=findOverlaps(hotspot,dnase) # 11
dnase_peak=hotspot[queryHits(z)]
dnase_peak=as.data.frame(dnase_peak)
dnase_peak=unique(dnase_peak) # 11

df3=data.frame(hotspot=paste("hotspot",c(1:34),sep=""),dnase_peak=numeric(34))
df3=merge(df3,dnase_peak,by="hotspot",all.x=TRUE)
sum(is.na(df3$seqnames)) #23
```

```
## [1] 23
```

```
df3$dnase_peak=ifelse(is.na(df3$seqnames),0,1)
df3$hotspot=as.character(df3$hotspot)
df3$hotspot=factor(df3$hotspot,levels=df1$hotspot)
df3$dnase_peak=factor(df3$dnase_peak)
df3$type="dnase_peak"
df3$val=1

###
### overlap with DNase and alltfbs peaks --> TF binding sites
###
tf=import("all_tfbs.bw")
sum(tf$score>0) # 1520159
```

```
## [1] 1520159
```

```
z=findOverlaps(hotspot,tf) # 12
tf_peak=hotspot[queryHits(z)] 
tf_peak=unique(tf_peak) # 12

z=findOverlaps(tf_peak,dnase) # 11
tf_peak=tf_peak[queryHits(z)]
tf_peak=as.data.frame(tf_peak)
tf_peak=unique(tf_peak) # 11

df5=data.frame(hotspot=paste("hotspot",c(1:34),sep=""),tf_peak=numeric(34))

df5=merge(df5,tf_peak,by="hotspot",all.x=TRUE)
sum(is.na(df5$seqnames)) #23
```

```
## [1] 23
```

```
df5$tf_peak=ifelse(is.na(df5$seqnames),0,1)
df5$hotspot=as.character(df5$hotspot)
df5$hotspot=factor(df5$hotspot,levels=df1$hotspot)
df5$tf_peak=factor(df5$tf_peak)
df5$type="tf_peak"
df5$val=1

###
### in gene region? choose most significant hotspot for that hotspot region
###
k <- read.delim("gastric_hotspot_snv_unique_annotated_remove5_corrected.tsv", stringsAsFactors=FALSE) 
k=GRanges(seqnames=k$chrom,IRanges(start=k$start,end=k$end),mut_region=k$mut_region,pval=k$pval,gene=k$gene)

z=findOverlaps(k,hotspot) #74
k=k[queryHits(z)]
k=as.data.frame(k)
k$hotspot=hotspot[subjectHits(z)]$hotspot
k=k[order(k$pval),]
k$gene=ifelse(is.na(k$gene),0,k$gene)

df6=aggregate(gene~hotspot,k,unique)

df6$ingene=ifelse(df6$gene=="0",0,1)
df6$ingene=factor(df6$ingene)
df6$type="gene"
df6$val=1

df6$hotspot=as.character(df6$hotspot)
df6$hotspot=factor(df6$hotspot,levels=df1$hotspot)

x1=df1[,c("hotspot","ctcf_motif","type")]
colnames(x1)=c("hotspot","val","type")
x2=df2[,c("hotspot","cons","type")]
colnames(x2)=c("hotspot","val","type")
x3=df3[,c("hotspot","dnase_peak","type")]
colnames(x3)=c("hotspot","val","type")
x5=df5[,c("hotspot","tf_peak","type")]
colnames(x5)=c("hotspot","val","type")
x6=df6[,c("hotspot","ingene","type")]
colnames(x6)=c("hotspot","val","type")

df=rbind(x1,x2,x3,x5,x6)
df$type=factor(df$type,levels=c("gene","conservation","dnase_peak","tf_peak","ctcf_motif"))

ggplot(df,aes(x=type,y=hotspot,fill=val))+geom_tile(color="white")+
  theme(axis.text.x = element_text(angle = 90, hjust = 1,size=9))+
  scale_fill_manual(values=c("#CCCCCC","#000000"))+
  theme(panel.grid.major = element_blank(),
        panel.grid.minor = element_blank(),
        panel.background = element_blank(),
        axis.line = element_line(colour="black"))
```

## Figure C part 2

Mutations in CBS hotspots

```
###
### For all samples (192)
###
maf.gastric <- maf.to.granges('gastric_RF_prefiltered.MAF') # 4143709, 192 unique sids
```

```
## [1] ">> Reading compact MAF ..."
```

```
maf.gastric=maf.gastric[-which(maf.gastric$sid %in% c("tan2001206", "tan20021007", "tan980319", "tan2000986", "tan980436"))] # 4119812, 187 unique sids

z=findOverlaps(maf.gastric,hotspot) #260
df=maf.gastric[queryHits(z)] 
nrow(unique(as.data.frame(df))) #260
```

```
## [1] 260
```

```
df=as.data.frame(df)
df$hotspot=hotspot[subjectHits(z)]$hotspot
nrow(unique(df[,c("hotspot","sid")])) #254
```

```
## [1] 254
```

```
df2=aggregate(width~sid+hotspot,df,sum) #254
df2$sid=as.character(df2$sid)

df3=data.frame(hotspot=rep(paste("hotspot",1:34,sep=""),187),samples=rep(unique(maf.gastric$sid),each=34),count=numeric(187*34)) #6358
df3=merge(df3,df2,by.x=c("hotspot","samples"),by.y=c("hotspot","sid"),all.x=TRUE)
df3$count=df3$width
df3$count=ifelse(is.na(df3$count),0,df3$count)
df3$hotspot=factor(df3$hotspot,levels=unique(df3$hotspot))

subtype_classification <- read.delim("subtype_classification.txt", stringsAsFactors=FALSE)
GS=subtype_classification[which(subtype_classification$Molecular.Subtype=="GS"),"Sample.ID"] # 19
GS=c(GS,"apollo1_new") # 20
CIN=subtype_classification[which(subtype_classification$Molecular.Subtype=="CIN"),"Sample.ID"] # 42
EBV=subtype_classification[which(subtype_classification$Molecular.Subtype=="EBV"),"Sample.ID"] # 17
MSI=subtype_classification[which(subtype_classification$Molecular.Subtype=="MSI"),"Sample.ID"] 
MSI=c(MSI,"CGP_donor_GC00031") # 19

subtype=c(CIN[which(CIN %in% maf.gastric$sid)],GS[which(GS %in% maf.gastric$sid)],EBV[which(EBV %in% maf.gastric$sid)],MSI[which(MSI %in% maf.gastric$sid)]) # 88
t=unique(maf.gastric$sid) # 187
t=as.character(t)
df3$hotspot=as.character(df3$hotspot)
df3$hotspot=factor(df3$hotspot,levels=levels(df1$hotspot))
df3$samples=as.character(df3$samples)
df3$samples=factor(df3$samples,levels=c(subtype,t[-which(t %in% subtype)]))

###
### For samples with subtype
###
x2=df2[which(df2$sid %in% c(GS,CIN,EBV,MSI)),] # 125
length(unique(x2$sid)) # 47
```

```
## [1] 47
```

```
x3=data.frame(hotspot=rep(paste("hotspot",1:34,sep=""),88),samples=rep(subtype,each=34),count=numeric(88*34)) # 2992
x3=merge(x3,x2,by.x=c("hotspot","samples"),by.y=c("hotspot","sid"),all.x=TRUE)
x3$count=x3$width
x3$count=ifelse(is.na(x3$count),0,x3$count)
x3$samples=as.character(x3$samples)
x3$samples=factor(x3$samples,levels=subtype)

table(x3$count)
```

```
## 
##    0    1    2 
## 2867  121    4
```

```
x3$hotspot=factor(x3$hotspot,levels=levels(df1$hotspot))
x3$count=ifelse(x3$count==2,1,x3$count)
table(x3$count)
```

```
## 
##    0    1 
## 2867  125
```

```
x3$count=factor(x3$count,levels=c(0,1))

ggplot(x3,aes(x=samples,y=hotspot,fill=count))+geom_tile()+
  theme(axis.text.x = element_text(angle = 90, hjust = 1,size=5))+
  scale_fill_manual(values=c("#CCCCCC","#000000"))+
  theme(legend.position = "none")
```

```
p1=ggplot(x3,aes(x=samples,y=hotspot,fill=count))+geom_tile()+
  theme(axis.text.x = element_text(angle = 90, hjust = 1,size=5))+
  scale_fill_manual(values=c("#CCCCCC","#000000"))+
  theme(legend.position = "none")+
  theme(axis.title.x=element_blank(),
        axis.text.x=element_blank(),
        axis.ticks.x=element_blank())+
theme(axis.text.y=element_blank())
p1
```

## Figure C part 3

Mutation count per sample

```
df=maf.gastric[which(maf.gastric$sid %in% c(CIN,MSI,EBV,GS))] # 2803519
df=as.data.frame(df)
df=aggregate(ral~sid,df,length)
colnames(df)[2]="mut.count"
df$subtype=numeric(nrow(df))
df$subtype=ifelse(df$sid %in% CIN,"CIN", df$subtype)
df$subtype=ifelse(df$sid %in% MSI,"MSI", df$subtype)
df$subtype=ifelse(df$sid %in% EBV,"EBV", df$subtype)
df$subtype=ifelse(df$sid %in% GS,"GS", df$subtype)
df$subtype=factor(df$subtype,levels=c("CIN","GS","EBV","MSI"))
df$sid=factor(df$sid,levels=subtype)

ggplot(df,aes(x=sid,y=mut.count,col=subtype))+geom_point()+
  theme(axis.text.x = element_text(angle = 90, hjust = 1,size=5))+
  scale_color_manual(values=c("#9966FF","#33FFFF","#669900","#FF6666"))+
  theme(legend.position = "none")+
  theme(panel.grid.major = element_blank(),
        panel.grid.minor = element_blank(),
        panel.background = element_blank(),
        axis.line = element_line(colour="black"))
```

```
p2=ggplot(df,aes(x=sid,y=mut.count,col=subtype))+geom_point()+ 
  theme(axis.text.x = element_text(angle = 90, hjust = 1,size=5))+
  scale_color_manual(values=c("#9966FF","#33FFFF","#669900","#FF6666"))+
  theme(legend.position = "none")+
  theme(panel.grid.major = element_blank(),
        panel.grid.minor = element_blank(),
        panel.background = element_blank(),
        axis.line = element_line(colour="black"))+ 
  theme(axis.title.x=element_blank(),
        axis.text.x=element_blank(),
        axis.ticks.x=element_blank())+
    theme(axis.text.y=element_blank()) # for grid arrange
p2
```

```
grid.arrange(arrangeGrob(p1,p2,ncol=1,nrow=2,heights=c(6,2)))
```
