# Supplementary material for: Mutation hotspots at CTCF binding sites coupled to chromosomal instability in gastrointestinal cancers
Source: Nat Commun. 2018 Apr 18;9:1520. doi: 10.1038/s41467-018-03828-2 (PMC5906695; doi:10.1038/s41467-018-03828-2)
Supplement: Supplementary file 8 — Supplementary Data 5 [file 41467_2018_3828_MOESM8_ESM.zip › Rmarkdowns/Figure 4/Figure4_CBS_CTCF_motifs_rev.html]

Figure 4 - CBS and CTCF motifs


# Figure 4 - CBS and CTCF motifs

This is the R Markdown for Figure 4, which consists of 7 parts.

Run FIMO using cluster on meme suite (threshold pval<0.01)

Download hg19 by `wget ftp://hgdownload.cse.ucsc.edu/goldenPath/hg19/bigZips/chromFa.tar.gz` and run FIMO chr by chr `tar -zxvf chromFa.tar.gz`

Download ctcf\_motif.pfm from jasper website:

A [ 87 167 281 56 8 744 40 107 851 5 333 54 12 56 104 372 82 117 402 ]

C [ 291 145 49 800 903 13 528 433 11 0 3 12 0 8 733 13 482 322 181 ]

G [ 76 414 449 21 0 65 334 48 32 903 566 504 890 775 5 507 307 73 266 ]

T [ 459 187 134 36 2 91 11 324 18 3 9 341 8 71 67 17 37 396 59 ]

Convert jaspar motif to meme motif by `jaspar2meme -pfm /mnt/projects/changmm/data/non_msi/ctcf`

Run FIMO by `qsub -cwd -pe OpenMP 1 -l mem_free=4G,h_rt=24:00:00 -b y /mnt/software/bin/fimo --oc output_chr1 --verbosity 1 --thresh 0.01 ctcf_motif.meme chr1.fa`

`sed 1d output_chr2/fimo.txt > c2.txt`

`cat output_chr1/fimo.txt c2.txt c3.txt | sed 1d > fimo_all.txt`

```
cores=1 # cores=9
```

## Figure G

Manhattan plot for CTCF specific hotspots

```
hotspot_candidates <- read.delim("LRmodel_CTCF_union_nonMSI_subtype-5_mutsigs.tsv", stringsAsFactors=FALSE) #2018
hotspot_candidates$CHR=ifelse(hotspot_candidates$seqnames=="chrX","23",substr(hotspot_candidates$seqnames,4,nchar(hotspot_candidates$seqnames)))
hotspot_candidates$CHR=as.numeric(hotspot_candidates$CHR)
hotspot_candidates$bp=ceiling((hotspot_candidates$start+hotspot_candidates$end)/2)
hotspot_candidates$mut_region=rownames(hotspot_candidates)
hotspot_candidates$id=hotspot_candidates$mut_region
hotspot_candidates=GRanges(seqnames=hotspot_candidates$seqnames,
                           IRanges(start=hotspot_candidates$start,end=hotspot_candidates$end),
                           pval=hotspot_candidates$pval,
                           fdr=hotspot_candidates$fdr,
                           mut_region=hotspot_candidates$mut_region,
                           length=hotspot_candidates$length,
                           p.bg=hotspot_candidates$p.bg,
                           k=hotspot_candidates$k)
# plot only the unique hotspots
hotspot=reduce(hotspot_candidates) #1813
hotspot$hotspot=c(1:length(hotspot))
z=findOverlaps(hotspot_candidates,hotspot)
t=as.data.frame(hotspot_candidates[queryHits(z)])
t$hotspot=hotspot[subjectHits(z)]$hotspot
t=t[order(t$pval,decreasing=FALSE),] #2018
q=t[!duplicated(t$hotspot),] #1813
hotspot_candidates=q

hotspot_candidates$seqnames=as.character(hotspot_candidates$seqnames)
hotspot_candidates$CHR=ifelse(hotspot_candidates$seqnames=="chrX","23",substr(hotspot_candidates$seqnames,4,nchar(hotspot_candidates$seqnames)))
hotspot_candidates$CHR=as.numeric(hotspot_candidates$CHR)
hotspot_candidates$bp=ceiling((hotspot_candidates$start+hotspot_candidates$end)/2)
hotspot_candidates$transcript_id=hotspot_candidates$mut_region

x=hotspot_candidates

manhattan.hs=function(x,div,highlight,cutoff,color){
# modified manhattan function
chr="CHR"
bp="bp"
p="pval"
snp="transcript_id"
fdr="fdr"
col = c("gray9","gray49")
chrlabs=c(1:22,"X")
# highlight=NULL
logp=TRUE
annotatePval=NULL
annotateTop=TRUE
suggestiveline=FALSE
genomewideline=FALSE

CHR=BP=P=FDR=index=NULL

if (!(chr %in% names(x))) stop(paste("Column", chr, "not found!"))
if (!(bp %in% names(x))) stop(paste("Column", bp, "not found!"))
if (!(p %in% names(x))) stop(paste("Column", p, "not found!"))
if (!(fdr %in% names(x))) stop(paste("Column", fdr, "not found!"))
if (!is.numeric(x[[chr]])) stop(paste(chr, "column should be numeric. Do you have 'X', 'Y', 'MT', etc? If so change to numbers"))
if (!is.numeric(x[[bp]])) stop(paste(bp, "column should be numeric."))
if (!is.numeric(x[[p]])) stop(paste(p, "column should be numeric."))
if (!is.numeric(x[[fdr]])) stop(paste(fdr, "column should be numeric."))

d=data.frame(transcript_id=x[["transcript_id"]], CHR=x[[chr]], BP=x[[bp]], P=x[[p]], FDR=x[[fdr]])

if (!is.null(x[[snp]])) d=transform(d, SNP=x[[snp]])

d <- subset(d, (is.numeric(CHR) & is.numeric(BP) & is.numeric(P) & is.numeric(FDR)))
d <- d[order(d$CHR, d$BP),]
if(logp){
  d$logp <- -log10(d$P)
} else {
  d$logp <- d$P
}
d$pos=NA

d$index=NA
ind = 0
for (i in unique(d$CHR)){
  ind = ind + 1
  d[d$CHR==i,]$index = ind
}

nchr=length(unique(d$CHR))
if(nchr==1){ 
  d$pos=d$BP
  ticks=floor(length(d$pos))/2+1
  xlabel = paste('Chromosome', unique(d$CHR),'position')
  labs = ticks
} else { 
  lastbase=0
  ticks=NULL
  for (i in unique(d$index)){
    if (i==1){
      d[d$index==i,]$pos=d[d$index==i, ]$BP
    } else {
      lastbase=lastbase+tail(subset(d,index==i-1)$BP,1)
      d[d$index==i,]$pos=d[d$index==i,]$BP+lastbase
    }
    ticks=c(ticks,(min(d[d$index==i,]$pos)+max(d[d$index==i,]$pos))/2+1)
  }
  xlabel='Chromosome'
  labs<-unique(d$CHR)
}

xmax=ceiling(max(d$pos)*1.03)
xmin=floor(max(d$pos)*-0.03)

# def_args<-list(xaxt='n',bty='n',xaxs='i',yaxs='i',las=1,pch=20,
#                xlim=c(xmin,xmax),ylim=c(0,ceiling(max(d$logp))),
#                xlab=xlabel,ylab=expression(-log[10](italic(p))))
# dotargs <- as.list(match.call())[-1L]
# do.call("plot",c(NA,dotargs,def_args[!names(def_args) %in% names(dotargs)]))

plot(runif(10), runif(10), 
     xlim=c(xmin,xmax), ylim=c(0,ceiling(max(d$logp))), 
     axes=FALSE, #Don't plot the axis 
     type="n",  #hide the points
     ylab=expression(-log[10](italic(p))), xlab=xlabel)

axis(2, seq(0, ceiling(max(d$logp)), div))

if (!is.null(chrlabs)){
  if(is.character(chrlabs)){
    if (length(chrlabs)==length(labs)){
      labs<-chrlabs
    } else {
      warning("You're trying to specify chromosome labels but the number of labels != number of chromosomes.")
    }
  } else {
    warning ("If you're trying to specify chromosome labels, chrlabs must be a character vector")
  }
}

if(nchr==1){ 
  axis(1,...)
} else {
  axis(1, at=ticks, labels=labs,las=2,cex.axis=0.7)
}

col=rep(col, max(d$CHR))

if (nchr==1){
  with(d, points(pos, logp, pch=20, col=col[1],...))
} else {
  icol=1
  for (i in unique(d$index)){
 with(d[d$index==unique(d$index)[i],],points(pos,logp,col=col[icol],pch=16))
    icol=icol+1
  }
}

if (suggestiveline) abline(h=suggestiveline, col="blue")
if (genomewideline) abline(h=genomewideline, col="red")

par(xpd=FALSE)

  # Highlight snps from a character vector
if (!is.null(highlight)){
  if (any(!(highlight %in% d$transcript_id))) warning("You're trying to highlight SNPs that don't exist in your results.")
  d.highlight=d[which(d$transcript_id %in% highlight),]
  with(d.highlight, points(pos, logp, col=color, pch=16))
}

abline(h=-log10(cutoff),col="black")
}

# find CBS hotspots to highlight, same hotspots as in figure 3A
hotspot <- read.delim("LRmodel_hotspot_nonMSI_prefiltered-5_corrected.tsv", stringsAsFactors=FALSE) #103627
hotspot$mut_region=rownames(hotspot)
hotspot=GRanges(seqnames=hotspot$chrom,IRanges(start=hotspot$start,end=hotspot$end),mut_region=hotspot$mut_region,pval=hotspot$pval,fdr=hotspot$fdr)
hotspot=hotspot[which(hotspot$pval<(0.01/2533374732))] #67
hotspot=reduce(hotspot) #34
hotspot$hotspot=c(1:length(hotspot))

# Identify CTCF hotspot
roi.ctcf <- bed.to.granges("ctcf_motif_union.bed")
ctcf.hotspot=hotspot[unique(queryHits(findOverlaps(hotspot,roi.ctcf)))] # 11

hs=GRanges(hotspot_candidates$seqnames,IRanges(hotspot_candidates$start,hotspot_candidates$end),transcript_id=hotspot_candidates$transcript_id)
z=findOverlaps(hs,ctcf.hotspot)
hs=hs[unique(queryHits(z))] # 11

highlight=hs$transcript_id
cutoff=0.01/47453 #0.01/n1

manhattan.hs(x,2,highlight,cutoff,"maroon1")
```

## Figure A-D

Frequency of CBS across subtypes

```
ctcf.motif=read.table("fimo_all.txt",sep="\t")
unique(ctcf.motif$V4-ctcf.motif$V3)+1 # 19
```

```
## [1] 19
```

```
ctcf.motif=GRanges(seqnames=ctcf.motif$V2,IRanges(start=ctcf.motif$V3,end=ctcf.motif$V4),pval=ctcf.motif$V7,qval=ctcf.motif$V8,dir=ctcf.motif$V5,motif=ctcf.motif$V9)
length(ctcf.motif)
```

```
## [1] 1751592
```

```
ctcf.peak=import("CTCF.bw")
sum(ctcf.peak$score>0)
```

```
## [1] 1147323
```

```
dnase.peak=import("E094-DNase.all.peaks.bed")
sum(dnase.peak$score>0)
```

```
## [1] 111721
```

```
dnase.fdr=read.table("E094-DNase.fdr0.01.peaks.bed")
dnase.fdr=GRanges(seqnames=dnase.fdr$V1,IRanges(start=dnase.fdr$V2,end=dnase.fdr$V3))

dnase.mac=read.table("E094-DNase.macs2.narrowPeak")
dnase.mac=GRanges(seqnames=dnase.mac$V1,IRanges(start=dnase.mac$V2,dnase.mac$V3))

dnase.peak=GRanges(seqnames=seqnames(dnase.peak),IRanges(start=start(dnase.peak),end=end(dnase.peak)))
dnase=c(dnase.peak,dnase.fdr,dnase.mac)
dnase=reduce(dnase)
length(dnase)
```

```
## [1] 223139
```

```
maf.gastric <- maf.to.granges('gastric_RF_prefiltered.MAF')
```

```
## [1] ">> Reading compact MAF ..."
```

```
maf.gastric=maf.gastric[-which(maf.gastric$sid %in% c("tan2001206", "tan20021007", "tan980319", "tan2000986", "tan980436"))] # 4119812
maf.gastric$id=c(1:length(maf.gastric))
length(maf.gastric)
```

```
## [1] 4119812
```

```
z=findOverlaps(ctcf.peak,ctcf.motif) # 183666
ctcf.peak.ovl=ctcf.peak[queryHits(z)]
ctcf.peak.ovl=as.data.frame(ctcf.peak.ovl)
ctcf.peak.ovl=unique(ctcf.peak.ovl) # 157222
ctcf.motif.ovl=ctcf.motif[subjectHits(z)]
ctcf.motif.ovl=as.data.frame(ctcf.motif.ovl)
ctcf.motif.ovl=unique(ctcf.motif.ovl) # 125228
ctcf.motif.ovl=GRanges(seqnames=ctcf.motif.ovl$seqnames,IRanges(start=ctcf.motif.ovl$start,end=ctcf.motif.ovl$end),pval=ctcf.motif.ovl$pval,qval=ctcf.motif.ovl$qval,dir=ctcf.motif.ovl$dir,motif=ctcf.motif.ovl$motif)

z=findOverlaps(dnase,ctcf.motif.ovl) # 47454
dnase.ovl=dnase[queryHits(z)]
dnase.ovl=as.data.frame(dnase.ovl)
dnase.ovl=unique(dnase.ovl) # 34018
motif.ovl=ctcf.motif.ovl[subjectHits(z)]
motif.ovl=as.data.frame(motif.ovl)
motif.ovl=unique(motif.ovl) # 47453
```

```
# save ctcf union motifs to bed file
write.table(motif.ovl[,c("seqnames","start","end")],file="ctcf_motif_union.bed")
```

```
# count the number of somatic mutations at each site
site=GRanges(seqnames=motif.ovl$seqnames,IRanges(start=(motif.ovl$start+motif.ovl$end)/2,end=(motif.ovl$start+motif.ovl$end)/2),pval=motif.ovl$pval,dir=motif.ovl$dir,motif=motif.ovl$motif)
site=site+1000
unique(width(site))
```

```
## [1] 2001
```

```
length(site)
```

```
## [1] 47453
```

```
mut=findOverlaps(site,maf.gastric) 
length(mut) # 94397
```

```
## [1] 94397
```

```
mut.table=maf.gastric[subjectHits(mut)] 
mut.table$region.start=start(site[queryHits(mut)])
mut.table$region.end=end(site[queryHits(mut)])
mut.table$region.chr=seqnames(site[queryHits(mut)])
mut.table$pval=site[queryHits(mut)]$pval
mut.table$dir=site[queryHits(mut)]$dir
mut.table$motif=site[queryHits(mut)]$motif
length(unique(mut.table$id)) # 65966
```

```
## [1] 65966
```

```
mut.table=as.data.frame(mut.table)
mut.table=mut.table[order(mut.table$id,mut.table$pval),]
mut.table=mut.table[!duplicated(mut.table$id),] 
mut.table$seqnames=as.character(mut.table$seqnames)
mut.table$region.chr=as.character(mut.table$region.chr)
sum(mut.table$seqnames==mut.table$region.chr)
```

```
## [1] 65966
```

```
mut.table$motif.start=(mut.table$region.start+mut.table$region.end)/2 # 187 unique sids
overall.table=mut.table

# split mut.table by subtype
subtype_classification <- read.delim("subtype_classification.txt", stringsAsFactors=FALSE)
GS=subtype_classification[which(subtype_classification$Molecular.Subtype=="GS"),"Sample.ID"] # 19
GS=c(GS,"apollo1_new") # 20
CIN=subtype_classification[which(subtype_classification$Molecular.Subtype=="CIN"),"Sample.ID"] # 42
EBV=subtype_classification[which(subtype_classification$Molecular.Subtype=="EBV"),"Sample.ID"] # 17
MSI=subtype_classification[which(subtype_classification$Molecular.Subtype=="MSI"),"Sample.ID"] # 18
MSI=c(MSI,"CGP_donor_GC00031") # 19

GS=GS[which(GS %in% unique(maf.gastric$sid))] # 11
CIN=CIN[which(CIN %in% unique(maf.gastric$sid))] # 41
EBV=EBV[which(EBV %in% unique(maf.gastric$sid))] # 17
MSI=MSI[which(MSI %in% unique(maf.gastric$sid))] # 19
print(c(length(GS),length(CIN),length(EBV),length(MSI)))
```

```
## [1] 11 41 17 19
```

```
sum(unique(mut.table$sid) %in% c(MSI,EBV,GS,CIN)) # 88 out of 187 of unique sids in mut.table have subtype classified
```

```
## [1] 88
```

```
mut.table=mut.table[which(mut.table$sid  %in% c(MSI,EBV,GS,CIN)),] # 49890
# GS
gs=mut.table[which(mut.table$sid %in% GS),] # 1540
nrow(gs)
```

```
## [1] 1540
```

```
# MSI
msi=mut.table[which(mut.table$sid %in% MSI),] # 35152
nrow(msi)
```

```
## [1] 35152
```

```
# EBV
ebv=mut.table[which(mut.table$sid %in% EBV),] # 3976
nrow(ebv)
```

```
## [1] 3976
```

```
# CIN
cin=mut.table[which(mut.table$sid %in% CIN),] # 9222
nrow(cin)
```

```
## [1] 9222
```

## Figure A

CIN subtype

```
table(cin$dir)
```

```
## 
##    -    + 
## 4556 4666
```

```
cin.pos=cin[which(cin$dir=="+"),] 
cin.neg=cin[which(cin$dir=="-"),] 

cin.pos$pos=cin.pos$start-cin.pos$motif.start
sum(cin.pos$pos==0)
```

```
## [1] 3
```

```
cin.pos.df=aggregate(seqnames~pos,cin.pos,length)
colnames(cin.pos.df)[2]="count"
df=data.frame(pos=seq(-1000,1000,1),x=0)
cin.pos.df=merge(df,cin.pos.df,all.x=TRUE,by="pos")
cin.pos.df$count=ifelse(is.na(cin.pos.df$count),0,cin.pos.df$count)

cin.neg$pos=cin.neg$motif.start-cin.neg$start
sum(cin.neg$pos==0)
```

```
## [1] 3
```

```
cin.neg.df=aggregate(seqnames~pos,cin.neg,length)
colnames(cin.neg.df)[2]="count"
df=data.frame(pos=seq(-1000,1000,1),x=0)
cin.neg.df=merge(df,cin.neg.df,all.x=TRUE,by="pos")
cin.neg.df$count=ifelse(is.na(cin.neg.df$count),0,cin.neg.df$count)

cin.z=merge(cin.pos.df,cin.neg.df,by="pos")
cin.z$mut=cin.z$count.x+cin.z$count.y
cin.z$norm.mut=(cin.z$count.x+cin.z$count.y)/length(CIN)

ggplot(data=cin.z,aes(x=pos,y=norm.mut))+
  geom_line()+
  ylim(c(0,2.5))+ggtitle(paste("CTCF_CIN","n=41",sep="\n"))+
  xlab("CBS + flanking region (bp)")+
  ylab("Normalized somatic substitutions")+
  theme(panel.grid.major = element_blank(),
        panel.grid.minor = element_blank(),
        panel.background = element_blank(),
        axis.line=element_line(colour="black"))
```

```
mean.peak.cin=sum(cin.z[which(cin.z$pos %in% seq(-14,14,1)),"mut"])/(29*47453*length(CIN))
mean.flank.cin=sum(cin.z[-which(cin.z$pos %in% seq(-14,14,1)),"mut"])/((2001-29)*47453*length(CIN)) 
print(c(mean.peak.cin,mean.flank.cin,(mean.peak.cin/mean.flank.cin)))
```

```
## [1] 1.549052e-05 2.175845e-06 7.119310e+00
```

## Figure B

EBV subtype

```
table(ebv$dir)
```

```
## 
##    -    + 
## 1996 1980
```

```
ebv.pos=ebv[which(ebv$dir=="+"),] 
ebv.neg=ebv[which(ebv$dir=="-"),] 

ebv.pos$pos=ebv.pos$start-ebv.pos$motif.start
sum(ebv.pos$pos==0)
```

```
## [1] 0
```

```
ebv.pos.df=aggregate(seqnames~pos,ebv.pos,length)
colnames(ebv.pos.df)[2]="count"
df=data.frame(pos=seq(-1000,1000,1),x=0)
ebv.pos.df=merge(df,ebv.pos.df,all.x=TRUE,by="pos")
ebv.pos.df$count=ifelse(is.na(ebv.pos.df$count),0,ebv.pos.df$count)

ebv.neg$pos=ebv.neg$motif.start-ebv.neg$start
sum(ebv.neg$pos==0)
```

```
## [1] 0
```

```
ebv.neg.df=aggregate(seqnames~pos,ebv.neg,length)
colnames(ebv.neg.df)[2]="count"
df=data.frame(pos=seq(-1000,1000,1),x=0)
ebv.neg.df=merge(df,ebv.neg.df,all.x=TRUE,by="pos")
ebv.neg.df$count=ifelse(is.na(ebv.neg.df$count),0,ebv.neg.df$count)

ebv.z=merge(ebv.pos.df,ebv.neg.df,by="pos")
ebv.z$mut=ebv.z$count.x+ebv.z$count.y
ebv.z$norm.mut=(ebv.z$count.x+ebv.z$count.y)/length(EBV)

ggplot(data=ebv.z,aes(x=pos,y=norm.mut))+
  geom_line()+
  ylim(c(0,2.5))+
  ggtitle(paste("CTCF_EBV","n=17",sep="\n"))+
  xlab("CBS + flanking region (bp)")+
  ylab("Normalized somatic substitutions")+
  theme(panel.grid.major = element_blank(),
        panel.grid.minor = element_blank(),
        panel.background = element_blank(),
        axis.line = element_line(colour="black"))
```

```
mean.peak.ebv=sum(ebv.z[which(ebv.z$pos %in% seq(-14,14,1)),"mut"])/(29*47453*length(EBV))
mean.flank.ebv=sum(ebv.z[-which(ebv.z$pos %in% seq(-14,14,1)),"mut"])/((2001-29)*47453*length(EBV)) 
print(c(mean.peak.ebv,mean.flank.ebv,(mean.peak.ebv/mean.flank.ebv)))
```

```
## [1] 4.231795e-06 2.437117e-06 1.736394e+00
```

## Figure C

GS subtype

```
table(gs$dir)
```

```
## 
##   -   + 
## 792 748
```

```
gs.pos=gs[which(gs$dir=="+"),] 
gs.neg=gs[which(gs$dir=="-"),] 

gs.pos$pos=gs.pos$start-gs.pos$motif.start
sum(gs.pos$pos==0)
```

```
## [1] 0
```

```
gs.pos.df=aggregate(seqnames~pos,gs.pos,length)
colnames(gs.pos.df)[2]="count"
df=data.frame(pos=seq(-1000,1000,1),x=0)
gs.pos.df=merge(df,gs.pos.df,all.x=TRUE,by="pos")
gs.pos.df$count=ifelse(is.na(gs.pos.df$count),0,gs.pos.df$count)

gs.neg$pos=gs.neg$motif.start-gs.neg$start
sum(gs.neg$pos==0)
```

```
## [1] 1
```

```
gs.neg.df=aggregate(seqnames~pos,gs.neg,length)
colnames(gs.neg.df)[2]="count"
df=data.frame(pos=seq(-1000,1000,1),x=0)
gs.neg.df=merge(df,gs.neg.df,all.x=TRUE,by="pos")
gs.neg.df$count=ifelse(is.na(gs.neg.df$count),0,gs.neg.df$count)

gs.z=merge(gs.pos.df,gs.neg.df,by="pos")
gs.z$mut=gs.z$count.x+gs.z$count.y
gs.z$norm.mut=(gs.z$count.x+gs.z$count.y)/length(GS)

ggplot(data=gs.z,aes(x=pos,y=norm.mut))+
  geom_line()+
  ylim(c(0,2.5))+
  ggtitle(paste("CTCF_GS","n=11",sep="\n"))+
  xlab("CBS + flanking region (bp)")+
  ylab("Normalized somatic substitutions")+
  theme(panel.grid.major = element_blank(),
        panel.grid.minor=element_blank(),
        panel.background = element_blank(),
        axis.line=element_line(colour="black"))
```

```
mean.peak.gs=sum(gs.z[which(gs.z$pos %in% seq(-14,14,1)),"mut"])/(29*47453*length(GS))
mean.flank.gs=sum(gs.z[-which(gs.z$pos %in% seq(-14,14,1)),"mut"])/((2001-29)*47453*length(GS)) 
print(c(mean.peak.gs,mean.flank.gs,(mean.peak.gs/mean.flank.gs)))
```

```
## [1] 6.606108e-06 1.398940e-06 4.722222e+00
```

## Figure D

MSI subtype

```
table(msi$dir)
```

```
## 
##     -     + 
## 17491 17661
```

```
msi.pos=msi[which(msi$dir=="+"),] 
msi.neg=msi[which(msi$dir=="-"),]

msi.pos$pos=msi.pos$start-msi.pos$motif.start
sum(msi.pos$pos==0)
```

```
## [1] 6
```

```
msi.pos.df=aggregate(seqnames~pos,msi.pos,length)
colnames(msi.pos.df)[2]="count"
df=data.frame(pos=seq(-1000,1000,1),x=0)
msi.pos.df=merge(df,msi.pos.df,all.x=TRUE,by="pos")
msi.pos.df$count=ifelse(is.na(msi.pos.df$count),0,msi.pos.df$count)

msi.neg$pos=msi.neg$motif.start-msi.neg$start
sum(msi.neg$pos==0)
```

```
## [1] 9
```

```
msi.neg.df=aggregate(seqnames~pos,msi.neg,length)
colnames(msi.neg.df)[2]="count"
df=data.frame(pos=seq(-1000,1000,1),x=0)
msi.neg.df=merge(df,msi.neg.df,all.x=TRUE,by="pos")
msi.neg.df$count=ifelse(is.na(msi.neg.df$count),0,msi.neg.df$count)

msi.z=merge(msi.pos.df,msi.neg.df,by="pos")
msi.z$mut=msi.z$count.x+msi.z$count.y
msi.z$norm.mut=(msi.z$count.x+msi.z$count.y)/length(MSI)

ggplot(data=msi.z,aes(x=pos,y=norm.mut))+
  geom_line()+
  ylim(c(0,2.5))+
  ggtitle(paste("CTCF_MSI","n=19",sep="\n"))+
  xlab("CBS + flanking region (bp)")+
  ylab("Normalized somatic substitutions")+
  theme(panel.grid.major = element_blank(),
        panel.grid.minor = element_blank(),
        panel.background = element_blank(),
        axis.line = element_line(colour = "black"))
```

```
mean.peak.msi=sum(msi.z[which(msi.z$pos %in% seq(-14,14,1)),"mut"])/(29*47453*length(MSI)) 
mean.flank.msi=sum(msi.z[-which(msi.z$pos %in% seq(-14,14,1)),"mut"])/((2001-29)*47453*length(MSI)) 
print(c(mean.peak.msi,mean.flank.msi,(mean.peak.msi/mean.flank.msi)))
```

```
## [1] 2.145594e-05 1.945534e-05 1.102830e+00
```

## Figure E

Comparison of mutation rate across CBS, flanking regions and genome-wide

```
chrOrder<-c(paste("chr",1:22,sep=""),"chrX")
seqi = seqinfo(Hsapiens)[seqnames(Hsapiens)[1:23]]

## remove non-coding transcripts from the list of rois
## merge all overlapping regions
transcript.biotype=read.delim("transcript_biotype_100316.txt", sep="\t", header=T)  # 215647
coding.transcripts=transcript.biotype[transcript.biotype$"Transcript.type"=="protein_coding",2] # 90273

mappability=import("wgEncodeCrgMapabilityAlign75mer.bigWig")
## number of bases covered
sum(as.numeric(width(mappability))) # 2861334447
```

```
## [1] 2861334447
```

```
## number of bases with mappability =1. i.e. read only maps to 1 genomic location
sum(as.numeric(width(mappability[mappability$score==1,]))) # 2583922142
```

```
## [1] 2583922142
```

```
## number of bases with mappability >=0.5. i.e. read maps to at most 2 genomic locations
sum(as.numeric(width(mappability[mappability$score>=0.5,]))) # 2656835188
```

```
## [1] 2656835188
```

```
## define reads that can map to more than 1 genomic location as non-mappable
nonmappable=mappability[mappability$score<1,]
# convert zero-based coordinates to one-based coordinates
nonmappable=shift(nonmappable,1)
nonmappable= reduce(nonmappable)
nonmappable=nonmappable[seqnames(nonmappable) %in% seqnames(seqi)]
seqlevels(nonmappable)=as.character(unique(seqnames(nonmappable)))
############## remove masked regions ##############
# CDS
roi.cds <- bed.to.granges('Ensembl75.CDS.bed')
roi.cds.ext <- reduce(roi.cds + 5) # extend each region with +/- 5 bases and get all non-overlapping regions
# immunoglobulin loci
ig.loci <- bed.to.granges('ig_loci.bed')
ig.loci <- reduce(ig.loci + 10**5) # extend each region with 100kb and get all non-overlapping regions
# combine mask regions
# one of the ig.loci located on sequence chr14 is out-of-bound, trim at this stage as there is no seqinfo associated with ig.loci and roi.cds.ext
mask.regions=reduce(trim(c(ig.loci,roi.cds.ext,nonmappable))) 

all.gs=maf.gastric[which(maf.gastric$sid %in% GS)] # 121882
all.msi=maf.gastric[which(maf.gastric$sid %in% MSI)] # 1698980
all.ebv=maf.gastric[which(maf.gastric$sid %in% EBV)] # 252054
all.cin=maf.gastric[which(maf.gastric$sid %in% CIN)] # 730603
all=maf.gastric # 4119812

# mask regions
all.gs=subtract.regions.from.roi(all.gs, mask.regions, cores=cores) # 116991
all.msi=subtract.regions.from.roi(all.msi, mask.regions, cores=cores) # 1602835
all.ebv=subtract.regions.from.roi(all.ebv, mask.regions, cores=cores) # 242518
all.cin=subtract.regions.from.roi(all.cin, mask.regions, cores=cores) # 696649
all=subtract.regions.from.roi(all,mask.regions, cores=cores) # 3887076

all.gs=all.gs[-which(seqnames(all.gs)=="chrY")] # 116877
all.msi=all.msi[-which(seqnames(all.msi)=="chrY")] # 1601784
all.ebv=all.ebv[-which(seqnames(all.ebv)=="chrY")] # 242172
all.cin=all.cin[-which(seqnames(all.cin)=="chrY")] # 696005
all=all[-which(seqnames(all)=="chrY")] # 3883595

genome.gs=length(all.gs)/(2533374732*length(GS))
genome.msi=length(all.msi)/(2533374732*length(MSI))
genome.ebv=length(all.ebv)/(2533374732*length(EBV))
genome.cin=length(all.cin)/(2533374732*length(CIN))
genome.all=length(all)/(2533374732*length(unique(maf.gastric$sid)))

print(c(genome.gs,genome.msi,genome.ebv,genome.cin,genome.all))
```

```
## [1] 4.194082e-06 3.327752e-05 5.623097e-06 6.700837e-06 8.197716e-06
```

```
df=data.frame(region=rep(c("CTCF motif site +- 5bp","flank","genome wide"),4),
              subtype=rep(c("GS","MSI","EBV","CIN"),each=3),
              mean=c(mean.peak.gs,mean.flank.gs,genome.gs,
                     mean.peak.msi,mean.flank.msi,genome.msi,
                     mean.peak.ebv,mean.flank.ebv,genome.ebv,
                     mean.peak.cin,mean.flank.cin,genome.cin))
df$subtype=factor(df$subtype,levels=c("CIN","GS","EBV","MSI"))

ggplot(data=df,aes(x=subtype,y=mean,fill=region))+geom_bar(stat="identity",position="dodge",colour="black")+ylab("Mutation rate")+ggtitle("Mutation rate at peak region vs flank region for each subtype")+xlab("Subtype")+
  theme(panel.grid.major = element_blank(),
        panel.grid.minor = element_blank(),
        panel.background = element_blank(),
        axis.line = element_line(colour="black"))+
  scale_fill_brewer(palette="Blues")
```

```
# CBS mutation rate
table(overall.table$dir)
```

```
## 
##     -     + 
## 32863 33103
```

```
overall.pos=overall.table[which(overall.table$dir=="+"),] 
overall.neg=overall.table[which(overall.table$dir=="-"),] 

overall.pos$pos=overall.pos$start-overall.pos$motif.start
sum(overall.pos$pos==0)
```

```
## [1] 14
```

```
overall.pos.df=aggregate(seqnames~pos,overall.pos,length)
colnames(overall.pos.df)[2]="count"
df=data.frame(pos=seq(-1000,1000,1),x=0)
overall.pos.df=merge(df,overall.pos.df,all.x=TRUE,by="pos")
overall.pos.df$count=ifelse(is.na(overall.pos.df$count),0,overall.pos.df$count)

overall.neg$pos=overall.neg$motif.start-overall.neg$start
sum(overall.neg$pos==0)
```

```
## [1] 19
```

```
overall.neg.df=aggregate(seqnames~pos,overall.neg,length)
colnames(overall.neg.df)[2]="count"
df=data.frame(pos=seq(-1000,1000,1),x=0)
overall.neg.df=merge(df,overall.neg.df,all.x=TRUE,by="pos")
overall.neg.df$count=ifelse(is.na(overall.neg.df$count),0,overall.neg.df$count)

overall.z=merge(overall.pos.df,overall.neg.df,by="pos")
overall.z$mut=overall.z$count.x+overall.z$count.y
overall.z$norm.mut=(overall.z$count.x+overall.z$count.y)/length(unique(overall.table$sid))

ggplot(data=overall.z,aes(x=pos,y=norm.mut))+
  geom_line()+
  ylim(c(0,2.5))+ggtitle(paste("CTCF_ALL","n=187",sep="\n"))+
  xlab("CBS + flanking region (bp)")+
  ylab("Normalized somatic substitutions")+
  theme(panel.grid.major = element_blank(),
        panel.grid.minor = element_blank(),
        panel.background = element_blank(),
        axis.line=element_line(colour="black"))
```

```
mean.peak.cbs=sum(overall.z[which(overall.z$pos %in% seq(-14,14,1)),"mut"])/(29*47453*length(unique(overall.table$sid)))
mean.flank.cbs=sum(overall.z[-which(overall.z$pos %in% seq(-14,14,1)),"mut"])/((2001-29)*47453*length(unique(overall.table$sid))) 
print(c(mean.peak.cbs,mean.flank.cbs,(mean.peak.cbs/mean.flank.cbs)))
```

```
## [1] 1.092728e-05 3.609015e-06 3.027773e+00
```

```
# calculate fold change
fc.cin=mean.peak.cin/genome.cin
fc.gs=mean.peak.gs/genome.gs
fc.ebv=mean.peak.ebv/genome.ebv
fc.msi=mean.peak.msi/genome.msi
fc.cbs=mean.peak.cbs/genome.all
print(c(fc.cin,fc.gs,fc.ebv,fc.msi,fc.cbs))
```

```
## [1] 2.3117285 1.5751021 0.7525737 0.6447579 1.3329662
```

```
fc2.cin=mean.peak.cin/mean.flank.cin
fc2.gs=mean.peak.gs/mean.flank.gs
fc2.ebv=mean.peak.ebv/mean.flank.ebv
fc2.msi=mean.peak.msi/mean.flank.msi
fc2.cbs=mean.peak.cbs/mean.flank.cbs
print(c(fc2.cin,fc2.gs,fc2.ebv,fc2.msi,fc2.cbs))
```

```
## [1] 7.119310 4.722222 1.736394 1.102830 3.027773
```

## Figure F

CTCF motifs + flank 5bp

```
motifs=GRanges(seqnames=motif.ovl$seqnames,
               IRanges(motif.ovl$start,motif.ovl$end),
               dir=motif.ovl$dir,
               pval=motif.ovl$pval,
               motif=motif.ovl$motif)
motifs=motifs+5
length(motifs)
```

```
## [1] 47453
```

```
z=findOverlaps(maf.gastric,motifs)
t=as.data.frame(maf.gastric[queryHits(z)]) # 3463
t$motif.seq=as.character(seqnames(motifs[subjectHits(z)]))
t$motif.start=start(motifs[subjectHits(z)])
t$motif.end=end(motifs[subjectHits(z)])
t$dir=motifs[subjectHits(z)]$dir
t$pval=motifs[subjectHits(z)]$pval
t$motif=motifs[subjectHits(z)]$motif

t=t[order(t$id,t$pval),]
t=t[!duplicated(t$id),] # 3093
t.pos=t[which(t$dir=="+"),]
t.neg=t[which(t$dir=="-"),]
```

## Figure F part 2

All CBS mutations Reference Alignment

```
# All
ref.seq.pos=GRanges(seqnames=t.pos$seqnames,
                IRanges(t.pos$motif.start,t.pos$motif.end),
                dir=t.pos$dir,
                motif=t.pos$motif,
                mut=t.pos$start,
                alt=t.pos$tal,
                ref=t.pos$ral)
extrSeq=Views(Hsapiens,ref.seq.pos)

z=as(extrSeq,"DNAStringSet") 
dnaSeq.pos=as.data.frame(ref.seq.pos)
dnaSeq.pos$dna=as.character(z) # 1551

dnaSeq.pos$motif=toupper(dnaSeq.pos$motif)
sum(dnaSeq.pos$motif==dnaSeq.pos$dna)
```

```
## [1] 0
```

```
dnaSeq.pos$a1=substr(dnaSeq.pos$dna,1,1)
dnaSeq.pos$a2=substr(dnaSeq.pos$dna,2,2)
dnaSeq.pos$a3=substr(dnaSeq.pos$dna,3,3)
dnaSeq.pos$a4=substr(dnaSeq.pos$dna,4,4)
dnaSeq.pos$a5=substr(dnaSeq.pos$dna,5,5)
dnaSeq.pos$a6=substr(dnaSeq.pos$dna,6,6)
dnaSeq.pos$a7=substr(dnaSeq.pos$dna,7,7)
dnaSeq.pos$a8=substr(dnaSeq.pos$dna,8,8)
dnaSeq.pos$a9=substr(dnaSeq.pos$dna,9,9)
dnaSeq.pos$a10=substr(dnaSeq.pos$dna,10,10)
dnaSeq.pos$a11=substr(dnaSeq.pos$dna,11,11)
dnaSeq.pos$a12=substr(dnaSeq.pos$dna,12,12)
dnaSeq.pos$a13=substr(dnaSeq.pos$dna,13,13)
dnaSeq.pos$a14=substr(dnaSeq.pos$dna,14,14)
dnaSeq.pos$a15=substr(dnaSeq.pos$dna,15,15)
dnaSeq.pos$a16=substr(dnaSeq.pos$dna,16,16)
dnaSeq.pos$a17=substr(dnaSeq.pos$dna,17,17)
dnaSeq.pos$a18=substr(dnaSeq.pos$dna,18,18)
dnaSeq.pos$a19=substr(dnaSeq.pos$dna,19,19)
dnaSeq.pos$a20=substr(dnaSeq.pos$dna,20,20)
dnaSeq.pos$a21=substr(dnaSeq.pos$dna,21,21)
dnaSeq.pos$a22=substr(dnaSeq.pos$dna,22,22)
dnaSeq.pos$a23=substr(dnaSeq.pos$dna,23,23)
dnaSeq.pos$a24=substr(dnaSeq.pos$dna,24,24)
dnaSeq.pos$a25=substr(dnaSeq.pos$dna,25,25)
dnaSeq.pos$a26=substr(dnaSeq.pos$dna,26,26)
dnaSeq.pos$a27=substr(dnaSeq.pos$dna,27,27)
dnaSeq.pos$a28=substr(dnaSeq.pos$dna,28,28)
dnaSeq.pos$a29=substr(dnaSeq.pos$dna,29,29)

ref.seq.neg=GRanges(seqnames=t.neg$seqnames,
                    IRanges(t.neg$motif.start,t.neg$motif.end),
                    dir=t.neg$dir,
                    motif=t.neg$motif,
                    mut=t.neg$start,
                    alt=t.neg$tal,
                    ref=t.neg$ral)
extrSeq=Views(Hsapiens,ref.seq.neg)

z=as(extrSeq,"DNAStringSet") 
dnaSeq.neg=as.data.frame(ref.seq.neg)
dnaSeq.neg$dna=as.character(z) # 1542

dnaSeq.neg$motif=toupper(dnaSeq.neg$motif)
sum(dnaSeq.neg$motif==dnaSeq.neg$dna) # 0
```

```
## [1] 0
```

```
z=reverseComplement(z)
dnaSeq.neg$rev.dna=as.character(z)
sum(dnaSeq.neg$motif==dnaSeq.neg$rev.dna)
```

```
## [1] 0
```

```
dnaSeq.neg$a1=substr(dnaSeq.neg$rev.dna,1,1)
dnaSeq.neg$a2=substr(dnaSeq.neg$rev.dna,2,2)
dnaSeq.neg$a3=substr(dnaSeq.neg$rev.dna,3,3)
dnaSeq.neg$a4=substr(dnaSeq.neg$rev.dna,4,4)
dnaSeq.neg$a5=substr(dnaSeq.neg$rev.dna,5,5)
dnaSeq.neg$a6=substr(dnaSeq.neg$rev.dna,6,6)
dnaSeq.neg$a7=substr(dnaSeq.neg$rev.dna,7,7)
dnaSeq.neg$a8=substr(dnaSeq.neg$rev.dna,8,8)
dnaSeq.neg$a9=substr(dnaSeq.neg$rev.dna,9,9)
dnaSeq.neg$a10=substr(dnaSeq.neg$rev.dna,10,10)
dnaSeq.neg$a11=substr(dnaSeq.neg$rev.dna,11,11)
dnaSeq.neg$a12=substr(dnaSeq.neg$rev.dna,12,12)
dnaSeq.neg$a13=substr(dnaSeq.neg$rev.dna,13,13)
dnaSeq.neg$a14=substr(dnaSeq.neg$rev.dna,14,14)
dnaSeq.neg$a15=substr(dnaSeq.neg$rev.dna,15,15)
dnaSeq.neg$a16=substr(dnaSeq.neg$rev.dna,16,16)
dnaSeq.neg$a17=substr(dnaSeq.neg$rev.dna,17,17)
dnaSeq.neg$a18=substr(dnaSeq.neg$rev.dna,18,18)
dnaSeq.neg$a19=substr(dnaSeq.neg$rev.dna,19,19)
dnaSeq.neg$a20=substr(dnaSeq.neg$rev.dna,20,20)
dnaSeq.neg$a21=substr(dnaSeq.neg$rev.dna,21,21)
dnaSeq.neg$a22=substr(dnaSeq.neg$rev.dna,22,22)
dnaSeq.neg$a23=substr(dnaSeq.neg$rev.dna,23,23)
dnaSeq.neg$a24=substr(dnaSeq.neg$rev.dna,24,24)
dnaSeq.neg$a25=substr(dnaSeq.neg$rev.dna,25,25)
dnaSeq.neg$a26=substr(dnaSeq.neg$rev.dna,26,26)
dnaSeq.neg$a27=substr(dnaSeq.neg$rev.dna,27,27)
dnaSeq.neg$a28=substr(dnaSeq.neg$rev.dna,28,28)
dnaSeq.neg$a29=substr(dnaSeq.neg$rev.dna,29,29)

df2=rbind(dnaSeq.pos[,12:40],dnaSeq.neg[,13:41]) # 3093
df=matrix(0,nrow=4,ncol=29)
rownames(df)=c("A","C","G","T")
for (i in 1:29){
  print(i)
  df[names(table(df2[,i])),i]=as.numeric(table(df2[,i]))
}
```

```
## [1] 1
## [1] 2
## [1] 3
## [1] 4
## [1] 5
## [1] 6
## [1] 7
## [1] 8
## [1] 9
## [1] 10
## [1] 11
## [1] 12
## [1] 13
## [1] 14
## [1] 15
## [1] 16
## [1] 17
## [1] 18
## [1] 19
## [1] 20
## [1] 21
## [1] 22
## [1] 23
## [1] 24
## [1] 25
## [1] 26
## [1] 27
## [1] 28
## [1] 29
```

```
colnames(df)=colnames(df2)
print(df)
```

```
##    a1   a2   a3   a4   a5   a6   a7   a8   a9  a10  a11  a12  a13  a14
## A 949 1123 1101 1024  825  333  583  927  260   41 2498  112  462 2756
## C 913  781  624  538  592  870  456  242 2499 3043   62 1907 1210   43
## G 618  626  645  527  637  296 1211 1374  149    0  257 1007  217  167
## T 613  563  723 1004 1039 1594  843  550  185    9  276   67 1204  127
##    a15  a16  a17  a18  a19  a20  a21  a22  a23  a24  a25  a26  a27 a28
## A   32 1488  134   62   77  372 1468  323  439 1336  599 1135  713 753
## C    0   13   42    2   54 2443   51 1450 1086  758  528  510  418 916
## G 3049 1575 1440 3014 2757   67 1490 1161  286  806  818  485 1384 769
## T   12   17 1477   15  205  211   84  159 1282  193 1148  963  578 655
##    a29
## A  851
## C 1039
## G  577
## T  626
```

```
#    a1   a2   a3   a4   a5   a6   a7   a8   a9  a10  a11  a12  a13  a14  a15  a16  a17  a18  a19  a20  a21  a22  a23  a24
# A 949 1123 1101 1024  825  333  583  927  260   41 2498  112  462 2756   32 1488  134   62   77  372 1468  323  439 1336
# C 913  781  624  538  592  870  456  242 2499 3043   62 1907 1210   43    0   13   42    2   54 2443   51 1450 1086  758
# G 618  626  645  527  637  296 1211 1374  149    0  257 1007  217  167 3049 1575 1440 3014 2757   67 1490 1161  286  806
# T 613  563  723 1004 1039 1594  843  550  185    9  276   67 1204  127   12   17 1477   15  205  211   84  159 1282  193
#    a25  a26  a27 a28  a29
# A  599 1135  713 753  851
# C  528  510  418 916 1039
# G  818  485 1384 769  577
# T 1148  963  578 655  626
pwm=makePWM(as.matrix(df/unique(colSums(df))),alphabet="DNA")
seqLogo(pwm,ic.scale=TRUE,xaxis=TRUE,yaxis=TRUE,xfontsize=15,yfontsize=15)
```

Alternate Alignment

```
dnaSeq.mut.pos=dnaSeq.pos
dnaSeq.mut.pos$alt=as.character(dnaSeq.mut.pos$alt)
dnaSeq.mut.pos$num=dnaSeq.mut.pos$mut-dnaSeq.mut.pos$start+1
for (i in 1:nrow(dnaSeq.mut.pos)){
  dnaSeq.mut.pos[i,paste("a",dnaSeq.mut.pos[i,"num"],sep="")]<-dnaSeq.mut.pos[i,"alt"]
}

dnaSeq.mut.neg=dnaSeq.neg
dnaSeq.mut.neg$alt=as.character(dnaSeq.mut.neg$alt)
dnaSeq.mut.neg$num=dnaSeq.mut.neg$end-dnaSeq.mut.neg$mut+1
for (i in 1:nrow(dnaSeq.mut.neg)){
  dnaSeq.mut.neg[i,paste("a",dnaSeq.mut.neg[i,"num"],sep="")]<-as.character(reverseComplement(DNAString(dnaSeq.mut.neg[i,"alt"])))
}

df7=rbind(dnaSeq.mut.pos[,12:40],dnaSeq.mut.neg[,13:41]) # 3093
df=matrix(0,nrow=4,ncol=29)
rownames(df)=c("A","C","G","T")
for (i in 1:29){
  print(i)
  df[names(table(df7[,i])),i]=as.numeric(table(df7[,i]))
}
```

```
## [1] 1
## [1] 2
## [1] 3
## [1] 4
## [1] 5
## [1] 6
## [1] 7
## [1] 8
## [1] 9
## [1] 10
## [1] 11
## [1] 12
## [1] 13
## [1] 14
## [1] 15
## [1] 16
## [1] 17
## [1] 18
## [1] 19
## [1] 20
## [1] 21
## [1] 22
## [1] 23
## [1] 24
## [1] 25
## [1] 26
## [1] 27
## [1] 28
## [1] 29
```

```
colnames(df)=colnames(df7)
print(df)
```

```
##    a1  a2  a3  a4  a5   a6   a7   a8   a9  a10  a11  a12  a13  a14  a15
## A 917 973 992 921 792  353  597  915  271   49 2473  117  464 2509   46
## C 932 881 698 617 641  904  461  263 2469 2999   74 1905 1213  111   11
## G 621 657 658 587 759  358 1238 1377  161    9  281  995  226  310 3015
## T 623 582 745 968 901 1478  797  538  192   36  265   76 1190  163   21
##    a16  a17  a18  a19  a20  a21  a22  a23  a24  a25  a26  a27 a28  a29
## A 1450  148   88  117  356 1418  306  433 1256  578 1076  643 723  846
## C   32   67    6   68 2402   75 1445 1090  798  560  534  469 925 1043
## G 1579 1406 2971 2710   79 1518 1183  298  847  840  509 1391 778  575
## T   32 1472   28  198  256   82  159 1272  192 1115  974  590 667  629
```

```
#    a1  a2  a3  a4  a5   a6   a7   a8   a9  a10  a11  a12  a13  a14  a15  a16  a17  a18  a19  a20  a21  a22  a23  a24  a25
# A 917 973 992 921 792  353  597  915  271   49 2473  117  464 2509   46 1450  148   88  117  356 1418  306  433 1256  578
# C 932 881 698 617 641  904  461  263 2469 2999   74 1905 1213  111   11   32   67    6   68 2402   75 1445 1090  798  560
# G 621 657 658 587 759  358 1238 1377  161    9  281  995  226  310 3015 1579 1406 2971 2710   79 1518 1183  298  847  840
# T 623 582 745 968 901 1478  797  538  192   36  265   76 1190  163   21   32 1472   28  198  256   82  159 1272  192 1115
#    a26  a27 a28  a29
# A 1076  643 723  846
# C  534  469 925 1043
# G  509 1391 778  575
# T  974  590 667  629
pwm=makePWM(as.matrix(df/unique(colSums(df))),alphabet="DNA")
seqLogo(pwm,ic.scale=TRUE,xaxis=TRUE,yaxis=TRUE,xfontsize=15,yfontsize=15)
```

```
df=rbind(dnaSeq.mut.pos[,c("ref","alt","num")],dnaSeq.mut.neg[,c("ref","alt","num")]) # 3093
df$count=1
df=aggregate(count~ref+alt+num,df,sum) # 317

for (i in 1:nrow(df)){
  if (!df$ref[i] %in% c("C","T")){
    df$alt[i]=as.character(reverseComplement(DNAString(df$alt[i])))
    df$ref[i]=as.character(reverseComplement(DNAString(df$ref[i])))
  }
}
df$mut=paste(df$ref,df$alt,sep="")
df$mut=factor(df$mut,levels=c("TG","TC","TA","CT","CG","CA"))
df$num=factor(df$num,levels=c(1:29))

df=aggregate(count~ref+alt+num+mut,df,sum) # 169
ggplot(df,aes(x=num,y=count,fill=mut))+
  geom_bar(stat="identity",colour="black")+
  theme(panel.grid.major = element_blank(),
        panel.grid.minor = element_blank(),
        panel.background = element_blank(),
        axis.line = element_line(colour="black"))+
  scale_fill_manual(values = c("#33CC00","#33CCFF","#FF9933","#CC33FF","#FFFF33","#FF0000"))
```

## Figure F part 3

Hotspot Mutations Reference Alignment

```
hotspot <- read.delim("LRmodel_hotspot_nonMSI_prefiltered-5_corrected.tsv", stringsAsFactors=FALSE)
hotspot$mut_region=rownames(hotspot)
hotspot=GRanges(seqnames=hotspot$chrom,IRanges(start=hotspot$start,end=hotspot$end),mut_region=hotspot$mut_region,pval=hotspot$pval,fdr=hotspot$fdr)
hotspot=hotspot[which(hotspot$pval<(0.01/2533374732))]
hotspot=reduce(hotspot)
hotspot$hotspot=c(1:length(hotspot))

motif=GRanges(seqnames=motif.ovl$seqnames,IRanges(start=motif.ovl$start,end=motif.ovl$end),pval=motif.ovl$pval,qval=motif.ovl$qval,dir=motif.ovl$dir,motif=motif.ovl$motif)
motif=motif+5

# some other mutations there but not in the hotspots (eg. 4 extra mutations at CBS +-5bp but not in the hotspot)
z=findOverlaps(maf.gastric,hotspot)
maf=maf.gastric[queryHits(z)]
maf$hotspot=hotspot[subjectHits(z)]$hotspot
length(unique(maf$id))
```

```
## [1] 260
```

```
z=findOverlaps(maf,motif) # 109
maf=maf[queryHits(z)]
maf=as.data.frame(maf)
maf$motif.start=start(motif[subjectHits(z)])
maf$motif.end=end(motif[subjectHits(z)])
maf$pval=motif[subjectHits(z)]$pval
maf$dir=motif[subjectHits(z)]$dir
maf$motif=motif[subjectHits(z)]$motif
length(unique(maf$id)) # 89
```

```
## [1] 89
```

```
maf=maf[order(maf$id,maf$pval),]
maf=maf[!duplicated(maf$id),] # 89
maf.pos=maf[which(maf$dir=="+"),] # 46
maf.neg=maf[which(maf$dir=="-"),] # 43

maf.ref.seq.pos=GRanges(seqnames=maf.pos$seqnames,
                    IRanges(maf.pos$motif.start,maf.pos$motif.end),
                    dir=maf.pos$dir,
                    motif=maf.pos$motif,
                    mut=maf.pos$start,
                    alt=maf.pos$tal,
                    ref=maf.pos$ral)
extrSeq=Views(Hsapiens,maf.ref.seq.pos)

z=as(extrSeq,"DNAStringSet") 
dnaSeq.pos.maf=as.data.frame(maf.ref.seq.pos)
dnaSeq.pos.maf$dna=as.character(z) 

dnaSeq.pos.maf$motif=toupper(dnaSeq.pos.maf$motif)
sum(dnaSeq.pos.maf$motif==dnaSeq.pos.maf$dna)
```

```
## [1] 0
```

```
dnaSeq.pos.maf$a1=substr(dnaSeq.pos.maf$dna,1,1)
dnaSeq.pos.maf$a2=substr(dnaSeq.pos.maf$dna,2,2)
dnaSeq.pos.maf$a3=substr(dnaSeq.pos.maf$dna,3,3)
dnaSeq.pos.maf$a4=substr(dnaSeq.pos.maf$dna,4,4)
dnaSeq.pos.maf$a5=substr(dnaSeq.pos.maf$dna,5,5)
dnaSeq.pos.maf$a6=substr(dnaSeq.pos.maf$dna,6,6)
dnaSeq.pos.maf$a7=substr(dnaSeq.pos.maf$dna,7,7)
dnaSeq.pos.maf$a8=substr(dnaSeq.pos.maf$dna,8,8)
dnaSeq.pos.maf$a9=substr(dnaSeq.pos.maf$dna,9,9)
dnaSeq.pos.maf$a10=substr(dnaSeq.pos.maf$dna,10,10)
dnaSeq.pos.maf$a11=substr(dnaSeq.pos.maf$dna,11,11)
dnaSeq.pos.maf$a12=substr(dnaSeq.pos.maf$dna,12,12)
dnaSeq.pos.maf$a13=substr(dnaSeq.pos.maf$dna,13,13)
dnaSeq.pos.maf$a14=substr(dnaSeq.pos.maf$dna,14,14)
dnaSeq.pos.maf$a15=substr(dnaSeq.pos.maf$dna,15,15)
dnaSeq.pos.maf$a16=substr(dnaSeq.pos.maf$dna,16,16)
dnaSeq.pos.maf$a17=substr(dnaSeq.pos.maf$dna,17,17)
dnaSeq.pos.maf$a18=substr(dnaSeq.pos.maf$dna,18,18)
dnaSeq.pos.maf$a19=substr(dnaSeq.pos.maf$dna,19,19)
dnaSeq.pos.maf$a20=substr(dnaSeq.pos.maf$dna,20,20)
dnaSeq.pos.maf$a21=substr(dnaSeq.pos.maf$dna,21,21)
dnaSeq.pos.maf$a22=substr(dnaSeq.pos.maf$dna,22,22)
dnaSeq.pos.maf$a23=substr(dnaSeq.pos.maf$dna,23,23)
dnaSeq.pos.maf$a24=substr(dnaSeq.pos.maf$dna,24,24)
dnaSeq.pos.maf$a25=substr(dnaSeq.pos.maf$dna,25,25)
dnaSeq.pos.maf$a26=substr(dnaSeq.pos.maf$dna,26,26)
dnaSeq.pos.maf$a27=substr(dnaSeq.pos.maf$dna,27,27)
dnaSeq.pos.maf$a28=substr(dnaSeq.pos.maf$dna,28,28)
dnaSeq.pos.maf$a29=substr(dnaSeq.pos.maf$dna,29,29)

maf.ref.seq.neg=GRanges(seqnames=maf.neg$seqnames,
                    IRanges(maf.neg$motif.start,maf.neg$motif.end),
                    dir=maf.neg$dir,
                    motif=maf.neg$motif,
                    mut=maf.neg$start,
                    alt=maf.neg$tal,
                    ref=maf.neg$ral)
extrSeq=Views(Hsapiens,maf.ref.seq.neg)

z=as(extrSeq,"DNAStringSet") 
dnaSeq.neg.maf=as.data.frame(maf.ref.seq.neg)
dnaSeq.neg.maf$dna=as.character(z) # 43

dnaSeq.neg.maf$motif=toupper(dnaSeq.neg.maf$motif)
sum(dnaSeq.neg.maf$motif==dnaSeq.neg.maf$dna)
```

```
## [1] 0
```

```
z=reverseComplement(z)
dnaSeq.neg.maf$rev.dna=as.character(z)
sum(dnaSeq.neg.maf$motif==dnaSeq.neg.maf$rev.dna)
```

```
## [1] 0
```

```
dnaSeq.neg.maf$a1=substr(dnaSeq.neg.maf$rev.dna,1,1)
dnaSeq.neg.maf$a2=substr(dnaSeq.neg.maf$rev.dna,2,2)
dnaSeq.neg.maf$a3=substr(dnaSeq.neg.maf$rev.dna,3,3)
dnaSeq.neg.maf$a4=substr(dnaSeq.neg.maf$rev.dna,4,4)
dnaSeq.neg.maf$a5=substr(dnaSeq.neg.maf$rev.dna,5,5)
dnaSeq.neg.maf$a6=substr(dnaSeq.neg.maf$rev.dna,6,6)
dnaSeq.neg.maf$a7=substr(dnaSeq.neg.maf$rev.dna,7,7)
dnaSeq.neg.maf$a8=substr(dnaSeq.neg.maf$rev.dna,8,8)
dnaSeq.neg.maf$a9=substr(dnaSeq.neg.maf$rev.dna,9,9)
dnaSeq.neg.maf$a10=substr(dnaSeq.neg.maf$rev.dna,10,10)
dnaSeq.neg.maf$a11=substr(dnaSeq.neg.maf$rev.dna,11,11)
dnaSeq.neg.maf$a12=substr(dnaSeq.neg.maf$rev.dna,12,12)
dnaSeq.neg.maf$a13=substr(dnaSeq.neg.maf$rev.dna,13,13)
dnaSeq.neg.maf$a14=substr(dnaSeq.neg.maf$rev.dna,14,14)
dnaSeq.neg.maf$a15=substr(dnaSeq.neg.maf$rev.dna,15,15)
dnaSeq.neg.maf$a16=substr(dnaSeq.neg.maf$rev.dna,16,16)
dnaSeq.neg.maf$a17=substr(dnaSeq.neg.maf$rev.dna,17,17)
dnaSeq.neg.maf$a18=substr(dnaSeq.neg.maf$rev.dna,18,18)
dnaSeq.neg.maf$a19=substr(dnaSeq.neg.maf$rev.dna,19,19)
dnaSeq.neg.maf$a20=substr(dnaSeq.neg.maf$rev.dna,20,20)
dnaSeq.neg.maf$a21=substr(dnaSeq.neg.maf$rev.dna,21,21)
dnaSeq.neg.maf$a22=substr(dnaSeq.neg.maf$rev.dna,22,22)
dnaSeq.neg.maf$a23=substr(dnaSeq.neg.maf$rev.dna,23,23)
dnaSeq.neg.maf$a24=substr(dnaSeq.neg.maf$rev.dna,24,24)
dnaSeq.neg.maf$a25=substr(dnaSeq.neg.maf$rev.dna,25,25)
dnaSeq.neg.maf$a26=substr(dnaSeq.neg.maf$rev.dna,26,26)
dnaSeq.neg.maf$a27=substr(dnaSeq.neg.maf$rev.dna,27,27)
dnaSeq.neg.maf$a28=substr(dnaSeq.neg.maf$rev.dna,28,28)
dnaSeq.neg.maf$a29=substr(dnaSeq.neg.maf$rev.dna,29,29)

df12=rbind(dnaSeq.pos.maf[,12:40],dnaSeq.neg.maf[,13:41]) # 89
df=matrix(0,nrow=4,ncol=29)
rownames(df)=c("A","C","G","T")
for (i in 1:29){
  print(i)
  df[names(table(df12[,i])),i]=as.numeric(table(df12[,i]))
}
```

```
## [1] 1
## [1] 2
## [1] 3
## [1] 4
## [1] 5
## [1] 6
## [1] 7
## [1] 8
## [1] 9
## [1] 10
## [1] 11
## [1] 12
## [1] 13
## [1] 14
## [1] 15
## [1] 16
## [1] 17
## [1] 18
## [1] 19
## [1] 20
## [1] 21
## [1] 22
## [1] 23
## [1] 24
## [1] 25
## [1] 26
## [1] 27
## [1] 28
## [1] 29
```

```
colnames(df)=colnames(df12)
print(df)
```

```
##   a1 a2 a3 a4 a5 a6 a7 a8 a9 a10 a11 a12 a13 a14 a15 a16 a17 a18 a19 a20
## A 44 65 64 50 42 20  9 29  0   0  89   0   9  81   0  60   0   0   0   0
## C 23  8  0  9  0 22 17  0 89  89   0  74  13   0   0   0   0   0   0  59
## G  0  9  9  6  0  0 20 46  0   0   0   9   0   0  89  29  42  89  81  14
## T 22  7 16 24 47 47 43 14  0   0   0   6  67   8   0   0  47   0   8  16
##   a21 a22 a23 a24 a25 a26 a27 a28 a29
## A  43  23  14  36  20  51   0   9  52
## C   0  38  22  53   0   7   8  37   7
## G  46  28   7   0  32  13  68   7   0
## T   0   0  46   0  37  18  13  36  30
```

```
#   a1 a2 a3 a4 a5 a6 a7 a8 a9 a10 a11 a12 a13 a14 a15 a16 a17 a18 a19 a20 a21 a22 a23 a24 a25 a26 a27 a28 a29
# A 44 65 64 50 42 20  9 29  0   0  89   0   9  81   0  60   0   0   0   0  43  23  14  36  20  51   0   9  52
# C 23  8  0  9  0 22 17  0 89  89   0  74  13   0   0   0   0   0   0  59   0  38  22  53   0   7   8  37   7
# G  0  9  9  6  0  0 20 46  0   0   0   9   0   0  89  29  42  89  81  14  46  28   7   0  32  13  68   7   0
# T 22  7 16 24 47 47 43 14  0   0   0   6  67   8   0   0  47   0   8  16   0   0  46   0  37  18  13  36  30
pwm=makePWM(as.matrix(df/unique(colSums(df))),alphabet="DNA")
seqLogo(pwm,ic.scale=TRUE,xaxis=TRUE,yaxis=TRUE,xfontsize=15,yfontsize=15)
```

Alternate Alignment

```
dnaSeq.mut.pos.maf=dnaSeq.pos.maf
dnaSeq.mut.pos.maf$alt=as.character(dnaSeq.mut.pos.maf$alt)
dnaSeq.mut.pos.maf$num=dnaSeq.mut.pos.maf$mut-dnaSeq.mut.pos.maf$start+1
for (i in 1:nrow(dnaSeq.mut.pos.maf)){
  dnaSeq.mut.pos.maf[i,paste("a",dnaSeq.mut.pos.maf[i,"num"],sep="")]<-dnaSeq.mut.pos.maf[i,"alt"]
}

dnaSeq.mut.neg.maf=dnaSeq.neg.maf
dnaSeq.mut.neg.maf$alt=as.character(dnaSeq.mut.neg.maf$alt)
dnaSeq.mut.neg.maf$num=dnaSeq.mut.neg.maf$end-dnaSeq.mut.neg.maf$mut+1
for (i in 1:nrow(dnaSeq.mut.neg.maf)){
  dnaSeq.mut.neg.maf[i,paste("a",dnaSeq.mut.neg.maf[i,"num"],sep="")]<-as.character(reverseComplement(DNAString(dnaSeq.mut.neg.maf[i,"alt"])))
}

df13=rbind(dnaSeq.mut.pos.maf[,12:40],dnaSeq.mut.neg.maf[,13:41]) # 89
df=matrix(0,nrow=4,ncol=29)
rownames(df)=c("A","C","G","T")
for (i in 1:29){
  print(i)
  df[names(table(df13[,i])),i]=as.numeric(table(df13[,i]))
}
```

```
## [1] 1
## [1] 2
## [1] 3
## [1] 4
## [1] 5
## [1] 6
## [1] 7
## [1] 8
## [1] 9
## [1] 10
## [1] 11
## [1] 12
## [1] 13
## [1] 14
## [1] 15
## [1] 16
## [1] 17
## [1] 18
## [1] 19
## [1] 20
## [1] 21
## [1] 22
## [1] 23
## [1] 24
## [1] 25
## [1] 26
## [1] 27
## [1] 28
## [1] 29
```

```
colnames(df)=colnames(df13)
print(df)
```

```
##   a1 a2 a3 a4 a5 a6 a7 a8 a9 a10 a11 a12 a13 a14 a15 a16 a17 a18 a19 a20
## A 43 53 46 41 40 20  9 26  0   0  89   0   9  73   1  56   0   0   0   0
## C 23 17 13 15  3 22 19  1 89  89   0  74  16   3   0   2   2   0   0  59
## G  0 11 12 10  5  2 23 47  0   0   0   9   2   4  88  30  42  89  81  14
## T 23  8 18 23 41 45 38 15  0   0   0   6  62   9   0   1  45   0   8  16
##   a21 a22 a23 a24 a25 a26 a27 a28 a29
## A  43  18  14  34  20  51   0   9  52
## C   0  40  22  53   0   7   8  37   7
## G  46  30   8   2  32  13  68   7   0
## T   0   1  45   0  37  18  13  36  30
```

```
#   a1 a2 a3 a4 a5 a6 a7 a8 a9 a10 a11 a12 a13 a14 a15 a16 a17 a18 a19 a20 a21 a22 a23 a24 a25 a26 a27 a28 a29
# A 43 53 46 41 40 20  9 26  0   0  89   0   9  73   1  56   0   0   0   0  43  18  14  34  20  51   0   9  52
# C 23 17 13 15  3 22 19  1 89  89   0  74  16   3   0   2   2   0   0  59   0  40  22  53   0   7   8  37   7
# G  0 11 12 10  5  2 23 47  0   0   0   9   2   4  88  30  42  89  81  14  46  30   8   2  32  13  68   7   0
# T 23  8 18 23 41 45 38 15  0   0   0   6  62   9   0   1  45   0   8  16   0   1  45   0  37  18  13  36  30
pwm=makePWM(as.matrix(df/colSums(df)),alphabet="DNA")
seqLogo(pwm,ic.scale=TRUE,xaxis=TRUE,yaxis=TRUE,xfontsize=15,yfontsize=15)
```

```
df=rbind(dnaSeq.mut.pos.maf[,c("ref","alt","num")],dnaSeq.mut.neg.maf[,c("ref","alt","num")]) # 89
df$count=1
df=aggregate(count~ref+alt+num,df,sum) # 46

for (i in 1:nrow(df)){
  if (!df$ref[i] %in% c("C","T")){
    df$alt[i]=as.character(reverseComplement(DNAString(df$alt[i])))
    df$ref[i]=as.character(reverseComplement(DNAString(df$ref[i])))
  }
}
df$mut=paste(df$ref,df$alt,sep="")
df$mut=factor(df$mut,levels=c("TG","TC","TA","CT","CG","CA"))
df$num=factor(df$num,levels=c(1:29))
df=aggregate(count~ref+alt+num+mut,df,sum) # 33

dff=data.frame(sub=0,num=c(1:29))
df=merge(df,dff,by="num",all.y=TRUE)
df$sub=ifelse(is.na(df$count),0,df$count)

ggplot(df,aes(x=num,y=sub,fill=mut))+
  geom_bar(stat="identity",colour="black")+
  theme(panel.grid.major = element_blank(),
        panel.grid.minor = element_blank(),
        panel.background = element_blank(),
        axis.line = element_line(colour="black"))+
  scale_fill_manual(values = c("#33CC00","#33CCFF","#FF9933","#CC33FF","#FFFF33","#FF0000"))
```

## Figure F part 1

Jasper CTCF motif Also for Figure 5B,E,H

```
pfm <- read.table("ctcf_pfm.txt", quote="\"", comment.char="", stringsAsFactors=FALSE) # downloaded from jasper website
colnames(pfm)=colnames(df12)[1:19]
pwm=makePWM(as.matrix(pfm/colSums(pfm)),alphabet="DNA")
seqLogo(pwm,ic.scale=TRUE,xaxis=TRUE,yaxis=TRUE,xfontsize=15,yfontsize=15)
```

```
seqLogo(pwm,ic.scale=FALSE,xaxis=TRUE,yaxis=TRUE,xfontsize=15,yfontsize=15)
```

```
# reverse complement for jaspar motif, obtained from jasper website
a=c(59,396,37,17,67,71,8,341,9,3,18,324,11,91,2,36,134,187,459)
c=c(266,73,307,507,5,775,890,504,566,903,32,48,334,65,0,21,449,414,76)
g=c(181,322,482,13,733,8,0,12,3,0,11,433,528,13,903,800,49,145,291)
t=c(402,117,82,372,104,56,12,54,333,5,851,107,40,744,8,56,281,167,87)
pfm=rbind(a,c,g,t)
pwm=makePWM(as.matrix(pfm/colSums(pfm)),alphabet="DNA")
seqLogo(pwm,ic.scale=FALSE,xaxis=TRUE,yaxis=TRUE,xfontsize=15,yfontsize=15)
```
