# Supplementary material for: Mutation hotspots at CTCF binding sites coupled to chromosomal instability in gastrointestinal cancers
Source: Nat Commun. 2018 Apr 18;9:1520. doi: 10.1038/s41467-018-03828-2 (PMC5906695; doi:10.1038/s41467-018-03828-2)
Supplement: Supplementary file 8 — Supplementary Data 5 [file 41467_2018_3828_MOESM8_ESM.zip › Rmarkdowns/Figure 5/Figure5_SNV_candidates_rev.html]

Figure 5 - SNV candidates


# Figure 5 - SNV candidates

This is the R Markdown for Figure 5, which consists of 9 parts.

## Figure C,F,I

Boxplots for gene expression in TCGA samples

```
## Overlap each hotspot with maf.gastric (prefiltered, both MSI and nonMSI) to identify
## mutated samples in hotspots
maf.gastric <- maf.to.granges('gastric_RF_prefiltered.MAF')
```

```
## [1] ">> Reading compact MAF ..."
```

```
maf.gastric=maf.gastric[-which(maf.gastric$sid %in% c("tan2001206", "tan20021007", "tan980319", "tan2000986", "tan980436"))] # 4119812, 187 unique sids

hotspot <- read.delim("LRmodel_hotspot_nonMSI_prefiltered-5_corrected.tsv", stringsAsFactors=FALSE)
hotspot$mut_region=rownames(hotspot)
hotspot=GRanges(seqnames=hotspot$chrom,IRanges(start=hotspot$start,end=hotspot$end),mut_region=hotspot$mut_region,pval=hotspot$pval,fdr=hotspot$fdr) # 74
hotspot=hotspot[which(hotspot$pval<(0.01/2533374732))] #67
hotspot=reduce(hotspot) #34
hotspot$hotspot=c(1:length(hotspot))

## TAN expression data
rnaseq.tan=read.table("gene_fpkm.txt", header=T, sep="\t", check.names=F)
rnaseq.tan=rnaseq.tan[,-1]
rnaseq.tan=rnaseq.tan[,-which(colnames(rnaseq.tan) %in% c("T2001206", "T20021007", "T980319", "T2000986", "T980436",
                                                          "N2001206", "N20021007", "N980319", "N2000986", "N980436"))]

rnaseq.tan.boxplot <- function(gene, mut.samples, rnaseq.data) {
  gene.rnaseq=rnaseq.data[rnaseq.data[,1]==gene,]
  gene.rnaseq=(gene.rnaseq[-1])
  gene.df=data.frame(id=names(gene.rnaseq),exp=as.numeric(gene.rnaseq), group=c(rep("Normal",14),rep("Tumor_WT",14)),stringsAsFactors =F)
  gene.df$group[names(gene.rnaseq)%in%mut.samples]="Tumor_Mut"
  print(gene.df[gene.df$group=="Tumor_Mut",])
  print(paste("median of Tumor_Mut: ",median(gene.df$exp[gene.df$group=="Tumor_Mut"])))
  print(paste("median of Tumor_WT: ",median(gene.df$exp[gene.df$group=="Tumor_WT"])))
  print(paste("median of Normal: ",median(gene.df$exp[gene.df$group=="Normal"])))
  print(wilcox.test(gene.df$exp[gene.df$group=="Tumor_Mut"],gene.df$exp[gene.df$group=="Tumor_WT"]))
  print(wilcox.test(gene.df$exp[gene.df$group=="Tumor_WT"],gene.df$exp[gene.df$group=="Normal"]))
  if (max(gene.df$exp[gene.df$group=="Tumor_Mut"])<1&&max(gene.df$exp[gene.df$group=="Tumor_WT"])<1) {
    print(paste(gene," not expressed"))
    return()
  }  
  p=ggplot(gene.df, aes(x=factor(group, levels = c("Normal","Tumor_WT","Tumor_Mut")), y=exp))+ stat_boxplot(geom ='errorbar')+geom_boxplot(outlier.shape = NA)+
    geom_jitter(aes(colour=group), size=2, width=0.1)+ scale_color_brewer(palette="Paired")+ylab(paste(gene,"fpkm"))+xlab(NULL)+
    theme(text = element_text(size=20),axis.text.x = element_text(size=20))+theme(legend.position="none")+
    theme(panel.grid.major = element_blank(),
          panel.grid.minor = element_blank(),
          panel.background = element_blank(),
          axis.line = element_line(colour="black"))
  print(p)
}

## TCGA expression data
rnaseq.tcga=read.table("STAD.rnaseqv2_RSEM_genes_normalized.txt", header=T, sep="\t", check.names=F, stringsAsFactors = F)
rnaseq.tcga=rnaseq.tcga[-1,]
names(rnaseq.tcga)=substr(names(rnaseq.tcga),1,15)
rnaseq.gene.id=do.call(rbind,strsplit(rnaseq.tcga[,1],"[|]"))
rnaseq.tcga[,1]=rnaseq.gene.id[,1]
tcga.samples=read.table("TCGA_sample_ID.txt", header=F, sep="\t", colClasses=c('character', 'character'))
all.tcga.samples=names(rnaseq.tcga)
tcga.tissue=substr(all.tcga.samples,14,15)
unique(tcga.tissue) # [1] "01" "11"
```

```
## [1] " R" "01" "11"
```

```
sum(tcga.tissue=="11") # 35
```

```
## [1] 35
```

```
normal.samples=all.tcga.samples[tcga.tissue=="11"]
tumor.samples=all.tcga.samples[all.tcga.samples%in%substr(tcga.samples[,1],1,15)]
rnaseq.tcga=rnaseq.tcga[,c("Hybridization R",tumor.samples,normal.samples)]

rnaseq.tcga.boxplot <- function(gene, mut.samples, rnaseq.data) {
  gene.rnaseq=rnaseq.data[rnaseq.data[,1]==gene,]
  gene.rnaseq=(gene.rnaseq[-1])
  gene.df=data.frame(id=names(gene.rnaseq),exp=as.numeric(gene.rnaseq), group=c(rep("Tumor_WT",35),rep("Normal",35)),stringsAsFactors =F)
  gene.df$group[names(gene.rnaseq)%in%mut.samples]="Tumor_Mut"
  print(gene.df[gene.df$group=="Tumor_Mut",])
  print(paste("median of Tumor_Mut: ",median(gene.df$exp[gene.df$group=="Tumor_Mut"])))
  print(paste("median of Tumor_WT: ",median(gene.df$exp[gene.df$group=="Tumor_WT"])))
  print(paste("median of Normal: ",median(gene.df$exp[gene.df$group=="Normal"])))
  print(wilcox.test(gene.df$exp[gene.df$group=="Tumor_Mut"],gene.df$exp[gene.df$group=="Tumor_WT"]))
  print(wilcox.test(gene.df$exp[gene.df$group=="Tumor_WT"],gene.df$exp[gene.df$group=="Normal"]))
  if (max(gene.df$exp[gene.df$group=="Tumor_Mut"])<5&&max(gene.df$exp[gene.df$group=="Tumor_WT"])<5) {
    print(paste(gene," not expressed"))
    return()
  }  
  p=ggplot(gene.df, aes(x=factor(group, levels = c("Normal","Tumor_WT","Tumor_Mut")), y=exp))+ stat_boxplot(geom ='errorbar')+geom_boxplot(outlier.shape = NA)+
    geom_jitter(aes(colour=group), size=2,width=0.1)+ scale_color_brewer(palette="Paired")+ylab(paste(gene,"fpkm"))+xlab(NULL)+
    theme(text = element_text(size=20),axis.text.x = element_text(size=20))+theme(legend.position="none")+
    theme(panel.grid.major = element_blank(),
          panel.grid.minor = element_blank(),
          panel.background = element_blank(),
          axis.line = element_line(colour="black"))
  print(p)
}
```

## Figure C

CENPQ

```
z=findOverlaps(maf.gastric,hotspot[1])
maf=maf.gastric[queryHits(z)] # 14
sid=unique(maf$sid) # 12
print(sid)
```

```
##  [1] TCGA-CG-4442 apollo10     TCGA-D7-6527 TCGA-D7-6822 HK-pfg119   
##  [6] HK-pfg072    HK-pfg144    tan76629543  HK-pfg272    apollo17    
## [11] apollo11     HK-pfg069   
## 192 Levels: apollo1_new apollo10 apollo11 apollo12 apollo13 ... TCGA-IN-7806
```

```
CENPQ.mut=c("TCGA-CG-4442-01", "TCGA-D7-6527-01","TCGA-D7-6822-01")
rnaseq.tcga.boxplot("CENPQ", CENPQ.mut, rnaseq.tcga)
```

```
##                 id      exp     group
## 15 TCGA-CG-4442-01 213.9701 Tumor_Mut
## 22 TCGA-D7-6527-01 143.7295 Tumor_Mut
## 25 TCGA-D7-6822-01 153.8845 Tumor_Mut
## [1] "median of Tumor_Mut:  153.8845"
## [1] "median of Tumor_WT:  81.44635"
## [1] "median of Normal:  59.6026"
## 
##  Wilcoxon rank sum test
## 
## data:  gene.df$exp[gene.df$group == "Tumor_Mut"] and gene.df$exp[gene.df$group == "Tumor_WT"]
## W = 90, p-value = 0.007028
## alternative hypothesis: true location shift is not equal to 0
## 
## 
##  Wilcoxon rank sum test
## 
## data:  gene.df$exp[gene.df$group == "Tumor_WT"] and gene.df$exp[gene.df$group == "Normal"]
## W = 795, p-value = 0.002843
## alternative hypothesis: true location shift is not equal to 0
```

## Figure F

KCNQ5

```
z=findOverlaps(maf.gastric,hotspot[2])
maf=maf.gastric[queryHits(z)] # 9
sid=unique(maf$sid) # 9
print(sid)
```

```
## [1] HK-pfg272         HK-pfg146         TCGA-BR-6452      TCGA-D7-6527     
## [5] TCGA-BR-4280      TCGA-D7-6822      CGP_donor_GC00052 HK-pfg030        
## [9] tan2000639       
## 192 Levels: apollo1_new apollo10 apollo11 apollo12 apollo13 ... TCGA-IN-7806
```

```
KCNQ5.mut=c("TCGA-BR-6452-01", "TCGA-D7-6527-01","TCGA-D7-6822-01","TCGA-BR-4280-01")
rnaseq.tcga.boxplot("KCNQ5", KCNQ5.mut, rnaseq.tcga)
```

```
##                 id    exp     group
## 2  TCGA-BR-4280-01 0.9970 Tumor_Mut
## 3  TCGA-BR-6452-01 1.5235 Tumor_Mut
## 22 TCGA-D7-6527-01 1.4091 Tumor_Mut
## 25 TCGA-D7-6822-01 1.6618 Tumor_Mut
## [1] "median of Tumor_Mut:  1.4663"
## [1] "median of Tumor_WT:  5.8134"
## [1] "median of Normal:  44.5613"
## 
##  Wilcoxon rank sum test
## 
## data:  gene.df$exp[gene.df$group == "Tumor_Mut"] and gene.df$exp[gene.df$group == "Tumor_WT"]
## W = 12, p-value = 0.005921
## alternative hypothesis: true location shift is not equal to 0
```

```
## Warning in wilcox.test.default(gene.df$exp[gene.df$group == "Tumor_WT"], :
## cannot compute exact p-value with ties
```

```
## 
##  Wilcoxon rank sum test with continuity correction
## 
## data:  gene.df$exp[gene.df$group == "Tumor_WT"] and gene.df$exp[gene.df$group == "Normal"]
## W = 144, p-value = 3.161e-07
## alternative hypothesis: true location shift is not equal to 0
```

## Figure I

SPG20

```
z=findOverlaps(maf.gastric,hotspot[30])
maf=maf.gastric[queryHits(z)] # 8
sid=unique(maf$sid) # 8
print(sid)
```

```
## [1] HK-pfg054         tan76629543       CGP_donor_GC00047 HK-pfg180        
## [5] TCGA-D7-6528      TCGA-D7-6822      HK-pfg034         TCGA-D7-6527     
## 192 Levels: apollo1_new apollo10 apollo11 apollo12 apollo13 ... TCGA-IN-7806
```

```
SPG20.mut=c("TCGA-D7-6528-01", "TCGA-D7-6527-01","TCGA-D7-6822-01")
rnaseq.tcga.boxplot("SPG20", SPG20.mut, rnaseq.tcga)
```

```
##                 id      exp     group
## 22 TCGA-D7-6527-01 207.6092 Tumor_Mut
## 23 TCGA-D7-6528-01  62.2996 Tumor_Mut
## 25 TCGA-D7-6822-01 142.9165 Tumor_Mut
## [1] "median of Tumor_Mut:  142.9165"
## [1] "median of Tumor_WT:  480.7931"
## [1] "median of Normal:  813.1966"
## 
##  Wilcoxon rank sum test
## 
## data:  gene.df$exp[gene.df$group == "Tumor_Mut"] and gene.df$exp[gene.df$group == "Tumor_WT"]
## W = 14, p-value = 0.04492
## alternative hypothesis: true location shift is not equal to 0
## 
## 
##  Wilcoxon rank sum test
## 
## data:  gene.df$exp[gene.df$group == "Tumor_WT"] and gene.df$exp[gene.df$group == "Normal"]
## W = 298, p-value = 0.0008196
## alternative hypothesis: true location shift is not equal to 0
```

Calculation for log2 fold change

```
rnaseq.tcga.fc <- function(gene, mut.samples, rnaseq.data) {
  gene.rnaseq=rnaseq.data[rnaseq.data[,1]==gene,]
  gene.rnaseq=(gene.rnaseq[-1])
  gene.df=data.frame(id=names(gene.rnaseq),exp=as.numeric(gene.rnaseq), group=c(rep("Tumor_WT",35),rep("Normal",35)),stringsAsFactors =F)
  gene.df$group[names(gene.rnaseq)%in%mut.samples]="Tumor_Mut"
  print(gene.df[gene.df$group=="Tumor_Mut",])
  print(paste("median of Tumor_Mut: ",median(gene.df$exp[gene.df$group=="Tumor_Mut"])))
  print(paste("median of Tumor_WT: ",median(gene.df$exp[gene.df$group=="Tumor_WT"])))
  print(paste("median of Normal: ",median(gene.df$exp[gene.df$group=="Normal"])))
  print(wilcox.test(gene.df$exp[gene.df$group=="Tumor_Mut"],gene.df$exp[gene.df$group=="Tumor_WT"]))
  print(wilcox.test(gene.df$exp[gene.df$group=="Tumor_WT"],gene.df$exp[gene.df$group=="Normal"]))
  print(quantile(gene.df$exp[gene.df$group=="Tumor_WT"]))
  if (quantile(gene.df$exp[gene.df$group=="Tumor_WT"])[4]>10 | sum(gene.df$exp[gene.df$group=="Tumor_Mut"]>10)>=1) {
    print(paste(gene," expressed"))
  }  else {
    print(paste(gene, " not expressed"))
  }
  log2fc=log2(median(gene.df$exp[gene.df$group=="Tumor_Mut"])/median(gene.df$exp[gene.df$group=="Tumor_WT"]))
  print(log2fc)
  return(log2fc)
}

CENPQ.1=rnaseq.tcga.fc("CENPQ", CENPQ.mut, rnaseq.tcga)
```

```
##                 id      exp     group
## 15 TCGA-CG-4442-01 213.9701 Tumor_Mut
## 22 TCGA-D7-6527-01 143.7295 Tumor_Mut
## 25 TCGA-D7-6822-01 153.8845 Tumor_Mut
## [1] "median of Tumor_Mut:  153.8845"
## [1] "median of Tumor_WT:  81.44635"
## [1] "median of Normal:  59.6026"
## 
##  Wilcoxon rank sum test
## 
## data:  gene.df$exp[gene.df$group == "Tumor_Mut"] and gene.df$exp[gene.df$group == "Tumor_WT"]
## W = 90, p-value = 0.007028
## alternative hypothesis: true location shift is not equal to 0
## 
## 
##  Wilcoxon rank sum test
## 
## data:  gene.df$exp[gene.df$group == "Tumor_WT"] and gene.df$exp[gene.df$group == "Normal"]
## W = 795, p-value = 0.002843
## alternative hypothesis: true location shift is not equal to 0
## 
##        0%       25%       50%       75%      100% 
##  44.78980  64.15203  81.44635 123.22825 185.64530 
## [1] "CENPQ  expressed"
## [1] 0.917926
```

```
KCNQ5.1=rnaseq.tcga.fc("KCNQ5", KCNQ5.mut, rnaseq.tcga)
```

```
##                 id    exp     group
## 2  TCGA-BR-4280-01 0.9970 Tumor_Mut
## 3  TCGA-BR-6452-01 1.5235 Tumor_Mut
## 22 TCGA-D7-6527-01 1.4091 Tumor_Mut
## 25 TCGA-D7-6822-01 1.6618 Tumor_Mut
## [1] "median of Tumor_Mut:  1.4663"
## [1] "median of Tumor_WT:  5.8134"
## [1] "median of Normal:  44.5613"
## 
##  Wilcoxon rank sum test
## 
## data:  gene.df$exp[gene.df$group == "Tumor_Mut"] and gene.df$exp[gene.df$group == "Tumor_WT"]
## W = 12, p-value = 0.005921
## alternative hypothesis: true location shift is not equal to 0
```

```
## Warning in wilcox.test.default(gene.df$exp[gene.df$group == "Tumor_WT"], :
## cannot compute exact p-value with ties
```

```
## 
##  Wilcoxon rank sum test with continuity correction
## 
## data:  gene.df$exp[gene.df$group == "Tumor_WT"] and gene.df$exp[gene.df$group == "Normal"]
## W = 144, p-value = 3.161e-07
## alternative hypothesis: true location shift is not equal to 0
## 
##       0%      25%      50%      75%     100% 
##  0.48400  2.66950  5.81340 12.32005 28.80110 
## [1] "KCNQ5  expressed"
## [1] -1.987202
```

```
SPG20.1=rnaseq.tcga.fc("SPG20", SPG20.mut, rnaseq.tcga)
```

```
##                 id      exp     group
## 22 TCGA-D7-6527-01 207.6092 Tumor_Mut
## 23 TCGA-D7-6528-01  62.2996 Tumor_Mut
## 25 TCGA-D7-6822-01 142.9165 Tumor_Mut
## [1] "median of Tumor_Mut:  142.9165"
## [1] "median of Tumor_WT:  480.7931"
## [1] "median of Normal:  813.1966"
## 
##  Wilcoxon rank sum test
## 
## data:  gene.df$exp[gene.df$group == "Tumor_Mut"] and gene.df$exp[gene.df$group == "Tumor_WT"]
## W = 14, p-value = 0.04492
## alternative hypothesis: true location shift is not equal to 0
## 
## 
##  Wilcoxon rank sum test
## 
## data:  gene.df$exp[gene.df$group == "Tumor_WT"] and gene.df$exp[gene.df$group == "Normal"]
## W = 298, p-value = 0.0008196
## alternative hypothesis: true location shift is not equal to 0
## 
##        0%       25%       50%       75%      100% 
##   46.0609  175.6913  480.7931  715.5404 1384.7957 
## [1] "SPG20  expressed"
## [1] -1.750244
```

## Figure A,D,G

Expression of genes within TAD region around hotspot

```
chrOrder<-c(paste("chr",1:22,sep=""),"chrX")
seqi = seqinfo(Hsapiens)[seqnames(Hsapiens)[1:23]]
seqnames=seqnames(seqinfo(Hsapiens))[1:23]

rnaseq.tcga=read.table("STAD.rnaseqv2_RSEM_genes_normalized.txt", header=T, sep="\t", check.names=F, stringsAsFactors = F)
rnaseq.tcga=rnaseq.tcga[-1,]
names(rnaseq.tcga)=substr(names(rnaseq.tcga),1,15)
rnaseq.gene.id=do.call(rbind,strsplit(rnaseq.tcga[,1],"[|]"))
rnaseq.tcga[,1]=rnaseq.gene.id[,2]
tcga.samples=read.table("TCGA_sample_ID.txt", header=F, sep="\t", colClasses=c('character', 'character'))
all.tcga.samples=names(rnaseq.tcga)
tcga.tissue=substr(all.tcga.samples,14,15)
unique(tcga.tissue) # [1] "01" "11"
```

```
## [1] " R" "01" "11"
```

```
sum(tcga.tissue=="11") # 35
```

```
## [1] 35
```

```
normal.samples=all.tcga.samples[tcga.tissue=="11"]
tumor.samples=all.tcga.samples[all.tcga.samples%in%substr(tcga.samples[,1],1,15)]
rnaseq.tcga=rnaseq.tcga[,c("Hybridization R",tumor.samples,normal.samples)]

# Get mutations
maf <- maf.to.granges('gastric_RF_prefiltered.MAF')
```

```
## [1] ">> Reading compact MAF ..."
```

```
maf=maf[-which(maf$sid %in% c("tan2001206", "tan20021007", "tan980319", "tan2000986", "tan980436"))] # 4119812
maf=maf[-which(as.character(seqnames(maf))=="chrY")] # 4116299

# Gene regions
genes=read.delim("gene_mart_export_entrez.txt", header=T, stringsAsFactors = F)
genes=with(genes, GRanges(Chromosome.scaffold.name, IRanges(Gene.start..bp., Gene.end..bp.), id=Gene.name,entrez=EntrezGene.ID))
seqlevelsStyle(genes)<- "UCSC"
genes=genes[seqnames(genes) %in% seqnames(seqi)]
seqlevels(genes)=as.character(unique(seqnames(genes))) #24497

# get hotspots
lr.hotspot=read.delim("LRmodel_hotspot_nonMSI_prefiltered-5_corrected.tsv", header=T, stringsAsFactors = F) # 7513
lr.hotspot$mut_region=row.names(lr.hotspot)
hotspot.filtered=lr.hotspot[lr.hotspot$pval<(0.01/2533374732),] #67
hotspot.ranges=with(hotspot.filtered, GRanges(chrom, IRanges(start, end), id=hotspot.filtered$mut_region))
hotspot.ranges=reduce(hotspot.ranges) # 34 unique ranges
# top 3 candidates
hotspot.ranges=hotspot.ranges[c(1,2,30)]

# hotspots that overlap CTCF binding sites
roi.ctcf <- bed.to.granges("ctcf_motif_union.bed") # 47453
hotspot.ctcf=hotspot.ranges[unique(queryHits(findOverlaps(hotspot.ranges,roi.ctcf)))] #3
# extend each hotspot by 2Mbp on each side
hotspot.ctcf.ext=hotspot.ctcf+2*10^6

# hotspot.ranges.ext=>tad regions
hotspot.ranges.ext=GRanges(seqnames=c("chr6","chr6","chr13"),IRanges(start=c(47480000,71920000,35760000),end=c(52000000,74160000,38320000)))

## find genes in each hotspot, match genes by entrez ID
hotspot.genes=lapply(hotspot.ranges.ext, function(x) {unique(genes$entrez[subjectHits(findOverlaps(x,genes))])})

rnaseq.pval <- function(gene, mut.samples, rnaseq.data) {
  if(!gene%in%rnaseq.tcga[,1]){
    return(c(0,2,-1,-1,-1))
  }
  gene.rnaseq=rnaseq.data[rnaseq.data[,1]==gene,]
  gene.rnaseq=(gene.rnaseq[-1])
  gene.df=data.frame(id=names(gene.rnaseq),exp=as.numeric(gene.rnaseq), group=c(rep("Tumor_WT",35),rep("Normal",35)),stringsAsFactors =F)
  gene.df$group[names(gene.rnaseq)%in%mut.samples]="Tumor_Mut"
  median.norm=median(gene.df$exp[gene.df$group=="Normal"])
  median.wt=median(gene.df$exp[gene.df$group=="Tumor_WT"])
  median.mut=median(gene.df$exp[gene.df$group=="Tumor_Mut"])
  if (max(gene.df$exp[gene.df$group=="Tumor_Mut"])<10&&max(gene.df$exp[gene.df$group=="Tumor_WT"])<10) {
    return(c(0,2,median.mut,median.wt,median.norm))
  }
 
  if (median.wt !=0) {
    log2fc=log2(median.mut/median.wt)
  } else{
    log2fc=0
  }
  pval=wilcox.test(gene.df$exp[gene.df$group=="Tumor_Mut"],gene.df$exp[gene.df$group=="Tumor_WT"])$p.value
  if (median.mut<1&&median.wt<1) {
    return(c(0,pval,median.mut,median.wt,median.norm))
  }   
  return(c(log2fc, pval,median.mut,median.wt,median.norm))
}

# TCGA samples in the hotspots
hp.muts=as.list(numeric(length(hotspot.ranges)))
for (i in 1:length(hotspot.ranges)){
  z=findOverlaps(maf,hotspot.ranges[i])
  z=maf[queryHits(z)]
  z=unique(as.character(z$sid))
  z=z[grepl("TCGA",z)]
  if (length(z)!=0){
    z=paste(z,"-01",sep="")
  } else {
    z="Nil"
  }
  hp.muts[[i]]=z
}

results=lapply(1:length(hp.muts), function(i){
    print(i)
    if(hp.muts[[i]][1]!="Nil"){
      if (sum(hp.muts[[i]] %in% colnames(rnaseq.tcga))>=1){
        out=lapply(hotspot.genes[[i]], function(g){suppressWarnings(rnaseq.pval(g, hp.muts[[i]], rnaseq.tcga))})
        out.df=do.call(rbind, out)
        colnames(out.df)=c("log2fc","pval","tumor.mut","tumor.wt","norm")
        rownames(out.df)=hotspot.genes[[i]]    
        out.ordered=out.df[order(out.df[,"pval"]),]
      } else {
        out.ordered=NULL
      }
    } else {
      out.ordered=NULL
    }
})
```

```
## [1] 1
## [1] 2
## [1] 3
```

```
# distance from start/end of gene to hotspot (choose the nearest endpoint)
results2=lapply(1:length(hotspot.genes), function(i){
  print(i)
  out=lapply(hotspot.genes[[i]],function(g) {
    if (! g %in% genes$entrez){
      print("error")
    } else if (unique(as.character(seqnames(genes[which(genes$entrez==g)])))!=as.character(seqnames(hotspot.ranges[i]))){
      print("error")
    } else {
      g2=genes[which(genes$entrez==g)]
      hs=hotspot.ranges[i]
      case1=abs(start(hs)-end(g2))
      case2=abs(end(hs)-start(g2))
      case3=abs(start(hs)-start(g2))
      case4=abs(end(hs)-end(g2))
      cases=c(case1,case2,case3,case4)
      if (length(cases)==4){
        if (which(cases==min(cases)) %in% c(1,3)){
          data.frame(dist=min(cases),side="left")
        } else {
          data.frame(dist=min(cases),side="right")
        }
      } else {
        if (which(cases==min(cases)) %in% c(1,2,5,6)){
          data.frame(dist=min(cases),side="left")
        } else {
          data.frame(dist=min(cases),side="right")
        }
      }
    }
  })
  out.df=do.call(rbind, out)
  rownames(out.df)=hotspot.genes[[i]]    
  out.df
})
```

```
## [1] 1
## [1] 2
## [1] 3
```

```
hs=which(results!="NULL") # 3
for (i in hs){
  print(i)
  df=results[[i]]
  df=as.data.frame(df)
  df$log.pval=-log10(df$pval)
  df$log.pval=ifelse(df$log2fc<0,-df$log.pval,df$log.pval)
  df$log.pval=ifelse(df$pval==2,0,df$log.pval)
  df$dist=results2[[i]][rownames(df),"dist"]
  df$side=results2[[i]][rownames(df),"side"]
  df$expr=ifelse(df$pval==2,"not","expr")
  df$sig=ifelse(df$pval<=0.05,"sig","not")
  df$dist=ifelse(df$side=="left",-df$dist,df$dist)
  df=df[order(df$dist,decreasing=FALSE),]
  df$gene=rownames(df)
    print(ggplot(df,aes(x=dist,y=log.pval))+
            geom_point(aes(shape=factor(expr),col=factor(sig)))+
            theme(axis.text.x = element_text(angle = 90, hjust = 1))+
            scale_colour_manual(values=c("#999999","#FF0000"))+
            scale_shape_manual(values=c(16,1))+
            theme(panel.grid.major = element_blank(),
                  panel.grid.minor = element_blank(),
                  panel.background = element_blank(),
                  axis.line=element_line(colour="black"))+
            geom_hline(yintercept=0)+
            theme(legend.position="none")+
    geom_text(aes(x=dist,y=log.pval,label=gene),hjust=0, vjust=0)
    )
}
```

```
## [1] 1
```

```
## [1] 2
```

```
## [1] 3
```

```
# SPG20: repeated SOHLH2, keep 1
```
