# Supplementary material for: Mutation hotspots at CTCF binding sites coupled to chromosomal instability in gastrointestinal cancers
Source: Nat Commun. 2018 Apr 18;9:1520. doi: 10.1038/s41467-018-03828-2 (PMC5906695; doi:10.1038/s41467-018-03828-2)
Supplement: Supplementary file 8 — Supplementary Data 5 [file 41467_2018_3828_MOESM8_ESM.zip › Rmarkdowns/Figure 6/Figure6_pancancer_rev.html]

Figure 6 - Pancancer


# Figure 6 - Pancancer

This is the R Markdown for Figure 6, which consists of 3 parts.

## Figure A

Proportions of mutated samples that overlapped with CBS hotspots for each cancer type

```
hotspot <- read.delim("LRmodel_hotspot_nonMSI_prefiltered-5_corrected.tsv", stringsAsFactors=FALSE)
hotspot$mut_region=rownames(hotspot)
hotspot=GRanges(seqnames=hotspot$chrom,IRanges(start=hotspot$start,end=hotspot$end),mut_region=hotspot$mut_region,pval=hotspot$pval,fdr=hotspot$fdr)
hotspot=hotspot[which(hotspot$pval<(0.01/2533374732))]
hotspot=reduce(hotspot)
hotspot$hotspot=c(1:length(hotspot))

# Identify CTCF hotspot
roi.ctcf <- bed.to.granges("ctcf_motif_union.bed")
ctcf.hotspot=hotspot[unique(queryHits(findOverlaps(hotspot,roi.ctcf)))]
other.hotspot=hotspot[-unique(queryHits(findOverlaps(hotspot,roi.ctcf)))]

# read in maf file
maf.gastric <- maf.to.granges('gastric_RF_prefiltered.MAF')
```

```
## [1] ">> Reading compact MAF ..."
```

```
maf.gastric$ctype=as.character(maf.gastric$ctype)
maf.gastric$ctype="gastric"
seqi = seqinfo(Hsapiens)[intersect(seqnames(seqinfo(Hsapiens))[1:23],as.character(seqnames(maf.gastric)))]
maf.gastric=maf.gastric[which(seqnames(maf.gastric) %in% seqnames(seqi))] # 4139879
maf.gastric=maf.gastric[-which(maf.gastric$sid %in% c("tan2001206", "tan20021007", "tan980319", "tan2000986", "tan980436"))] # 4116299

maf.pancan <- maf.to.granges('pancancer.maf.compact.filt500k.classes')
```

```
## [1] ">> Reading compact MAF ..."
```

```
maf.pancan=maf.pancan[which(seqnames(maf.pancan) %in% seqnames(seqi))] # 11598778

maf.total=c(maf.pancan,maf.gastric) # 15715077
maf.total=maf.total[-which(maf.total$ctype=="all")] # 15707250 remove ALL cancer as it only has 1 sample
# number of cancer types
length(unique(maf.total$ctype)) # 22
```

```
## [1] 22
```

```
# number of individuals per cancer type
maf.total$sid=as.character(maf.total$sid)
maf.total$ctype=as.character(maf.total$ctype)
maf.total=as.data.frame(maf.total)
maf.total=maf.total[with(maf.total, order(ctype,sid)),]
ind.maf=split(maf.total, maf.total$ctype) # split by cancer type, 22 cancers
sample.count=sapply(ind.maf, function(x) {length(unique(x$sid))}) # number of samples per cancer type
print(sample.count)
```

```
##     aml    astr    blca   brain    brca     cll     crc gastric    hnsc 
##       7     101      20      43     172      28      23     187      29 
##    kich    kirc    lihc    luad    lusc    lymp    medu      ov    paad 
##      15      29      88      64      20      24     100       5      15 
##    prad    skcm    thca    ucec 
##      20      16       9      29
```

```
mut.count=sapply(ind.maf, function(x) {nrow(x)}) # number of mutations per cancer type
print(mut.count)
```

```
##     aml    astr    blca   brain    brca     cll     crc gastric    hnsc 
##    3650   12971  372469  198173 1060612   54613 1086874 4116299  357421 
##    kich    kirc    lihc    luad    lusc    lymp    medu      ov    paad 
##    5019  142419  897472 2619420  887341  141843  139293   23101  122586 
##    prad    skcm    thca    ucec 
##   29862 2248676    9795 1177341
```

```
plot(x=sample.count,y=mut.count,xlab="Number of samples",ylab="Number of mutations",col="blue",pch=19)
text(x=sample.count,y=mut.count,labels=names(sample.count),cex=0.7,pos=3)
abline(lm(mut.count~sample.count),col="red")
```

```
log.sample=log10(sample.count)
log.mut=log10(mut.count)

plot(x=log.sample,y=log.mut,xlab="log10(number of samples)",ylab="log10(number of mutations)",col="blue",pch=19)
text(x=log.sample,y=log.mut,labels=names(sample.count),cex=0.7,pos=3)
abline(lm(log.mut~log.sample),col="red")
```

```
hist(mut.count)
```

```
df=data.frame(ctype=names(mut.count),mut=as.numeric(mut.count))
ggplot(df,aes(x=ctype,y=mut,col=ctype))+geom_point()+theme(axis.text.x = element_text(angle = 90, hjust = 1))+
  ggtitle("Number of mutations per cancer type")
```

```
maf.total$sid=factor(maf.total$sid,levels=unique(maf.total$sid))
ind.maf2=split(maf.total, maf.total$sid) # split by sample IDs, 1044 samples
ind.maf2=sapply(ind.maf2, function(x) {nrow(x)})
print(ind.maf2)
```

```
##            400220            426980            452198            573988 
##               290               839               117               148 
##            758168            804168            869586              PA10 
##               471               618              1167               107 
##             PA102             PA103             PA105             PA107 
##                93                49               339               132 
##             PA109              PA11             PA110             PA112 
##               145               361               103                83 
##             PA116             PA117              PA12             PA131 
##               238               262               207               315 
##             PA134             PA136             PA138              PA14 
##                70               243               106               112 
##             PA143             PA145             PA148             PA149 
##               244               165                76               111 
##             PA157             PA166              PA17              PA20 
##                74               339               107               241 
##              PA21              PA22              PA25               PA3 
##               184               292                98               110 
##              PA36               PA4              PA41              PA43 
##               342               123               132               221 
##              PA46              PA48               PA5              PA53 
##               173               117                82               104 
##              PA54              PA55              PA56              PA58 
##               125                91               100               207 
##              PA59              PA62              PA63              PA64 
##               111               115               291                63 
##              PA65              PA69              PA70              PA73 
##               816               301                99               349 
##              PA75              PA79               PA8              PA81 
##               452                93                62               108 
##              PA82              PA83              PA84              PA85 
##               136               193               195                85 
##              PA86              PA87               PA9              PA90 
##               106               130               127                91 
##              PA93              PA96          SJLGG001          SJLGG002 
##               558               132                51                39 
##          SJLGG003          SJLGG004          SJLGG005          SJLGG006 
##                18                26                29                 7 
##         SJLGG006R          SJLGG007          SJLGG008          SJLGG009 
##                 7                21               159                39 
##          SJLGG010          SJLGG011          SJLGG012          SJLGG013 
##                49                83               100                12 
##          SJLGG015          SJLGG016          SJLGG018          SJLGG019 
##                24                 2                27                 8 
##          SJLGG020          SJLGG021          SJLGG022          SJLGG024 
##                45                15                 3                32 
##          SJLGG025          SJLGG026          SJLGG027          SJLGG028 
##                23                 6                36                35 
##          SJLGG029          SJLGG030          SJLGG031          SJLGG032 
##                44                19                 5                42 
##          SJLGG033          SJLGG034          SJLGG035          SJLGG037 
##                30               198                25                25 
##          SJLGG038          SJLGG039          SJLGG040          SJLGG042 
##                63                12                51               125 
##      TCGA-BL-A13J      TCGA-BT-A20P      TCGA-BT-A20Q      TCGA-BT-A20T 
##             11457              9656              8334             29294 
##      TCGA-BT-A20V      TCGA-BT-A3PH      TCGA-BT-A3PJ      TCGA-C4-A0F7 
##             11428             37085             47193              8116 
##      TCGA-CF-A27C      TCGA-CF-A3MF      TCGA-DK-A1A5      TCGA-DK-A1A6 
##             12229              3069             27633             31044 
##      TCGA-DK-A1A7      TCGA-DK-A1AA      TCGA-DK-A1AE      TCGA-DK-A1AG 
##              2344              8883             12611              8572 
##      TCGA-DK-A3IL      TCGA-FT-A3EE      TCGA-GD-A2C5      TCGA-H4-A2HQ 
##             17230             11447             22743             52101 
##      TCGA-02-2483      TCGA-02-2485      TCGA-06-0157      TCGA-06-0214 
##              3938              5837              4758              6148 
##      TCGA-06-0686      TCGA-06-0744      TCGA-06-0745      TCGA-06-2557 
##              6982              9769              6288              5335 
##      TCGA-06-2570      TCGA-06-5411      TCGA-06-5415      TCGA-14-1823 
##              3102              4078              4842              4775 
##      TCGA-14-2554      TCGA-15-1444      TCGA-19-2620      TCGA-19-2624 
##             10287              1910              7686              3894 
##      TCGA-19-2629      TCGA-19-5960      TCGA-26-5132      TCGA-26-5135 
##              7385              5989              6822              7267 
##      TCGA-27-1831      TCGA-27-2523      TCGA-27-2528      TCGA-32-1970 
##              5500              6822              6133              7681 
##      TCGA-41-5651      TCGA-CS-5395      TCGA-CS-6668      TCGA-DB-5278 
##              9877              5133              2617               405 
##      TCGA-DU-5874      TCGA-DU-6401      TCGA-DU-6407      TCGA-DU-7009 
##              3767              2183              1848              1525 
##      TCGA-DU-7301      TCGA-E1-5318      TCGA-E1-5319      TCGA-EZ-7264 
##              2474              2482              1823              2294 
##      TCGA-FG-5964      TCGA-FG-8182      TCGA-HT-7602      TCGA-HT-7689 
##              2573              2539               931              3437 
##      TCGA-HT-7695      TCGA-HW-7487      TCGA-IK-7675           PD3851a 
##              1328              1986              5723              1561 
##           PD3890a           PD3904a           PD3905a           PD3945a 
##              5608              5379              4272              9934 
##           PD3989a           PD4005a           PD4006a           PD4069a 
##              2080              5773              8711              2068 
##           PD4072a           PD4080a           PD4085a           PD4086a 
##             26885              1746              2438              1929 
##           PD4088a           PD4103a           PD4107a           PD4109a 
##              1576              5042              9879              9405 
##           PD4115a           PD4116a           PD4120a           PD4192a 
##              9548              7682             67364              3439 
##           PD4194a           PD4198a           PD4199a           PD4224a 
##              1241              4180              6524              7955 
##           PD4225a           PD4248a           PD4255a           PD4261a 
##              2104              2188              4584              1955 
##           PD4266a           PD4267a           PD4315a           PD4604a 
##              2706              2417              4001              8585 
##           PD4605a           PD4606a           PD4607a           PD4608a 
##              2450              1390             25464              2306 
##           PD4613a           PD4826a           PD4833a           PD4836a 
##              1986              3973              7026              4433 
##           PD4841a           PD4847a           PD4951a           PD4952a 
##              7192             11521              2973             12156 
##           PD4953a           PD4954a           PD4955a           PD4957a 
##              7193              5341              6218              2961 
##           PD4958a           PD4959a           PD4962a           PD4963a 
##              8332              4234              5542              1481 
##           PD4965a           PD4966a           PD4967a           PD4968a 
##              2845              1043              3579              4206 
##           PD4970a           PD4971a           PD4972a           PD4975a 
##              2170              2520              1054              6867 
##           PD4976a           PD4980a           PD4981a           PD4982a 
##              5640              4128              1844              1564 
##           PD4983a           PD4985a           PD4986a           PD5928a 
##              1614              1821              1803              6492 
##           PD5934a           PD5935a           PD5936a           PD5942a 
##             11873             10701              2562              6146 
##           PD5944a           PD5947a           PD5951a           PD5956a 
##              2785              1659              4020              4447 
##           PD6018a           PD6041a           PD6042a           PD6043a 
##              2732              1613              6544             22443 
##           PD6044a           PD6045a           PD6046a           PD6049a 
##              2461              2079              3334              7261 
##           PD6409a           PD6410a           PD6411a           PD6413a 
##              6133              7132              7559              4834 
##           PD6417a           PD6418a           PD6422a           PD6466b 
##              1442              1924              4675              1510 
##           PD6719a           PD6720a           PD6721a           PD6722a 
##              4593              1876              2053             10978 
##           PD7199a           PD7201a           PD7207a           PD7208a 
##              1079              1462              2582              2269 
##           PD7209a           PD7210a           PD7212a           PD7214a 
##              1533              1273              1885              1813 
##           PD7215a           PD7216a           PD7217a           PD7218a 
##              6871              1537             10005              1826 
##           PD7219a           PD7221a           PD7321a           PD7404a 
##             12608              1873              6384             12663 
##           PD7409a           PD7431a           PD7433a           PD8618a 
##              7730              3946              2012              1357 
##           PD8622a           PD8623a      TCGA-A1-A0SM      TCGA-A2-A04P 
##              1733              1694              2197              9645 
##      TCGA-A2-A04Q      TCGA-A2-A04T      TCGA-A2-A04X      TCGA-A2-A0CM 
##              2484              7800              3956              7954 
##      TCGA-A2-A0D0      TCGA-A2-A0D1      TCGA-A2-A0D2      TCGA-A2-A0EY 
##              7874              4285              7872             20698 
##      TCGA-A2-A0YG      TCGA-A7-A0CE      TCGA-A8-A075      TCGA-A8-A07B 
##              4817              8382              7938              6547 
##      TCGA-A8-A07I      TCGA-A8-A08B      TCGA-A8-A08L      TCGA-A8-A08S 
##              4567              4263             22281              4411 
##      TCGA-A8-A092      TCGA-A8-A094      TCGA-A8-A09I      TCGA-A8-A09X 
##              7656             20919             14011              4516 
##      TCGA-AN-A0AT      TCGA-AN-A0G0      TCGA-AO-A03L      TCGA-AO-A03N 
##              9962              3630              2827              9390 
##      TCGA-AO-A0J2      TCGA-AO-A0J4      TCGA-AO-A0J6      TCGA-AO-A0JM 
##              8238              7386              9513              3629 
##      TCGA-AQ-A04J      TCGA-AR-A0TX      TCGA-B6-A0I2      TCGA-B6-A0I6 
##              5352             30398              4662             10182 
##      TCGA-B6-A0IJ      TCGA-B6-A0IQ      TCGA-B6-A0RE      TCGA-B6-A0RT 
##             13794              5605             10993              4868 
##      TCGA-B6-A0RU      TCGA-BH-A0B3      TCGA-BH-A0B9      TCGA-BH-A0E0 
##              4011              2191              2890              3294 
##      TCGA-BH-A0WA      TCGA-BH-A18R      TCGA-BH-A18U      TCGA-C8-A12L 
##              7248              3765             12126              6710 
##      TCGA-C8-A12Q      TCGA-C8-A130      TCGA-E2-A14P      TCGA-E2-A152 
##             11618              3939              8353             13271 
##      TCGA-E2-A15E      TCGA-E2-A15H      TCGA-GM-A2DF     001-0002-03TD 
##              2709              1938              3422               955 
##     003-0005-09TD        012-02-1TD               125               128 
##              1087               990              1843              1701 
##               137               141               151               178 
##              2024              3228              2497              3061 
##               192                26               277               282 
##              3197              1935               598              1901 
##               294               306               308               318 
##               564              1732              2298              1308 
##               342               343               367               393 
##              1986              1689              2589              2191 
##               467               473               477               519 
##              2985              2199              3128              1641 
##               523               564      CLL4-ARTICLE      TCGA-A6-2680 
##              3456               821              1009             10876 
##      TCGA-A6-2681      TCGA-A6-2683      TCGA-A6-6141      TCGA-AA-3516 
##             13941             25529            225030            150924 
##      TCGA-AA-3555      TCGA-AA-3666      TCGA-AA-3956      TCGA-AA-A01R 
##            252803             27457             16278            120682 
##      TCGA-AA-A01S      TCGA-AA-A01T      TCGA-AA-A02O      TCGA-AA-A02Y 
##              6684             12814             19877             12467 
##      TCGA-AA-A03F      TCGA-AF-2691      TCGA-AG-3574      TCGA-AG-3582 
##             14692             23279              8627              6727 
##      TCGA-AG-3885      TCGA-AG-3896      TCGA-AG-3901      TCGA-AG-4015 
##             10152             13864              8515             12661 
##      TCGA-AG-A032      TCGA-D5-6540       apollo1_new          apollo10 
##              7236             85759              6688             18230 
##          apollo11          apollo12          apollo13          apollo14 
##             14105             13200              7925             22276 
##          apollo15          apollo16          apollo17          apollo19 
##             17799             87606             17914             28620 
##           apollo2          apollo20          apollo21          apollo22 
##             11828             18049             12992             27974 
##          apollo23          apollo24          apollo25           apollo3 
##             25686             86137              5123              9164 
##           apollo6           apollo7           apollo8           apollo9 
##              5463             33824              4526            264557 
## CGP_donor_GC00002 CGP_donor_GC00014 CGP_donor_GC00015 CGP_donor_GC00017 
##             30100              1754              4754              6268 
## CGP_donor_GC00018 CGP_donor_GC00019 CGP_donor_GC00020 CGP_donor_GC00027 
##              2304              8111             18526              1259 
## CGP_donor_GC00028 CGP_donor_GC00029 CGP_donor_GC00030 CGP_donor_GC00031 
##              3752              9525              1132             55172 
## CGP_donor_GC00033 CGP_donor_GC00037 CGP_donor_GC00038 CGP_donor_GC00039 
##             14105              2017              9376             22213 
## CGP_donor_GC00047 CGP_donor_GC00048 CGP_donor_GC00049 CGP_donor_GC00050 
##             12779             14744              8174              3351 
## CGP_donor_GC00051 CGP_donor_GC00052 CGP_donor_GC00053 CGP_donor_GC00054 
##             14357             34962             13444             11154 
##         HK-pfg005         HK-pfg008         HK-pfg022         HK-pfg030 
##              1023            103405              9321             15795 
##         HK-pfg031         HK-pfg032         HK-pfg034         HK-pfg035 
##              1528              5847             20996              8207 
##         HK-pfg036         HK-pfg038         HK-pfg039         HK-pfg043 
##              3928             17010              7431             59461 
##         HK-pfg050         HK-pfg052         HK-pfg054         HK-pfg057 
##              2308              6055             24378             39194 
##         HK-pfg058         HK-pfg059         HK-pfg060         HK-pfg062 
##             15104             35611             19495             17395 
##         HK-pfg064         HK-pfg065         HK-pfg068         HK-pfg069 
##             12645             21725             92284             18423 
##         HK-pfg072         HK-pfg073         HK-pfg076         HK-pfg081 
##             17806             10990             36577             16456 
##         HK-pfg082         HK-pfg088         HK-pfg089         HK-pfg092 
##              5620             14292             21753             49544 
##         HK-pfg097         HK-pfg103         HK-pfg104         HK-pfg105 
##              7731             16767              7203             19221 
##         HK-pfg106         HK-pfg107         HK-pfg108         HK-pfg115 
##              9730             19610             32188             15505 
##         HK-pfg116         HK-pfg118         HK-pfg119         HK-pfg120 
##             28492              9910             17069             26231 
##         HK-pfg121         HK-pfg122         HK-pfg124         HK-pfg125 
##             10164             74549             11220             16112 
##         HK-pfg127         HK-pfg129         HK-pfg130         HK-pfg132 
##             48082             28085              9683              2201 
##         HK-pfg135         HK-pfg136         HK-pfg142         HK-pfg143 
##              8849              3422              1814             50372 
##         HK-pfg144         HK-pfg145         HK-pfg146         HK-pfg151 
##             18087             18608             30020              3353 
##         HK-pfg156         HK-pfg157         HK-pfg160         HK-pfg164 
##              1093              7050             63918              2403 
##         HK-pfg166         HK-pfg167         HK-pfg173         HK-pfg180 
##              5792              8659             12942             27601 
##         HK-pfg181         HK-pfg182         HK-pfg205         HK-pfg212 
##             98316             10649             12931             47770 
##         HK-pfg213         HK-pfg217         HK-pfg220         HK-pfg222 
##              3520             12923              3076             16503 
##         HK-pfg228         HK-pfg258         HK-pfg272         HK-pfg277 
##              4095              7114             36743             15265 
##         HK-pfg282         HK-pfg311         HK-pfg316         HK-pfg317 
##              7316             14764             10097              6803 
##         HK-pfg344         HK-pfg373         HK-pfg375         HK-pfg378 
##             15917             10631              3344              7930 
##         HK-pfg398         HK-pfg413         HK-pfg416         HK-pfg424 
##              1018             14277              4479              6381 
##        tan2000639       tan21080055       tan76629543         tan980401 
##             19888              8447             35245              5061 
##         tan980417         tan980437         tan980447         tan990275 
##              6038             32643             12876             13719 
##         tan990489      TCGA-BR-4255      TCGA-BR-4280      TCGA-BR-6452 
##             60905              3655             56130            278055 
##      TCGA-BR-6456      TCGA-BR-6564      TCGA-BR-7722      TCGA-BR-8373 
##              6057              2225              4939             16620 
##      TCGA-BR-8381      TCGA-BR-8486      TCGA-BR-8682      TCGA-BR-8690 
##              8214              4826              7212             21719 
##      TCGA-BR-A4J4      TCGA-CD-5799      TCGA-CD-5802      TCGA-CD-8529 
##              8681              3964              4771             22125 
##      TCGA-CG-4442      TCGA-CG-4443      TCGA-CG-4474      TCGA-CG-5723 
##             96689              9925              9525            101427 
##      TCGA-CG-5724      TCGA-CG-5730      TCGA-D7-5579      TCGA-D7-6518 
##             26467             15607             18994              6070 
##      TCGA-D7-6519      TCGA-D7-6527      TCGA-D7-6528      TCGA-D7-6815 
##              5400             54771             48481             11669 
##      TCGA-D7-6822      TCGA-D7-8570      TCGA-D7-A4YX      TCGA-EQ-5647 
##             61132             11952              8188              9670 
##      TCGA-F1-6177      TCGA-F1-6875      TCGA-FP-7998      TCGA-HF-7136 
##             73327             11279              3240             10781 
##      TCGA-HU-8245      TCGA-HU-8608      TCGA-HU-A4G6      TCGA-HU-A4H0 
##              7448              8423             10837             21876 
##      TCGA-IN-7806      TCGA-BA-4076      TCGA-BA-4077      TCGA-BA-5153 
##              8882             64796             24722              3003 
##      TCGA-BA-6873      TCGA-BA-A4IH      TCGA-BB-4225      TCGA-CN-4737 
##              5119              3657              5561              4475 
##      TCGA-CN-4741      TCGA-CN-5365      TCGA-CN-5374      TCGA-CR-5250 
##             12375              6760             20355              3333 
##      TCGA-CR-6467      TCGA-CR-6470      TCGA-CR-6472      TCGA-CR-6480 
##              3154              2849             42043              7068 
##      TCGA-CR-6482      TCGA-CR-6487      TCGA-CR-7385      TCGA-CR-7391 
##              3713             12231              3978               310 
##      TCGA-CR-7404      TCGA-CV-5442      TCGA-CV-5443      TCGA-CV-6433 
##             11544             26712              4745              5467 
##      TCGA-CV-6961      TCGA-CV-7090      TCGA-CV-7100      TCGA-CV-7180 
##             45692              5123              2766              8035 
##      TCGA-CV-7255      TCGA-CV-7416      TCGA-KL-8323      TCGA-KL-8325 
##              9499              8336              2226               108 
##      TCGA-KL-8341      TCGA-KL-8342      TCGA-KM-8440      TCGA-KN-8418 
##                92               187               322                70 
##      TCGA-KN-8424      TCGA-KN-8429      TCGA-KN-8434      TCGA-KN-8435 
##                29               169                25               923 
##      TCGA-KO-8406      TCGA-KO-8407      TCGA-KO-8411      TCGA-KO-8416 
##                90               338               277                16 
##      TCGA-KO-8417      TCGA-A3-3308      TCGA-A3-3324      TCGA-A3-3370 
##               147              6855              3944              3318 
##      TCGA-A3-3372      TCGA-A3-3387      TCGA-AK-3428      TCGA-AK-3454 
##              6574              4510              4539              3388 
##      TCGA-AK-3455      TCGA-B0-5094      TCGA-B0-5693      TCGA-B2-4101 
##              4319              6242              3049              4557 
##      TCGA-BP-4327      TCGA-BP-4781      TCGA-BP-4968      TCGA-BP-4977 
##              2745              6686              3616              3451 
##      TCGA-BP-5010      TCGA-BP-5168      TCGA-CJ-4639      TCGA-CJ-4885 
##              4793              8375              3898              4781 
##      TCGA-CJ-4899      TCGA-CJ-4918      TCGA-CJ-5682      TCGA-CJ-6033 
##              2818              6959              6328              6148 
##      TCGA-CW-6087      TCGA-CW-6093      TCGA-CZ-4856      TCGA-CZ-5454 
##              5905              7839              5604              3532 
##      TCGA-CZ-5987      TCGA-DV-5566             HX10T             HX11T 
##              3690              3956             10523              9564 
##             HX12T             HX13T             HX14T             HX15T 
##             16083             24125             11880              8907 
##             HX16T             HX17T             HX18T             HX19T 
##             10329             22851              7931             12572 
##             HX20T             HX21T             HX22T             HX23T 
##              6021             14566              3979              3937 
##             HX25T             HX28T             HX30T             HX33T 
##              4687              9120             11335             15304 
##             HX35T              HX4T              HX5T              HX9T 
##             11361              9408              9876              9219 
##         RK001_C01         RK002_C01         RK003_C01         RK004_C01 
##             20104             12998              7095             15946 
##         RK005_C01         RK006_C01         RK006_C02         RK007_C01 
##              2529             17261             19069             11577 
##         RK010_C01         RK012_C01         RK015_C01         RK016_C01 
##              7055              7174             13024              8082 
##         RK018_C01         RK019_C01         RK020_C01         RK021_C01 
##              1432             16428              5135             16377 
##         RK022_C01         RK023_C01         RK024_C01         RK025_C01 
##             12586             19534              4965              8364 
##         RK026_C01         RK027_C01         RK029_C01         RK031_C01 
##              6296             23222              7225              6984 
##         RK032_C01         RK033_C01         RK034_C01         RK035_C01 
##             11053              3924              4861             25727 
##         RK036_C01         RK037_C01         RK041_C01         RK042_C01 
##              4640              5134             20069              8447 
##         RK046_C01         RK046_C02         RK047_C01         RK048_C01 
##              8969             11339              8411             14434 
##         RK049_C01         RK050_C01         RK051_C01         RK054_C01 
##              2788              8908             11844              6322 
##         RK055_C01         RK056_C01         RK063_C01         RK067_C01 
##              1948             17116              6405             14261 
##         RK068_C01         RK069_C01         RK075_C01         RK079_C01 
##              2537              4717              8904             12543 
##         RK083_C01         RK084_C01         RK086_C01         RK089_C01 
##              8690              9000             14761              9831 
##         RK092_C01         RK098_C01         RK099_C01         RK100_C01 
##             11129              6496              6978              8898 
##         RK106_C01         RK107_C01         RK108_C01         RK109_C01 
##             12299             12011              6943              5597 
##         RK126_C01         RK130_C01         RK133_C01         RK137_C01 
##             18275              4929              5959              8343 
##         RK138_C01         RK141_C01         LU-A08-43        LUAD-2GUGK 
##              2889              5103             11920             47253 
##        LUAD-5V8LT        LUAD-AEIUF       LUAD-D02326       LUAD-E00934 
##            305665             34708             10475            115244 
##       LUAD-E01014       LUAD-E01278       LUAD-E01317        LUAD-FH5PJ 
##             24720             49496             37783             37322 
##        LUAD-QY22Z       LUAD-S00488       LUAD-S01302       LUAD-S01331 
##             76262             76974            131255             45740 
##       LUAD-S01341       LUAD-S01345       LUAD-S01346       LUAD-S01356 
##             16920             41426             87912             71297 
##       LUAD-S01381       LUAD-S01404       LUAD-S01405       LUAD-S01467 
##             29245             35801             51249             47868 
##       LUAD-S01478        LUAD-U6SJ7      TCGA-05-4389      TCGA-05-4395 
##             81649             32240             21340             40408 
##      TCGA-05-4396      TCGA-05-4397      TCGA-05-4398      TCGA-05-4420 
##             40311             93860            104046             32025 
##      TCGA-05-4422      TCGA-05-4432      TCGA-05-5429      TCGA-38-4628 
##              4226             75763              2887             12648 
##      TCGA-38-4630      TCGA-44-2659      TCGA-44-2665      TCGA-44-2666 
##             13995             48536              3508              1924 
##      TCGA-44-6148      TCGA-49-4486      TCGA-49-4510      TCGA-49-4512 
##               418             11652              5195              4239 
##      TCGA-49-6742      TCGA-50-5066      TCGA-50-6597      TCGA-55-1594 
##             45176             40445              7476             16151 
##      TCGA-55-1596      TCGA-55-6982      TCGA-55-6986      TCGA-55-7281 
##             23402             24669              1703             62944 
##      TCGA-64-1678      TCGA-64-1680      TCGA-67-3771      TCGA-67-3772 
##             56781              2796            137860              2796 
##      TCGA-67-6215      TCGA-73-4659      TCGA-73-4666      TCGA-75-5147 
##              9082             23611             40372              4621 
##      TCGA-75-6203      TCGA-78-7143      TCGA-78-7146      TCGA-78-7156 
##              1268               849             42012             25209 
##      TCGA-78-7535      TCGA-91-6840      TCGA-21-1076      TCGA-21-1078 
##             17679             15113             41528              5036 
##      TCGA-21-1082      TCGA-22-1016      TCGA-34-2596      TCGA-34-2600 
##             42331             49685             42150             50905 
##      TCGA-43-3394      TCGA-43-3920      TCGA-56-1622      TCGA-60-2695 
##             29910             43366             40603             26507 
##      TCGA-60-2698      TCGA-60-2711      TCGA-60-2713      TCGA-60-2719 
##            114374             18063             34938             23703 
##      TCGA-60-2722      TCGA-60-2724      TCGA-60-2726      TCGA-66-2756 
##             50593             50729             61534             72091 
##      TCGA-66-2757      TCGA-66-2766           4101316           4105105 
##             33167             56128              2391              3644 
##           4108101           4112512           4116738           4119027 
##              3379              2202              8062              2587 
##           4121361           4125240           4133511           4135350 
##              6335              2106              3758             13325 
##           4142267           4158726           4159170           4163639 
##              1636              4360              3459             39117 
##           4175837           4177856           4182393           4189200 
##              4694              3807              1644              3711 
##           4189998           4190495           4193278           4194218 
##              3729              4946              2791              2192 
##           4194891                G1           LFS_MB1           LFS_MB2 
##              1829             16139              2277              1816 
##           LFS_MB4               MB1             MB101             MB102 
##              1373               458              4268              1864 
##             MB104             MB106             MB107             MB108 
##              2487               442              1963               601 
##             MB110             MB112             MB113             MB114 
##              3687              1243              1216               558 
##             MB115             MB117             MB119              MB12 
##               656              1114               262               236 
##             MB121             MB122             MB124             MB125 
##               634              1182              1796              3257 
##             MB126             MB127             MB128             MB129 
##              3643              2881              9867              1339 
##             MB130             MB131             MB132             MB134 
##              1136              1274               320               692 
##             MB139              MB15              MB16              MB17 
##               649               977               398               725 
##              MB18              MB19               MB2              MB20 
##              1217               735               393               952 
##              MB21              MB23              MB24              MB26 
##               392              2174               308               903 
##              MB28               MB3              MB31              MB32 
##                59              1086              1839               605 
##              MB34              MB35              MB36              MB37 
##              3510               338              1363                95 
##              MB38              MB39              MB40              MB45 
##               785               235               126              1720 
##              MB46              MB49               MB5              MB50 
##               753               769               620              1301 
##              MB51             MB518              MB53              MB56 
##               523              1219              1738              2873 
##              MB57              MB58              MB59               MB6 
##               833              1714              2469              1167 
##              MB60              MB61             MB612              MB63 
##              1347              2810               349              2118 
##              MB64              MB66              MB67              MB69 
##               985              2035              1042              2286 
##               MB7              MB70              MB74              MB75 
##               816              1736              2362              2232 
##              MB77              MB78              MB79               MB8 
##              1893              3268              1833               817 
##             MB800              MB81              MB82              MB83 
##              1132              4368              1755               860 
##              MB84              MB85              MB86              MB88 
##              1133               232               520               309 
##              MB89               MB9              MB90              MB91 
##               598               607               326              1023 
##              MB92              MB94              MB95              MB96 
##              1785              2157               312               370 
##              MB98              MB99      TCGA-13-0723      TCGA-13-0890 
##              1013               759              3936              4642 
##      TCGA-13-1411      TCGA-24-0980      TCGA-24-1103         APGI_1839 
##              4530              3886              6107              5502 
##         APGI_1840         APGI_1956         APGI_1992         APGI_2000 
##             12906              5088             10167              8281 
##         APGI_2051         APGI_2057         APGI_2060         APGI_2119 
##              5509             15580              6992              4318 
##         APGI_2137         APGI_2150         APGI_2157         APGI_2179 
##             11921              1301              7500             15323 
##         APGI_2202         APGI_2353      TCGA-CH-5750      TCGA-CH-5763 
##              4147              8051              3615               394 
##      TCGA-CH-5771      TCGA-CH-5788      TCGA-CH-5789      TCGA-EJ-5503 
##              2303              3361               104               161 
##      TCGA-EJ-5506      TCGA-EJ-7791      TCGA-G9-6336      TCGA-G9-6365 
##               170              1345              1550              2519 
##      TCGA-G9-6370      TCGA-G9-7522      TCGA-HC-7075      TCGA-HC-7079 
##                95              1090              2899                58 
##      TCGA-HC-7233      TCGA-HC-7737      TCGA-HC-7740      TCGA-HC-7744 
##              2765              1385               892              1852 
##      TCGA-HC-8258      TCGA-HI-7169      TCGA-D9-A148      TCGA-DA-A1IC 
##               804              2500             46655            141622 
##      TCGA-EB-A24D      TCGA-EE-A29B      TCGA-EE-A2M5      TCGA-EE-A2MI 
##             88859             94850            371487            232209 
##      TCGA-EE-A3J5      TCGA-EE-A3JI      TCGA-ER-A19E      TCGA-ER-A19T 
##            337346            163855             71067              4571 
##      TCGA-ER-A2NF      TCGA-ER-A3ES      TCGA-FS-A1ZD      TCGA-FS-A1ZK 
##              3070              1305             39120            216156 
##      TCGA-FS-A1ZP      TCGA-GN-A26C      TCGA-BJ-A191      TCGA-DJ-A13W 
##             91750            344754              2065              1015 
##      TCGA-EL-A3H1      TCGA-EL-A3TB      TCGA-EM-A3AL      TCGA-EM-A3AQ 
##              2005               903               774               871 
##      TCGA-FE-A3PD      TCGA-FK-A3S3      TCGA-FK-A3SE      TCGA-A5-A0G9 
##               968               539               655             24043 
##      TCGA-A5-A0GA      TCGA-A5-A0GE      TCGA-A5-A0GG      TCGA-AP-A052 
##             25028              3536             17197              3692 
##      TCGA-AP-A053      TCGA-AP-A054      TCGA-AP-A05A      TCGA-AP-A05D 
##              4410            111303              6817              3692 
##      TCGA-AP-A0L8      TCGA-AP-A0L9      TCGA-AP-A0LD      TCGA-AP-A0LE 
##              6663              8987             34912             41318 
##      TCGA-AP-A0LH      TCGA-AP-A0LI      TCGA-AP-A0LL      TCGA-AP-A0LO 
##              3766             14150              3298            337569 
##      TCGA-AX-A05S      TCGA-AX-A1CI      TCGA-B5-A0JN      TCGA-B5-A11G 
##             31021              4131             15116             62743 
##      TCGA-B5-A11H      TCGA-BK-A0CC      TCGA-BS-A0TC      TCGA-BS-A0TD 
##             64733              6429            286464              3861 
##      TCGA-BS-A0TE      TCGA-BS-A0TG      TCGA-D1-A16G      TCGA-EY-A1GW 
##             38592              4031              4893              4946
```

```
hist(ind.maf2)
```

```
df=data.frame(ctype=rep(unique(maf.total$ctype),as.numeric(sample.count)),mut=as.numeric(ind.maf2))
ggplot(df,aes(x=ctype,y=mut,col=ctype))+geom_point()+
  theme(axis.text.x = element_text(angle = 90, hjust = 1))+
  ggtitle("Number of mutations per sample in each cancer type")
```

Set cutoff as 200000 mutations, remove samples with more than 200000 mutation count.

```
sum(ind.maf2>=200000) # 12, 2 of which are gastric samples
```

```
## [1] 12
```

```
maf.total=c(maf.pancan,maf.gastric) # 15715077
maf.total=maf.total[-which(maf.total$ctype=="all")] # 15707250
maf.total$ctype=as.character(maf.total$ctype)
maf.total$sid=as.character(maf.total$sid)
maf.total$ctype=factor(maf.total$ctype)
maf.total2=maf.total[which(! maf.total$sid %in% names(ind.maf2[which(ind.maf2>=200000)]))] # 12255155
length(unique(maf.total2$ctype)) # 22
```

```
## [1] 22
```

```
ind.maf4=split(maf.total2, maf.total2$ctype) # split by cancer type
sample.count4=sapply(ind.maf4, function(x) {length(unique(x$sid))}) # 1032
print(sample.count4) # number of samples per cancer type
```

```
##     aml    astr    blca   brain    brca     cll     crc gastric    hnsc 
##       7     101      20      43     172      28      21     185      29 
##    kich    kirc    lihc    luad    lusc    lymp    medu      ov    paad 
##      15      29      88      63      20      24     100       5      15 
##    prad    skcm    thca    ucec 
##      20      11       9      27
```

```
# Find the number of mutated samples at the CBS hotspots (11)
ovl=findOverlaps(maf.total2,ctcf.hotspot) # 128
pancan.cbs=maf.total2[queryHits(ovl)]

ind.cbs=split(pancan.cbs, pancan.cbs$ctype) # 22
cbs.count=sapply(ind.cbs, function(x) {length(unique(x$sid))})
print(cbs.count) # number of samples that overlapped with CBS hotspots per cancer type
```

```
##     aml    astr    blca   brain    brca     cll     crc gastric    hnsc 
##       0       0       0       0       5       0       4      47       0 
##    kich    kirc    lihc    luad    lusc    lymp    medu      ov    paad 
##       0       0      10       4       1       1       0       0       1 
##    prad    skcm    thca    ucec 
##       0       0       0       0
```

```
mut.frac=cbs.count/sample.count4
print(mut.frac)
```

```
##        aml       astr       blca      brain       brca        cll 
## 0.00000000 0.00000000 0.00000000 0.00000000 0.02906977 0.00000000 
##        crc    gastric       hnsc       kich       kirc       lihc 
## 0.19047619 0.25405405 0.00000000 0.00000000 0.00000000 0.11363636 
##       luad       lusc       lymp       medu         ov       paad 
## 0.06349206 0.05000000 0.04166667 0.00000000 0.00000000 0.06666667 
##       prad       skcm       thca       ucec 
## 0.00000000 0.00000000 0.00000000 0.00000000
```

```
nonmut.frac=(sample.count4-cbs.count)/sample.count4
unique(mut.frac+nonmut.frac)
```

```
## [1] 1
```

```
### stacked barplot
df=data.frame(ctype=rep(names(sample.count4),2),group=rep(c("mut","nonmut"),each=22),rate=c(mut.frac,nonmut.frac)) # 44
df$cancer=rep(c("Acute myeloid leukemia",
                "Astrocytoma","Bladder urothelial carcinoma",
                "Brain cancer","Breast cancer",
                "Chronic lymphocytic leukemia","Colorectal cancer","Gastric cancer",
                "Head and neck squamous cell","Kidney chromophobe",
                "Kidney renal clear cell carcinoma","Liver hepatocellular carcinoma",
                "Lung adenocarcinoma","Lung squamous cell carcinoma",
                "Lymphoma","Medulloblastoma",
                "Ovarian cancer","Pancreas adenocarcinoma",
                "Prostate adenocarcinoma","Skin cutaneous melanoma",
                "Thyroid cancer","Uterine corpus endometrial carcinoma"),2)
# remove cancer types with less than 10 samples
df$ctype=as.character(df$ctype)
df=df[which(! df$ctype %in% names(sample.count4[which(sample.count4<10)])),] # AML, OV and THCA have less than 10 samples

ggplot(df,aes(x=cancer,y=rate,fill=group))+geom_bar(stat="identity",position=position_stack(reverse=TRUE))+
  coord_flip()+scale_fill_manual(values=c("#FF6633","#999999"))+
  theme(text = element_text(size=20),axis.text.x = element_text(size=20),legend.position="none",panel.background =element_rect(fill = "white", color="black"))
```

```
# cutoff at 0.3
nonmut.frac2=0.3-mut.frac

df2=data.frame(ctype=rep(names(sample.count4),2),group=rep(c("mut","nonmut"),each=22),rate=c(mut.frac,nonmut.frac2)) #44
df2$cancer=rep(c("Acute myeloid leukemia",
                "Astrocytoma","Bladder urothelial carcinoma",
                "Brain cancer","Breast cancer",
                "Chronic lymphocytic leukemia","Colorectal cancer","Gastric cancer",
                "Head and neck squamous cell","Kidney chromophobe",
                "Kidney renal clear cell carcinoma","Liver hepatocellular carcinoma",
                "Lung adenocarcinoma","Lung squamous cell carcinoma",
                "Lymphoma","Medulloblastoma",
                "Ovarian cancer","Pancreas adenocarcinoma",
                "Prostate adenocarcinoma","Skin cutaneous melanoma",
                "Thyroid cancer","Uterine corpus endometrial carcinoma"),2)
# remove cancer types with less than 10 samples
df2$ctype=as.character(df2$ctype)
df2=df2[which(! df2$ctype %in% names(sample.count4[which(sample.count4<10)])),] # AML, OV and UCEC have less than 10 samples

cancer.type=df2[which(df2$group=="mut"),]
cancer.type=cancer.type[order(cancer.type$rate,decreasing=FALSE),]
cancer.type=cancer.type$ctype
df2$ctype=factor(df2$ctype,levels=cancer.type)
df2=df2[order(df2$ctype),]
df2$cancer=factor(df2$cancer,levels=unique(df2$cancer))

ggplot(df2,aes(x=cancer,y=rate,fill=group))+geom_bar(stat="identity",position=position_stack(reverse=TRUE))+
  coord_flip()+scale_fill_manual(values=c("#FF6633","#999999"))+
  theme(text = element_text(size=20),axis.text.x = element_text(size=20),legend.position="none",panel.background =element_rect(fill = "white", color="black"))
```

## Figure C

Proportion of samples in each cancer type that overlap with each of the 11 CTCF hotspots

```
# For each of the 11 CTCF hotspots, barplot showing the number of mutated samples contributed by each ctype
k <- read.delim("gastric_hotspot_snv_unique_annotated_remove5_corrected.tsv", stringsAsFactors=FALSE)
k=GRanges(k$chrom,IRanges(k$start,k$end),pval=k$pval)
z=findOverlaps(ctcf.hotspot,k) # 11
ovl=ctcf.hotspot[queryHits(z)]
ovl$pval=k[subjectHits(z)]$pval
ovl=as.data.frame(ovl)
ovl=ovl[order(ovl$pval,decreasing=FALSE),]
print(ovl)
```

```
##    seqnames     start       end width strand hotspot         pval
## 1      chr6  50570094  50570120    27      *       1 5.403225e-23
## 4      chr8  71000992  71001012    21      *       9 1.085230e-18
## 6      chr1 209422184 209422213    30      *      15 1.936060e-16
## 5      chr2  49173777  49173807    31      *      10 4.050512e-16
## 7      chr4 182064580 182064603    24      *      19 3.090696e-15
## 8      chrX 104435116 104435138    23      *      20 4.261193e-15
## 9     chr14  70285576  70285601    26      *      27 8.395098e-15
## 2      chr6  73122093  73122115    23      *       2 9.160762e-15
## 3      chr8  70576141  70576166    26      *       8 6.116142e-14
## 10    chr13  36552830  36552850    21      *      30 2.570223e-13
## 11     chr3 164903700 164903728    29      *      32 5.722585e-13
```

```
print(as.data.frame(ctcf.hotspot))
```

```
##    seqnames     start       end width strand hotspot
## 1      chr6  50570094  50570120    27      *       1
## 2      chr6  73122093  73122115    23      *       2
## 3      chr8  70576141  70576166    26      *       8
## 4      chr8  71000992  71001012    21      *       9
## 5      chr2  49173777  49173807    31      *      10
## 6      chr1 209422184 209422213    30      *      15
## 7      chr4 182064580 182064603    24      *      19
## 8      chrX 104435116 104435138    23      *      20
## 9     chr14  70285576  70285601    26      *      27
## 10    chr13  36552830  36552850    21      *      30
## 11     chr3 164903700 164903728    29      *      32
```

```
# reorder ctcf.hotspot according to increasing p-values
ctcf.hotspot=ctcf.hotspot[as.numeric(rownames(ovl))] # arrange in decreasing significance
ctcf.hotspot$hotspot=paste("hotspot",c(1:11),sep="")
ovl=findOverlaps(maf.total2,ctcf.hotspot) # 128
pancan.cbs=maf.total2[queryHits(ovl)]
pancan.cbs$hotspot=ctcf.hotspot[subjectHits(ovl)]$hotspot
pancan.cbs=as.data.frame(pancan.cbs)
pancan.cbs=aggregate(sid~ctype+hotspot,pancan.cbs,unique) # 33
pancan.cbs$count=numeric(nrow(pancan.cbs))
for (i in 1:nrow(pancan.cbs)){
  pancan.cbs$count[i]=length(pancan.cbs$sid[[i]])
}
pancan.cbs$hotspot=factor(pancan.cbs$hotspot,levels=ctcf.hotspot$hotspot)

# in proportions
pancan.cbs$prop=numeric(nrow(pancan.cbs))
for(i in 1:nrow(pancan.cbs)){
  pancan.cbs$prop[i]=pancan.cbs$count[i]/sample.count4[pancan.cbs$ctype[i]]
}
# remove cancer types with less than 10 samples
pancan.cbs=pancan.cbs[which(!pancan.cbs$ctype %in% names(sample.count4[which(sample.count4<10)])),] # 33

# stacked barplot
ggplot(pancan.cbs,aes(x=hotspot,y=prop,fill=ctype))+geom_bar(stat="identity",colour="black")+
  theme(axis.text.x = element_text(angle = 90, hjust = 1))+
  theme(text = element_text(size=20),axis.text.x = element_text(size=20),legend.position="none",panel.background =element_rect(fill = "white", color="black"))+
  theme(legend.position = "right")+
  scale_fill_brewer(palette = "Set1")
```

```
# clustered barplot
pancan.cbs2=data.frame(ctype=rep(unique(pancan.cbs$ctype),each=length(unique(pancan.cbs$hotspot))),hotspot=rep(unique(pancan.cbs$hotspot),length(unique(pancan.cbs$ctype))))
pancan.cbs2=merge(pancan.cbs2,pancan.cbs,by=c("ctype","hotspot"),all.x=TRUE)
pancan.cbs2$prop=ifelse(is.na(pancan.cbs2$prop),0,pancan.cbs2$prop)
pancan.cbs2$perc=pancan.cbs2$prop*100

ggplot(pancan.cbs2,aes(x=hotspot,y=perc,fill=ctype))+geom_bar(stat="identity",colour="black",position = "dodge")+
  theme(axis.text.x = element_text(angle = 90, hjust = 1))+
  theme(text = element_text(size=20),axis.text.x = element_text(size=20),legend.position="none",panel.background =element_rect(fill = "white", color="black"))+
  theme(legend.position = "right")+
  scale_fill_brewer(palette = "Set1")
```

## Figure B

Mutation rate in overlaps between CBS hotspots and each cancer type

```
# Find the number of mutated samples at overlap regions of ctcf motif and ctcf peak
ctcf.motif=read.table("fimo_all.txt",sep="\t")
unique(ctcf.motif$V4-ctcf.motif$V3)+1 #19
```

```
## [1] 19
```

```
ctcf.motif=GRanges(seqnames=ctcf.motif$V2,IRanges(start=ctcf.motif$V3,end=ctcf.motif$V4),pval=ctcf.motif$V7,qval=ctcf.motif$V8,dir=ctcf.motif$V5,motif=ctcf.motif$V9)
length(ctcf.motif)
```

```
## [1] 1751592
```

```
ctcf.peak=import("CTCF.bw")
sum(ctcf.peak$score>0)
```

```
## [1] 1147323
```

```
z=findOverlaps(ctcf.peak,ctcf.motif) # 183666
ctcf.peak.ovl=ctcf.peak[queryHits(z)]
ctcf.peak.ovl=as.data.frame(ctcf.peak.ovl)
ctcf.peak.ovl=unique(ctcf.peak.ovl) # 157222
ctcf.motif.ovl=ctcf.motif[subjectHits(z)]
ctcf.motif.ovl=as.data.frame(ctcf.motif.ovl)
ctcf.motif.ovl=unique(ctcf.motif.ovl) # 125228
ctcf.motif.ovl=GRanges(seqnames=ctcf.motif.ovl$seqnames,IRanges(start=ctcf.motif.ovl$start,end=ctcf.motif.ovl$end),pval=ctcf.motif.ovl$pval,qval=ctcf.motif.ovl$qval,dir=ctcf.motif.ovl$dir,motif=ctcf.motif.ovl$motif)

# extend regions by 5bp
ctcf.motif.ovl=ctcf.motif.ovl+5

ovl=findOverlaps(maf.total2,ctcf.motif.ovl) # 16722
pancan.cbs=maf.total2[unique(queryHits(ovl))] # 15152
ind.cbs=split(pancan.cbs, pancan.cbs$ctype)
cbs.count=sapply(ind.cbs, function(x) {length(unique(x$sid))}) # number of samples per cancer type that overlapped with CBS hotspots
print(cbs.count)
```

```
##     aml    astr    blca   brain    brca     cll     crc gastric    hnsc 
##       2      11      20      43     164      17      21     179      27 
##    kich    kirc    lihc    luad    lusc    lymp    medu      ov    paad 
##       2      29      87      63      20      23      63       5      15 
##    prad    skcm    thca    ucec 
##      12      11       6      27
```

```
cbs.mut.count=sapply(ind.cbs, function(x) {length(x)}) # number of mutations per cancaer type that overlapped with CBS hotspots
print(cbs.mut.count)
```

```
##     aml    astr    blca   brain    brca     cll     crc gastric    hnsc 
##       4      12     609     218    1416      31     687    5295     382 
##    kich    kirc    lihc    luad    lusc    lymp    medu      ov    paad 
##       3     173    1221    2346     805     135     137      36     131 
##    prad    skcm    thca    ucec 
##      29     961      14     507
```

```
# calculate mutation rate
df2=data.frame(ctype=names(sample.count4),mut.rate=cbs.mut.count/(sum(width(ctcf.motif.ovl))*sample.count4))
df2=df2[which(!df2$ctype %in% names(sample.count4[which(sample.count4<10)])),] # 19
df2$ctype=as.character(df2$ctype)
df2$ctype=factor(df2$ctype,levels=cancer.type)

ggplot(df2,aes(x=ctype,y=mut.rate))+geom_bar(stat="identity",position=position_stack(reverse=TRUE),colour="black")+
  coord_flip()+
  theme(text = element_text(size=20),axis.text.x = element_text(size=20),legend.position="none",panel.background =element_rect(fill = "white", color="black"))
```
