# Supplementary material for: Mutation hotspots at CTCF binding sites coupled to chromosomal instability in gastrointestinal cancers
Source: Nat Commun. 2018 Apr 18;9:1520. doi: 10.1038/s41467-018-03828-2 (PMC5906695; doi:10.1038/s41467-018-03828-2)
Supplement: Supplementary file 8 — Supplementary Data 5 [file 41467_2018_3828_MOESM8_ESM.zip › Rmarkdowns/Figure 7/Figure7_CNV_breakpoints_rev.html]

Figure 7 - CNV breakpoints


# Figure 7 - CNV breakpoints

This is the Rmarkdown for Figure 7, which consists of 6 parts.

## Figure A-B

Distance from nearest breakpoint for CIN subtype

```
cnv<-read.delim('gastric_cnv_cns.txt', header=T, sep=" ")
```

Each start and each end is a different breakpoint.

```
head(cnv)
```

```
##   chromosome  start    end
## 1          1  10999  20991
## 2          1  20991 139896
## 3          1 139896 157882
## 4          1 157882 227638
## 5          1 227638 253698
## 6          1 253698 259712
##                                                                                                                                                                                                       gene
## 1                       AL627309.1,CICP27,DDX11L1,FAM138A,MIR1302-10,OR4F5,OR4G11P,OR4G4P,RNU6-1100P,RP11-34P13.13,RP11-34P13.14,RP11-34P13.15,RP11-34P13.16,RP11-34P13.7,RP11-34P13.8,RP11-34P13.9,WASH7P
## 2                       AL627309.1,CICP27,DDX11L1,FAM138A,MIR1302-10,OR4F5,OR4G11P,OR4G4P,RNU6-1100P,RP11-34P13.13,RP11-34P13.14,RP11-34P13.15,RP11-34P13.16,RP11-34P13.7,RP11-34P13.8,RP11-34P13.9,WASH7P
## 3                       AL627309.1,CICP27,DDX11L1,FAM138A,MIR1302-10,OR4F5,OR4G11P,OR4G4P,RNU6-1100P,RP11-34P13.13,RP11-34P13.14,RP11-34P13.15,RP11-34P13.16,RP11-34P13.7,RP11-34P13.8,RP11-34P13.9,WASH7P
## 4 AL627309.1,CICP27,DDX11L1,FAM138A,MIR1302-10,OR4F5,OR4G11P,OR4G4P,RNU6-1100P,RP11-34P13.13,RP11-34P13.14,RP11-34P13.15,RP11-34P13.16,RP11-34P13.7,RP11-34P13.8,RP11-34P13.9,WASH7P,AP006222.1,AP006222.2
## 5                                                                                                                                                                                    AP006222.1,AP006222.2
## 6                                                                                                                                                                                    AP006222.1,AP006222.2
##         log2 probes       sid
## 1  0.9422150     20 HK-pfg082
## 2  0.4673770    237 HK-pfg082
## 3 -0.0652516     36 HK-pfg082
## 4  0.1975410     40 HK-pfg082
## 5  0.6484330     52 HK-pfg082
## 6  0.2114650     12 HK-pfg082
```

```
cnv.filtered<-cnv[cnv$log2<log2(1.5/2)|cnv$log2>log2(2.5/2),] # 302413

subtype_classification <- read.delim("subtype_classification.txt", stringsAsFactors=FALSE)
CIN=subtype_classification[which(subtype_classification$Molecular.Subtype=="CIN"),"Sample.ID"] # 42

cnv.cin=cnv.filtered[which(cnv.filtered$sid %in% CIN),] # 80718
length(unique(cnv.cin$sid)) # 42
```

```
## [1] 42
```

```
cnv.cin$sid=factor(cnv.cin$sid)

# count number of CNVs in each CIN sample 
sample.bp.count=table(cnv.cin$sid)

bp1 <-with(cnv.cin, GRanges(chromosome, IRanges(start, start), sid=sid))
bp2 <-with(cnv.cin, GRanges(chromosome, IRanges(end, end), sid=sid))  
bp <- c(bp1,bp2)
bp=as.data.frame(bp)
bp=unique(bp) # 130020
bp=with(bp, GRanges(seqnames, IRanges(start,end), sid=sid))
seqlevelsStyle(bp)<- "UCSC"

maf.gastric <- maf.to.granges('gastric_RF_prefiltered.MAF')
```

```
## [1] ">> Reading compact MAF ..."
```

```
maf.gastric=maf.gastric[-which(seqnames(maf.gastric)=="chrY")] # 4139879
maf.gastric=maf.gastric[-which(maf.gastric$sid %in% c("tan2001206", "tan20021007", "tan980319", "tan2000986", "tan980436"))] # 4116299
maf.cin=maf.gastric[maf.gastric$sid%in%CIN] # 729958
maf.cin$sid=factor(maf.cin$sid) # 41

hotspot <- read.delim("LRmodel_hotspot_nonMSI_prefiltered-5_corrected.tsv", stringsAsFactors=FALSE)
hotspot$mut_region=rownames(hotspot)
hotspot=GRanges(seqnames=hotspot$chrom,IRanges(start=hotspot$start,end=hotspot$end),mut_region=hotspot$mut_region,pval=hotspot$pval,fdr=hotspot$fdr)
hotspot=hotspot[which(hotspot$pval<(0.01/2533374732))]
hotspot=reduce(hotspot)
hotspot$hotspot=c(1:length(hotspot))

# Identify CTCF hotspot
roi.ctcf <- bed.to.granges("ctcf_motif_union.bed")
ctcf.hotspot=hotspot[unique(queryHits(findOverlaps(hotspot,roi.ctcf)))] # 11
other.hotspot=hotspot[-unique(queryHits(findOverlaps(hotspot,roi.ctcf)))] # 24

# Identify all CBS with mutations or flanking mutations
mut.ctcf=roi.ctcf[unique(queryHits(findOverlaps(roi.ctcf+5, maf.cin)))] # 871

## Identify all boundary CBS with mutations or flanking mutations
ctcf.boundary<-bed.to.granges("ctcf_union_boundary.bed")
mut.boundary=ctcf.boundary[unique(queryHits(findOverlaps(ctcf.boundary+5, maf.cin)))] # 589

## Identify all nonboundary CBS with mutations or flanking mutations
ctcf.non.boundary<-bed.to.granges("ctcf_union_nonboundary.bed")
mut.non.boundary=ctcf.non.boundary[unique(queryHits(findOverlaps(ctcf.non.boundary+5, maf.cin)))] # 282

## Identify all control CBS with mutations or flanking mutations
ctcf.control <- read.table("ctcf_controls.txt",header=T)
ctcf.control <- with(ctcf.control, GRanges(seqnames, IRanges(start, end)))
mut.control=ctcf.control[unique(queryHits(findOverlaps(ctcf.control+5, maf.cin)))] # 125

###
### calclulate distance to the nearest breakpoint from CBS
###
mut.samples <- function(roi, bp, maf) {
  z=findOverlaps(maf,roi)
  maf.roi=maf[queryHits(z)]
  sid=unique(maf.roi$sid)
  sid=as.character(sid)
  return(sid)
}

# find the distance to the nearest breakpoint in each sample
find.nearest.bp <- function(roi, bp.ind) {
  # could be done on all roils in parallel)
  nearest.bp=lapply(bp.ind,  function(x) {
    dis=distanceToNearest(roi, x)
    dis.v=dis@elementMetadata$distance
    v=rep(-1,length(roi))
    v[queryHits(dis)]=dis.v
    v
  }) 
  nearest.bp=do.call(cbind, nearest.bp)
  # for samples with no breakpoints in the same chromosome, set the distance to roi as the max distance for that site
  nearest.bp=apply(nearest.bp,2, function(x) {
    i=which(x==-1)
    x[i]=max(x)
    x
  })
  return(nearest.bp)
}

bp.ind<-split(bp, bp$sid)

## ctcf hotspots
mut.samples.hs <- lapply(ctcf.hotspot, function(x) {mut.samples(x, bp, maf.cin)})
nearest.bp.hs<- find.nearest.bp(ctcf.hotspot, bp.ind)
nearest.bp2 <-lapply(1:nrow(nearest.bp.hs), function(i){
  sid=mut.samples.hs[[i]]
  mut.dist=nearest.bp.hs[i,colnames(nearest.bp.hs)%in%sid]
  nonmut.dist=nearest.bp.hs[i,!colnames(nearest.bp.hs)%in%sid]
  list(mut.dist, nonmut.dist)
})
dist.hs.mut=unlist(lapply(nearest.bp2, function(x){x[[1]]}))
dist.hs.nonmut=unlist(lapply(nearest.bp2 , function(x){x[[2]]}))  
wilcox.test(dist.hs.mut, dist.hs.nonmut) # p= 0.0018
```

```
## 
##  Wilcoxon rank sum test with continuity correction
## 
## data:  dist.hs.mut and dist.hs.nonmut
## W = 5763.5, p-value = 0.001844
## alternative hypothesis: true location shift is not equal to 0
```

```
boxplot(log(dist.hs.mut), log(dist.hs.nonmut), ylab="distance to the nearest breakpoint log (bp)", names=c("mutated", "non-mutated"), outline=F, notch=T)
```

```
### other hotspots
mut.samples.hs <- lapply(other.hotspot, function(x) {mut.samples(x, bp, maf.cin)})
nearest.bp.hs<- find.nearest.bp(other.hotspot, bp.ind)
nearest.bp2 <-lapply(1:nrow(nearest.bp.hs), function(i){
  sid=mut.samples.hs[[i]]
  mut.dist=nearest.bp.hs[i,colnames(nearest.bp.hs)%in%sid]
  nonmut.dist=nearest.bp.hs[i,!colnames(nearest.bp.hs)%in%sid]
  list(mut.dist, nonmut.dist)
})
dist.hs2.mut=unlist(lapply(nearest.bp2, function(x){x[[1]]}))
dist.hs2.nonmut=unlist(lapply(nearest.bp2 , function(x){x[[2]]}))  
wilcox.test(dist.hs2.mut, dist.hs2.nonmut) # p= 0.5311
```

```
## 
##  Wilcoxon rank sum test with continuity correction
## 
## data:  dist.hs2.mut and dist.hs2.nonmut
## W = 20003, p-value = 0.5311
## alternative hypothesis: true location shift is not equal to 0
```

```
boxplot(log(dist.hs2.mut), log(dist.hs2.nonmut), ylab="distance to the nearest breakpoint log (bp)", names=c("mutated", "non-mutated"), outline=F, notch=T)
```

```
### boundary CBS
mut.samples.boundary <- lapply(mut.boundary+5, function(x) {mut.samples(x, bp, maf.cin)})
nearest.bp.boundary<- find.nearest.bp(mut.boundary, bp.ind)
nearest.bp2 <-lapply(1:nrow(nearest.bp.boundary), function(i){
  sid=mut.samples.boundary[[i]]
  mut.dist=nearest.bp.boundary[i,colnames(nearest.bp.boundary)%in%sid]
  nonmut.dist=nearest.bp.boundary[i,!colnames(nearest.bp.boundary)%in%sid]
  list(mut.dist, nonmut.dist)
})
dist.boundary.mut=unlist(lapply(nearest.bp2, function(x){x[[1]]}))
dist.boundary.nonmut=unlist(lapply(nearest.bp2 , function(x){x[[2]]}))  
wilcox.test(dist.boundary.mut, dist.boundary.nonmut) # p-value = 5.72e-16
```

```
## 
##  Wilcoxon rank sum test with continuity correction
## 
## data:  dist.boundary.mut and dist.boundary.nonmut
## W = 6629300, p-value = 5.72e-16
## alternative hypothesis: true location shift is not equal to 0
```

```
quantile(dist.boundary.mut)
```

```
##        0%       25%       50%       75%      100% 
##        15    245446   1103124   4171739 100183526
```

```
quantile(dist.boundary.nonmut)
```

```
##        0%       25%       50%       75%      100% 
##         0    526534   2391018   6925464 113869473
```

```
boxplot(log(dist.boundary.mut), log(dist.boundary.nonmut), ylab="distance to the nearest breakpoint log (bp)", names=c("mutated", "non-mutated"), outline=F, notch=T)
```

```
### non boundary CBS
mut.samples.boundary <- lapply(mut.non.boundary+5, function(x) {mut.samples(x, bp, maf.cin)})
nearest.bp.boundary<- find.nearest.bp(mut.non.boundary, bp.ind)
nearest.bp2 <-lapply(1:nrow(nearest.bp.boundary), function(i){
  sid=mut.samples.boundary[[i]]
  mut.dist=nearest.bp.boundary[i,colnames(nearest.bp.boundary)%in%sid]
  nonmut.dist=nearest.bp.boundary[i,!colnames(nearest.bp.boundary)%in%sid]
  list(mut.dist, nonmut.dist)
})
dist.nonboundary.mut=unlist(lapply(nearest.bp2, function(x){x[[1]]}))
dist.nonboundary.nonmut=unlist(lapply(nearest.bp2 , function(x){x[[2]]}))  
wilcox.test(dist.nonboundary.mut, dist.nonboundary.nonmut) # p-value = 0.0002277
```

```
## 
##  Wilcoxon rank sum test with continuity correction
## 
## data:  dist.nonboundary.mut and dist.nonboundary.nonmut
## W = 1674100, p-value = 0.0002277
## alternative hypothesis: true location shift is not equal to 0
```

```
quantile(dist.nonboundary.mut)
```

```
##         0%        25%        50%        75%       100% 
##      268.0   311582.5  1722544.0  4627776.2 37483564.0
```

```
quantile(dist.nonboundary.nonmut)
```

```
##          0%         25%         50%         75%        100% 
##         0.0    546682.5   2369807.0   6706649.0 107700451.0
```

```
boxplot(log(dist.nonboundary.mut), log(dist.nonboundary.nonmut), ylab="distance to the nearest breakpoint log (bp)", names=c("mutated", "non-mutated"), outline=F, notch=T)
```

## Figure A

Distance from nearest breakpoint at CTCF hotspots and other hotspots

```
distance=data.frame(dist=c(dist.hs.mut,dist.hs.nonmut,dist.hs2.mut,dist.hs2.nonmut,dist.boundary.mut,dist.boundary.nonmut,dist.nonboundary.mut,dist.nonboundary.nonmut),
                    mut=c(rep("mut",length(dist.hs.mut)), rep("nonmut", length(dist.hs.nonmut)),rep("mut",length(dist.hs2.mut)), rep("nonmut", length(dist.hs2.nonmut)),rep("mut",length(dist.boundary.mut)), rep("nonmut", length(dist.boundary.nonmut)),rep("mut",length(dist.nonboundary.mut)), rep("nonmut", length(dist.nonboundary.nonmut))),
                    type=c(rep("CTCF.hotspots", length(dist.hs.mut)+length(dist.hs.nonmut)),rep("other.hotspots", length(dist.hs2.mut)+length(dist.hs2.nonmut)), rep("boundary.CBS", length(dist.boundary.mut)+length(dist.boundary.nonmut)), rep("non-boundary.CBS", length(dist.nonboundary.mut)+length(dist.nonboundary.nonmut))))
# replace 0 with 1 for plot on log scale
distance[distance$dist==0,"dist"]=1

df1=distance[which(distance$type %in% c("CTCF.hotspots","other.hotspots")),] # 1478
df2=distance[which(distance$type %in% c("boundary.CBS","non-boundary.CBS")),] # 36582

# re-arrange the x-axis
distance$type <- factor(distance$type,levels = c("CTCF.hotspots","other.hotspots", "boundary.CBS","non-boundary.CBS"),ordered = TRUE)
p0<-ggplot(aes(y = log10(dist), x = type, fill = mut), data = distance) + 
  geom_boxplot(outlier.shape=NA,notch=TRUE)+  
  ylab(paste("Distance from nearest breakpoint",sep=""))+ scale_fill_manual(values=c("#E69F00", "#999999"))+
  theme(panel.grid.major = element_blank(), panel.grid.minor = element_blank(), panel.background = element_blank(), axis.line = element_line(colour="black"))
p1<-p0+coord_cartesian(ylim = c(3.6,8.5))
p1
```

```
df1$type <- factor(df1$type,levels = c("CTCF.hotspots","other.hotspots"),ordered = TRUE)
p0<-ggplot(aes(y = log10(dist), x = type, fill = mut), data = df1) + 
  geom_boxplot(outlier.shape=NA,notch=TRUE)+  
  ylab(paste("Distance from nearest breakpoint",sep=""))+ scale_fill_manual(values=c("#E69F00", "#999999"))+
  theme(panel.grid.major = element_blank(), panel.grid.minor = element_blank(), panel.background = element_blank(), axis.line = element_line(colour="black"))
p1<-p0+coord_cartesian(ylim = c(3.6,8.5))
print(p1)
```

## Figure B

Distance from nearest breakpoint at boundary CBS vs non-boundary CBS

```
df2$type <- factor(df2$type,levels = c("boundary.CBS","non-boundary.CBS"),ordered = TRUE)
p0<-ggplot(aes(y = log10(dist), x = type, fill = mut), data = df2) + 
  geom_boxplot(outlier.shape=NA,notch=TRUE)+  
  ylab(paste("Distance from nearest breakpoint",sep=""))+ scale_fill_manual(values=c("#E69F00", "#999999"))+
  theme(panel.grid.major = element_blank(), panel.grid.minor = element_blank(), panel.background = element_blank(), axis.line = element_line(colour="black"))
p1<-p0+coord_cartesian(ylim = c(3.6,8.5))
print(p1)
```

## Figure C

Mutation rate vs CNV breakpoints

```
chrOrder<-c(paste("chr",1:22,sep=""),"chrX")
seqi = seqinfo(Hsapiens)[seqnames(Hsapiens)[1:23]]
seqnames=seqnames(seqinfo(Hsapiens))[1:23]

## Tile genome into equally sized bins
binsize = 1000*1e3
cores=4
genome.bins <- tileGenome(seqi, tilewidth=binsize, cut.last.tile.in.chrom=TRUE)
nbin = length(genome.bins) # 3053 1mb bins; 30376 100kb bins
names(genome.bins)=paste("n", seq(1:nbin), sep="")
genome.bins.grl<- split(genome.bins, names(genome.bins))

## mask regions
mappability=import("wgEncodeCrgMapabilityAlign75mer.bigWig")
## define reads that can map to more than 1 genomic location as non-mappable
nonmappable=mappability[mappability$score<1,]
# convert zero-based coordinates to one-based coordinates
nonmappable=shift(nonmappable,1)
nonmappable= reduce(nonmappable)
nonmappable=nonmappable[seqnames(nonmappable) %in% seqnames(seqi)]
seqlevels(nonmappable)=as.character(unique(seqnames(nonmappable)))

# trim ends of each chromosome that are N's
mappability.grl=split(mappability, seqnames(mappability))
ranges=lapply(mappability.grl, range)
ranges=GRangesList(ranges)
ranges=unlist(ranges)
extrSeq=Views(Hsapiens,ranges) # check that all N's at chromosome ends are trimmed
# define chromosome ends to be masked
ranges=ranges[seqnames(ranges) %in% seqnames(seqi)] # 2441960
seqlevels(ranges)=as.character(unique(seqnames(ranges)))
end1=GRanges(seqnames(ranges), IRanges(1, start(ranges)-1))
end2=GRanges(seqnames(ranges), IRanges(end(ranges)+1, seqlengths(seqi[as.character(seqnames(ranges))])))

## mask CDS and ig loci
roi.cds <- bed.to.granges('Ensembl75.CDS.bed')
roi.cds.ext <- reduce(roi.cds + 5) # extend each region with +/- 5 bases and get all non-overlapping regions
# immunoglobulin loci
ig.loci <- bed.to.granges('ig_loci.bed')
ig.loci <- reduce(ig.loci + 10**5) # extend each region with 100kb and get all non-overlapping regions
# combine mask regions
mask.regions=reduce(trim(c(ig.loci,roi.cds.ext,nonmappable,end1,end2))) 

genome.bins.grl.masked= subtract.regions.from.roi(genome.bins.grl, mask.regions, cores=cores)
genome.bins.grl.masked=genome.bins.grl.masked[sum(width(genome.bins.grl.masked))>=250000] # 2924
mean(sum(width(genome.bins.grl.masked))) # 904014.2
median(sum(width(genome.bins.grl.masked))) # 927924.5

## Use only CIN subtypes
cnv<-read.delim('gastric_cnv_cns.txt', header=T, sep=" ") # 956516
cnv.filtered<-cnv[cnv$log2<log2(1.5/2)|cnv$log2>log2(2.5/2),] # 302413

subtype_classification <- read.delim("subtype_classification.txt", stringsAsFactors=FALSE)
CIN=subtype_classification[which(subtype_classification$Molecular.Subtype=="CIN"),"Sample.ID"] # 42

cnv.cin=cnv.filtered[which(cnv.filtered$sid %in% CIN),] # unfiltered:215200; filtered:80718
length(unique(cnv.cin$sid)) # 42
cnv.cin$sid=factor(cnv.cin$sid)

# count number of CNVs in each CIN sample 
sample.bp.count=table(cnv.cin$sid)

# get CNV break points
bp1 <-with(cnv.cin, GRanges(chromosome, IRanges(start, start), sid=sid))
bp2 <-with(cnv.cin, GRanges(chromosome, IRanges(end, end), sid=sid))  
bp <- c(bp1,bp2)
bp=as.data.frame(bp)
bp=unique(bp) # unfiltered:245545; filtered:130020
bp=with(bp, GRanges(seqnames, IRanges(start,end), sid=sid))
seqlevelsStyle(bp)<- "UCSC"

# get breakpoint coverage
## calculate mutation rate for each bin
genome.bins.length= sum(width(genome.bins.grl.masked))
genome.bins.length=data.frame(name=names(genome.bins.length),length=genome.bins.length)
bp.ovl <- findOverlaps(genome.bins.grl.masked, bp)
bp.ovl.m = as.matrix(bp.ovl)
genome.bins.mutcount=tapply(bp.ovl.m[,2], names(genome.bins.grl.masked)[bp.ovl.m[,1]], function(s) length(s))
genome.bins.mutcount=data.frame(name=names(genome.bins.mutcount),mutcount=genome.bins.mutcount)
genome.bins.mutrate=merge(genome.bins.length,genome.bins.mutcount,all=T)
genome.bins.mutrate[is.na(genome.bins.mutrate)]=0
genome.bins.mutrate$mut.rate=genome.bins.mutrate$mutcount/genome.bins.mutrate$length

## get mutations for CIN samples
maf.gastric <- maf.to.granges('gastric_RF_prefiltered.MAF') # 4143709
maf.cin=maf.gastric[maf.gastric$sid%in%CIN]
maf.cin=maf.cin[-which(maf.cin$sid %in% c("tan2001206", "tan20021007", "tan980319", "tan2000986", "tan980436"))]
maf.cin$sid=factor(maf.cin$sid)
nind=length(unique(maf.cin$sid))

# Identify CTCF hotspot
roi.ctcf <- bed.to.granges("ctcf_motif_union.bed")
roi.ctcf=roi.ctcf+5
length(unique(queryHits(findOverlaps(maf.cin,roi.ctcf)))) # 912 CBS mutations in CIN samples

# bin all genome bins according to breakpoint rate
bprate.bin=.bincode(genome.bins.mutrate$mut.rate, quantile(genome.bins.mutrate$mut.rate, seq(0,1,0.2)))
genome.bins.mutrate=cbind(genome.bins.mutrate, bprate.bin)
genome.bins.ls=split(genome.bins.mutrate, bprate.bin)
# calulate the CBS mutation rate in each bin
out=lapply(names(genome.bins.ls), function(x) {
 ranges= unlist(genome.bins.grl.masked[as.character(genome.bins.ls[[x]]$name)])
 ctcf.in.range=reduce(roi.ctcf[queryHits(findOverlaps(roi.ctcf,ranges))])
 cbs.mutrate=mutrate=mut.count=NULL
 for (i in unique(as.character(maf.cin$sid))){
   print(i)
   mut.count=c(mut.count,length(unique(queryHits(findOverlaps(maf.cin[which(maf.cin$sid==i)],ctcf.in.range)))))
   cbs.mutrate=c(cbs.mutrate,length(unique(queryHits(findOverlaps(maf.cin[which(maf.cin$sid==i)],ctcf.in.range))))/sum(width(ctcf.in.range)))
   mutrate=c(mutrate,length(unique(queryHits(findOverlaps(maf.cin[which(maf.cin$sid==i)],ranges))))/sum(width(ranges)))
 }
 data.frame(mut.count,cbs.mutrate,mutrate,bin=x)
})

output=do.call(rbind, out) # 210

# save summarized output to table
write.table(output,"fig7c.table.txt",sep="\t",col.names=TRUE,row.names=TRUE)
```

Read in summarized table

```
output=read.table("fig7c.table.txt",header=TRUE)
```

```
for (i in 1:5){
  print(sum(output[which(output$bin==i),"mut.count"]))
}
```

```
## [1] 104
## [1] 163
## [1] 195
## [1] 213
## [1] 221
```

```
out=output[,c(2:4)]
colnames(out)=c("CBS","bg","bin")
out$bin=factor(out$bin)
out2=melt(out)
```

```
## Using bin as id variables
```

```
colnames(out2)=c("bin","type","mutrate")
out2.mean=aggregate(mutrate~bin+type,out2,mean)
out2.se=aggregate(mutrate~bin+type,out2,FUN=function(x) sd(x)/sqrt(length(x)))
df=out2.mean
colnames(df)[3]="mean.mutrate"
df$se.mutrate=out2.se$mutrate
df$type=as.character(df$type)
df$type=factor(df$type,levels=c("bg","CBS"))

out3=out
out3$rate=out$CBS/out$bg
out3.mean=aggregate(rate~bin,out3,mean)
out3.se=aggregate(rate~bin,out3,FUN=function(x) sd(x)/sqrt(length(x)))
df2=out3.mean
colnames(df2)[2]="mean.mutrate"
df2$se.mutrate=out3.se$rate

print(ggplot(df,aes(x=bin,y=mean.mutrate,fill=type))+
        geom_bar(position=position_dodge(),stat="identity")+
        geom_errorbar(aes(ymin=mean.mutrate-se.mutrate,ymax=mean.mutrate+se.mutrate),width=.1,position=position_dodge(0.9))+
        ggtitle(paste("Association between CNV and mutation rate"))+theme(axis.text.x=element_text(size=30,angle=90,vjust=1), axis.text.y=element_text(size=20))+
        labs(x = "CNV breakpoint density bins", y = "mutation rate")+
        scale_fill_manual(values=c("grey","black"))+
        theme(panel.grid.major = element_blank(), panel.grid.minor = element_blank(), panel.background = element_blank(), axis.line = element_line(colour="black")))
```

## Figure D

CBS mutation rate/overall mutation rate vs CNV breakpoints

```
print(ggplot(df2,aes(x=bin,y=mean.mutrate))+geom_bar(position=position_dodge(),stat="identity")+
        geom_errorbar(aes(ymin=mean.mutrate-se.mutrate,ymax=mean.mutrate+se.mutrate),width=.1,position=position_dodge(0.9))+
        ggtitle(paste("Association between CNV and CBS mutation rate"))+theme(axis.text.x=element_text(size=30,angle=90,vjust=1), axis.text.y=element_text(size=20))+
        labs(x = "CNV breakpoint density bins", y = "CBS mutation rate/overall mutation rate")+
        scale_fill_manual(values=c("black"))+
        theme(panel.grid.major = element_blank(), panel.grid.minor = element_blank(), panel.background = element_blank(), axis.line = element_line(colour="black")))
```

```
summary(lm(rate~factor(bin),out3))
```

```
## 
## Call:
## lm(formula = rate ~ factor(bin), data = out3)
## 
## Residuals:
##     Min      1Q  Median      3Q     Max 
## -2.6876 -2.0175 -0.3534  1.4277  6.4418 
## 
## Coefficients:
##              Estimate Std. Error t value Pr(>|t|)    
## (Intercept)   2.01750    0.32477   6.212 2.98e-09 ***
## factor(bin)2  0.07159    0.45929   0.156    0.876    
## factor(bin)3  0.26124    0.45929   0.569    0.570    
## factor(bin)4  0.67010    0.45929   1.459    0.146    
## factor(bin)5  0.64622    0.45929   1.407    0.161    
## ---
## Signif. codes:  0 '***' 0.001 '**' 0.01 '*' 0.05 '.' 0.1 ' ' 1
## 
## Residual standard error: 2.08 on 200 degrees of freedom
## Multiple R-squared:  0.01843,    Adjusted R-squared:  -0.001202 
## F-statistic: 0.9388 on 4 and 200 DF,  p-value: 0.4425
```

```
# Coefficients:
#              Estimate Std. Error t value Pr(>|t|)    
# (Intercept)   2.01750    0.32477   6.212 2.98e-09 ***
# factor(bin)2  0.07159    0.45929   0.156    0.876    
# factor(bin)3  0.26124    0.45929   0.569    0.570    
# factor(bin)4  0.67010    0.45929   1.459    0.146    
# factor(bin)5  0.64622    0.45929   1.407    0.161   

df2$bin=as.numeric(as.character(df2$bin))
summary(lm(mean.mutrate~bin,df2))
```

```
## 
## Call:
## lm(formula = mean.mutrate ~ bin, data = df2)
## 
## Residuals:
##        1        2        3        4        5 
##  0.04836 -0.06914 -0.06859  0.15118 -0.06180 
## 
## Coefficients:
##             Estimate Std. Error t value Pr(>|t|)    
## (Intercept)  1.78004    0.11881  14.982 0.000645 ***
## bin          0.18910    0.03582   5.279 0.013258 *  
## ---
## Signif. codes:  0 '***' 0.001 '**' 0.01 '*' 0.05 '.' 0.1 ' ' 1
## 
## Residual standard error: 0.1133 on 3 degrees of freedom
## Multiple R-squared:  0.9028, Adjusted R-squared:  0.8704 
## F-statistic: 27.86 on 1 and 3 DF,  p-value: 0.01326
```

```
# Coefficients:
#             Estimate Std. Error t value Pr(>|t|)    
# (Intercept)  1.78004    0.11881  14.982 0.000645 ***
# bin          0.18910    0.03582   5.279 0.013258 *  

summary(lm(cbs.mutrate~factor(bin),output))
```

```
## 
## Call:
## lm(formula = cbs.mutrate ~ factor(bin), data = output)
## 
## Residuals:
##        Min         1Q     Median         3Q        Max 
## -2.674e-05 -1.620e-05 -1.047e-05  5.300e-06  1.525e-04 
## 
## Coefficients:
##                Estimate Std. Error t value Pr(>|t|)    
## (Intercept)   2.674e-05  4.478e-06   5.972 1.05e-08 ***
## factor(bin)2 -6.174e-06  6.332e-06  -0.975   0.3308    
## factor(bin)3 -7.483e-06  6.332e-06  -1.182   0.2387    
## factor(bin)4 -9.720e-06  6.332e-06  -1.535   0.1264    
## factor(bin)5 -1.120e-05  6.332e-06  -1.769   0.0784 .  
## ---
## Signif. codes:  0 '***' 0.001 '**' 0.01 '*' 0.05 '.' 0.1 ' ' 1
## 
## Residual standard error: 2.867e-05 on 200 degrees of freedom
## Multiple R-squared:  0.01835,    Adjusted R-squared:  -0.001287 
## F-statistic: 0.9344 on 4 and 200 DF,  p-value: 0.445
```

```
# Coefficients:
#                Estimate Std. Error t value Pr(>|t|)    
# (Intercept)   2.674e-05  4.478e-06   5.972 1.05e-08 ***
# factor(bin)2 -6.174e-06  6.332e-06  -0.975   0.3308    
# factor(bin)3 -7.483e-06  6.332e-06  -1.182   0.2387    
# factor(bin)4 -9.720e-06  6.332e-06  -1.535   0.1264    
# factor(bin)5 -1.120e-05  6.332e-06  -1.769   0.0784 . 

output$bin=as.numeric(as.character(output$bin))
summary(lm(mutrate~factor(bin),data=output))
```

```
## 
## Call:
## lm(formula = mutrate ~ factor(bin), data = output)
## 
## Residuals:
##        Min         1Q     Median         3Q        Max 
## -9.115e-06 -3.421e-06 -1.548e-06  1.056e-06  2.995e-05 
## 
## Coefficients:
##                Estimate Std. Error t value Pr(>|t|)    
## (Intercept)   1.036e-05  9.678e-07  10.706  < 2e-16 ***
## factor(bin)2 -2.779e-06  1.369e-06  -2.031 0.043599 *  
## factor(bin)3 -4.163e-06  1.369e-06  -3.042 0.002667 ** 
## factor(bin)4 -5.389e-06  1.369e-06  -3.938 0.000114 ***
## factor(bin)5 -5.883e-06  1.369e-06  -4.298 2.69e-05 ***
## ---
## Signif. codes:  0 '***' 0.001 '**' 0.01 '*' 0.05 '.' 0.1 ' ' 1
## 
## Residual standard error: 6.197e-06 on 200 degrees of freedom
## Multiple R-squared:  0.1066, Adjusted R-squared:  0.08875 
## F-statistic: 5.967 on 4 and 200 DF,  p-value: 0.0001481
```

```
#                Estimate Std. Error t value Pr(>|t|)    
# (Intercept)   1.036e-05  9.678e-07  10.706  < 2e-16 ***
# factor(bin)2 -2.779e-06  1.369e-06  -2.031 0.043599 *  
# factor(bin)3 -4.163e-06  1.369e-06  -3.042 0.002667 ** 
# factor(bin)4 -5.389e-06  1.369e-06  -3.938 0.000114 ***
# factor(bin)5 -5.883e-06  1.369e-06  -4.298 2.69e-05 ***
```

## Figure E

VAF distribution of somatic mutations

```
###
### Identify the 11 CBS hotspots
###
hotspot=read.delim("LRmodel_hotspot_nonMSI_prefiltered-5_corrected.tsv", header=T, stringsAsFactors = F)
hotspot=GRanges(seqnames=hotspot$chrom,IRanges(start=hotspot$start,end=hotspot$end),pval=hotspot$pval,fdr=hotspot$fdr)
hotspot=hotspot[which(hotspot$pval<(0.01/2533374732))]
hotspot=reduce(hotspot)
hotspot$hotspot=paste("hotspot",c(1:length(hotspot)),sep="")

roi.ctcf <- bed.to.granges("ctcf_motif_union.bed") # 47453
ctcf.hotspot=hotspot[unique(queryHits(findOverlaps(hotspot,roi.ctcf)))] #11

ctcf.hotspot$hotspot=paste("cbs",c(1:length(ctcf.hotspot)),sep="")

###
### Identify all mutations in these 11 CBS hotspots
###
maf.gastric <- maf.to.granges('gastric_RF_prefiltered.MAF') # 4143709
```

```
## [1] ">> Reading compact MAF ..."
```

```
maf.gastric=maf.gastric[-which(maf.gastric$sid %in% c("tan2001206", "tan20021007", "tan980319", "tan2000986", "tan980436"))] # 4119812
maf.gastric=maf.gastric[-which(as.character(seqnames(maf.gastric))=="chrY")] # 4116299

z=findOverlaps(maf.gastric,ctcf.hotspot) # 91
maf=maf.gastric[queryHits(z)] # 48 unique samples, 91
maf$hotspot=ctcf.hotspot[subjectHits(z)]$hotspot

samples=unique(as.character(maf$sid))
maf.gastric$sid=as.character(maf.gastric$sid)
maf.gastric2=as.data.frame(maf.gastric)
maf.gastric2$seqnames=as.character(maf.gastric2$seqnames)
maf.gastric2$seqnames=substr(maf.gastric2$seqnames,4,nchar(maf.gastric2$seqnames))
maf.gastric2=with(maf.gastric2,GRanges(seqnames,IRanges(start,end),sid=sid)) # 4116299
```

```
###
### CNV filter
###
cnv<-read.delim('gastric_cnv_cns.txt', header=T, sep=" ") #956516
cnv.filtered<-cnv[cnv$log2>log2(1.5/2) & cnv$log2<log2(2.5/2),] # diploid, 654103
cnv.filtered=with(cnv.filtered,GRanges(chromosome,IRanges(start,end),sid=sid,log2=log2))
cnv.not.diploid=cnv[cnv$log2<log2(1.5/2) | cnv$log2>log2(2.5/2),] #not diploid
cnv.not.diploid=with(cnv.not.diploid,GRanges(chromosome,IRanges(start,end),sid=sid,log2=log2)) #302413
cnv.not.diploid=cnv.not.diploid[which(seqnames(cnv.not.diploid)!="Y")] #296649
seqlevelsStyle(cnv.not.diploid)<- "UCSC"

cnv.ind<-split(cnv.filtered, cnv.filtered$sid)
cnv.not.ind<-split(cnv.not.diploid, cnv.not.diploid$sid)

cnv.width=data.frame(samples=names(cnv.ind),width=numeric(length(cnv.ind)))
for (i in 1:nrow(cnv.width)){
  cnv.width$width[i]=sum(as.numeric(width(cnv.ind[[as.character(cnv.width$samples[i])]])))
} # some samples sum of width not close to genomic range, hence have to define diploid regions differently

# define new diploid regions
chrOrder<-c(paste("chr",1:22,sep=""),"chrX")
seqi = seqinfo(Hsapiens)[seqnames(Hsapiens)[1:23]]
genome=GRanges(seqnames(seqi),IRanges(1,as.numeric(seqlengths(seqi))))

## mask regions - non-mappable, end1, end2 and cnv non-diploid
mappability=import("wgEncodeCrgMapabilityAlign75mer.bigWig")
## define reads that can map to more than 1 genomic location as non-mappable
nonmappable=mappability[mappability$score<1,]
# convert zero-based coordinates to one-based coordinates
nonmappable=shift(nonmappable,1)
nonmappable= reduce(nonmappable)
nonmappable=nonmappable[seqnames(nonmappable) %in% seqnames(seqi)]
seqlevels(nonmappable)=as.character(unique(seqnames(nonmappable)))

# trim ends of each chromosome that are N's
mappability.grl=split(mappability, seqnames(mappability))
ranges=lapply(mappability.grl, range)
ranges=GRangesList(ranges)
ranges=unlist(ranges)
extrSeq=Views(Hsapiens,ranges) # check that all N's at chromosome ends are trimmed
# define chromosome ends to be masked
ranges=ranges[seqnames(ranges) %in% seqnames(seqi)] #2441960
seqlevels(ranges)=as.character(unique(seqnames(ranges)))
end1=GRanges(seqnames(ranges), IRanges(1, start(ranges)-1))
end2=GRanges(seqnames(ranges), IRanges(end(ranges)+1, seqlengths(seqi[as.character(seqnames(ranges))])))

cores=4
diploid=mclapply(names(cnv.not.ind),FUN=function(x){
  print(x)
  cnv.remove=cnv.not.ind[[x]]
  cnv.remove=with(cnv.remove,GRanges(seqnames,IRanges(start,end)))
  mask.regions=reduce(trim(c(nonmappable,end1,end2,cnv.remove)))
  genome.masked=subtract.regions.from.roi(genome,mask.regions,cores=cores)
  genome.masked
},mc.cores=10)
names(diploid)=names(cnv.not.ind)

cnv.width2=data.frame(samples=names(diploid),width=numeric(length(diploid)))
for (i in 1:nrow(cnv.width2)){
  cnv.width2$width[i]=sum(as.numeric(width(diploid[[as.character(cnv.width2$samples[i])]])))
}

samples=samples[which(samples %in% names(diploid))] #40

seqlevelsStyle(maf.gastric2)="UCSC"
###
### Extract allele frequency from mutect files of all mutations in all samples with mutations in the 11 CBS hotspots
###
split1=mclapply(samples[c(1:4)],FUN=function(x){
  print(x)
  vcf=readVcf(paste(x,"-mutect.vcf.gz",sep=""),"hg19")
  mut=maf.gastric2[which(maf.gastric2$sid==x)]
  t=findOverlaps(mut,diploid[[x]])
  mut=mut[unique(queryHits(t))]
  gr=rowRanges(vcf)[which(rowRanges(vcf)$FILTER=="PASS")]
  seqlevelsStyle(gr)="UCSC"
  gen=as.data.frame(geno(vcf)$FREQ)[which(rowRanges(vcf)$FILTER=="PASS"),]
  t=findOverlaps(mut,gr)
  print(length(t))
  af=gen[subjectHits(t),]
  af=af[,which(grepl("-T",colnames(af)))]
  af=unlist(af)
  af=as.data.frame(af)
  af$seqnames=as.character(seqnames(gr[subjectHits(t)]))
  af$pos=start(gr[subjectHits(t)])
  af$sid=x
  af
},mc.cores=5)
names(split1)=samples[c(1:4)]
saveRDS(split1,file="split1_violin_v2.RDS")

split2=mclapply(samples[c(7:11)],FUN=function(x){
  print(x)
  vcf=readVcf(paste(x,"-mutect.vcf.gz",sep=""),"hg19")
  mut=maf.gastric2[which(maf.gastric2$sid==x)]
  t=findOverlaps(mut,diploid[[x]])
  mut=mut[unique(queryHits(t))]
  gr=rowRanges(vcf)[which(rowRanges(vcf)$FILTER=="PASS")]
  seqlevelsStyle(gr)="UCSC"
  gen=as.data.frame(geno(vcf)$FREQ)[which(rowRanges(vcf)$FILTER=="PASS"),]
  t=findOverlaps(mut,gr)
  print(length(t))
  af=gen[subjectHits(t),]
  af=af[,which(grepl("-T",colnames(af)))]
  af=unlist(af)
  af=as.data.frame(af)
  af$seqnames=as.character(seqnames(gr[subjectHits(t)]))
  af$pos=start(gr[subjectHits(t)])
  af$sid=x
  af
},mc.cores=5)
names(split2)=samples[7:11]
saveRDS(split2,file="split2_violin_v2.RDS")

split3=mclapply(samples[c(12:16)],FUN=function(x){
  print(x)
  vcf=readVcf(paste(x,"-mutect.vcf.gz",sep=""),"hg19")
  mut=maf.gastric2[which(maf.gastric2$sid==x)]
  t=findOverlaps(mut,diploid[[x]])
  mut=mut[unique(queryHits(t))]
  gr=rowRanges(vcf)[which(rowRanges(vcf)$FILTER=="PASS")]
  seqlevelsStyle(gr)="UCSC"
  gen=as.data.frame(geno(vcf)$FREQ)[which(rowRanges(vcf)$FILTER=="PASS"),]
  t=findOverlaps(mut,gr)
  print(length(t))
  af=gen[subjectHits(t),]
  af=af[,which(grepl("-T",colnames(af)))]
  af=unlist(af)
  af=as.data.frame(af)
  af$seqnames=as.character(seqnames(gr[subjectHits(t)]))
  af$pos=start(gr[subjectHits(t)])
  af$sid=x
  af
},mc.cores=5)
names(split3)=samples[12:16]
saveRDS(split3,file="split3_violin_v2.RDS")

split4=mclapply(samples[c(17:21)],FUN=function(x){
  print(x)
  vcf=readVcf(paste(x,"-mutect.vcf.gz",sep=""),"hg19")
  mut=maf.gastric2[which(maf.gastric2$sid==x)]
  t=findOverlaps(mut,diploid[[x]])
  mut=mut[unique(queryHits(t))]
  gr=rowRanges(vcf)[which(rowRanges(vcf)$FILTER=="PASS")]
  seqlevelsStyle(gr)="UCSC"
  gen=as.data.frame(geno(vcf)$FREQ)[which(rowRanges(vcf)$FILTER=="PASS"),]
  t=findOverlaps(mut,gr)
  print(length(t))
  af=gen[subjectHits(t),]
  af=af[,which(grepl("-T",colnames(af)))]
  af=unlist(af)
  af=as.data.frame(af)
  af$seqnames=as.character(seqnames(gr[subjectHits(t)]))
  af$pos=start(gr[subjectHits(t)])
  af$sid=x
  af
},mc.cores=5)
names(split4)=samples[17:21]
saveRDS(split4,file="split4_violin_v2.RDS")

split5=mclapply(samples[c(22:26)],FUN=function(x){
  print(x)
  vcf=readVcf(paste(x,"-mutect.vcf.gz",sep=""),"hg19")
  mut=maf.gastric2[which(maf.gastric2$sid==x)]
  t=findOverlaps(mut,diploid[[x]])
  mut=mut[unique(queryHits(t))]
  gr=rowRanges(vcf)[which(rowRanges(vcf)$FILTER=="PASS")]
  seqlevelsStyle(gr)="UCSC"
  gen=as.data.frame(geno(vcf)$FREQ)[which(rowRanges(vcf)$FILTER=="PASS"),]
  t=findOverlaps(mut,gr)
  print(length(t))
  af=gen[subjectHits(t),]
  af=af[,which(grepl("-T",colnames(af)))]
  af=unlist(af)
  af=as.data.frame(af)
  af$seqnames=as.character(seqnames(gr[subjectHits(t)]))
  af$pos=start(gr[subjectHits(t)])
  af$sid=x
  af
},mc.cores=5)
names(split5)=samples[22:26]
saveRDS(split5,file="split5_violin_v2.RDS")

split6=mclapply(samples[c(27:31)],FUN=function(x){
  print(x)
  vcf=readVcf(paste(x,"-mutect.vcf.gz",sep=""),"hg19")
  mut=maf.gastric2[which(maf.gastric2$sid==x)]
  t=findOverlaps(mut,diploid[[x]])
  mut=mut[unique(queryHits(t))]
  gr=rowRanges(vcf)[which(rowRanges(vcf)$FILTER=="PASS")]
  seqlevelsStyle(gr)="UCSC"
  gen=as.data.frame(geno(vcf)$FREQ)[which(rowRanges(vcf)$FILTER=="PASS"),]
  t=findOverlaps(mut,gr)
  print(length(t))
  af=gen[subjectHits(t),]
  af=af[,which(grepl("-T",colnames(af)))]
  af=unlist(af)
  af=as.data.frame(af)
  af$seqnames=as.character(seqnames(gr[subjectHits(t)]))
  af$pos=start(gr[subjectHits(t)])
  af$sid=x
  af
},mc.cores=5)
names(split6)=samples[27:31]
saveRDS(split6,file="split6_violin_v2.RDS")

split7=mclapply(samples[c(32:36)],FUN=function(x){
  print(x)
  vcf=readVcf(paste(x,"-mutect.vcf.gz",sep=""),"hg19")
  mut=maf.gastric2[which(maf.gastric2$sid==x)]
  t=findOverlaps(mut,diploid[[x]])
  mut=mut[unique(queryHits(t))]
  gr=rowRanges(vcf)[which(rowRanges(vcf)$FILTER=="PASS")]
  seqlevelsStyle(gr)="UCSC"
  gen=as.data.frame(geno(vcf)$FREQ)[which(rowRanges(vcf)$FILTER=="PASS"),]
  t=findOverlaps(mut,gr)
  print(length(t))
  af=gen[subjectHits(t),]
  af=af[,which(grepl("-T",colnames(af)))]
  af=unlist(af)
  af=as.data.frame(af)
  af$seqnames=as.character(seqnames(gr[subjectHits(t)]))
  af$pos=start(gr[subjectHits(t)])
  af$sid=x
  af
},mc.cores=5)
names(split7)=samples[32:36]
saveRDS(split7,file="split7_violin_v2.RDS")

split8=mclapply(samples[c(37:40)],FUN=function(x){
  print(x)
  vcf=readVcf(paste(x,"-mutect.vcf.gz",sep=""),"hg19")
  mut=maf.gastric2[which(maf.gastric2$sid==x)]
  t=findOverlaps(mut,diploid[[x]])
  mut=mut[unique(queryHits(t))]
  gr=rowRanges(vcf)[which(rowRanges(vcf)$FILTER=="PASS")]
  seqlevelsStyle(gr)="UCSC"
  gen=as.data.frame(geno(vcf)$FREQ)[which(rowRanges(vcf)$FILTER=="PASS"),]
  t=findOverlaps(mut,gr)
  print(length(t))
  af=gen[subjectHits(t),]
  af=af[,which(grepl("-T",colnames(af)))]
  af=unlist(af)
  af=as.data.frame(af)
  af$seqnames=as.character(seqnames(gr[subjectHits(t)]))
  af$pos=start(gr[subjectHits(t)])
  af$sid=x
  af
},mc.cores=5)
names(split8)=samples[37:40]
saveRDS(split8,file="split8_violin_v2.RDS")

split9=mclapply(samples[5],FUN=function(x){
  print(x)
  vcf=readVcf(paste(x,"-mutect.vcf.gz",sep=""),"hg19")
  mut=maf.gastric2[which(maf.gastric2$sid==x)]
  t=findOverlaps(mut,diploid[[x]])
  mut=mut[unique(queryHits(t))]
  gr=rowRanges(vcf)[which(rowRanges(vcf)$FILTER=="PASS")]
  seqlevelsStyle(gr)="UCSC"
  gen=as.data.frame(geno(vcf)$FREQ)[which(rowRanges(vcf)$FILTER=="PASS"),]
  t=findOverlaps(mut,gr)
  print(length(t))
  af=gen[subjectHits(t),]
  af=af[,which(grepl("-T",colnames(af)))]
  af=unlist(af)
  af=as.data.frame(af)
  af$seqnames=as.character(seqnames(gr[subjectHits(t)]))
  af$pos=start(gr[subjectHits(t)])
  af$sid=x
  af
},mc.cores=5)
names(split9)=samples[5]
saveRDS(split9,file="split9_violin_v2.RDS")

split10=mclapply(samples[6],FUN=function(x){
  print(x)
  if (x=="HK-pfg076"){
    filename="HK-pfg076-T"
  }
  vcf=readVcf(paste(filename,"-mutect.vcf.gz",sep=""),"hg19")
  mut=maf.gastric2[which(maf.gastric2$sid==x)]
  t=findOverlaps(mut,diploid[[x]])
  mut=mut[unique(queryHits(t))]
  gr=rowRanges(vcf)[which(rowRanges(vcf)$FILTER=="PASS")]
  seqlevelsStyle(gr)="UCSC"
  gen=as.data.frame(geno(vcf)$FREQ)[which(rowRanges(vcf)$FILTER=="PASS"),]
  t=findOverlaps(mut,gr)
  print(length(t))
  af=gen[subjectHits(t),]
  af=af[,which(grepl("-T",colnames(af)))]
  af=unlist(af)
  af=as.data.frame(af)
  af$seqnames=as.character(seqnames(gr[subjectHits(t)]))
  af$pos=start(gr[subjectHits(t)])
  af$sid=x
  af
},mc.cores=5)
names(split10)=samples[6]
saveRDS(split10,file="split10_violin_v2.RDS")

df=NULL
for (i in c(1:10)){
  split=readRDS(paste(i,"_violin_v2.RDS",sep=""))
  split=do.call(rbind,split)
  rownames(split)=NULL
  df=rbind(df,split)
  print(i)
} #1088340


# save summarized output to table
write.table(df,"fig7e_table.df.txt",sep="\t",quote=FALSE,col.names=TRUE,row.names=FALSE)
```

Read in summarized table

```
df=read.table("fig7e_table.df.txt",header=TRUE)
```

```
  ggplot(df,aes(x=factor(sid),y=af))+geom_violin(aes(fill=sid))+
    theme(panel.grid.major = element_blank(),
          panel.grid.minor = element_blank(),
          panel.background = element_blank(),
          axis.line = element_line(colour="black"))+
    theme(axis.title.y=element_blank(),
          axis.text.y=element_blank(),
          axis.ticks.y=element_blank())+
    coord_flip()
```

```
  ggplot(df,aes(x=factor(sid),y=af))+geom_violin(aes(fill="grey"))+
    scale_fill_manual(values="#999999")+
    theme(panel.grid.major = element_blank(),
          panel.grid.minor = element_blank(),
          panel.background = element_blank(),
          axis.line = element_line(colour="black"))+
    theme(axis.title.y=element_blank(),
          axis.text.y=element_blank(),
          axis.ticks.y=element_blank())+
    theme(legend.position="none")+
    coord_flip()
```

```
  # individual plot
  remove=NULL
  p=as.list(numeric(40))
  names(p)=unique(df$sid)
  for (x in unique(df$sid)){
    print(x)
    dat=df[which(df$sid==x),]
    dat=with(dat,GRanges(seqnames,IRanges(pos,pos),af=af,sid=sid))
    seqlevelsStyle(dat)<- "UCSC"
    mut=maf[which(maf$sid==x)]
    z=findOverlaps(dat,mut)
    cbs=dat[queryHits(z)]$af
    print(cbs)
    if (length(z)==0){
      remove=c(remove,x)
    }
    dat=as.data.frame(dat)
    p[[x]]<-  ggplot(dat,aes(x=factor(sid),y=af))+geom_violin(aes(fill="grey"))+
      scale_fill_manual(values="#999999")+
      theme(panel.grid.major = element_blank(),
            panel.grid.minor = element_blank(),
            panel.background = element_blank(),
            axis.line = element_line(colour="black"))+
      theme(axis.title.y=element_blank(),
            axis.text.y=element_blank(),
            axis.ticks.y=element_blank())+
      theme(legend.position="none")+
      geom_hline(yintercept=cbs,color="red")+
      coord_flip(ylim=c(0,1))+
      theme(axis.title.x=element_blank(),
            axis.text.x=element_blank(),
            axis.ticks.x=element_blank())
  }
```

```
## [1] "TCGA-BR-8690"
## [1] 0.132 0.152
## [1] "apollo21"
## [1] 0.788
## [1] "HK-pfg116"
## [1] 0.519 0.411
## [1] "HK-pfg092"
## [1] 0.315 0.154 0.191
## [1] "HK-pfg054"
## [1] 0.395 0.101
## [1] "CGP_donor_GC00047"
## [1] 0.174 0.160
## [1] "TCGA-D7-6528"
## [1] 0.214 0.292 0.340
## [1] "TCGA-D7-6822"
## [1] 0.426 0.367 0.474
## [1] "TCGA-D7-6527"
## [1] 0.382 0.402 0.143 0.192
## [1] "apollo23"
## [1] 0.464
## [1] "tan2000639"
## [1] 0.230 0.217
## [1] "apollo10"
## [1] 0.316 0.265
## [1] "tan980437"
## [1] 0.169 0.183
## [1] "TCGA-BR-4280"
## [1] 0.226 0.351
## [1] "HK-pfg424"
## [1] 0.173
## [1] "HK-pfg038"
## [1] 0.544
## [1] "CGP_donor_GC00054"
## numeric(0)
## [1] "CGP_donor_GC00051"
## [1] 0.286
## [1] "TCGA-CG-5730"
## [1] 0.615
## [1] "HK-pfg064"
## [1] 0.264
## [1] "HK-pfg107"
## numeric(0)
## [1] "TCGA-CG-4442"
## [1] 0.419
## [1] "HK-pfg119"
## numeric(0)
## [1] "HK-pfg072"
## [1] 0.167
## [1] "HK-pfg272"
## [1] 0.133 0.175
## [1] "apollo17"
## [1] 0.686 0.523
## [1] "apollo11"
## [1] 0.292
## [1] "HK-pfg069"
## [1] 0.318
## [1] "TCGA-BR-6452"
## [1] 0.219
## [1] "CGP_donor_GC00052"
## [1] 0.242
## [1] "HK-pfg030"
## [1] 0.197
## [1] "TCGA-IN-7806"
## [1] 0.384
## [1] "apollo16"
## [1] 0.222
## [1] "HK-pfg060"
## numeric(0)
## [1] "HK-pfg277"
## numeric(0)
## [1] "TCGA-EQ-5647"
## numeric(0)
## [1] "apollo2"
## [1] 0.289
## [1] "TCGA-CG-5724"
## numeric(0)
## [1] "tan76629543"
## [1] 0.338 0.475 0.190 0.372
## [1] "HK-pfg076"
## [1] 0.158
```

```
  # remove 7 samples with cbs hotspot mutations filtered out by diploid regions
  
  p=p[which(!names(p) %in% remove)]
  grid.arrange(arrangeGrob(p[[1]],p[[2]],p[[3]],p[[4]],p[[5]],
                           p[[6]],p[[7]],p[[8]],p[[9]],p[[10]],
                           p[[11]],p[[12]],p[[13]],p[[14]],p[[15]],
                           p[[16]],p[[17]],p[[18]],p[[19]],p[[20]],
                           ncol=1,nrow=20,heights=rep(1,20)))
```

```
# violin_1-20_diploid.pdf
  
    grid.arrange(arrangeGrob(p[[21]],p[[22]],p[[23]],p[[24]],p[[25]],
                           p[[26]],p[[27]],p[[28]],p[[29]],p[[30]],
                           p[[31]],p[[32]],p[[33]],
                           ncol=1,nrow=20,heights=rep(1,20)))
```

```
# violin_21-40_diploid.pdf
```

## Figure F Variant allele frequency comparison between CBS hotspot mutations and known driver mutations

```
### get driver mutations
roi.cds <- bed.to.granges('Ensembl75.CDS.bed')
roi.cds.ext <- reduce(roi.cds + 5) # extend each region with +/- 5 bases and get all non-overlapping regions, 208217

maf.gastric=maf.to.granges("gastric_RF_prefiltered.MAF")

genes=read.delim("gene_coordinates_biomart_16feb17.txt",sep="\t",header=T)

gene=genes[which(genes$Associated.Gene.Name %in% c("TP53","CDH1","SMAD4","ARID1A","MUC6","KRAS","PIK3CA","APC")),]
gene=GRanges(seqnames=paste("chr",gene$Chromosome.scaffold.name,sep=""),IRanges(gene$Gene.Start..bp.,gene$Gene.End..bp.),gene=gene$Associated.Gene.Name)
z=findOverlaps(gene,roi.cds.ext) #150
cds=roi.cds.ext[subjectHits(z)]
z=findOverlaps(maf.gastric,cds) #170
driver.muts=maf.gastric[queryHits(z)]

df=as.data.frame(driver.muts)
df$seqnames=as.character(df$seqnames)
df$seqnames=substr(df$seqnames,4,nchar(df$seqnames))
unique(df[,c("seqnames","start","end","ral","tal")]) #135

### run VEP
vep_pos <- read.csv("vep_pos_v2.txt", sep="", stringsAsFactors=FALSE) #638 HIGH and MODERATE
vep_pos=unique(vep_pos) #34, 124
chr=gsub("\\:.*","",vep_pos$Location)
vep_pos$Location=gsub("*.\\:","",vep_pos$Location)
pos=gsub(".*\\-","",vep_pos$Location)
pos=as.numeric(pos)

vep=GRanges(seqnames=paste("chr",chr,sep=""),IRanges(pos,pos)) #34, 124
z=findOverlaps(driver.muts,vep) #39, 161
driver.muts.trunc=driver.muts[queryHits(z)]
driver.muts.trunc2=driver.muts[queryHits(z)]

df=as.data.frame(driver.muts.trunc2)
df=df[which(df$seqnames != "chr17"),] #83
```

```
candidate.muts <- read.csv("candidate_muts.csv", header=FALSE, stringsAsFactors=FALSE) #37
table(candidate.muts$V1) #27
```

```
## 
##          apollo10          apollo11          apollo17          apollo23 
##                 1                 1                 1                 1 
## CGP_donor_GC00047 CGP_donor_GC00052         HK-pfg030         HK-pfg034 
##                 1                 1                 1                 1 
##         HK-pfg054         HK-pfg069         HK-pfg072         HK-pfg092 
##                 1                 1                 1                 1 
##         HK-pfg119         HK-pfg144         HK-pfg146         HK-pfg180 
##                 1                 1                 1                 1 
##         HK-pfg272         HK-pfg344         HK-pfg378        tan2000639 
##                 2                 1                 1                 1 
##       tan76629543      TCGA-BR-4280      TCGA-BR-6452      TCGA-CG-4442 
##                 3                 1                 1                 1 
##      TCGA-D7-6527      TCGA-D7-6528      TCGA-D7-6822 
##                 3                 2                 5
```

```
# choose equal number of mutations from each sample

# get allele frequency from mutect files

# matched samples in hotspots and drivers, except TP53
df1=candidate.muts
df1=df1[c(1,2,17,18,21,25,27,30,31,33),] #10

driver.muts <- read.csv("driver_muts.csv", header=FALSE, stringsAsFactors=FALSE) #32
df2=driver.muts
df2=df2[c(1:4,6,23,24,28:30),] #10
df3=driver.muts
df3=df3[c(28,3,24,23,2,6,4,30,29,1),] #10

wilcox.test(df1$V4,df2$V4) # default is 2-sided test
```

```
## 
##  Wilcoxon rank sum test
## 
## data:  df1$V4 and df2$V4
## W = 37, p-value = 0.3527
## alternative hypothesis: true location shift is not equal to 0
```

```
wilcox.test(df1$V4,df3$V4,paired=TRUE)
```

```
## 
##  Wilcoxon signed rank test
## 
## data:  df1$V4 and df3$V4
## V = 20, p-value = 0.4922
## alternative hypothesis: true location shift is not equal to 0
```

```
df=data.frame(group=rep(c("candidate","driver"),each=10),AF=c(df1$V4,df3$V4),samples=c(df1$V1,df3$V1))
df$pair=rep(c(1:10),2)
df=df[with(df,order(group,AF)),]
df[which(df$group=="candidate"),"AF"][5] #0.217
```

```
## [1] 0.217
```

```
df[which(df$group=="driver"),"AF"][5] #0.245
```

```
## [1] 0.245
```

```
df$col=numeric(nrow(df))
df[which(df$group=="candidate"),"col"][5] <- 1
df[which(df$group=="driver"),"col"][5] <- 1

print(ggplot(df,aes(factor(df$group),AF,group=pair))+geom_point(aes(colour=factor(col)))+geom_line(linetype="dashed")+scale_color_manual(values=c("#999999","#FF3300"))+
  theme(legend.position = "none")+ylab("Allele fraction")+xlab(NULL)+ylim(c(0,1))+
  theme(panel.grid.major = element_blank(),
        panel.grid.minor = element_blank(),
        panel.background = element_blank(),
        axis.line = element_line(colour="black")))
```
