# Supplementary material for: Mutation hotspots at CTCF binding sites coupled to chromosomal instability in gastrointestinal cancers
Source: Nat Commun. 2018 Apr 18;9:1520. doi: 10.1038/s41467-018-03828-2 (PMC5906695; doi:10.1038/s41467-018-03828-2)
Supplement: Supplementary file 8 — Supplementary Data 5 [file 41467_2018_3828_MOESM8_ESM.zip › Rmarkdowns/Supplementary Figure 1/Supplementary_Figure1_Summary_of_data_rev.html]

Supplementary Figure 1 - Summary of data


# Supplementary Figure 1 - Summary of data

This is the R Markdown for Supplementary Figure 1, which consists of 4 parts.

## Figure C

Comparing Mutation count with at proportion of C>A in all samples

```
outliers=c("HK-pfg138","tan2000721","CGP_donor_GC00021","CGP_donor_GC00007","tan2000085","apollo18","CGP_donor_GC00036","CGP_donor_GC00034","tan990068","CGP_donor_GC00016","CGP_donor_GC00006","CGP_donor_GC00032","CGP_donor_GC00035")
out=data.frame(samples=outliers,mut.count=numeric(length(outliers)),ca.count=numeric(length(outliers)))
```

```
for (i in outliers){
  print(i)
  file=read.delim(paste("truth_",i,".txt",sep=""), stringsAsFactors=FALSE) # read each outlier's truth file
  file=file[which(file$X.CHROM!="Y"),]
  out[which(out$samples==i),"mut.count"]<-nrow(file)
  file$mut=paste(file$REF,file$ALT,sep=">")
  file[which(file$mut=="A>T"),"mut"]<-"T>A"
  file[which(file$mut=="A>C"),"mut"]<-"T>G"
  file[which(file$mut=="A>G"),"mut"]<-"T>C"
  file[which(file$mut=="G>A"),"mut"]<-"C>T"
  file[which(file$mut=="G>C"),"mut"]<-"C>G"
  file[which(file$mut=="G>T"),"mut"]<-"C>A"
  out[which(out$samples==i),"ca.count"]<-sum(file$mut=="C>A")/nrow(file)
}

# save summarized output to table
write.table(out,file="suppfig1.out.txt",sep="\t",quote=FALSE,col.names=TRUE,row.names=FALSE)
```

Read in summarized table

```
out=read.table("suppfig1.out.txt",header=TRUE)
```

```
gastric_consolidated_meta <- read.delim("gastric_consolidated_meta.tsv", stringsAsFactors=FALSE) #212
out$ctype=numeric(length(outliers))
for (i in 1:nrow(out)){
  out$ctype[i]=gastric_consolidated_meta[which(gastric_consolidated_meta$Sample.ID==out$samples[i]),"Batch"]
}

maf.gastric=maf.to.granges("gastric_RF.MAF") #4192877
```

```
## [1] ">> Reading compact MAF ..."
```

```
maf.gastric=maf.gastric[-which(seqnames(maf.gastric)=="chrY"),]

maf.gastric=as.data.frame(maf.gastric)
maf.gastric=maf.gastric[-which(maf.gastric$tal=="FALSE"),] #4192875
mut.count=aggregate(seqnames~sid+ctype,maf.gastric,length) #192
colnames(mut.count)[3]="mut.count"

maf.gastric$mut=paste(maf.gastric$ral,maf.gastric$tal,sep=">")
table(maf.gastric$mut)
```

```
## 
##    A>C    A>G    A>T    C>A    C>G    C>T    G>A    G>C    G>T    T>A 
## 359585 531294 199858 298590  91380 612608 612968  91549 299114 200253 
##    T>C    T>G 
## 530311 361508
```

```
#    A>C    A>G    A>T    C>A    C>G    C>T    G>A    G>C    G>T 
# 359585 531294 199858 298590  91380 612608 612968  91549 299114 
#    T>A    T>C    T>G 
# 200253 530311 361508 
maf.gastric[which(maf.gastric$mut=="A>T"),"mut"]<-"T>A"
maf.gastric[which(maf.gastric$mut=="A>C"),"mut"]<-"T>G"
maf.gastric[which(maf.gastric$mut=="A>G"),"mut"]<-"T>C"
maf.gastric[which(maf.gastric$mut=="G>A"),"mut"]<-"C>T"
maf.gastric[which(maf.gastric$mut=="G>C"),"mut"]<-"C>G"
maf.gastric[which(maf.gastric$mut=="G>T"),"mut"]<-"C>A"
table(maf.gastric$mut)
```

```
## 
##     C>A     C>G     C>T     T>A     T>C     T>G 
##  597704  182929 1225576  400111 1061605  721093
```

```
#     C>A     C>G     C>T     T>A     T>C     T>G 
#  597704  182929 1225576  400111 1061605  721093 
ca.count=aggregate(seqnames~sid+mut,maf.gastric,length) #1152=192*6
colnames(ca.count)[3]="spectrum_count"

df=merge(mut.count,ca.count,by="sid") #1152
df$ca.count=df$spectrum_count/df$mut.count
df=df[which(df$mut=="C>A"),] #192

df=df[,c("sid","ctype","mut.count","ca.count")]
colnames(out)[1]="sid"
df=rbind(df,out[,c("sid","ctype","mut.count","ca.count")]) #205

df$ctype=as.character(df$ctype)
df$ctype=ifelse(df$ctype=="tan","SG",df$ctype)
df$ctype=factor(df$ctype,levels=c("SG","HK","TCGA","ICGC"))

ggplot(df,aes(x=ca.count,y=mut.count,col=ctype))+
  geom_point()+
  scale_y_log10()+
  scale_color_manual(values=c("#33CC00","#FFCC00","#0066CC","#FF0000"))+
    theme(panel.grid.major = element_blank(),
        panel.grid.minor = element_blank(),
        panel.background = element_blank(),
        axis.line = element_line(colour="black"))
```

```
plot(x=df$ca.count,y=log10(df$mut.count))
text(x=df$ca.count,y=log10(df$mut.count),labels=df$sid,cex=0.7,pos=3)
```

## Figure D

Mutation spectrum/signature across batches and subtypes

```
maf.gastric <- maf.to.granges('gastric_RF_prefiltered.MAF') # 4143709, 192 unique sids
```

```
## [1] ">> Reading compact MAF ..."
```

```
maf.gastric=maf.gastric[-which(maf.gastric$sid %in% c("tan2001206", "tan20021007", "tan980319", "tan2000986", "tan980436"))] # 4119812
maf.gastric=maf.gastric[-which(seqnames(maf.gastric)=="chrY")] # 4116299
t=as.data.frame(maf.gastric)
t$seqnames=as.character(t$seqnames)

subtype_classification <- read.delim("subtype_classification.txt", stringsAsFactors=FALSE)
GS=subtype_classification[which(subtype_classification$Molecular.Subtype=="GS"),"Sample.ID"] # 19
GS=c(GS,"apollo1_new") # 20
CIN=subtype_classification[which(subtype_classification$Molecular.Subtype=="CIN"),"Sample.ID"] # 42
EBV=subtype_classification[which(subtype_classification$Molecular.Subtype=="EBV"),"Sample.ID"] # 17
MSI=subtype_classification[which(subtype_classification$Molecular.Subtype=="MSI"),"Sample.ID"] # 18
MSI=c(MSI,"CGP_donor_GC00031") # 19

GS=GS[which(GS %in% unique(maf.gastric$sid))] # 11
CIN=CIN[which(CIN %in% unique(maf.gastric$sid))] # 41
EBV=EBV[which(EBV %in% unique(maf.gastric$sid))] # 17
MSI=MSI[which(MSI %in% unique(maf.gastric$sid))] # 19

t$subtype="others"

t$subtype=ifelse(t$sid %in% GS,"GS",t$subtype)
t$subtype=ifelse(t$sid %in% MSI,"MSI",t$subtype)
t$subtype=ifelse(t$sid %in% CIN,"CIN",t$subtype)
t$subtype=ifelse(t$sid %in% EBV,"EBV",t$subtype)

maf.gastric=GRanges(seqnames=t$seqnames,
                    IRanges(start=t$start,end=t$end),
                    ral=t$ral,
                    tal=t$tal,
                    sid=t$sid,
                    ctype=t$ctype,
                    subtype=t$subtype,
                    id=t$sid)

# Based on ctype
gastric_vr.a1 <- VRanges(
  seqnames=seqnames(maf.gastric),
  ranges=ranges(maf.gastric),
  ref=maf.gastric$ral,
  alt=maf.gastric$tal,
  sampleNames=maf.gastric$sid,
  seqinfo=seqinfo(maf.gastric),
  ctype=maf.gastric$ctype
)

idx_snv = ref(gastric_vr.a1) %in% DNA_BASES & alt(gastric_vr.a1) %in% DNA_BASES
gastric_vr.a1[!idx_snv]
```

```
## VRanges object with 2 ranges and 1 metadata column:
##       seqnames               ranges strand         ref              alt
##          <Rle>            <IRanges>  <Rle> <character> <characterOrRle>
##   [1]    chr15 [87219183, 87219183]      +           A            FALSE
##   [2]     chr4 [24367756, 24367756]      +           G            FALSE
##           totalDepth       refDepth       altDepth   sampleNames
##       <integerOrRle> <integerOrRle> <integerOrRle> <factorOrRle>
##   [1]           <NA>           <NA>           <NA>     tan990489
##   [2]           <NA>           <NA>           <NA>  TCGA-BR-6452
##       softFilterMatrix |    ctype
##               <matrix> | <factor>
##   [1]                  |      tan
##   [2]                  |     TCGA
##   -------
##   seqinfo: 23 sequences from an unspecified genome; no seqlengths
##   hardFilters: NULL
```

```
gastric_vr.a1 = gastric_vr.a1[idx_snv]

gastric_motif.a1 = mutationContext(gastric_vr.a1, BSgenome.Hsapiens.UCSC.hg19)

gastric_mm.a1 = motifMatrix(gastric_motif.a1, group = "ctype", normalize = TRUE)

plotMutationSpectrum(gastric_motif.a1, "ctype",normalize = T) # change colour in illustrator
```

## Figure B

Mutation count/Coverage across batches

```
mutations=read.table('gastric_RF.MAF', header=F, sep="\t")
colnames(mutations)=c("chrom","start","end","ral","tal","sample","batch")
mutations$sample=as.character(mutations$sample)
mutations=mutations[-which(mutations$sample %in% c("tan2001206", "tan20021007", "tan980319", "tan2000986", "tan980436")),] # remove 5 oxidative damaged samples, 4168662
head(mutations)
```

```
##   chrom  start    end ral tal            sample batch
## 1  chr1 564462 564462   A   T          apollo23   tan
## 2  chr1 603321 603321   C   T          apollo24   tan
## 3  chr1 705871 705871   G   A          apollo16   tan
## 4  chr1 756678 756678   T   A         HK-pfg277    HK
## 5  chr1 756885 756885   C   T CGP_donor_GC00020  ICGC
## 6  chr1 766779 766779   C   T          apollo19   tan
```

```
samples=unique(mutations[,c("sample","batch")])
samples=samples[order(samples[,"sample"]),]
mut.count=table(mutations[,"sample"])
head(mut.count)
```

```
## 
## apollo1_new    apollo10    apollo11    apollo12    apollo13    apollo14 
##        6744       18458       14299       13330        8044       22392
```

```
df=data.frame(count=as.numeric(mut.count), batch=as.character(samples[,"batch"]), stringsAsFactors = F)
map=list("TCGA"="TCGA", "HK"="HK", "ICGC"="ICGC", "tan"="SG")
df$batch=sapply(df$batch, function(x) {map[[x]]})
```

## Figure B part 2

Mutation count across batches

```
ggplot(df, aes(x=factor(batch, levels = c("SG","HK","TCGA","ICGC")), y=count))+
  stat_boxplot(geom ='errorbar')+geom_boxplot(outlier.shape = NA)+
  geom_jitter(aes(colour=factor(batch,levels=c("SG","HK","TCGA","ICGC"))), size=2,width=0.1)+
  scale_color_manual(values=c("#33CC00","#FFCC00","#0066CC","#FF0000"))+
  scale_y_log10()+labs(x="", y="mutation count")+
  theme(text = element_text(size=20),
        axis.text.x = element_text(size=20),
        legend.position="none",
        panel.background =element_rect(fill = "white", color="black"))
```

## Figure B part 1

Coverage across batches

```
mutations$batch=as.character(mutations$batch)
meta_data=read.delim("gastric_consolidated_meta.tsv", stringsAsFactors=FALSE)
meta_data=meta_data[which(meta_data$Sample.ID %in% unique(mutations$sample) | meta_data$Sample.ID=="apollo1"),] # 187
meta_data=meta_data[,c("Sample.ID","WGS.mean.coverage","Batch")]
meta_data$Sample.ID[1]="apollo1_new"
meta_data$ctype=numeric(nrow(meta_data))
for (i in 1:nrow(meta_data)){
  meta_data$ctype[i]=unique(mutations[which(mutations$sample==meta_data$Sample.ID[i]),]$batch)
}
meta_data$ctype=sapply(meta_data$ctype, function(x) {map[[x]]})
colnames(meta_data)[2]="coverage"
meta_data$coverage=as.numeric(meta_data$coverage)
head(meta_data)
```

```
##     Sample.ID coverage Batch ctype
## 1 apollo1_new     42.1    SG    SG
## 2    apollo10     40.1    SG    SG
## 3    apollo11     39.6    SG    SG
## 4    apollo12     42.6    SG    SG
## 5    apollo13     43.6    SG    SG
## 6    apollo14     40.1    SG    SG
```

```
summary(meta_data$coverage)
```

```
##    Min. 1st Qu.  Median    Mean 3rd Qu.    Max. 
##   28.10   41.60   65.60   65.27   89.60  106.60
```

```
ggplot(meta_data, aes(x=factor(ctype, levels = c("SG","HK","TCGA","ICGC")), y=coverage))+
  stat_boxplot(geom ='errorbar')+geom_boxplot(outlier.shape = NA)+
  geom_jitter(aes(colour=factor(ctype,levels=c("SG","HK","TCGA","ICGC"))), size=2,width=0.1)+
  scale_color_manual(values=c("#33CC00","#FFCC00","#0066CC","#FF0000")) +labs(x="", y="coverage")+
  theme(text = element_text(size=20),
        axis.text.x = element_text(size=20),
        legend.position="none",
        panel.background =element_rect(fill = "white",color="black"))
```
