# Supplementary material for: Mutation hotspots at CTCF binding sites coupled to chromosomal instability in gastrointestinal cancers
Source: Nat Commun. 2018 Apr 18;9:1520. doi: 10.1038/s41467-018-03828-2 (PMC5906695; doi:10.1038/s41467-018-03828-2)
Supplement: Supplementary file 8 — Supplementary Data 5 [file 41467_2018_3828_MOESM8_ESM.zip › Rmarkdowns/Supplementary Figure 10/supplementary_Figure10_cnv_residual_expression_rev.html]

Supplementary Figure 10 - CNV residual expression


# Supplementary Figure 10 - CNV residual expression

This is the R Markdown for Supplementary Figure 10, which consists of 3 parts.

## Figure A-C

Boxplots for cnv residual expression

```
residual.expr.boxplot <- function(gene, mut.samples, rnaseq.data, samples) {
  expr=rnaseq.data[rnaseq.data[,1]==gene,]
  expr=(expr[-1])
  expr.v=as.numeric(expr)

  cnv.gene=as.numeric(cnv.wgs[row.names(cnv.wgs)==gene,])
  
  mut.status=rep("WT", 35)
  names(mut.status)=names(rnaseq.data)[-1]
  mut.status[mut.samples]="MUT"  
  
  df=data.frame(expr=expr.v,cnv=cnv.gene,purity=purity.wgs[,2],mut=mut.status)  
  model=glm(expr~cnv+purity+cnv*purity, data=df)
  print(summary(model)$coefficients)
  
  residual.exp=expr.v-model$fitted.values
  residual.exp.wt=residual.exp[mut.status=="WT"]
  residual.exp.mut=residual.exp[mut.status=="MUT"]
  df=cbind(df,residual.exp)
  print(wilcox.test(residual.exp.wt,residual.exp.mut))
  
  p=ggplot(df, aes(x=factor(mut.status, levels = c("WT","MUT")), y=residual.exp))+ stat_boxplot(geom ='errorbar')+geom_boxplot(outlier.shape = NA)+
    geom_jitter(aes(colour=mut.status), size=2,width=0.1)+ scale_color_brewer(palette="Paired")+ylab(paste(gene,"expression"))+xlab(NULL)+
    theme(text = element_text(size=20),axis.text.x = element_text(size=20))+theme(legend.position="none") +
    theme(panel.grid.major = element_blank(),
          panel.grid.minor = element_blank(),
          panel.background = element_blank(),
          axis.line = element_line(colour="black"))
  print(p)
}

rnaseq.tcga=read.table("STAD.rnaseqv2_RSEM_genes_normalized_WGS.txt", header=T, sep="\t", check.names=F, stringsAsFactors = F)
rnaseq.tcga=rnaseq.tcga[-1,]
names(rnaseq.tcga)=substr(names(rnaseq.tcga),1,15)
rnaseq.gene.id=do.call(rbind,strsplit(rnaseq.tcga[,1],"[|]"))
rnaseq.tcga[,1]=rnaseq.gene.id[,1]

tcga.samples=read.table("TCGA_sample_ID.txt", header=F, sep="\t", colClasses=c('character', 'character'))
tcga.samples[,1]=substr(tcga.samples[,1], 1, 15)

cnv=read.table("all_thresholded.by_genes.txt", header=T, sep="\t", check.names=F)
names(cnv)=substr(names(cnv),1,15)
cnv.wgs=cnv[,names(rnaseq.tcga)[-1]]
row.names(cnv.wgs)=cnv[,1]

purity=read.table("STAD_purity.csv", sep=",", header=F)
row.names(purity)=purity[,1]
purity.wgs=purity[names(rnaseq.tcga)[-1],] # 35 samples with purity estimates
```

## Figure A

CENPQ

```
residual.expr.boxplot ("CENPQ", mut.samples=c("TCGA-CG-4442-01","TCGA-D7-6527-01","TCGA-D7-6822-01"), rnaseq.tcga, tcga.samples)
```

```
##              Estimate Std. Error    t value   Pr(>|t|)
## (Intercept)  58.95040   23.38562  2.5207969 0.01706806
## cnv          23.85210   53.50387  0.4458014 0.65883880
## purity       73.13457   45.06575  1.6228416 0.11475064
## cnv:purity  -13.05929   90.15122 -0.1448598 0.88575930
## 
##  Wilcoxon rank sum test
## 
## data:  residual.exp.wt and residual.exp.mut
## W = 8, p-value = 0.01253
## alternative hypothesis: true location shift is not equal to 0
```

## Figure B

SPG20

```
residual.expr.boxplot ("SPG20", mut.samples=c("TCGA-D7-6528-01", "TCGA-D7-6527-01", "TCGA-D7-6822-01"), rnaseq.tcga, tcga.samples)
```

```
##              Estimate Std. Error    t value    Pr(>|t|)
## (Intercept)  747.0421   235.1254  3.1772072 0.003357712
## cnv          161.6286   336.8036  0.4798899 0.634674460
## purity      -432.4249   478.0143 -0.9046276 0.372639735
## cnv:purity  -424.5854   670.0988 -0.6336161 0.530978886
## 
##  Wilcoxon rank sum test
## 
## data:  residual.exp.wt and residual.exp.mut
## W = 88, p-value = 0.01253
## alternative hypothesis: true location shift is not equal to 0
```

## Figure C

KCNQ5

```
residual.expr.boxplot ("KCNQ5", mut.samples=c("TCGA-BR-6452-01","TCGA-D7-6527-01","TCGA-BR-4280-01","TCGA-D7-6822-01"), rnaseq.tcga, tcga.samples)
```

```
##               Estimate Std. Error    t value     Pr(>|t|)
## (Intercept)  24.205432   3.187015  7.5950155 1.458193e-08
## cnv           3.834959   6.641462  0.5774269 5.678216e-01
## purity      -32.543422   6.102963 -5.3323967 8.279227e-06
## cnv:purity   -8.410217  11.616099 -0.7240138 4.744860e-01
## 
##  Wilcoxon rank sum test
## 
## data:  residual.exp.wt and residual.exp.mut
## W = 104, p-value = 0.02739
## alternative hypothesis: true location shift is not equal to 0
```
