# Supplementary material for: Mutation hotspots at CTCF binding sites coupled to chromosomal instability in gastrointestinal cancers
Source: Nat Commun. 2018 Apr 18;9:1520. doi: 10.1038/s41467-018-03828-2 (PMC5906695; doi:10.1038/s41467-018-03828-2)
Supplement: Supplementary file 8 — Supplementary Data 5 [file 41467_2018_3828_MOESM8_ESM.zip › Rmarkdowns/Supplementary Figure 11/Supplementary_Figure11_tissue_specific_pancancer_rev.html]

Supplementary Figure 11 - Tissue specific pancancer


# Supplementary Figure 11 - Tissue specific pancancer

This is the R Markdown for Supplementary Figure 11, which consists of 1 part.

## Figure A

Tissue specific version of Figure 6B

```
hotspot <- read.delim("LRmodel_hotspot_nonMSI_prefiltered-5_corrected.tsv", stringsAsFactors=FALSE)
hotspot$mut_region=rownames(hotspot)
hotspot=GRanges(seqnames=hotspot$chrom,IRanges(start=hotspot$start,end=hotspot$end),mut_region=hotspot$mut_region,pval=hotspot$pval,fdr=hotspot$fdr)
hotspot=hotspot[which(hotspot$pval<(0.01/2533374732))]
hotspot=reduce(hotspot)
hotspot$hotspot=c(1:length(hotspot))

# Identify CTCF hotspot
roi.ctcf <- bed.to.granges("ctcf_motif_union.bed")
ctcf.hotspot=hotspot[unique(queryHits(findOverlaps(hotspot,roi.ctcf)))]
other.hotspot=hotspot[-unique(queryHits(findOverlaps(hotspot,roi.ctcf)))]

# read in maf file
maf.gastric <- maf.to.granges('gastric_RF_prefiltered.MAF')
```

```
## [1] ">> Reading compact MAF ..."
```

```
maf.gastric$ctype=as.character(maf.gastric$ctype)
maf.gastric$ctype="gastric"
seqi = seqinfo(Hsapiens)[intersect(seqnames(seqinfo(Hsapiens))[1:23],as.character(seqnames(maf.gastric)))]
maf.gastric=maf.gastric[which(seqnames(maf.gastric) %in% seqnames(seqi))] # 4139879
maf.gastric=maf.gastric[-which(maf.gastric$sid %in% c("tan2001206", "tan20021007", "tan980319", "tan2000986", "tan980436"))] # 4116299

maf.pancan <- maf.to.granges('pancancer.maf.compact.filt500k.classes')
```

```
## [1] ">> Reading compact MAF ..."
```

```
maf.pancan=maf.pancan[which(seqnames(maf.pancan) %in% seqnames(seqi))] # 11598778

maf.total=c(maf.pancan,maf.gastric) # 15715077
maf.total=maf.total[-which(maf.total$ctype=="all")] # 15707250 remove ALL cancer as it only has 1 sample
# number of cancer types
length(unique(maf.total$ctype)) # 22
```

```
## [1] 22
```

```
# number of individuals per cancer type
maf.total$sid=as.character(maf.total$sid)
maf.total$ctype=as.character(maf.total$ctype)
maf.total=as.data.frame(maf.total)
maf.total=maf.total[with(maf.total, order(ctype,sid)),]
ind.maf=split(maf.total, maf.total$ctype) # split by cancer type
sample.count=sapply(ind.maf, function(x) {length(unique(x$sid))}) # number of samples per cancer type
print(sample.count)
```

```
##     aml    astr    blca   brain    brca     cll     crc gastric    hnsc 
##       7     101      20      43     172      28      23     187      29 
##    kich    kirc    lihc    luad    lusc    lymp    medu      ov    paad 
##      15      29      88      64      20      24     100       5      15 
##    prad    skcm    thca    ucec 
##      20      16       9      29
```

```
mut.count=sapply(ind.maf, function(x) {nrow(x)}) # number of mutations per cancer type
print(mut.count)
```

```
##     aml    astr    blca   brain    brca     cll     crc gastric    hnsc 
##    3650   12971  372469  198173 1060612   54613 1086874 4116299  357421 
##    kich    kirc    lihc    luad    lusc    lymp    medu      ov    paad 
##    5019  142419  897472 2619420  887341  141843  139293   23101  122586 
##    prad    skcm    thca    ucec 
##   29862 2248676    9795 1177341
```

```
maf.total$sid=factor(maf.total$sid,levels=unique(maf.total$sid))
ind.maf2=split(maf.total, maf.total$sid) # 1044, split by sample IDs
ind.maf2=sapply(ind.maf2, function(x) {nrow(x)})
print(ind.maf2)
```

```
##            400220            426980            452198            573988 
##               290               839               117               148 
##            758168            804168            869586              PA10 
##               471               618              1167               107 
##             PA102             PA103             PA105             PA107 
##                93                49               339               132 
##             PA109              PA11             PA110             PA112 
##               145               361               103                83 
##             PA116             PA117              PA12             PA131 
##               238               262               207               315 
##             PA134             PA136             PA138              PA14 
##                70               243               106               112 
##             PA143             PA145             PA148             PA149 
##               244               165                76               111 
##             PA157             PA166              PA17              PA20 
##                74               339               107               241 
##              PA21              PA22              PA25               PA3 
##               184               292                98               110 
##              PA36               PA4              PA41              PA43 
##               342               123               132               221 
##              PA46              PA48               PA5              PA53 
##               173               117                82               104 
##              PA54              PA55              PA56              PA58 
##               125                91               100               207 
##              PA59              PA62              PA63              PA64 
##               111               115               291                63 
##              PA65              PA69              PA70              PA73 
##               816               301                99               349 
##              PA75              PA79               PA8              PA81 
##               452                93                62               108 
##              PA82              PA83              PA84              PA85 
##               136               193               195                85 
##              PA86              PA87               PA9              PA90 
##               106               130               127                91 
##              PA93              PA96          SJLGG001          SJLGG002 
##               558               132                51                39 
##          SJLGG003          SJLGG004          SJLGG005          SJLGG006 
##                18                26                29                 7 
##         SJLGG006R          SJLGG007          SJLGG008          SJLGG009 
##                 7                21               159                39 
##          SJLGG010          SJLGG011          SJLGG012          SJLGG013 
##                49                83               100                12 
##          SJLGG015          SJLGG016          SJLGG018          SJLGG019 
##                24                 2                27                 8 
##          SJLGG020          SJLGG021          SJLGG022          SJLGG024 
##                45                15                 3                32 
##          SJLGG025          SJLGG026          SJLGG027          SJLGG028 
##                23                 6                36                35 
##          SJLGG029          SJLGG030          SJLGG031          SJLGG032 
##                44                19                 5                42 
##          SJLGG033          SJLGG034          SJLGG035          SJLGG037 
##                30               198                25                25 
##          SJLGG038          SJLGG039          SJLGG040          SJLGG042 
##                63                12                51               125 
##      TCGA-BL-A13J      TCGA-BT-A20P      TCGA-BT-A20Q      TCGA-BT-A20T 
##             11457              9656              8334             29294 
##      TCGA-BT-A20V      TCGA-BT-A3PH      TCGA-BT-A3PJ      TCGA-C4-A0F7 
##             11428             37085             47193              8116 
##      TCGA-CF-A27C      TCGA-CF-A3MF      TCGA-DK-A1A5      TCGA-DK-A1A6 
##             12229              3069             27633             31044 
##      TCGA-DK-A1A7      TCGA-DK-A1AA      TCGA-DK-A1AE      TCGA-DK-A1AG 
##              2344              8883             12611              8572 
##      TCGA-DK-A3IL      TCGA-FT-A3EE      TCGA-GD-A2C5      TCGA-H4-A2HQ 
##             17230             11447             22743             52101 
##      TCGA-02-2483      TCGA-02-2485      TCGA-06-0157      TCGA-06-0214 
##              3938              5837              4758              6148 
##      TCGA-06-0686      TCGA-06-0744      TCGA-06-0745      TCGA-06-2557 
##              6982              9769              6288              5335 
##      TCGA-06-2570      TCGA-06-5411      TCGA-06-5415      TCGA-14-1823 
##              3102              4078              4842              4775 
##      TCGA-14-2554      TCGA-15-1444      TCGA-19-2620      TCGA-19-2624 
##             10287              1910              7686              3894 
##      TCGA-19-2629      TCGA-19-5960      TCGA-26-5132      TCGA-26-5135 
##              7385              5989              6822              7267 
##      TCGA-27-1831      TCGA-27-2523      TCGA-27-2528      TCGA-32-1970 
##              5500              6822              6133              7681 
##      TCGA-41-5651      TCGA-CS-5395      TCGA-CS-6668      TCGA-DB-5278 
##              9877              5133              2617               405 
##      TCGA-DU-5874      TCGA-DU-6401      TCGA-DU-6407      TCGA-DU-7009 
##              3767              2183              1848              1525 
##      TCGA-DU-7301      TCGA-E1-5318      TCGA-E1-5319      TCGA-EZ-7264 
##              2474              2482              1823              2294 
##      TCGA-FG-5964      TCGA-FG-8182      TCGA-HT-7602      TCGA-HT-7689 
##              2573              2539               931              3437 
##      TCGA-HT-7695      TCGA-HW-7487      TCGA-IK-7675           PD3851a 
##              1328              1986              5723              1561 
##           PD3890a           PD3904a           PD3905a           PD3945a 
##              5608              5379              4272              9934 
##           PD3989a           PD4005a           PD4006a           PD4069a 
##              2080              5773              8711              2068 
##           PD4072a           PD4080a           PD4085a           PD4086a 
##             26885              1746              2438              1929 
##           PD4088a           PD4103a           PD4107a           PD4109a 
##              1576              5042              9879              9405 
##           PD4115a           PD4116a           PD4120a           PD4192a 
##              9548              7682             67364              3439 
##           PD4194a           PD4198a           PD4199a           PD4224a 
##              1241              4180              6524              7955 
##           PD4225a           PD4248a           PD4255a           PD4261a 
##              2104              2188              4584              1955 
##           PD4266a           PD4267a           PD4315a           PD4604a 
##              2706              2417              4001              8585 
##           PD4605a           PD4606a           PD4607a           PD4608a 
##              2450              1390             25464              2306 
##           PD4613a           PD4826a           PD4833a           PD4836a 
##              1986              3973              7026              4433 
##           PD4841a           PD4847a           PD4951a           PD4952a 
##              7192             11521              2973             12156 
##           PD4953a           PD4954a           PD4955a           PD4957a 
##              7193              5341              6218              2961 
##           PD4958a           PD4959a           PD4962a           PD4963a 
##              8332              4234              5542              1481 
##           PD4965a           PD4966a           PD4967a           PD4968a 
##              2845              1043              3579              4206 
##           PD4970a           PD4971a           PD4972a           PD4975a 
##              2170              2520              1054              6867 
##           PD4976a           PD4980a           PD4981a           PD4982a 
##              5640              4128              1844              1564 
##           PD4983a           PD4985a           PD4986a           PD5928a 
##              1614              1821              1803              6492 
##           PD5934a           PD5935a           PD5936a           PD5942a 
##             11873             10701              2562              6146 
##           PD5944a           PD5947a           PD5951a           PD5956a 
##              2785              1659              4020              4447 
##           PD6018a           PD6041a           PD6042a           PD6043a 
##              2732              1613              6544             22443 
##           PD6044a           PD6045a           PD6046a           PD6049a 
##              2461              2079              3334              7261 
##           PD6409a           PD6410a           PD6411a           PD6413a 
##              6133              7132              7559              4834 
##           PD6417a           PD6418a           PD6422a           PD6466b 
##              1442              1924              4675              1510 
##           PD6719a           PD6720a           PD6721a           PD6722a 
##              4593              1876              2053             10978 
##           PD7199a           PD7201a           PD7207a           PD7208a 
##              1079              1462              2582              2269 
##           PD7209a           PD7210a           PD7212a           PD7214a 
##              1533              1273              1885              1813 
##           PD7215a           PD7216a           PD7217a           PD7218a 
##              6871              1537             10005              1826 
##           PD7219a           PD7221a           PD7321a           PD7404a 
##             12608              1873              6384             12663 
##           PD7409a           PD7431a           PD7433a           PD8618a 
##              7730              3946              2012              1357 
##           PD8622a           PD8623a      TCGA-A1-A0SM      TCGA-A2-A04P 
##              1733              1694              2197              9645 
##      TCGA-A2-A04Q      TCGA-A2-A04T      TCGA-A2-A04X      TCGA-A2-A0CM 
##              2484              7800              3956              7954 
##      TCGA-A2-A0D0      TCGA-A2-A0D1      TCGA-A2-A0D2      TCGA-A2-A0EY 
##              7874              4285              7872             20698 
##      TCGA-A2-A0YG      TCGA-A7-A0CE      TCGA-A8-A075      TCGA-A8-A07B 
##              4817              8382              7938              6547 
##      TCGA-A8-A07I      TCGA-A8-A08B      TCGA-A8-A08L      TCGA-A8-A08S 
##              4567              4263             22281              4411 
##      TCGA-A8-A092      TCGA-A8-A094      TCGA-A8-A09I      TCGA-A8-A09X 
##              7656             20919             14011              4516 
##      TCGA-AN-A0AT      TCGA-AN-A0G0      TCGA-AO-A03L      TCGA-AO-A03N 
##              9962              3630              2827              9390 
##      TCGA-AO-A0J2      TCGA-AO-A0J4      TCGA-AO-A0J6      TCGA-AO-A0JM 
##              8238              7386              9513              3629 
##      TCGA-AQ-A04J      TCGA-AR-A0TX      TCGA-B6-A0I2      TCGA-B6-A0I6 
##              5352             30398              4662             10182 
##      TCGA-B6-A0IJ      TCGA-B6-A0IQ      TCGA-B6-A0RE      TCGA-B6-A0RT 
##             13794              5605             10993              4868 
##      TCGA-B6-A0RU      TCGA-BH-A0B3      TCGA-BH-A0B9      TCGA-BH-A0E0 
##              4011              2191              2890              3294 
##      TCGA-BH-A0WA      TCGA-BH-A18R      TCGA-BH-A18U      TCGA-C8-A12L 
##              7248              3765             12126              6710 
##      TCGA-C8-A12Q      TCGA-C8-A130      TCGA-E2-A14P      TCGA-E2-A152 
##             11618              3939              8353             13271 
##      TCGA-E2-A15E      TCGA-E2-A15H      TCGA-GM-A2DF     001-0002-03TD 
##              2709              1938              3422               955 
##     003-0005-09TD        012-02-1TD               125               128 
##              1087               990              1843              1701 
##               137               141               151               178 
##              2024              3228              2497              3061 
##               192                26               277               282 
##              3197              1935               598              1901 
##               294               306               308               318 
##               564              1732              2298              1308 
##               342               343               367               393 
##              1986              1689              2589              2191 
##               467               473               477               519 
##              2985              2199              3128              1641 
##               523               564      CLL4-ARTICLE      TCGA-A6-2680 
##              3456               821              1009             10876 
##      TCGA-A6-2681      TCGA-A6-2683      TCGA-A6-6141      TCGA-AA-3516 
##             13941             25529            225030            150924 
##      TCGA-AA-3555      TCGA-AA-3666      TCGA-AA-3956      TCGA-AA-A01R 
##            252803             27457             16278            120682 
##      TCGA-AA-A01S      TCGA-AA-A01T      TCGA-AA-A02O      TCGA-AA-A02Y 
##              6684             12814             19877             12467 
##      TCGA-AA-A03F      TCGA-AF-2691      TCGA-AG-3574      TCGA-AG-3582 
##             14692             23279              8627              6727 
##      TCGA-AG-3885      TCGA-AG-3896      TCGA-AG-3901      TCGA-AG-4015 
##             10152             13864              8515             12661 
##      TCGA-AG-A032      TCGA-D5-6540       apollo1_new          apollo10 
##              7236             85759              6688             18230 
##          apollo11          apollo12          apollo13          apollo14 
##             14105             13200              7925             22276 
##          apollo15          apollo16          apollo17          apollo19 
##             17799             87606             17914             28620 
##           apollo2          apollo20          apollo21          apollo22 
##             11828             18049             12992             27974 
##          apollo23          apollo24          apollo25           apollo3 
##             25686             86137              5123              9164 
##           apollo6           apollo7           apollo8           apollo9 
##              5463             33824              4526            264557 
## CGP_donor_GC00002 CGP_donor_GC00014 CGP_donor_GC00015 CGP_donor_GC00017 
##             30100              1754              4754              6268 
## CGP_donor_GC00018 CGP_donor_GC00019 CGP_donor_GC00020 CGP_donor_GC00027 
##              2304              8111             18526              1259 
## CGP_donor_GC00028 CGP_donor_GC00029 CGP_donor_GC00030 CGP_donor_GC00031 
##              3752              9525              1132             55172 
## CGP_donor_GC00033 CGP_donor_GC00037 CGP_donor_GC00038 CGP_donor_GC00039 
##             14105              2017              9376             22213 
## CGP_donor_GC00047 CGP_donor_GC00048 CGP_donor_GC00049 CGP_donor_GC00050 
##             12779             14744              8174              3351 
## CGP_donor_GC00051 CGP_donor_GC00052 CGP_donor_GC00053 CGP_donor_GC00054 
##             14357             34962             13444             11154 
##         HK-pfg005         HK-pfg008         HK-pfg022         HK-pfg030 
##              1023            103405              9321             15795 
##         HK-pfg031         HK-pfg032         HK-pfg034         HK-pfg035 
##              1528              5847             20996              8207 
##         HK-pfg036         HK-pfg038         HK-pfg039         HK-pfg043 
##              3928             17010              7431             59461 
##         HK-pfg050         HK-pfg052         HK-pfg054         HK-pfg057 
##              2308              6055             24378             39194 
##         HK-pfg058         HK-pfg059         HK-pfg060         HK-pfg062 
##             15104             35611             19495             17395 
##         HK-pfg064         HK-pfg065         HK-pfg068         HK-pfg069 
##             12645             21725             92284             18423 
##         HK-pfg072         HK-pfg073         HK-pfg076         HK-pfg081 
##             17806             10990             36577             16456 
##         HK-pfg082         HK-pfg088         HK-pfg089         HK-pfg092 
##              5620             14292             21753             49544 
##         HK-pfg097         HK-pfg103         HK-pfg104         HK-pfg105 
##              7731             16767              7203             19221 
##         HK-pfg106         HK-pfg107         HK-pfg108         HK-pfg115 
##              9730             19610             32188             15505 
##         HK-pfg116         HK-pfg118         HK-pfg119         HK-pfg120 
##             28492              9910             17069             26231 
##         HK-pfg121         HK-pfg122         HK-pfg124         HK-pfg125 
##             10164             74549             11220             16112 
##         HK-pfg127         HK-pfg129         HK-pfg130         HK-pfg132 
##             48082             28085              9683              2201 
##         HK-pfg135         HK-pfg136         HK-pfg142         HK-pfg143 
##              8849              3422              1814             50372 
##         HK-pfg144         HK-pfg145         HK-pfg146         HK-pfg151 
##             18087             18608             30020              3353 
##         HK-pfg156         HK-pfg157         HK-pfg160         HK-pfg164 
##              1093              7050             63918              2403 
##         HK-pfg166         HK-pfg167         HK-pfg173         HK-pfg180 
##              5792              8659             12942             27601 
##         HK-pfg181         HK-pfg182         HK-pfg205         HK-pfg212 
##             98316             10649             12931             47770 
##         HK-pfg213         HK-pfg217         HK-pfg220         HK-pfg222 
##              3520             12923              3076             16503 
##         HK-pfg228         HK-pfg258         HK-pfg272         HK-pfg277 
##              4095              7114             36743             15265 
##         HK-pfg282         HK-pfg311         HK-pfg316         HK-pfg317 
##              7316             14764             10097              6803 
##         HK-pfg344         HK-pfg373         HK-pfg375         HK-pfg378 
##             15917             10631              3344              7930 
##         HK-pfg398         HK-pfg413         HK-pfg416         HK-pfg424 
##              1018             14277              4479              6381 
##        tan2000639       tan21080055       tan76629543         tan980401 
##             19888              8447             35245              5061 
##         tan980417         tan980437         tan980447         tan990275 
##              6038             32643             12876             13719 
##         tan990489      TCGA-BR-4255      TCGA-BR-4280      TCGA-BR-6452 
##             60905              3655             56130            278055 
##      TCGA-BR-6456      TCGA-BR-6564      TCGA-BR-7722      TCGA-BR-8373 
##              6057              2225              4939             16620 
##      TCGA-BR-8381      TCGA-BR-8486      TCGA-BR-8682      TCGA-BR-8690 
##              8214              4826              7212             21719 
##      TCGA-BR-A4J4      TCGA-CD-5799      TCGA-CD-5802      TCGA-CD-8529 
##              8681              3964              4771             22125 
##      TCGA-CG-4442      TCGA-CG-4443      TCGA-CG-4474      TCGA-CG-5723 
##             96689              9925              9525            101427 
##      TCGA-CG-5724      TCGA-CG-5730      TCGA-D7-5579      TCGA-D7-6518 
##             26467             15607             18994              6070 
##      TCGA-D7-6519      TCGA-D7-6527      TCGA-D7-6528      TCGA-D7-6815 
##              5400             54771             48481             11669 
##      TCGA-D7-6822      TCGA-D7-8570      TCGA-D7-A4YX      TCGA-EQ-5647 
##             61132             11952              8188              9670 
##      TCGA-F1-6177      TCGA-F1-6875      TCGA-FP-7998      TCGA-HF-7136 
##             73327             11279              3240             10781 
##      TCGA-HU-8245      TCGA-HU-8608      TCGA-HU-A4G6      TCGA-HU-A4H0 
##              7448              8423             10837             21876 
##      TCGA-IN-7806      TCGA-BA-4076      TCGA-BA-4077      TCGA-BA-5153 
##              8882             64796             24722              3003 
##      TCGA-BA-6873      TCGA-BA-A4IH      TCGA-BB-4225      TCGA-CN-4737 
##              5119              3657              5561              4475 
##      TCGA-CN-4741      TCGA-CN-5365      TCGA-CN-5374      TCGA-CR-5250 
##             12375              6760             20355              3333 
##      TCGA-CR-6467      TCGA-CR-6470      TCGA-CR-6472      TCGA-CR-6480 
##              3154              2849             42043              7068 
##      TCGA-CR-6482      TCGA-CR-6487      TCGA-CR-7385      TCGA-CR-7391 
##              3713             12231              3978               310 
##      TCGA-CR-7404      TCGA-CV-5442      TCGA-CV-5443      TCGA-CV-6433 
##             11544             26712              4745              5467 
##      TCGA-CV-6961      TCGA-CV-7090      TCGA-CV-7100      TCGA-CV-7180 
##             45692              5123              2766              8035 
##      TCGA-CV-7255      TCGA-CV-7416      TCGA-KL-8323      TCGA-KL-8325 
##              9499              8336              2226               108 
##      TCGA-KL-8341      TCGA-KL-8342      TCGA-KM-8440      TCGA-KN-8418 
##                92               187               322                70 
##      TCGA-KN-8424      TCGA-KN-8429      TCGA-KN-8434      TCGA-KN-8435 
##                29               169                25               923 
##      TCGA-KO-8406      TCGA-KO-8407      TCGA-KO-8411      TCGA-KO-8416 
##                90               338               277                16 
##      TCGA-KO-8417      TCGA-A3-3308      TCGA-A3-3324      TCGA-A3-3370 
##               147              6855              3944              3318 
##      TCGA-A3-3372      TCGA-A3-3387      TCGA-AK-3428      TCGA-AK-3454 
##              6574              4510              4539              3388 
##      TCGA-AK-3455      TCGA-B0-5094      TCGA-B0-5693      TCGA-B2-4101 
##              4319              6242              3049              4557 
##      TCGA-BP-4327      TCGA-BP-4781      TCGA-BP-4968      TCGA-BP-4977 
##              2745              6686              3616              3451 
##      TCGA-BP-5010      TCGA-BP-5168      TCGA-CJ-4639      TCGA-CJ-4885 
##              4793              8375              3898              4781 
##      TCGA-CJ-4899      TCGA-CJ-4918      TCGA-CJ-5682      TCGA-CJ-6033 
##              2818              6959              6328              6148 
##      TCGA-CW-6087      TCGA-CW-6093      TCGA-CZ-4856      TCGA-CZ-5454 
##              5905              7839              5604              3532 
##      TCGA-CZ-5987      TCGA-DV-5566             HX10T             HX11T 
##              3690              3956             10523              9564 
##             HX12T             HX13T             HX14T             HX15T 
##             16083             24125             11880              8907 
##             HX16T             HX17T             HX18T             HX19T 
##             10329             22851              7931             12572 
##             HX20T             HX21T             HX22T             HX23T 
##              6021             14566              3979              3937 
##             HX25T             HX28T             HX30T             HX33T 
##              4687              9120             11335             15304 
##             HX35T              HX4T              HX5T              HX9T 
##             11361              9408              9876              9219 
##         RK001_C01         RK002_C01         RK003_C01         RK004_C01 
##             20104             12998              7095             15946 
##         RK005_C01         RK006_C01         RK006_C02         RK007_C01 
##              2529             17261             19069             11577 
##         RK010_C01         RK012_C01         RK015_C01         RK016_C01 
##              7055              7174             13024              8082 
##         RK018_C01         RK019_C01         RK020_C01         RK021_C01 
##              1432             16428              5135             16377 
##         RK022_C01         RK023_C01         RK024_C01         RK025_C01 
##             12586             19534              4965              8364 
##         RK026_C01         RK027_C01         RK029_C01         RK031_C01 
##              6296             23222              7225              6984 
##         RK032_C01         RK033_C01         RK034_C01         RK035_C01 
##             11053              3924              4861             25727 
##         RK036_C01         RK037_C01         RK041_C01         RK042_C01 
##              4640              5134             20069              8447 
##         RK046_C01         RK046_C02         RK047_C01         RK048_C01 
##              8969             11339              8411             14434 
##         RK049_C01         RK050_C01         RK051_C01         RK054_C01 
##              2788              8908             11844              6322 
##         RK055_C01         RK056_C01         RK063_C01         RK067_C01 
##              1948             17116              6405             14261 
##         RK068_C01         RK069_C01         RK075_C01         RK079_C01 
##              2537              4717              8904             12543 
##         RK083_C01         RK084_C01         RK086_C01         RK089_C01 
##              8690              9000             14761              9831 
##         RK092_C01         RK098_C01         RK099_C01         RK100_C01 
##             11129              6496              6978              8898 
##         RK106_C01         RK107_C01         RK108_C01         RK109_C01 
##             12299             12011              6943              5597 
##         RK126_C01         RK130_C01         RK133_C01         RK137_C01 
##             18275              4929              5959              8343 
##         RK138_C01         RK141_C01         LU-A08-43        LUAD-2GUGK 
##              2889              5103             11920             47253 
##        LUAD-5V8LT        LUAD-AEIUF       LUAD-D02326       LUAD-E00934 
##            305665             34708             10475            115244 
##       LUAD-E01014       LUAD-E01278       LUAD-E01317        LUAD-FH5PJ 
##             24720             49496             37783             37322 
##        LUAD-QY22Z       LUAD-S00488       LUAD-S01302       LUAD-S01331 
##             76262             76974            131255             45740 
##       LUAD-S01341       LUAD-S01345       LUAD-S01346       LUAD-S01356 
##             16920             41426             87912             71297 
##       LUAD-S01381       LUAD-S01404       LUAD-S01405       LUAD-S01467 
##             29245             35801             51249             47868 
##       LUAD-S01478        LUAD-U6SJ7      TCGA-05-4389      TCGA-05-4395 
##             81649             32240             21340             40408 
##      TCGA-05-4396      TCGA-05-4397      TCGA-05-4398      TCGA-05-4420 
##             40311             93860            104046             32025 
##      TCGA-05-4422      TCGA-05-4432      TCGA-05-5429      TCGA-38-4628 
##              4226             75763              2887             12648 
##      TCGA-38-4630      TCGA-44-2659      TCGA-44-2665      TCGA-44-2666 
##             13995             48536              3508              1924 
##      TCGA-44-6148      TCGA-49-4486      TCGA-49-4510      TCGA-49-4512 
##               418             11652              5195              4239 
##      TCGA-49-6742      TCGA-50-5066      TCGA-50-6597      TCGA-55-1594 
##             45176             40445              7476             16151 
##      TCGA-55-1596      TCGA-55-6982      TCGA-55-6986      TCGA-55-7281 
##             23402             24669              1703             62944 
##      TCGA-64-1678      TCGA-64-1680      TCGA-67-3771      TCGA-67-3772 
##             56781              2796            137860              2796 
##      TCGA-67-6215      TCGA-73-4659      TCGA-73-4666      TCGA-75-5147 
##              9082             23611             40372              4621 
##      TCGA-75-6203      TCGA-78-7143      TCGA-78-7146      TCGA-78-7156 
##              1268               849             42012             25209 
##      TCGA-78-7535      TCGA-91-6840      TCGA-21-1076      TCGA-21-1078 
##             17679             15113             41528              5036 
##      TCGA-21-1082      TCGA-22-1016      TCGA-34-2596      TCGA-34-2600 
##             42331             49685             42150             50905 
##      TCGA-43-3394      TCGA-43-3920      TCGA-56-1622      TCGA-60-2695 
##             29910             43366             40603             26507 
##      TCGA-60-2698      TCGA-60-2711      TCGA-60-2713      TCGA-60-2719 
##            114374             18063             34938             23703 
##      TCGA-60-2722      TCGA-60-2724      TCGA-60-2726      TCGA-66-2756 
##             50593             50729             61534             72091 
##      TCGA-66-2757      TCGA-66-2766           4101316           4105105 
##             33167             56128              2391              3644 
##           4108101           4112512           4116738           4119027 
##              3379              2202              8062              2587 
##           4121361           4125240           4133511           4135350 
##              6335              2106              3758             13325 
##           4142267           4158726           4159170           4163639 
##              1636              4360              3459             39117 
##           4175837           4177856           4182393           4189200 
##              4694              3807              1644              3711 
##           4189998           4190495           4193278           4194218 
##              3729              4946              2791              2192 
##           4194891                G1           LFS_MB1           LFS_MB2 
##              1829             16139              2277              1816 
##           LFS_MB4               MB1             MB101             MB102 
##              1373               458              4268              1864 
##             MB104             MB106             MB107             MB108 
##              2487               442              1963               601 
##             MB110             MB112             MB113             MB114 
##              3687              1243              1216               558 
##             MB115             MB117             MB119              MB12 
##               656              1114               262               236 
##             MB121             MB122             MB124             MB125 
##               634              1182              1796              3257 
##             MB126             MB127             MB128             MB129 
##              3643              2881              9867              1339 
##             MB130             MB131             MB132             MB134 
##              1136              1274               320               692 
##             MB139              MB15              MB16              MB17 
##               649               977               398               725 
##              MB18              MB19               MB2              MB20 
##              1217               735               393               952 
##              MB21              MB23              MB24              MB26 
##               392              2174               308               903 
##              MB28               MB3              MB31              MB32 
##                59              1086              1839               605 
##              MB34              MB35              MB36              MB37 
##              3510               338              1363                95 
##              MB38              MB39              MB40              MB45 
##               785               235               126              1720 
##              MB46              MB49               MB5              MB50 
##               753               769               620              1301 
##              MB51             MB518              MB53              MB56 
##               523              1219              1738              2873 
##              MB57              MB58              MB59               MB6 
##               833              1714              2469              1167 
##              MB60              MB61             MB612              MB63 
##              1347              2810               349              2118 
##              MB64              MB66              MB67              MB69 
##               985              2035              1042              2286 
##               MB7              MB70              MB74              MB75 
##               816              1736              2362              2232 
##              MB77              MB78              MB79               MB8 
##              1893              3268              1833               817 
##             MB800              MB81              MB82              MB83 
##              1132              4368              1755               860 
##              MB84              MB85              MB86              MB88 
##              1133               232               520               309 
##              MB89               MB9              MB90              MB91 
##               598               607               326              1023 
##              MB92              MB94              MB95              MB96 
##              1785              2157               312               370 
##              MB98              MB99      TCGA-13-0723      TCGA-13-0890 
##              1013               759              3936              4642 
##      TCGA-13-1411      TCGA-24-0980      TCGA-24-1103         APGI_1839 
##              4530              3886              6107              5502 
##         APGI_1840         APGI_1956         APGI_1992         APGI_2000 
##             12906              5088             10167              8281 
##         APGI_2051         APGI_2057         APGI_2060         APGI_2119 
##              5509             15580              6992              4318 
##         APGI_2137         APGI_2150         APGI_2157         APGI_2179 
##             11921              1301              7500             15323 
##         APGI_2202         APGI_2353      TCGA-CH-5750      TCGA-CH-5763 
##              4147              8051              3615               394 
##      TCGA-CH-5771      TCGA-CH-5788      TCGA-CH-5789      TCGA-EJ-5503 
##              2303              3361               104               161 
##      TCGA-EJ-5506      TCGA-EJ-7791      TCGA-G9-6336      TCGA-G9-6365 
##               170              1345              1550              2519 
##      TCGA-G9-6370      TCGA-G9-7522      TCGA-HC-7075      TCGA-HC-7079 
##                95              1090              2899                58 
##      TCGA-HC-7233      TCGA-HC-7737      TCGA-HC-7740      TCGA-HC-7744 
##              2765              1385               892              1852 
##      TCGA-HC-8258      TCGA-HI-7169      TCGA-D9-A148      TCGA-DA-A1IC 
##               804              2500             46655            141622 
##      TCGA-EB-A24D      TCGA-EE-A29B      TCGA-EE-A2M5      TCGA-EE-A2MI 
##             88859             94850            371487            232209 
##      TCGA-EE-A3J5      TCGA-EE-A3JI      TCGA-ER-A19E      TCGA-ER-A19T 
##            337346            163855             71067              4571 
##      TCGA-ER-A2NF      TCGA-ER-A3ES      TCGA-FS-A1ZD      TCGA-FS-A1ZK 
##              3070              1305             39120            216156 
##      TCGA-FS-A1ZP      TCGA-GN-A26C      TCGA-BJ-A191      TCGA-DJ-A13W 
##             91750            344754              2065              1015 
##      TCGA-EL-A3H1      TCGA-EL-A3TB      TCGA-EM-A3AL      TCGA-EM-A3AQ 
##              2005               903               774               871 
##      TCGA-FE-A3PD      TCGA-FK-A3S3      TCGA-FK-A3SE      TCGA-A5-A0G9 
##               968               539               655             24043 
##      TCGA-A5-A0GA      TCGA-A5-A0GE      TCGA-A5-A0GG      TCGA-AP-A052 
##             25028              3536             17197              3692 
##      TCGA-AP-A053      TCGA-AP-A054      TCGA-AP-A05A      TCGA-AP-A05D 
##              4410            111303              6817              3692 
##      TCGA-AP-A0L8      TCGA-AP-A0L9      TCGA-AP-A0LD      TCGA-AP-A0LE 
##              6663              8987             34912             41318 
##      TCGA-AP-A0LH      TCGA-AP-A0LI      TCGA-AP-A0LL      TCGA-AP-A0LO 
##              3766             14150              3298            337569 
##      TCGA-AX-A05S      TCGA-AX-A1CI      TCGA-B5-A0JN      TCGA-B5-A11G 
##             31021              4131             15116             62743 
##      TCGA-B5-A11H      TCGA-BK-A0CC      TCGA-BS-A0TC      TCGA-BS-A0TD 
##             64733              6429            286464              3861 
##      TCGA-BS-A0TE      TCGA-BS-A0TG      TCGA-D1-A16G      TCGA-EY-A1GW 
##             38592              4031              4893              4946
```

Set cutoff as 200000 mutations, remove samples with more than 200000 mutation count.

```
sum(ind.maf2>=200000) # 12, 2 of which are gastric samples
```

```
## [1] 12
```

```
maf.total=c(maf.pancan,maf.gastric) # 15715077
maf.total=maf.total[-which(maf.total$ctype=="all")] # 15707250
maf.total$ctype=as.character(maf.total$ctype)
maf.total$sid=as.character(maf.total$sid)
maf.total$ctype=factor(maf.total$ctype)
maf.total2=maf.total[which(! maf.total$sid %in% names(ind.maf2[which(ind.maf2>=200000)]))] # 12255155
length(unique(maf.total2$ctype)) # 22
```

```
## [1] 22
```

```
ind.maf4=split(maf.total2, maf.total2$ctype) # split by cancer type
sample.count4=sapply(ind.maf4, function(x) {length(unique(x$sid))}) # 1032
print(sample.count4) # number of samples per cancer type
```

```
##     aml    astr    blca   brain    brca     cll     crc gastric    hnsc 
##       7     101      20      43     172      28      21     185      29 
##    kich    kirc    lihc    luad    lusc    lymp    medu      ov    paad 
##      15      29      88      63      20      24     100       5      15 
##    prad    skcm    thca    ucec 
##      20      11       9      27
```

```
# Find the number of mutated samples at the CBS hotspots (11)
ovl=findOverlaps(maf.total2,ctcf.hotspot) # 128
pancan.cbs=maf.total2[queryHits(ovl)]
```

Tissue specific CBS

```
ctcf.motif=read.table("fimo_all.txt",sep="\t")
unique(ctcf.motif$V4-ctcf.motif$V3)+1 # 19
ctcf.motif=GRanges(seqnames=ctcf.motif$V2,IRanges(start=ctcf.motif$V3,end=ctcf.motif$V4),pval=ctcf.motif$V7,qval=ctcf.motif$V8,dir=ctcf.motif$V5,motif=ctcf.motif$V9)
length(ctcf.motif)

ctcf.peak=import("CTCF.bw")
sum(ctcf.peak$score>0)

z=findOverlaps(ctcf.peak,ctcf.motif) # 183666
ctcf.peak.ovl=ctcf.peak[queryHits(z)]
ctcf.peak.ovl=as.data.frame(ctcf.peak.ovl)
ctcf.peak.ovl=unique(ctcf.peak.ovl) # 157222
ctcf.motif.ovl=ctcf.motif[subjectHits(z)]
ctcf.motif.ovl=as.data.frame(ctcf.motif.ovl)
ctcf.motif.ovl=unique(ctcf.motif.ovl) # 125228
ctcf.motif.ovl=GRanges(seqnames=ctcf.motif.ovl$seqnames,IRanges(start=ctcf.motif.ovl$start,end=ctcf.motif.ovl$end),pval=ctcf.motif.ovl$pval,qval=ctcf.motif.ovl$qval,dir=ctcf.motif.ovl$dir,motif=ctcf.motif.ovl$motif)

# ### Overlap between ctcf_motif, tf binding and dnase for each cancer type, if tissue cant be matched, use generic filter
# colorectal
dnase.peak=import("E084-DNase.hotspot.all.peaks.bed")
sum(dnase.peak$score>0)
dnase.peak=GRanges(seqnames=seqnames(dnase.peak),IRanges(start=start(dnase.peak),end=end(dnase.peak)))

dnase.fdr=read.table("E084-DNase.hotspot.fdr0.01.peaks.bed")
dnase.fdr=GRanges(seqnames=dnase.fdr$V1,IRanges(start=dnase.fdr$V2,end=dnase.fdr$V3))

dnase.mac=read.table("E084-DNase.macs2.narrowPeak")
dnase.mac=GRanges(seqnames=dnase.mac$V1,IRanges(start=dnase.mac$V2,dnase.mac$V3))

dnase=c(dnase.peak,dnase.fdr,dnase.mac)
dnase=reduce(dnase)
length(dnase)

z=findOverlaps(dnase,ctcf.motif.ovl)
dnase.ovl=dnase[queryHits(z)]
dnase.ovl=as.data.frame(dnase.ovl)
dnase.ovl=unique(dnase.ovl)
motif.ovl=ctcf.motif.ovl[subjectHits(z)]
motif.ovl=as.data.frame(motif.ovl)
motif.ovl=unique(motif.ovl) # 47308
roi.crc=with(motif.ovl,GRanges(seqnames,IRanges(start,end)))

# liver hepatocellular
dnase.mac=read.table("E118-DNase.macs2.narrowPeak")
dnase.mac=GRanges(seqnames=dnase.mac$V1,IRanges(start=dnase.mac$V2,dnase.mac$V3))

dnase=c(dnase.mac)
dnase=reduce(dnase)
length(dnase)

z=findOverlaps(dnase,ctcf.motif.ovl)
dnase.ovl=dnase[queryHits(z)]
dnase.ovl=as.data.frame(dnase.ovl)
dnase.ovl=unique(dnase.ovl)
motif.ovl=ctcf.motif.ovl[subjectHits(z)]
motif.ovl=as.data.frame(motif.ovl)
motif.ovl=unique(motif.ovl) # 58795
roi.lihc=with(motif.ovl,GRanges(seqnames,IRanges(start,end)))

# pancreas
dnase.peak=import("E098-DNase.hotspot.all.peaks.bed")
sum(dnase.peak$score>0)
dnase.peak=GRanges(seqnames=seqnames(dnase.peak),IRanges(start=start(dnase.peak),end=end(dnase.peak)))

dnase.fdr=read.table("E098-DNase.hotspot.fdr0.01.peaks.bed")
dnase.fdr=GRanges(seqnames=dnase.fdr$V1,IRanges(start=dnase.fdr$V2,end=dnase.fdr$V3))

dnase.mac=read.table("E098-DNase.macs2.narrowPeak")
dnase.mac=GRanges(seqnames=dnase.mac$V1,IRanges(start=dnase.mac$V2,dnase.mac$V3))

dnase=c(dnase.peak,dnase.fdr,dnase.mac)
dnase=reduce(dnase)
length(dnase)

z=findOverlaps(dnase,ctcf.motif.ovl)
dnase.ovl=dnase[queryHits(z)]
dnase.ovl=as.data.frame(dnase.ovl)
dnase.ovl=unique(dnase.ovl)
motif.ovl=ctcf.motif.ovl[subjectHits(z)]
motif.ovl=as.data.frame(motif.ovl)
motif.ovl=unique(motif.ovl) # 54840
roi.paad=with(motif.ovl,GRanges(seqnames,IRanges(start,end)))

# lung adeno/ lung squamous cell
dnase.peak=import("E088-DNase.hotspot.all.peaks.bed")
sum(dnase.peak$score>0)
dnase.peak=GRanges(seqnames=seqnames(dnase.peak),IRanges(start=start(dnase.peak),end=end(dnase.peak)))

dnase.fdr=read.table("E088-DNase.hotspot.fdr0.01.peaks.bed")
dnase.fdr=GRanges(seqnames=dnase.fdr$V1,IRanges(start=dnase.fdr$V2,end=dnase.fdr$V3))

dnase.mac=read.table("E088-DNase.macs2.narrowPeak")
dnase.mac=GRanges(seqnames=dnase.mac$V1,IRanges(start=dnase.mac$V2,dnase.mac$V3))

dnase=c(dnase.peak,dnase.fdr,dnase.mac)
dnase=reduce(dnase)
length(dnase)

z=findOverlaps(dnase,ctcf.motif.ovl)
dnase.ovl=dnase[queryHits(z)]
dnase.ovl=as.data.frame(dnase.ovl)
dnase.ovl=unique(dnase.ovl)
motif.ovl=ctcf.motif.ovl[subjectHits(z)]
motif.ovl=as.data.frame(motif.ovl)
motif.ovl=unique(motif.ovl) # 70758
roi.luad=with(motif.ovl,GRanges(seqnames,IRanges(start,end)))

# lymphoma
dnase.peak=import("E032-DNase.hotspot.all.peaks.bed")
sum(dnase.peak$score>0)
dnase.peak=GRanges(seqnames=seqnames(dnase.peak),IRanges(start=start(dnase.peak),end=end(dnase.peak)))

dnase.fdr=read.table("E032-DNase.hotspot.fdr0.01.peaks.bed")
dnase.fdr=GRanges(seqnames=dnase.fdr$V1,IRanges(start=dnase.fdr$V2,end=dnase.fdr$V3))

dnase.mac=read.table("E032-DNase.macs2.narrowPeak")
dnase.mac=GRanges(seqnames=dnase.mac$V1,IRanges(start=dnase.mac$V2,dnase.mac$V3))

dnase=c(dnase.peak,dnase.fdr,dnase.mac)
dnase=reduce(dnase)
length(dnase)

z=findOverlaps(dnase,ctcf.motif.ovl)
dnase.ovl=dnase[queryHits(z)]
dnase.ovl=as.data.frame(dnase.ovl)
dnase.ovl=unique(dnase.ovl)
motif.ovl=ctcf.motif.ovl[subjectHits(z)]
motif.ovl=as.data.frame(motif.ovl)
motif.ovl=unique(motif.ovl) # 56632
roi.lymp=with(motif.ovl,GRanges(seqnames,IRanges(start,end)))

# breast
dnase.peak=import("E028-DNase.hotspot.all.peaks.bed")
sum(dnase.peak$score>0)
dnase.peak=GRanges(seqnames=seqnames(dnase.peak),IRanges(start=start(dnase.peak),end=end(dnase.peak)))

dnase.fdr=read.table("E028-DNase.hotspot.fdr0.01.peaks.bed")
dnase.fdr=GRanges(seqnames=dnase.fdr$V1,IRanges(start=dnase.fdr$V2,end=dnase.fdr$V3))

dnase.mac=read.table("E028-DNase.macs2.narrowPeak")
dnase.mac=GRanges(seqnames=dnase.mac$V1,IRanges(start=dnase.mac$V2,dnase.mac$V3))

dnase=c(dnase.peak,dnase.fdr,dnase.mac)
dnase=reduce(dnase)
length(dnase)

z=findOverlaps(dnase,ctcf.motif.ovl)
dnase.ovl=dnase[queryHits(z)]
dnase.ovl=as.data.frame(dnase.ovl)
dnase.ovl=unique(dnase.ovl)
motif.ovl=ctcf.motif.ovl[subjectHits(z)]
motif.ovl=as.data.frame(motif.ovl)
motif.ovl=unique(motif.ovl) # 58075
roi.brca=with(motif.ovl,GRanges(seqnames,IRanges(start,end)))

# skin cutaneous
dnase.peak=import("E059-DNase.hotspot.all.peaks.bed")
sum(dnase.peak$score>0)
dnase.peak=GRanges(seqnames=seqnames(dnase.peak),IRanges(start=start(dnase.peak),end=end(dnase.peak)))

dnase.fdr=read.table("E059-DNase.hotspot.fdr0.01.peaks.bed")
dnase.fdr=GRanges(seqnames=dnase.fdr$V1,IRanges(start=dnase.fdr$V2,end=dnase.fdr$V3))

dnase.mac=read.table("E059-DNase.macs2.narrowPeak")
dnase.mac=GRanges(seqnames=dnase.mac$V1,IRanges(start=dnase.mac$V2,dnase.mac$V3))

dnase=c(dnase.peak,dnase.fdr,dnase.mac)
dnase=reduce(dnase)
length(dnase)

z=findOverlaps(dnase,ctcf.motif.ovl)
dnase.ovl=dnase[queryHits(z)]
dnase.ovl=as.data.frame(dnase.ovl)
dnase.ovl=unique(dnase.ovl)
motif.ovl=ctcf.motif.ovl[subjectHits(z)]
motif.ovl=as.data.frame(motif.ovl)
motif.ovl=unique(motif.ovl) # 62179
roi.skcm=with(motif.ovl,GRanges(seqnames,IRanges(start,end)))

# medulloblast/brain
dnase.peak=import("E081-DNase.hotspot.all.peaks.bed")
sum(dnase.peak$score>0)
dnase.peak=GRanges(seqnames=seqnames(dnase.peak),IRanges(start=start(dnase.peak),end=end(dnase.peak)))

dnase.fdr=read.table("E081-DNase.hotspot.fdr0.01.peaks.bed")
dnase.fdr=GRanges(seqnames=dnase.fdr$V1,IRanges(start=dnase.fdr$V2,end=dnase.fdr$V3))

dnase.mac=read.table("E081-DNase.macs2.narrowPeak")
dnase.mac=GRanges(seqnames=dnase.mac$V1,IRanges(start=dnase.mac$V2,dnase.mac$V3))

dnase=c(dnase.peak,dnase.fdr,dnase.mac)
dnase=reduce(dnase)
length(dnase)

z=findOverlaps(dnase,ctcf.motif.ovl)
dnase.ovl=dnase[queryHits(z)]
dnase.ovl=as.data.frame(dnase.ovl)
dnase.ovl=unique(dnase.ovl)
motif.ovl=ctcf.motif.ovl[subjectHits(z)]
motif.ovl=as.data.frame(motif.ovl)
motif.ovl=unique(motif.ovl) # 54201
roi.medu=with(motif.ovl,GRanges(seqnames,IRanges(start,end)))

# kidney renal/kidney chromophobe
dnase.peak=import("E086-DNase.hotspot.all.peaks.bed")
sum(dnase.peak$score>0)
dnase.peak=GRanges(seqnames=seqnames(dnase.peak),IRanges(start=start(dnase.peak),end=end(dnase.peak)))

dnase.fdr=read.table("E086-DNase.hotspot.fdr0.01.peaks.bed")
dnase.fdr=GRanges(seqnames=dnase.fdr$V1,IRanges(start=dnase.fdr$V2,end=dnase.fdr$V3))

dnase.mac=read.table("E086-DNase.macs2.narrowPeak")
dnase.mac=GRanges(seqnames=dnase.mac$V1,IRanges(start=dnase.mac$V2,dnase.mac$V3))

dnase=c(dnase.peak,dnase.fdr,dnase.mac)
dnase=reduce(dnase)
length(dnase)

z=findOverlaps(dnase,ctcf.motif.ovl)
dnase.ovl=dnase[queryHits(z)]
dnase.ovl=as.data.frame(dnase.ovl)
dnase.ovl=unique(dnase.ovl)
motif.ovl=ctcf.motif.ovl[subjectHits(z)]
motif.ovl=as.data.frame(motif.ovl)
motif.ovl=unique(motif.ovl) # 70382
roi.kirc=with(motif.ovl,GRanges(seqnames,IRanges(start,end)))

# chronic leukemia
dnase.peak=import("E032-DNase.hotspot.all.peaks.bed")
sum(dnase.peak$score>0)
dnase.peak=GRanges(seqnames=seqnames(dnase.peak),IRanges(start=start(dnase.peak),end=end(dnase.peak)))

dnase.fdr=read.table("E032-DNase.hotspot.fdr0.01.peaks.bed")
dnase.fdr=GRanges(seqnames=dnase.fdr$V1,IRanges(start=dnase.fdr$V2,end=dnase.fdr$V3))

dnase.mac=read.table("E032-DNase.macs2.narrowPeak")
dnase.mac=GRanges(seqnames=dnase.mac$V1,IRanges(start=dnase.mac$V2,dnase.mac$V3))

dnase=c(dnase.peak,dnase.fdr,dnase.mac)
dnase=reduce(dnase)
length(dnase)

z=findOverlaps(dnase,ctcf.motif.ovl)
dnase.ovl=dnase[queryHits(z)]
dnase.ovl=as.data.frame(dnase.ovl)
dnase.ovl=unique(dnase.ovl)
motif.ovl=ctcf.motif.ovl[subjectHits(z)]
motif.ovl=as.data.frame(motif.ovl)
motif.ovl=unique(motif.ovl) # 56632
roi.cll=with(motif.ovl,GRanges(seqnames,IRanges(start,end)))

# astrocytoma
dnase.mac=read.table("E125-DNase.macs2.narrowPeak")
dnase.mac=GRanges(seqnames=dnase.mac$V1,IRanges(start=dnase.mac$V2,dnase.mac$V3))

dnase=c(dnase.mac)
dnase=reduce(dnase)
length(dnase)

z=findOverlaps(dnase,ctcf.motif.ovl)
dnase.ovl=dnase[queryHits(z)]
dnase.ovl=as.data.frame(dnase.ovl)
dnase.ovl=unique(dnase.ovl)
motif.ovl=ctcf.motif.ovl[subjectHits(z)]
motif.ovl=as.data.frame(motif.ovl)
motif.ovl=unique(motif.ovl) # 50731
roi.astr=with(motif.ovl,GRanges(seqnames,IRanges(start,end)))

# aml
dnase.mac=read.table("E123-DNase.macs2.narrowPeak")
dnase.mac=GRanges(seqnames=dnase.mac$V1,IRanges(start=dnase.mac$V2,dnase.mac$V3))

dnase=c(dnase.mac)
dnase=reduce(dnase)
length(dnase)

z=findOverlaps(dnase,ctcf.motif.ovl)
dnase.ovl=dnase[queryHits(z)]
dnase.ovl=as.data.frame(dnase.ovl)
dnase.ovl=unique(dnase.ovl)
motif.ovl=ctcf.motif.ovl[subjectHits(z)]
motif.ovl=as.data.frame(motif.ovl)
motif.ovl=unique(motif.ovl) # 57533
roi.aml=with(motif.ovl,GRanges(seqnames,IRanges(start,end)))

# extend regions by 5bp
roi.ctcf.ext=roi.ctcf+5
roi.crc.ext=roi.crc+5
roi.lihc.ext=roi.lihc+5
roi.paad.ext=roi.paad+5
roi.luad.ext=roi.luad+5
roi.lymp.ext=roi.lymp+5
roi.brca.ext=roi.brca+5
roi.skcm.ext=roi.skcm+5
roi.medu.ext=roi.medu+5
roi.kirc.ext=roi.kirc+5
roi.cll.ext=roi.cll+5
roi.astr.ext=roi.astr+5
roi.aml.ext=roi.aml+5

ctcf.all=list(roi.ctcf.ext,roi.crc.ext,roi.lihc.ext,roi.paad.ext,roi.luad.ext,roi.luad.ext,
              roi.lymp.ext,roi.brca.ext,roi.skcm.ext,roi.medu.ext,roi.kirc.ext,roi.kirc.ext,
              roi.cll.ext,roi.medu.ext,roi.astr.ext,roi.aml.ext)
names(ctcf.all)=c("gastric","crc","lihc","paad","luad","lusc","lymp","brca","skcm","medu",
                  "kirc","kich","cll","brain","astr","aml") #16 ctype
# save summarized output to rds
saveRDS(ctcf.all,file="suppfig11_ctcf_all.RDS")
```

Read in summarized file

```
ctcf.all=readRDS("suppfig11_ctcf_all.RDS") #16
```

```
maf.total2=maf.total2[which(!maf.total2$ctype %in% c("blca","hnsc","prad","thca","ucec","ov"))] #10909199, 16 ctype
maf.total2$ctype=as.character(maf.total2$ctype)
maf.total2=split(maf.total2,maf.total2$ctype)

pancan.cbs=mclapply(names(maf.total2),FUN=function(x){
  ovl=findOverlaps(maf.total2[[x]],ctcf.all[[x]])
  maf.total2[[x]][unique(queryHits(ovl))]
},mc.cores=1) #mc.cores=5
names(pancan.cbs)=names(maf.total2)
cbs.count=sapply(pancan.cbs, function(x) {length(unique(x$sid))}) # number of samples per cancer type that overlapped 

cbs.mut.count=sapply(pancan.cbs, function(x) {length(x)}) # number of mutations per cancaer type that overlapped with CBS 

mut.rate=sapply(names(cbs.mut.count),FUN=function(x){
  cbs.mut.count[x]/(sum(width(ctcf.all[[x]]))*sample.count4[x])
})

df2=data.frame(ctype=names(cbs.mut.count),mut.rate=mut.rate)
df2=df2[which(!df2$ctype %in% names(sample.count4[which(sample.count4<10)])),] # 15
df2$ctype=as.character(df2$ctype)
df2$ctype=factor(df2$ctype,levels=c("astr","brain","cll","kich","kirc","medu","skcm","brca","lymp","lusc","luad","paad","lihc","crc","gastric"))

ggplot(df2,aes(x=ctype,y=mut.rate))+geom_bar(stat="identity",position=position_stack(reverse=TRUE),colour="black")+
  coord_flip()+
  theme(text = element_text(size=20),axis.text.x = element_text(size=20),legend.position="none",panel.background =element_rect(fill = "white", color="black"))
```
