# Supplementary material for: Mutation hotspots at CTCF binding sites coupled to chromosomal instability in gastrointestinal cancers
Source: Nat Commun. 2018 Apr 18;9:1520. doi: 10.1038/s41467-018-03828-2 (PMC5906695; doi:10.1038/s41467-018-03828-2)
Supplement: Supplementary file 8 — Supplementary Data 5 [file 41467_2018_3828_MOESM8_ESM.zip › Rmarkdowns/Supplementary Figure 2/Supplementary_Figure2_feature_models_rev.html]

Supplementary Figure 2 - SNV/indel/CTCF specific models


# Supplementary Figure 2 - SNV/indel/CTCF specific models

This is the R Markdown for Supplementary Figure 2, which consists of 3 parts.

## Figure A

SNV hotspot model

```
load("LRmodel_gastric_genome_nonMSI_prefiltered_trunc")
t=summary(LRmodel)
df=coef(t)
df=as.data.frame(df)
df$feat=rownames(df)
df=df[-1,] # remove intercept
colnames(df)=c("estimate","std.error","z.value","p.value","feat")
df=df[order(df$z.value,decreasing=TRUE),]

# save summarized output to table
write.table(df,"snv_coef.txt",quote=FALSE,row.names=TRUE,sep="\t",col.names=TRUE)
```

Read in summarized table

```
df=read.table("snv_coef.txt")
```

```
df$feat=factor(df$feat,levels=df$feat)

ggplot(df,aes(x=feat,y=z.value))+geom_bar(stat="identity")+
  theme(panel.grid.major = element_blank(),
        panel.grid.minor = element_blank(),
        panel.background = element_blank(),
        axis.line = element_line(colour="black"))+
  theme(axis.text.x = element_text(angle = 90, hjust = 1))
```

```
# colour by feat
df$feat=as.character(df$feat)
df$type=df$feat
df$type=ifelse(grepl("_E",df$type),"epigenetic features",df$type)
df$type=ifelse(grepl("_meta",df$type),"epigenetic features",df$type)
df$type=ifelse(grepl("tumor",df$type),"epigenetic features",df$type)
df$type=ifelse(grepl("three",df$type),"nucleotide context",df$type)
df$type=ifelse(grepl("five",df$type),"nucleotide context",df$type)
df$type=ifelse(grepl("one",df$type),"nucleotide context",df$type)
df$type=ifelse(df$type %in% c("SMC3","CTCF"),"epigenetic features",df$type)

df$type=factor(df$type)
df$feat=factor(df$feat,levels=df$feat)

ggplot(df,aes(x=feat,y=z.value,fill=type))+geom_bar(stat="identity")+
  theme(panel.grid.major = element_blank(),
        panel.grid.minor = element_blank(),
        panel.background = element_blank(),
        axis.line = element_line(colour="black"))+
  scale_fill_manual(values=c("#66FF00","#FFFF00","#FF0000","#FF0099","#9900FF"))+
  theme(axis.text.x = element_text(angle = 90, hjust = 1))
```

## Figure B

Indel hotspot model

```
load("LRmodel_gastric_genome_nonMSI_indels_prefiltered_trunc_poly_v2")
t=summary(LRmodel)
df=coef(t)
df=as.data.frame(df)
df$feat=rownames(df)
df=df[-1,] # remove intercept
colnames(df)=c("estimate","std.error","z.value","p.value","feat")
df=df[order(df$z.value,decreasing=TRUE),]

# save summarized output to table
write.table(df,"indel_coef.txt",quote=FALSE,sep="\t",col.names=TRUE,row.names=TRUE)
```

Read in summarized table

```
df=read.table("indel_coef.txt")
```

```
df$feat=factor(df$feat,levels=df$feat)

ggplot(df,aes(x=feat,y=z.value))+geom_bar(stat="identity")+
  theme(panel.grid.major = element_blank(),
        panel.grid.minor = element_blank(),
        panel.background = element_blank(),
        axis.line = element_line(colour="black"))+
  theme(axis.text.x = element_text(angle = 90, hjust = 1))
```

```
# colour by feat
df$feat=as.character(df$feat)
df$type=df$feat
df$type=ifelse(grepl("_E",df$type),"epigenetic features",df$type)
df$type=ifelse(grepl("_meta",df$type),"epigenetic features",df$type)
df$type=ifelse(grepl("poly",df$type),"nucleotide context",df$type)

df$type=factor(df$type)
df$feat=factor(df$feat,levels=df$feat)

ggplot(df,aes(x=feat,y=z.value,fill=type))+geom_bar(stat="identity")+
  theme(panel.grid.major = element_blank(),
        panel.grid.minor = element_blank(),
        panel.background = element_blank(),
        axis.line = element_line(colour="black"))+
  scale_fill_manual(values=c("#66FF00","#FFFF00","#FF0000","#FF0099","#9900FF"))+
  theme(axis.text.x = element_text(angle = 90, hjust = 1))
```

## Figure C

CTCF specific model

```
load("LRmodel_gastric_CTCF_union_nonMSI_subtype-5_mutsigs")
df=summary(LRmodel)$coefficients
df=as.data.frame(df)
df$feat=rownames(df)
df=df[-1,] # remove intercept
colnames(df)=c("estimate","std.error","z.value","p.value","feat")
df=df[order(df$z.value,decreasing=TRUE),]

# save summarized output to table
write.table(df,"ctcf_specific_coef_edited.txt",quote=FALSE,row.names=TRUE,sep="\t",col.names=TRUE)
```

Read in summarized table

```
df=read.table("ctcf_specific_coef_edited.txt")
```

```
df$feat=factor(df$feat,levels=df$feat)

ggplot(df,aes(x=feat,y=z.value))+geom_bar(stat="identity")+
  theme(panel.grid.major = element_blank(),
        panel.grid.minor = element_blank(),
        panel.background = element_blank(),
        axis.line = element_line(colour="black"))+
  theme(axis.text.x = element_text(angle = 90, hjust = 1))
```

```
# colour by feat
df$feat=as.character(df$feat)
df$type=df$feat
df$type=ifelse(grepl("three",df$type),"nucleotide context",df$type)
df$type=ifelse(grepl("one",df$type),"nucleotide context",df$type)
df$type=ifelse(grepl("sub",df$type),"subtype",df$type)
df$type=ifelse(grepl("mutsig",df$type),"mutsig",df$type)

df$type=factor(df$type)
df$feat=factor(df$feat,levels=df$feat)

ggplot(df,aes(x=feat,y=z.value,fill=type))+geom_bar(stat="identity")+
  theme(panel.grid.major = element_blank(),
        panel.grid.minor = element_blank(),
        panel.background = element_blank(),
        axis.line = element_line(colour="black"))+
  scale_fill_manual(values=c("#666666","#FFFF00","#FF0000","#FF9933","#FF0099","#9900FF","#3399FF"))+
  theme(axis.text.x = element_text(angle = 90, hjust = 1))
```
