# Supplementary material for: Mutation hotspots at CTCF binding sites coupled to chromosomal instability in gastrointestinal cancers
Source: Nat Commun. 2018 Apr 18;9:1520. doi: 10.1038/s41467-018-03828-2 (PMC5906695; doi:10.1038/s41467-018-03828-2)
Supplement: Supplementary file 8 — Supplementary Data 5 [file 41467_2018_3828_MOESM8_ESM.zip › Rmarkdowns/Supplementary Figure 3/supplementary_Figure3_hotspot_mutation_location_rev.html]

Supplementary Figure 3 - Location of mutations in CBS hotspots


# Supplementary Figure 3 - Location of mutations in CBS hotspots

This is the R Markdown for Supplementary Figure 3, which consists of 18 parts.

## Figure A-R

Location of mutations in various hotspots

```
# Each Hotspot Mutation
ctcf.motif=read.table("fimo_all.txt",sep="\t")
unique(ctcf.motif$V4-ctcf.motif$V3)+1 #19
```

```
## [1] 19
```

```
ctcf.motif=GRanges(seqnames=ctcf.motif$V2,IRanges(start=ctcf.motif$V3,end=ctcf.motif$V4),pval=ctcf.motif$V7,qval=ctcf.motif$V8,dir=ctcf.motif$V5,motif=ctcf.motif$V9)
length(ctcf.motif)
```

```
## [1] 1751592
```

```
ctcf.peak=import("CTCF.bw")
sum(ctcf.peak$score>0)
```

```
## [1] 1147323
```

```
dnase.peak=import("E094-DNase.all.peaks.bed")
sum(dnase.peak$score>0)
```

```
## [1] 111721
```

```
dnase.fdr=read.table("E094-DNase.fdr0.01.peaks.bed")
dnase.fdr=GRanges(seqnames=dnase.fdr$V1,IRanges(start=dnase.fdr$V2,end=dnase.fdr$V3))

dnase.mac=read.table("E094-DNase.macs2.narrowPeak")
dnase.mac=GRanges(seqnames=dnase.mac$V1,IRanges(start=dnase.mac$V2,dnase.mac$V3))

dnase.peak=GRanges(seqnames=seqnames(dnase.peak),IRanges(start=start(dnase.peak),end=end(dnase.peak)))
dnase=c(dnase.peak,dnase.fdr,dnase.mac)
dnase=reduce(dnase)
length(dnase)
```

```
## [1] 223139
```

```
z=findOverlaps(ctcf.peak,ctcf.motif) #183666
ctcf.peak.ovl=ctcf.peak[queryHits(z)]
ctcf.peak.ovl=as.data.frame(ctcf.peak.ovl)
ctcf.peak.ovl=unique(ctcf.peak.ovl) #157222
ctcf.motif.ovl=ctcf.motif[subjectHits(z)]
ctcf.motif.ovl=as.data.frame(ctcf.motif.ovl)
ctcf.motif.ovl=unique(ctcf.motif.ovl) #125228
ctcf.motif.ovl=GRanges(seqnames=ctcf.motif.ovl$seqnames,IRanges(start=ctcf.motif.ovl$start,end=ctcf.motif.ovl$end),pval=ctcf.motif.ovl$pval,qval=ctcf.motif.ovl$qval,dir=ctcf.motif.ovl$dir,motif=ctcf.motif.ovl$motif)

z=findOverlaps(dnase,ctcf.motif.ovl) #47454
dnase.ovl=dnase[queryHits(z)]
dnase.ovl=as.data.frame(dnase.ovl)
dnase.ovl=unique(dnase.ovl) #34018
motif.ovl=ctcf.motif.ovl[subjectHits(z)]
motif.ovl=as.data.frame(motif.ovl)
motif.ovl=unique(motif.ovl) #47453

maf.gastric <- maf.to.granges('gastric_RF_prefiltered.MAF')
```

```
## [1] ">> Reading compact MAF ..."
```

```
maf.gastric=maf.gastric[-which(maf.gastric$sid %in% c("tan2001206", "tan20021007", "tan980319", "tan2000986", "tan980436"))] #4119812
maf.gastric$id=c(1:length(maf.gastric))
length(maf.gastric)
```

```
## [1] 4119812
```

```
# Reference Alignment
hotspot <- read.delim("LRmodel_hotspot_nonMSI_prefiltered-5_corrected.tsv", stringsAsFactors=FALSE)
hotspot$mut_region=rownames(hotspot)
hotspot=GRanges(seqnames=hotspot$chrom,IRanges(start=hotspot$start,end=hotspot$end),mut_region=hotspot$mut_region,pval=hotspot$pval,fdr=hotspot$fdr)
hotspot=hotspot[which(hotspot$pval<(0.01/2533374732))]
hotspot=reduce(hotspot)
hotspot$hotspot=c(1:length(hotspot))

motif=GRanges(seqnames=motif.ovl$seqnames,IRanges(start=motif.ovl$start,end=motif.ovl$end),pval=motif.ovl$pval,qval=motif.ovl$qval,dir=motif.ovl$dir,motif=motif.ovl$motif)
motif=motif+5

ovl=findOverlaps(hotspot,motif)
hotspot=hotspot[unique(queryHits(ovl))] #11

hotspot2=GRanges(seqnames=c("chr3","chr4","chr12","chr7","chr10","chr10","chr11"),IRanges(start=c(115533809,10556430,88242208,137139127,108384794,81134501,123349289),end=c(115533827,10556448,88242226,137139145,108384812,81134519,123349307))) #7
hotspot2=hotspot2+5
hotspot2$hotspot=c(1:length(hotspot2))

hotspot=c(hotspot,hotspot2)
hotspot$hotspot=c(1:length(hotspot)) #18

# some other mutations there but not in the hotspots (eg. 4 extra mutations at CBS +-5bp but not in the hotspot)
for (i in 1:length(hotspot)){
#  print(i)
  z=findOverlaps(maf.gastric,hotspot[i])
  maf=maf.gastric[queryHits(z)]
  maf$hotspot=hotspot[subjectHits(z)]$hotspot
  length(unique(maf$id))
  z=findOverlaps(maf,motif)
  maf=maf[queryHits(z)]
  maf=as.data.frame(maf)
  maf$motif.start=start(motif[subjectHits(z)])
  maf$motif.end=end(motif[subjectHits(z)])
  maf$pval=motif[subjectHits(z)]$pval
  maf$dir=motif[subjectHits(z)]$dir
  maf$motif=motif[subjectHits(z)]$motif
  length(unique(maf$id))
  
  maf=maf[order(maf$id,maf$pval),]
  maf=maf[!duplicated(maf$id),]
  maf.pos=maf[which(maf$dir=="+"),]
  maf.neg=maf[which(maf$dir=="-"),]
  
  maf.ref.seq.pos=GRanges(seqnames=maf.pos$seqnames,
                          IRanges(maf.pos$motif.start,maf.pos$motif.end),
                          dir=maf.pos$dir,
                          motif=maf.pos$motif,
                          mut=maf.pos$start,
                          alt=maf.pos$tal,
                          ref=maf.pos$ral)
  extrSeq=Views(Hsapiens,maf.ref.seq.pos)
  
  z=as(extrSeq,"DNAStringSet") 
  dnaSeq.pos.maf=as.data.frame(maf.ref.seq.pos)
  dnaSeq.pos.maf$dna=as.character(z) 
  
  dnaSeq.pos.maf$motif=toupper(dnaSeq.pos.maf$motif)
  sum(dnaSeq.pos.maf$motif==dnaSeq.pos.maf$dna)
  
  dnaSeq.pos.maf$a1=substr(dnaSeq.pos.maf$dna,1,1)
  dnaSeq.pos.maf$a2=substr(dnaSeq.pos.maf$dna,2,2)
  dnaSeq.pos.maf$a3=substr(dnaSeq.pos.maf$dna,3,3)
  dnaSeq.pos.maf$a4=substr(dnaSeq.pos.maf$dna,4,4)
  dnaSeq.pos.maf$a5=substr(dnaSeq.pos.maf$dna,5,5)
  dnaSeq.pos.maf$a6=substr(dnaSeq.pos.maf$dna,6,6)
  dnaSeq.pos.maf$a7=substr(dnaSeq.pos.maf$dna,7,7)
  dnaSeq.pos.maf$a8=substr(dnaSeq.pos.maf$dna,8,8)
  dnaSeq.pos.maf$a9=substr(dnaSeq.pos.maf$dna,9,9)
  dnaSeq.pos.maf$a10=substr(dnaSeq.pos.maf$dna,10,10)
  dnaSeq.pos.maf$a11=substr(dnaSeq.pos.maf$dna,11,11)
  dnaSeq.pos.maf$a12=substr(dnaSeq.pos.maf$dna,12,12)
  dnaSeq.pos.maf$a13=substr(dnaSeq.pos.maf$dna,13,13)
  dnaSeq.pos.maf$a14=substr(dnaSeq.pos.maf$dna,14,14)
  dnaSeq.pos.maf$a15=substr(dnaSeq.pos.maf$dna,15,15)
  dnaSeq.pos.maf$a16=substr(dnaSeq.pos.maf$dna,16,16)
  dnaSeq.pos.maf$a17=substr(dnaSeq.pos.maf$dna,17,17)
  dnaSeq.pos.maf$a18=substr(dnaSeq.pos.maf$dna,18,18)
  dnaSeq.pos.maf$a19=substr(dnaSeq.pos.maf$dna,19,19)
  dnaSeq.pos.maf$a20=substr(dnaSeq.pos.maf$dna,20,20)
  dnaSeq.pos.maf$a21=substr(dnaSeq.pos.maf$dna,21,21)
  dnaSeq.pos.maf$a22=substr(dnaSeq.pos.maf$dna,22,22)
  dnaSeq.pos.maf$a23=substr(dnaSeq.pos.maf$dna,23,23)
  dnaSeq.pos.maf$a24=substr(dnaSeq.pos.maf$dna,24,24)
  dnaSeq.pos.maf$a25=substr(dnaSeq.pos.maf$dna,25,25)
  dnaSeq.pos.maf$a26=substr(dnaSeq.pos.maf$dna,26,26)
  dnaSeq.pos.maf$a27=substr(dnaSeq.pos.maf$dna,27,27)
  dnaSeq.pos.maf$a28=substr(dnaSeq.pos.maf$dna,28,28)
  dnaSeq.pos.maf$a29=substr(dnaSeq.pos.maf$dna,29,29)
  
  maf.ref.seq.neg=GRanges(seqnames=maf.neg$seqnames,
                          IRanges(maf.neg$motif.start,maf.neg$motif.end),
                          dir=maf.neg$dir,
                          motif=maf.neg$motif,
                          mut=maf.neg$start,
                          alt=maf.neg$tal,
                          ref=maf.neg$ral)
  extrSeq=Views(Hsapiens,maf.ref.seq.neg)
  
  z=as(extrSeq,"DNAStringSet") 
  dnaSeq.neg.maf=as.data.frame(maf.ref.seq.neg)
  dnaSeq.neg.maf$dna=as.character(z)
  
  dnaSeq.neg.maf$motif=toupper(dnaSeq.neg.maf$motif)
  sum(dnaSeq.neg.maf$motif==dnaSeq.neg.maf$dna)
  
  z=reverseComplement(z)
  dnaSeq.neg.maf$rev.dna=as.character(z)
  sum(dnaSeq.neg.maf$motif==dnaSeq.neg.maf$rev.dna)
  
  dnaSeq.neg.maf$a1=substr(dnaSeq.neg.maf$rev.dna,1,1)
  dnaSeq.neg.maf$a2=substr(dnaSeq.neg.maf$rev.dna,2,2)
  dnaSeq.neg.maf$a3=substr(dnaSeq.neg.maf$rev.dna,3,3)
  dnaSeq.neg.maf$a4=substr(dnaSeq.neg.maf$rev.dna,4,4)
  dnaSeq.neg.maf$a5=substr(dnaSeq.neg.maf$rev.dna,5,5)
  dnaSeq.neg.maf$a6=substr(dnaSeq.neg.maf$rev.dna,6,6)
  dnaSeq.neg.maf$a7=substr(dnaSeq.neg.maf$rev.dna,7,7)
  dnaSeq.neg.maf$a8=substr(dnaSeq.neg.maf$rev.dna,8,8)
  dnaSeq.neg.maf$a9=substr(dnaSeq.neg.maf$rev.dna,9,9)
  dnaSeq.neg.maf$a10=substr(dnaSeq.neg.maf$rev.dna,10,10)
  dnaSeq.neg.maf$a11=substr(dnaSeq.neg.maf$rev.dna,11,11)
  dnaSeq.neg.maf$a12=substr(dnaSeq.neg.maf$rev.dna,12,12)
  dnaSeq.neg.maf$a13=substr(dnaSeq.neg.maf$rev.dna,13,13)
  dnaSeq.neg.maf$a14=substr(dnaSeq.neg.maf$rev.dna,14,14)
  dnaSeq.neg.maf$a15=substr(dnaSeq.neg.maf$rev.dna,15,15)
  dnaSeq.neg.maf$a16=substr(dnaSeq.neg.maf$rev.dna,16,16)
  dnaSeq.neg.maf$a17=substr(dnaSeq.neg.maf$rev.dna,17,17)
  dnaSeq.neg.maf$a18=substr(dnaSeq.neg.maf$rev.dna,18,18)
  dnaSeq.neg.maf$a19=substr(dnaSeq.neg.maf$rev.dna,19,19)
  dnaSeq.neg.maf$a20=substr(dnaSeq.neg.maf$rev.dna,20,20)
  dnaSeq.neg.maf$a21=substr(dnaSeq.neg.maf$rev.dna,21,21)
  dnaSeq.neg.maf$a22=substr(dnaSeq.neg.maf$rev.dna,22,22)
  dnaSeq.neg.maf$a23=substr(dnaSeq.neg.maf$rev.dna,23,23)
  dnaSeq.neg.maf$a24=substr(dnaSeq.neg.maf$rev.dna,24,24)
  dnaSeq.neg.maf$a25=substr(dnaSeq.neg.maf$rev.dna,25,25)
  dnaSeq.neg.maf$a26=substr(dnaSeq.neg.maf$rev.dna,26,26)
  dnaSeq.neg.maf$a27=substr(dnaSeq.neg.maf$rev.dna,27,27)
  dnaSeq.neg.maf$a28=substr(dnaSeq.neg.maf$rev.dna,28,28)
  dnaSeq.neg.maf$a29=substr(dnaSeq.neg.maf$rev.dna,29,29)
  
  df12=rbind(dnaSeq.pos.maf[,12:40],dnaSeq.neg.maf[,13:41])
  df=matrix(0,nrow=4,ncol=29)
  rownames(df)=c("A","C","G","T")
  for (j in 1:29){
#    print(j)
    df[names(table(df12[,j])),j]=as.numeric(table(df12[,j]))
  }
  colnames(df)=colnames(df12)

  # Alternate
if(nrow(dnaSeq.pos.maf)!=0 & nrow(dnaSeq.neg.maf)!=0){
  dnaSeq.mut.pos.maf=dnaSeq.pos.maf
  dnaSeq.mut.pos.maf$alt=as.character(dnaSeq.mut.pos.maf$alt)
  dnaSeq.mut.pos.maf$num=dnaSeq.mut.pos.maf$mut-dnaSeq.mut.pos.maf$start+1
  for (p in 1:nrow(dnaSeq.mut.pos.maf)){
    dnaSeq.mut.pos.maf[p,paste("a",dnaSeq.mut.pos.maf[p,"num"],sep="")]<-dnaSeq.mut.pos.maf[p,"alt"]
  }
  
  dnaSeq.mut.neg.maf=dnaSeq.neg.maf
  dnaSeq.mut.neg.maf$alt=as.character(dnaSeq.mut.neg.maf$alt)
  dnaSeq.mut.neg.maf$num=dnaSeq.mut.neg.maf$end-dnaSeq.mut.neg.maf$mut+1
  for (q in 1:nrow(dnaSeq.mut.neg.maf)){
    dnaSeq.mut.neg.maf[q,paste("a",dnaSeq.mut.neg.maf[q,"num"],sep="")]<-as.character(reverseComplement(DNAString(dnaSeq.mut.neg.maf[q,"alt"])))
  }
  
  df13=rbind(dnaSeq.mut.pos.maf[,12:40],dnaSeq.mut.neg.maf[,13:41])
} else if(nrow(dnaSeq.pos.maf)!=0){
  dnaSeq.mut.pos.maf=dnaSeq.pos.maf
  dnaSeq.mut.pos.maf$alt=as.character(dnaSeq.mut.pos.maf$alt)
  dnaSeq.mut.pos.maf$num=dnaSeq.mut.pos.maf$mut-dnaSeq.mut.pos.maf$start+1
  for (p in 1:nrow(dnaSeq.mut.pos.maf)){
    dnaSeq.mut.pos.maf[p,paste("a",dnaSeq.mut.pos.maf[p,"num"],sep="")]<-dnaSeq.mut.pos.maf[p,"alt"]
  }
  
  df13=dnaSeq.mut.pos.maf[,12:40]
  
} else if (nrow(dnaSeq.neg.maf)!=0){
  dnaSeq.mut.neg.maf=dnaSeq.neg.maf
  dnaSeq.mut.neg.maf$alt=as.character(dnaSeq.mut.neg.maf$alt)
  dnaSeq.mut.neg.maf$num=dnaSeq.mut.neg.maf$end-dnaSeq.mut.neg.maf$mut+1
  for (q in 1:nrow(dnaSeq.mut.neg.maf)){
    dnaSeq.mut.neg.maf[q,paste("a",dnaSeq.mut.neg.maf[q,"num"],sep="")]<-as.character(reverseComplement(DNAString(dnaSeq.mut.neg.maf[q,"alt"])))
  }
  
  df13=dnaSeq.mut.neg.maf[,13:41]
  
  }
  
  df=matrix(0,nrow=4,ncol=29)
  rownames(df)=c("A","C","G","T")
  for (r in 1:29){
   # print(r)
    df[names(table(df13[,r])),r]=as.numeric(table(df13[,r]))
  }
  colnames(df)=colnames(df13)

  if (nrow(dnaSeq.pos.maf)!=0 & nrow(dnaSeq.neg.maf)!=0){
    df=rbind(dnaSeq.mut.pos.maf[,c("ref","alt","num")],dnaSeq.mut.neg.maf[,c("ref","alt","num")])
  } else if (nrow(dnaSeq.pos.maf)!=0){
    df=dnaSeq.mut.pos.maf[,c("ref","alt","num")]
  } else if (nrow(dnaSeq.neg.maf)!=0){
    df=dnaSeq.mut.neg.maf[,c("ref","alt","num")]
  }
  df$count=1
  df=aggregate(count~ref+alt+num,df,sum)
  
  for (s in 1:nrow(df)){
    if (!df$ref[s] %in% c("C","T")){
      df$alt[s]=as.character(reverseComplement(DNAString(df$alt[s])))
      df$ref[s]=as.character(reverseComplement(DNAString(df$ref[s])))
    }
  }
  df$mut=paste(df$ref,df$alt,sep="")
  df$mut=factor(df$mut,levels=c("TG","TC","TA","CT","CG","CA"))
  df$num=factor(df$num,levels=c(1:29))
  df=aggregate(count~ref+alt+num+mut,df,sum)
  
  dff=data.frame(sub=0,num=c(1:29))
  df=merge(df,dff,by="num",all.y=TRUE)
  df$sub=ifelse(is.na(df$count),0,df$count)
  
  t=ggplot(df,aes(x=num,y=sub,fill=mut))+
    geom_bar(stat="identity",colour="black")+
    theme(panel.grid.major = element_blank(),
          panel.grid.minor = element_blank(),
          panel.background = element_blank(),
          axis.line = element_line(colour="black"))+
    scale_fill_manual(values = c("#33CC00","#33CCFF","#FF9933","#CC33FF","#FFFF33","#FF0000"))+
    ggtitle(paste(seqnames(hotspot[i]),start(hotspot[i]),end(hotspot[i]),sep=","))
  plot(t)
}
```
