# Supplementary material for: Mutation hotspots at CTCF binding sites coupled to chromosomal instability in gastrointestinal cancers
Source: Nat Commun. 2018 Apr 18;9:1520. doi: 10.1038/s41467-018-03828-2 (PMC5906695; doi:10.1038/s41467-018-03828-2)
Supplement: Supplementary file 8 — Supplementary Data 5 [file 41467_2018_3828_MOESM8_ESM.zip › Rmarkdowns/Supplementary Figure 4/Supplementary_Figure4_correlation_mutrate_mutsig_rev.html]

Supplementary Figure 4


# Supplementary Figure 4

This is the R Markdown for Supplementary Figure 4, which consists of 1 part.

## Figure A

Correlation between proportions of CBS mutations and COSMIC mutation signatures

```
### all samples
## get ctcf mutations
maf.gastric <- maf.to.granges('gastric_RF_prefiltered.MAF')
```

```
## [1] ">> Reading compact MAF ..."
```

```
maf.gastric=maf.gastric[-which(maf.gastric$sid %in% c("tan2001206", "tan20021007", "tan980319", "tan2000986", "tan980436"))] #4119812
maf.gastric=maf.gastric[seqnames(maf.gastric)!="chrY"]
maf.gastric=maf.gastric[-which(maf.gastric$tal=="FALSE")]
length(maf.gastric) #4116297
```

```
## [1] 4116297
```

```
length(unique(maf.gastric$sid)) #187
```

```
## [1] 187
```

```
# read in ctcf 
motif.ovl=bed.to.granges("ctcf_motif_union.bed")

# overlap maf.gastric with ctcf to get only ctcf mutations
z=findOverlaps(maf.gastric,motif.ovl)
ctcf.muts=maf.gastric[unique(queryHits(z))]
length(unique(ctcf.muts$sid))
```

```
## [1] 164
```

```
ctcf.muts=as.data.frame(ctcf.muts)
mut.count.sample=aggregate(seqnames~sid,ctcf.muts,length) #1809 2
colnames(mut.count.sample)[2]="count"
mut.count.sample$sid=as.character(mut.count.sample$sid)

df=data.frame(sid=unique(maf.gastric$sid))
df$sid=as.character(df$sid)
mut.count.sample=merge(df,mut.count.sample,by="sid",all.x=TRUE)
mut.count.sample[is.na(mut.count.sample$count),"count"]=0
```

```
## corr with mut sig
# COSMIC mutation signatures
z <- maf.to.granges('gastric_RF_prefiltered.MAF')
z=z[seqnames(z)!="chrY"]
z=z[-which(z$tal=="FALSE")]
length(z) #4139877
length(unique(z$sid)) #192, includes the 5 samples

z=as.data.frame(z)
colnames(z)=c("chr","pos","end","width","strand","ref","alt","Sample","batch")
z=z[,c("Sample","chr","pos","ref","alt")]
sigs.input <- mut.to.sigs.input(mut.ref = z, 
                                sample.id = "Sample", 
                                chr = "chr", 
                                pos = "pos", 
                                ref = "ref", 
                                alt = "alt",
                                bsg = BSgenome.Hsapiens.UCSC.hg19) #192 96

mutsig=as.list(numeric(nrow(sigs.input)))
names(mutsig)=rownames(sigs.input)
for (i in 1:nrow(sigs.input)){
  print(i)
  mutsig[[i]]=whichSignatures(tumor.ref = sigs.input,
                              signatures.ref = signatures.cosmic,
                              sample.id = rownames(sigs.input)[i],
                              contexts.needed = TRUE,
                              tri.counts.method = 'default')
}

sigs=matrix(nrow=192,ncol=31)
sigs=as.data.frame(sigs)
rownames(sigs)=names(mutsig)
colnames(sigs)=c(paste("Signature.",c(1:30),sep=""),"Signature.unknown")

for (i in colnames(sigs)[-ncol(sigs)]){
  print(i)
  sigs[,i]=unlist(lapply(mutsig,FUN=function(x) {
    x$weights[i]
  }))
}

sigs$Signature.unknown=unlist(lapply(mutsig,FUN=function(x){
  x$unknown
}))

# save summarized output to table
write.table(sigs,"mutsig_gastric.txt",col.names=TRUE,row.names=TRUE,quote=FALSE,sep="\t")
```

Read in summarized table

```
sigs=read.delim("mutsig_gastric.txt",header=TRUE,stringsAsFactors = FALSE)
```

```
# cor between counts and mut sig weights
corr=numeric(31)
names(corr)=colnames(sigs)
for (i in 1:ncol(sigs)){
  corr[i]=suppressWarnings(cor.test(mut.count.sample$count,sigs[mut.count.sample$sid,i])$estimate)
}

# cor between prop counts and mut sig weights
df=table(as.character(maf.gastric$sid)) #187
df=as.data.frame(df)
colnames(df)=c("sid","total.count")
df$sid=as.character(df$sid)
mut.prop.sample=merge(df,mut.count.sample,by="sid",all.x=TRUE)
mut.prop.sample$prop=mut.prop.sample$count/mut.prop.sample$total.count

corr3=numeric(31)
names(corr3)=colnames(sigs)
for (i in 1:ncol(sigs)){
  corr3[i]=suppressWarnings(cor.test(mut.prop.sample$prop,sigs[mut.prop.sample$sid,i])$estimate)
}

df1=as.data.frame(corr3)
df1$sig=rownames(df1)
df1[is.na(df1$corr3),"corr3"]=0

df1$sig=as.character(df1$sig)
df1$sig=factor(df1$sig,levels=c(paste("Signature.",c(1:30),sep=""),"Signature.unknown"))

print(ggplot(df1,aes(x=sig,y=corr3))+geom_bar(stat="identity",position = "dodge")+ theme(axis.text.x = element_text(angle = 90, hjust = 1))+
  xlab("COSMIC signatures")+ylab(paste("Correlation between proportion of CBS mutations", "and mutation signatures",sep="\n"))+
  theme(panel.grid.major = element_blank(),
        panel.grid.minor = element_blank(),
        panel.background = element_blank(),
        axis.line = element_line(colour="black"))+
  geom_hline(yintercept = 0))
```
