# Supplementary material for: Mutation hotspots at CTCF binding sites coupled to chromosomal instability in gastrointestinal cancers
Source: Nat Commun. 2018 Apr 18;9:1520. doi: 10.1038/s41467-018-03828-2 (PMC5906695; doi:10.1038/s41467-018-03828-2)
Supplement: Supplementary file 8 — Supplementary Data 5 [file 41467_2018_3828_MOESM8_ESM.zip › Rmarkdowns/Supplementary Figure 5/Supplementary_Figure5_scatter_hotspots_significance_rev.html]

Supplementary Figure 5 - Scatterplot representation of hotspot significance


# Supplementary Figure 5 - Scatterplot representation of hotspot significance

This is the R Markdown for Supplementary Figure 5, which consists of 4 parts.

## Figure A

SNV hotspots Number of mutated samples vs pvalues

```
hotspots=read.delim("snv_hotspot_edited.txt") # unique hotspots
hotspots$lg=-log10(hotspots$pval)
hotspots$candidate=ifelse(hotspots$pval<(0.01/2533374732),"1","0")
ctcf=read.table("ctcf_motif_union.bed")
ctcf=with(ctcf,GRanges(V1,IRanges(V2,V3)))
hotspots=with(hotspots,GRanges(seqnames,IRanges(start,end),pval=pval,length=length,p.bg=p.bg,k=k,fdr=fdr,lg=lg,candidate=candidate))
hotspots$cbs=numeric(length(hotspots))
hotspots[unique(queryHits(findOverlaps(hotspots,ctcf)))]$cbs="1"
hotspots$type=ifelse(hotspots$candidate=="1" & hotspots$cbs=="1","1","0")
hotspots=as.data.frame(hotspots)
ggplot(hotspots,aes(x=k,y=lg,col=type))+geom_point()+
    theme(panel.grid.major = element_blank(),
        panel.grid.minor = element_blank(),
        panel.background = element_blank(),
        axis.line = element_line(colour="black"))+
        scale_color_manual(values=c("black","maroon1"))+
  geom_hline(yintercept=-log10(0.01/2533374732))
```

## Figure B

CBS hotspots Number of mutated samples vs pvalues

```
hotspots2=read.delim("ctcf_specific_edited.txt")
hotspots2$lg=-log10(hotspots2$pval)
hotspots2=with(hotspots2,GRanges(seqnames,IRanges(start,end),pval=pval,length=length,p.bg=p.bg,k=k,fdr=fdr,lg=lg))

hotspots=hotspots[which(hotspots$type=="1"),]
hotspots=with(hotspots,GRanges(seqnames,IRanges(start,end)))

hotspots2$candidate=numeric(length(hotspots2))
hotspots2[unique(queryHits(findOverlaps(hotspots2,hotspots)))]$candidate="1"
hotspots2=as.data.frame(hotspots2)
hotspots2$candidate=factor(hotspots2$candidate)

ggplot(hotspots2,aes(x=k,y=lg,col=candidate))+geom_point()+
    theme(panel.grid.major = element_blank(),
        panel.grid.minor = element_blank(),
        panel.background = element_blank(),
        axis.line = element_line(colour="black"))+
        scale_color_manual(values=c("black","maroon1"))+
  geom_hline(yintercept=-log10(0.01/47453))
```

## Figure C

Indel hotspots Number of mutated samples vs pvalues

```
hotspots=read.delim("indel_hotspot_edited.txt")
hotspots$lg=-log10(hotspots$pval)
hotspots$candidate=ifelse(hotspots$pval<(0.01/2533374732),"1","0")
ggplot(hotspots,aes(x=k,y=lg,col=candidate))+geom_point()+
    theme(panel.grid.major = element_blank(),
        panel.grid.minor = element_blank(),
        panel.background = element_blank(),
        axis.line = element_line(colour="black"))+
        scale_color_manual(values=c("black","orange"))+
  geom_hline(yintercept=-log10(0.01/2533374732))
```

## Figure D

Indel gene region Number of mutated samples vs pvalues

```
hotspots=read.delim("LRmodel_gene_region_nonMSI_indel_remove5_edited_annotated_min1.tsv")
hotspots$lg=-log10(hotspots$pval)
hotspots$candidate=ifelse(hotspots$Associated.Gene.Name %in% c("LIPF","PGC","MUC6"),"1","0")
ggplot(hotspots,aes(x=k,y=lg,col=candidate))+geom_point()+
    theme(panel.grid.major = element_blank(),
        panel.grid.minor = element_blank(),
        panel.background = element_blank(),
        axis.line = element_line(colour="black"))+
        scale_color_manual(values=c("black","orange"))+
  geom_hline(yintercept=-log10(0.01/33786))
```
