# Supplementary material for: Mutation hotspots at CTCF binding sites coupled to chromosomal instability in gastrointestinal cancers
Source: Nat Commun. 2018 Apr 18;9:1520. doi: 10.1038/s41467-018-03828-2 (PMC5906695; doi:10.1038/s41467-018-03828-2)
Supplement: Supplementary file 8 — Supplementary Data 5 [file 41467_2018_3828_MOESM8_ESM.zip › Rmarkdowns/Supplementary Figure 9/supplementary_Figure9_sg_expression_rev.html]

Supplementary Figure 3 - SG expression


# Supplementary Figure 3 - SG expression

This is the R Markdown for Supplementary Figure 3, which consists of 3 parts.

## Figure A-C

Boxplots for SG expression

```
## Overlap each hotspot with maf.gastric (prefiltered, both MSI and nonMSI) to identify
## mutated samples in hotspots
maf.gastric <- maf.to.granges('gastric_RF_prefiltered.MAF')
```

```
## [1] ">> Reading compact MAF ..."
```

```
# 4143709, 192 unique sids
maf.gastric=maf.gastric[-which(maf.gastric$sid %in% c("tan2001206", "tan20021007", "tan980319", "tan2000986", "tan980436"))] # 4119812, 187 unique sids

hotspot <- read.delim("LRmodel_hotspot_nonMSI_prefiltered-5_corrected.tsv", stringsAsFactors=FALSE)
hotspot$mut_region=rownames(hotspot)
hotspot=GRanges(seqnames=hotspot$chrom,IRanges(start=hotspot$start,end=hotspot$end),mut_region=hotspot$mut_region,pval=hotspot$pval,fdr=hotspot$fdr)
hotspot=hotspot[which(hotspot$pval<(0.01/2533374732))]
hotspot=reduce(hotspot)
hotspot$hotspot=c(1:length(hotspot))

## TAN expression data
rnaseq.tan=read.table("gene_fpkm.txt", header=T, sep="\t", check.names=F)
rnaseq.tan=rnaseq.tan[,-1]
rnaseq.tan=rnaseq.tan[,-which(colnames(rnaseq.tan) %in% c("T2001206", "T20021007", "T980319", "T2000986", "T980436",
                                                          "N2001206", "N20021007", "N980319", "N2000986", "N980436"))]

rnaseq.tan.boxplot <- function(gene, mut.samples, rnaseq.data) {
  gene.rnaseq=rnaseq.data[rnaseq.data[,1]==gene,]
  gene.rnaseq=(gene.rnaseq[-1])
  gene.df=data.frame(id=names(gene.rnaseq),exp=as.numeric(gene.rnaseq), group=c(rep("Normal",14),rep("Tumor_WT",14)),stringsAsFactors =F)
  gene.df$group[names(gene.rnaseq)%in%mut.samples]="Tumor_Mut"
  print(gene.df[gene.df$group=="Tumor_Mut",])
  print(paste("median of Tumor_Mut: ",median(gene.df$exp[gene.df$group=="Tumor_Mut"])))
  print(paste("median of Tumor_WT: ",median(gene.df$exp[gene.df$group=="Tumor_WT"])))
  print(paste("median of Normal: ",median(gene.df$exp[gene.df$group=="Normal"])))
  print(wilcox.test(gene.df$exp[gene.df$group=="Tumor_Mut"],gene.df$exp[gene.df$group=="Tumor_WT"]))
  print(wilcox.test(gene.df$exp[gene.df$group=="Tumor_WT"],gene.df$exp[gene.df$group=="Normal"]))
  if (max(gene.df$exp[gene.df$group=="Tumor_Mut"])<1&&max(gene.df$exp[gene.df$group=="Tumor_WT"])<1) {
    print(paste(gene," not expressed"))
  }  
  p=ggplot(gene.df, aes(x=factor(group, levels = c("Normal","Tumor_WT","Tumor_Mut")), y=exp))+ stat_boxplot(geom ='errorbar')+geom_boxplot(outlier.shape = NA)+
    geom_jitter(aes(colour=group), size=2, width=0.1)+ scale_color_brewer(palette="Paired")+ylab(paste(gene,"fpkm"))+xlab(NULL)+
    theme(text = element_text(size=20),axis.text.x = element_text(size=20))+theme(legend.position="none")+
    theme(panel.grid.major = element_blank(),
          panel.grid.minor = element_blank(),
          panel.background = element_blank(),
          axis.line = element_line(colour="black"))
  print(p)
}
```

## Figure A

CENPQ

```
z=findOverlaps(maf.gastric,hotspot[1])
maf=maf.gastric[queryHits(z)] # 14
sid=unique(maf$sid) # 12

CENPQ.mut=c("T76629543")
rnaseq.tan.boxplot("CENPQ", CENPQ.mut, rnaseq.tan)
```

```
##           id     exp     group
## 17 T76629543 9.00158 Tumor_Mut
## [1] "median of Tumor_Mut:  9.00158"
## [1] "median of Tumor_WT:  5.04728"
## [1] "median of Normal:  2.862805"
## 
##  Wilcoxon rank sum test
## 
## data:  gene.df$exp[gene.df$group == "Tumor_Mut"] and gene.df$exp[gene.df$group == "Tumor_WT"]
## W = 12, p-value = 0.2857
## alternative hypothesis: true location shift is not equal to 0
## 
## 
##  Wilcoxon rank sum test
## 
## data:  gene.df$exp[gene.df$group == "Tumor_WT"] and gene.df$exp[gene.df$group == "Normal"]
## W = 141, p-value = 0.01448
## alternative hypothesis: true location shift is not equal to 0
```

## Figure B

SPG20

```
z=findOverlaps(maf.gastric,hotspot[30])
maf=maf.gastric[queryHits(z)] # 8
sid=unique(maf$sid) # 8

SPG20.mut=c("T76629543")
rnaseq.tan.boxplot("SPG20", SPG20.mut, rnaseq.tan)
```

```
##           id     exp     group
## 17 T76629543 1.36257 Tumor_Mut
## [1] "median of Tumor_Mut:  1.36257"
## [1] "median of Tumor_WT:  9.62506"
## [1] "median of Normal:  18.82735"
## 
##  Wilcoxon rank sum test
## 
## data:  gene.df$exp[gene.df$group == "Tumor_Mut"] and gene.df$exp[gene.df$group == "Tumor_WT"]
## W = 0, p-value = 0.1429
## alternative hypothesis: true location shift is not equal to 0
## 
## 
##  Wilcoxon rank sum test
## 
## data:  gene.df$exp[gene.df$group == "Tumor_WT"] and gene.df$exp[gene.df$group == "Normal"]
## W = 49, p-value = 0.04265
## alternative hypothesis: true location shift is not equal to 0
```

## Figure C

KCNQ5

```
z=findOverlaps(maf.gastric,hotspot[2])
maf=maf.gastric[queryHits(z)] # 9
sid=unique(maf$sid) # 9

KCNQ5.mut=c("T2000639")
rnaseq.tan.boxplot("KCNQ5", KCNQ5.mut, rnaseq.tan)
```

```
##          id        exp     group
## 23 T2000639 0.00922566 Tumor_Mut
## [1] "median of Tumor_Mut:  0.00922566"
## [1] "median of Tumor_WT:  0.209007"
## [1] "median of Normal:  0.319851"
## 
##  Wilcoxon rank sum test
## 
## data:  gene.df$exp[gene.df$group == "Tumor_Mut"] and gene.df$exp[gene.df$group == "Tumor_WT"]
## W = 1, p-value = 0.2857
## alternative hypothesis: true location shift is not equal to 0
## 
## 
##  Wilcoxon rank sum test
## 
## data:  gene.df$exp[gene.df$group == "Tumor_WT"] and gene.df$exp[gene.df$group == "Normal"]
## W = 56, p-value = 0.09448
## alternative hypothesis: true location shift is not equal to 0
## 
## [1] "KCNQ5  not expressed"
```
