# Supplementary material for: Mutation hotspots at CTCF binding sites coupled to chromosomal instability in gastrointestinal cancers
Source: Nat Commun. 2018 Apr 18;9:1520. doi: 10.1038/s41467-018-03828-2 (PMC5906695; doi:10.1038/s41467-018-03828-2)
Supplement: Supplementary file 8 — Supplementary Data 5 [file 41467_2018_3828_MOESM8_ESM.zip › Rmarkdowns/Table/Supplementary_Table1_meta_data_epigenetic_features_rev.html]

Supplementary Table 1-2 - Meta Data and Epigentic Features


# Supplementary Table 1-2 - Meta Data and Epigentic Features

This is the Rmarkdown for Supplementary Table 1-2.

## Table 1

Meta data

```
meta <- read.delim("gastric_consolidated_meta.tsv", stringsAsFactors=FALSE)
# choose columns to include
meta=meta[,which(!colnames(meta) %in% c("Alias","Molecular.Subtype.original","Laurens.Classification.original","Tumor.Site.original",
                                       "mutect","freebayes","vardict","varscan","atleast.1","atleast.2","atleast.3","atleast.4","Ratio",
                                       "Freebayes_Indels","Vardict_Indels","Varscan_Indels","Atleast.1.caller","Atleast.2.callers",
                                       "X3.callers","Ratio.1"))]
colnames(meta)[26]<-"SNV Mutation Count"
colnames(meta)[27]<-"Indel Mutation Count"

# replace apollo1 with apollo1_new
meta_new <- read.delim("gastric_consolidated_meta_new.tsv", stringsAsFactors = FALSE)

meta[which(meta$Sample.ID=="apollo1"),"SNV Mutation Count"]<-meta_new[which(meta_new$Sample.ID=="apollo1_new"),"ML"]
meta[which(meta$Sample.ID=="apollo1"),"Indel Mutation Count"]<-meta_new[which(meta_new$Sample.ID=="apollo1_new"),"ML_Indels"]

meta[which(meta$Sample.ID=="apollo1"),"Sample.ID"]<-"apollo1_new"
print(head(meta))
```

```
##     Sample.ID Status Molecular.Subtype Laurens.Classification Tumor.Site
## 1 apollo1_new     OK                GS             intestinal     Cardia
## 2    apollo10     OK                GS                  mixed Middle 1/3
## 3    apollo11     OK               CIN             intestinal Middle 1/3
## 4    apollo12     OK               CIN             intestinal Distal 1/3
## 5    apollo13     OK                GS             intestinal  Upper 1/3
## 6    apollo14     OK               EBV             intestinal Middle 1/3
##   Sex Age Stage Stage_T Stage_N Stage_M RNAseq.data histone.data
## 1      NA                            NA           0            0
## 2      NA                            NA           0            0
## 3      NA                            NA           0            0
## 4      NA                            NA           0            0
## 5      NA                            NA           0            0
## 6      NA                            NA           0            0
##   WGS.tumor..mapped.reads..M. WGS.tumor..mapped.fraction....
## 1                      1367.1                           98.1
## 2                      1336.1                           98.1
## 3                      1403.1                           97.1
## 4                      1454.1                           98.1
## 5                      1484.1                           97.1
## 6                      1346.1                           97.1
##   WGS.tumor..dupl.fraction.... WGS.tumor..read.length WGS.tumor..coverage
## 1                          1.1                    100                40.1
## 2                          1.1                    100                40.1
## 3                          1.1                    100                41.1
## 4                          1.1                    100                43.1
## 5                          1.1                    100                44.1
## 6                          1.1                    100                40.1
##   WGS.normal..mapped.reads..M. WGS.normal..mapped.fraction....
## 1                       1488.1                            98.1
## 2                       1348.1                            97.1
## 3                       1294.1                            98.1
## 4                       1417.1                            98.1
## 5                       1468.1                            97.1
## 6                       1344.1                            98.1
##   WGS.normal..dupl.fraction.... WGS.normal..read.length
## 1                           1.1                     100
## 2                           1.1                     100
## 3                           1.1                     100
## 4                           1.1                     100
## 5                           1.1                     100
## 6                           1.1                     100
##   WGS.normal..coverage WGS.mean.coverage Batch SNV Mutation Count
## 1                 44.1              42.1    SG               6744
## 2                 40.1              40.1    SG              18458
## 3                 38.1              39.6    SG              14299
## 4                 42.1              42.6    SG              13330
## 5                 43.1              43.6    SG               8044
## 6                 40.1              40.1    SG              22392
##   Indel Mutation Count
## 1                  175
## 2                 1111
## 3                  693
## 4                  650
## 5                  557
## 6                   66
```

```
# write output to excel file
write.csv(meta,file="Supplementary_Table1.csv",quote=FALSE)
```

## Table 2

Epigenetic features for lasso selection

```
feat.site=readRDS('feat.matrix.all_nonMSI_sites_indels_prefiltered_median_poly_v2.rds')
feat.site=feat.site[,-c(10:15,70:75, 77:101)]

features=colnames(feat.site)
features=features[-1]
features=data.frame(features)
print(head(features))
```

```
##         features
## 1  local.mutrate
## 2  mean.rep.time
## 3 H3K27Ac_normal
## 4 H3K4Me1_normal
## 5 H3K4Me3_normal
## 6  H3K27Ac_tumor
```

```
# write output to text file
write.table(features,file="features.txt",quote=FALSE,row.names=FALSE,col.names=TRUE)
```
