# Supplementary material for: Mutation hotspots at CTCF binding sites coupled to chromosomal instability in gastrointestinal cancers
Source: Nat Commun. 2018 Apr 18;9:1520. doi: 10.1038/s41467-018-03828-2 (PMC5906695; doi:10.1038/s41467-018-03828-2)
Supplement: Supplementary file 8 — Supplementary Data 5 [file 41467_2018_3828_MOESM8_ESM.zip › Rmarkdowns/Table/supplementary_Table3_5_analysis_results_rev.html]

Supplementary Table 3-5 - Analysis Results


# Supplementary Table 3-5 - Analysis Results

This is the R Markdown document for Supplementary Table 3-5. List of unique hotspots results from the 4 analysis; SNV hotspot, indel hotspot, indel gene recurrence and CTCF specific models.

## Table 5

CTCF specific

```
hotspot_candidates <- read.delim("LRmodel_CTCF_union_nonMSI_subtype-5_mutsigs.tsv", stringsAsFactors=FALSE) #2018
print(head(hotspot_candidates))
```

```
##           seqnames    start      end         pval length         p.bg  k
## CTCF37970     chr6 50570087 50570105 1.323750e-14     29 0.0017738416 11
## CTCF42916     chr8 70576154 70576172 2.313179e-14     29 0.0004729119  8
## CTCF42919     chr8 71000980 71000998 8.646835e-14     29 0.0005584217  8
## CTCF12929    chr14 70285590 70285608 4.752653e-13     29 0.0003699613  7
## CTCF37969     chr6 50570085 50570103 1.218353e-12     29 0.0019161790 10
## CTCF24457     chr2 49173790 49173808 4.483090e-11     29 0.0012332557  8
##                    fdr
## CTCF37970 5.488365e-10
## CTCF42916 5.488365e-10
## CTCF42919 1.367728e-09
## CTCF12929 5.638191e-09
## CTCF37969 1.156291e-08
## CTCF24457 3.545601e-07
```

```
# create file for supplementary figure 5
hotspot_candidates=with(hotspot_candidates,GRanges(seqnames,IRanges(start,end),pval=pval,p.bg=p.bg,k=k,fdr=fdr))
hotspot_candidates=hotspot_candidates+5

hotspot=reduce(hotspot_candidates) #1807
hotspot$hotspot=c(1:length(hotspot))
z=findOverlaps(hotspot_candidates,hotspot)
t=as.data.frame(hotspot_candidates[queryHits(z)])
t$hotspot=hotspot[subjectHits(z)]$hotspot
t$reduce.seqnames=as.character(seqnames(hotspot[subjectHits(z)]))
t$reduce.start=start(hotspot[subjectHits(z)])
t$reduce.end=end(hotspot[subjectHits(z)])

t=t[order(t$pval,decreasing=FALSE),] #2018
q=t[!duplicated(t$hotspot),] #1807
hs=with(q,GRanges(seqnames,IRanges(reduce.start,reduce.end),pval=pval,p.bg=p.bg,k=k,fdr=fdr))

maf.gastric <- maf.to.granges('gastric_RF_nonMSI_prefiltered.MAF') #2444729
```

```
## [1] ">> Reading compact MAF ..."
```

```
maf.gastric=maf.gastric[-which(maf.gastric$sid %in% c("tan2001206", "tan20021007", "tan980319", "tan2000986", "tan980436"))] #2420832
maf.gastric$mut=c(1:length(maf.gastric))

z=findOverlaps(hs,maf.gastric) #2387
hs=hs[queryHits(z)]
hs$mut=maf.gastric[subjectHits(z)]$mut
hs$sid=maf.gastric[subjectHits(z)]$sid
hs=as.data.frame(hs)
nrow(unique(hs[,1:9])) #1807
```

```
## [1] 1807
```

```
hs1=aggregate(sid~seqnames+start+end+width+strand+pval+p.bg+k+fdr,hs,function(x) length(unique(x))) #1807
sum(hs1$k==hs1$sid) #1806
```

```
## [1] 1806
```

```
hs1=hs1[,c("seqnames","start","end","pval","width","p.bg","sid","fdr")]
colnames(hs1)[5]<-"length"
colnames(hs1)[7]<-"k"
hs1=hs1[order(hs1$pval,decreasing=FALSE),]
print(head(hs1))
```

```
##   seqnames     start       end         pval length         p.bg  k
## 2     chr6  50570080  50570110 1.323750e-14     31 0.0017738416 11
## 1     chr8  70576149  70576177 2.313179e-14     29 0.0004729119  8
## 3     chr8  71000975  71001003 8.646835e-14     29 0.0005584217  8
## 4    chr14  70285585  70285613 4.752653e-13     29 0.0003699613  7
## 5     chr2  49173785  49173813 4.483090e-11     29 0.0012332557  8
## 6     chrX 104435103 104435131 3.212833e-10     29 0.0009486426  7
##            fdr
## 2 5.488365e-10
## 1 5.488365e-10
## 3 1.367728e-09
## 4 5.638191e-09
## 5 3.545601e-07
## 6 2.177979e-06
```

```
# write output to excel file
write.table(hs1,file="ctcf_specific_edited.txt",row.names=TRUE,col.names=TRUE,quote=FALSE,sep="\t")
```

## Table 3

Indel hotspot

```
hotspot_candidates <- read.delim("LRmodel_hotspot_indel_nonMSI_prefiltered_poly_v2_remove5_corrected.tsv", stringsAsFactors=FALSE) #62893
hotspot_candidates=with(hotspot_candidates,GRanges(chrom,IRanges(start,end),pval=pval,p.bg=p.bg,k=k,fdr=fdr))

hotspot=reduce(hotspot_candidates) #62739
hotspot$hotspot=c(1:length(hotspot))
z=findOverlaps(hotspot_candidates,hotspot)
t=as.data.frame(hotspot_candidates[queryHits(z)])
t$hotspot=hotspot[subjectHits(z)]$hotspot
t$reduce.seqnames=as.character(seqnames(hotspot[subjectHits(z)]))
t$reduce.start=start(hotspot[subjectHits(z)])
t$reduce.end=end(hotspot[subjectHits(z)])

t=t[order(t$pval,decreasing=FALSE),] #62893
q=t[!duplicated(t$hotspot),] #62739
hs=with(q,GRanges(seqnames,IRanges(reduce.start,reduce.end),pval=pval,p.bg=p.bg,k=k,fdr=fdr))

maf.gastric <- maf.to.granges('gastric_RF_indels_nonMSI_prefiltered_noPoly_v2.MAF') #64514
```

```
## [1] ">> Reading compact MAF ..."
```

```
maf.gastric=maf.gastric[-which(maf.gastric$sid %in% c("tan2001206", "tan20021007", "tan980319", "tan2000986", "tan980436"))] #64363
maf.gastric$mut=c(1:length(maf.gastric))

z=findOverlaps(hs,maf.gastric) #63164
hs=hs[queryHits(z)]
hs$mut=maf.gastric[subjectHits(z)]$mut
hs$sid=maf.gastric[subjectHits(z)]$sid
hs=as.data.frame(hs)
nrow(unique(hs[,1:9])) #62739
```

```
## [1] 62739
```

```
hs1=aggregate(sid~seqnames+start+end+width+strand+pval+p.bg+k+fdr,hs,function(x) length(unique(x))) #62739
sum(hs1$k==hs1$sid) #62723
```

```
## [1] 62723
```

```
hs1=hs1[,c("seqnames","start","end","pval","width","p.bg","sid","fdr")]
colnames(hs1)[5]<-"length"
colnames(hs1)[7]<-"k"
hs1=hs1[order(hs1$pval,decreasing=FALSE),]
print(head(hs1))
```

```
##   seqnames     start       end         pval length         p.bg k
## 1     chr6 168136120 168136140 6.446386e-16     21 2.133639e-06 4
## 2     chr6  41709379  41709409 1.934078e-14     31 4.994021e-06 4
## 3     chr7  15339441  15339469 3.261472e-11     29 3.493836e-06 3
## 4     chr1 216970886 216970906 8.828096e-11     21 4.109631e-05 4
## 5    chr12 102863053 102863073 4.766799e-10     21 8.544028e-06 3
## 6     chr8  12640952  12640972 1.391545e-09     21 1.221287e-05 3
##            fdr
## 1 1.633111e-06
## 2 2.449872e-05
## 3 1.377088e-02
## 4 3.194982e-02
## 5 1.509511e-01
## 6 3.917005e-01
```

```
# write output to excel file
write.table(hs1,file="indel_hotspot_edited.txt",row.names=TRUE,col.names=TRUE,quote=FALSE,sep="\t")
```

Indel gene recurrence

```
results <- read.delim("LRmodel_gene_region_nonMSI_indel_remove5_edited_annotated_min1.tsv", stringsAsFactors=FALSE) #9741
print(head(results))
```

```
##                id         pval length         p.bg  k          fdr
## 1 ENSG00000182333 1.892170e-17  14807 0.0040810449 16 6.395724e-13
## 2 ENSG00000096088 6.166189e-08  16717 0.0020162384  7 1.042117e-03
## 3 ENSG00000184956 4.919971e-07  17851 0.0040567333  8 5.543332e-03
## 4 ENSG00000267093 4.711702e-06   2418 0.0001846803  3 3.981506e-02
## 5 ENSG00000090382 4.557391e-05   6908 0.0011411620  4 3.080888e-01
## 6 ENSG00000143153 9.557405e-05  27119 0.0043107480  6 5.384164e-01
##   Chromosome.scaffold.name Gene.Start..bp. Gene.End..bp.
## 1                       10        90424198      90438571
## 2                        6        41704449      41721847
## 3                       11         1012821       1036706
## 4                       19        55911113      55911787
## 5                       12        69742121      69748014
## 6                        1       169074935     169101960
##   Associated.Gene.Name      Gene.type
## 1                 LIPF protein_coding
## 2                  PGC protein_coding
## 3                 MUC6 protein_coding
## 4       CTD-2105E13.13        lincRNA
## 5                  LYZ protein_coding
## 6               ATP1B1 protein_coding
```

```
# write output to excel file
write.table(results[which(results$fdr!=1),],file="indel_gene.txt",row.names=FALSE,col.names=TRUE,quote=FALSE,sep="\t")
```

## Table 4

SNV hotspot

```
hotspot_candidates <- read.delim("LRmodel_hotspot_nonMSI_prefiltered-5_corrected.tsv", stringsAsFactors=FALSE) #103627
hotspot_candidates=with(hotspot_candidates,GRanges(chrom,IRanges(start,end),pval=pval,p.bg=p.bg,k=k,fdr=fdr))

hotspot=reduce(hotspot_candidates) #64988
hotspot$hotspot=c(1:length(hotspot))
z=findOverlaps(hotspot_candidates,hotspot)
t=as.data.frame(hotspot_candidates[queryHits(z)])
t$hotspot=hotspot[subjectHits(z)]$hotspot
t$reduce.seqnames=as.character(seqnames(hotspot[subjectHits(z)]))
t$reduce.start=start(hotspot[subjectHits(z)])
t$reduce.end=end(hotspot[subjectHits(z)])

t=t[order(t$pval,decreasing=FALSE),] #103627
q=t[!duplicated(t$hotspot),] #64988
hs=with(q,GRanges(seqnames,IRanges(reduce.start,reduce.end),pval=pval,p.bg=p.bg,k=k,fdr=fdr))

maf.gastric <- maf.to.granges('gastric_RF_nonMSI_prefiltered.MAF')
```

```
## [1] ">> Reading compact MAF ..."
```

```
maf.gastric=maf.gastric[-which(maf.gastric$sid %in% c("tan2001206", "tan20021007", "tan980319", "tan2000986", "tan980436"))] #2420832
maf.gastric$mut=c(1:length(maf.gastric))

z=findOverlaps(hs,maf.gastric) #136454
hs=hs[queryHits(z)]
hs$mut=maf.gastric[subjectHits(z)]$mut
hs$sid=maf.gastric[subjectHits(z)]$sid
hs=as.data.frame(hs)
nrow(unique(hs[,1:9])) #64988
```

```
## [1] 64988
```

```
hs1=aggregate(sid~seqnames+start+end+width+strand+pval+p.bg+k+fdr,hs,function(x) length(unique(x))) #64988
sum(hs1$k==hs1$sid) #64831
```

```
## [1] 64831
```

```
hs1=hs1[,c("seqnames","start","end","pval","width","p.bg","sid","fdr")]
colnames(hs1)[5]<-"length"
colnames(hs1)[7]<-"k"
hs1=hs1[order(hs1$pval,decreasing=FALSE),]
print(head(hs1))
```

```
##   seqnames     start       end         pval length         p.bg  k
## 1     chr6  50570094  50570120 5.403225e-23     27 0.0002929608 11
## 3     chr7  68391104  68391132 8.362901e-19     29 0.0002539073  9
## 2     chr8  71000992  71001012 1.085230e-18     21 0.0001326314  8
## 4     chr7 136495924 136495948 6.731174e-17     25 0.0004144562  9
## 5     chr2  57627616  57627640 1.317567e-16     25 0.0002420961  8
## 6     chr1 209422184 209422222 1.936060e-16     39 0.0001186775  7
##            fdr
## 1 6.705654e-14
## 3 3.436618e-10
## 2 3.436618e-10
## 4 1.894732e-08
## 5 2.967051e-08
## 6 3.438354e-08
```

```
# write output to excel file
write.table(hs1,file="snv_hotspot_edited.txt",row.names=FALSE,col.names=TRUE,quote=FALSE,sep="\t")
```
